# Circulating metabolites associated with kidney function decline and incident CKD: a multi-platform population-based study

**DOI:** 10.1093/ckj/sfad286

**Published:** 2023-11-16

**Authors:** Anna C van der Burgh, Sven Geurts, Shahzad Ahmad, M Arfan Ikram, Layal Chaker, Pietro Manuel Ferraro, Mohsen Ghanbari

**Affiliations:** Department of Epidemiology, Erasmus University Medical Center, Rotterdam, The Netherlands; Department of Epidemiology, Erasmus University Medical Center, Rotterdam, The Netherlands; Department of Epidemiology, Erasmus University Medical Center, Rotterdam, The Netherlands; Department of Epidemiology, Erasmus University Medical Center, Rotterdam, The Netherlands; Department of Epidemiology, Erasmus University Medical Center, Rotterdam, The Netherlands; Department of Internal Medicine, Erasmus University Medical Center, Rotterdam, The Netherlands; Division of Nephrology, Department of Medicine, Università degli Studi di Verona, Verona, Italy; Department of Epidemiology, Erasmus University Medical Center, Rotterdam, The Netherlands

**Keywords:** albuminuria, chronic kidney disease, glomerular filtration rate, kidney function, metabolomics

## Abstract

**Background:**

Investigation of circulating metabolites associated with kidney function and chronic kidney disease (CKD) risk could enhance our understanding of underlying pathways and identify new biomarkers for kidney function.

**Methods:**

We selected participants from the population-based Rotterdam Study with data on circulating metabolites and estimated glomerular filtration rate based on serum creatinine (eGFRcreat) available at the same time point. Data on eGFR based on serum cystatin C (eGFRcys) and urine albumin-to-creatinine ratio (ACR) were also included. CKD was defined as eGFRcreat <60 ml/min per 1.73 m^2^. Data on circulating metabolites (n_total_ = 1381) was obtained from the Nightingale and Metabolon platform. Linear regression, linear mixed, and Cox proportional-hazards regression analyses were conducted to study the associations between metabolites and kidney function. We performed bidirectional two-sample Mendelian randomization analyses to investigate causality of the identified associations.

**Results:**

We included 3337 and 1540 participants with data from Nightingale and Metabolon, respectively. A total of 1381 metabolites (243 from Nightingale and 1138 from Metabolon) were included in the analyses. A large number of metabolites were significantly associated with eGFRcreat, eGFRcys, ACR, and CKD, including 16 metabolites that were associated with all four outcomes. Among these, C-glycosyltryptophan (HR 1.50, 95%CI 1.31;1.71) and X-12026 (HR 1.46, 95%CI 1.26;1.68) were most strongly associated with CKD risk. We revealed sex differences in the associations of 11-ketoetiocholanolone glucuronide and 11-beta-glucuronide with the kidney function assessments. No causal associations between the identified metabolites and kidney function were observed.

**Conclusion:**

Our study indicates that several circulating metabolites are associated with kidney function which are likely to have potential as biomarkers, rather than as molecules involved in the pathophysiology of kidney function decline.

KEY LEARNING POINTS **What was known**:

- There is a need for improving chronic kidney disease (CKD) prevention, as the burden of CKD is expected to increase in the upcoming years.
- Current kidney function biomarkers have several limitations, as they can be affected by factors other than kidney function.
- Therefore, identification of additional biomarkers is needed to better estimate kidney function especially in clinical practice, which could improve early detection of CKD.

**This study adds**:

- In the current study, we reveal that 16 metabolites are significantly associated with estimated glomerular filtration rate (eGFR) based on serum creatinine, eGFR based on serum cystatin C, urine albumin-to-creatinine ratio, and CKD.
- Glycosyltryptophan and X-12026 had the highest HRs for CKD.
- No evidence for causal associations between the identified metabolites and kidney function was found.

**Potential impact**:

- Circulating metabolites that were associated with kidney function were more likely to be disease biomarkers, rather than molecules involved in the pathophysiology of kidney function decline.
- The identified metabolites might have potential as new biomarkers for kidney function, which should be a focus of future studies.

## INTRODUCTION

Chronic kidney disease (CKD) is an umbrella term covering a wide range of heterogeneous disorders causing progressive alterations in kidney function and structure [1]. Prevention of CKD can be achieved by managing classical CKD risk factors, including hypertension, diabetes, and obesity [2–4]. Efforts have been made to develop prediction models that can be used for the identification of individuals at a higher risk for developing CKD [5, 6]. In addition, screening for CKD in high-risk individuals has been advised [6, 7]. However, despite all efforts made to prevent CKD, the burden of CKD is expected to increase even further in the upcoming years [8–10]. This highlights the need for improving CKD prevention, and obtaining more insights into the currently incompletely understood biological and pathophysiological pathways underlying CKD development and identification of new biomarkers for kidney function and its decline is therefore crucial.

Metabolomics has emerged as a promising molecular approach to investigate etiology and to identify biomarkers that could eventually provide insight into the pathogenesis of complex diseases. Moreover, circulating metabolites in plasma are relatively easy to measure and therefore have many potential uses, including population screening. Hence, investigating the metabolites potentially involved in kidney function decline in individuals without CKD may help to understand the molecular pathways underlying CKD development. Moreover, investigation of circulating metabolites may also lead to the identification of new potential biomarkers for CKD. Currently, serum creatinine is the most commonly used biomarker to detect kidney function decline in clinical practice [11], although levels of serum creatinine can be affected by factors other than kidney function, such as changes in muscle mass [12–14] and in dietary habits [13–15]. Other filtration markers such as serum cystatin C have therefore gained more interest, although serum cystatin C can also be affected by factors other than kidney function, such as inflammation and diabetes [16, 17]. Therefore, identification of additional biomarkers is needed to better estimate kidney function in clinical practice, which could improve early detection of CKD.

In the current study, we aimed to identify new biomarkers for kidney function, including testing causal association, using metabolomics data. In addition, we aimed to unravel potential pathophysiological pathways underlying CKD development. To do so, we included a wide-range of metabolites across two commonly used metabolomics platforms as well as different and multiple assessments of kidney function from a prospective population-based cohort study.

## MATERIALS AND METHODS

### Study design and population

This study was embedded within the Rotterdam Study, an ongoing prospective population-based cohort study designed to investigate the occurrence and determinants of age-related diseases in the general population. Further details regarding the objectives and design of the study have been reported previously [18] and a more detailed description of the study design and population included in this study can be found in Methods S1 (see online supplementary material).

### Assessment of plasma metabolites and kidney function

Detailed information on the assessment of plasma metabolites and kidney function can be found in Methods S2 (see online supplementary material). In short, metabolites were measured using the Nightingale and the Metabolon platform. The Nightingale platform enables simultaneous quantification of 249 lipoprotein subclasses and metabolites including amino acids, ketone bodies, and gluconeogenesis-related metabolites, of which 39 are clinically validated. The metabolites present in this platform were quantified using fasted ethylenediaminetetraacetic acid (EDTA) plasma samples by high-throughput proton nuclear magnetic resonance (NMR) metabolomics (Nightingale Health Ltd, Helsinki, Finland). After pre-processing and QC, the remaining metabolites from this platform included in our study were *n* = 243. Furthermore, 1387 circulating metabolites were available from the Metabolon platform. These metabolites are from different biochemical pathways, including lipids, amino acids, xenobiotics, nucleotides, cofactors and vitamins, peptides, carbohydrates, energy-related metabolites, and uncharacterized metabolites. The metabolites present in this platform were quantified using mass spectrometry (MS) technique (developed by Metabolon, Inc.). After pre-processing and QC, the remaining metabolites from this platform included in our study were n = 1138 (for more details see Methods S2, see online supplementary material). Serum creatinine measurements were performed using an enzymatic assay method and expressed in µmol/L [19]. Measurements of serum creatinine from the Rotterdam Study were supplemented with measurements from the Star-MDC database [20]. Serum cystatin C measurements were performed using particle-enhanced immunonephelometric assay and expressed in mg/L. Both eGFRcreat and eGFRcys were calculated according to the Chronic Kidney Disease Epidemiology Collaboration (CKD-EPI) formula [21, 22], without incorporation of race [23]. Incident CKD was defined as the first follow-up eGFR assessment <60 ml/min per 1.73 m^2^. Four alternative definitions of incident CKD and/or CKD progression were considered as well, in order to exclude cases of acute kidney disease (Methods S2, see online supplementary material). These include: (i) two consecutive eGFRcreat measurements <60 ml/min per 1.73 m^2^; (ii) a single eGFRcreat <45 ml/min/1.73 m^2^; (iii) eGFRcreat <60 ml/min per 1.73 m^2^ determined with eGFRcreat slope; and (iv) the composite outcome of 40% loss of eGFRcreat or kidney failure. The slope of eGFRcreat is defined as the individual slope of the participants, determined by all available measurements of the participant (median of five assessments per participant in the total population of the Rotterdam Study with at least one eGFRcreat assessment (n_participants_ = 12 062; n_assessments_ = 85 922)), calculated using a linear mixed effects model and reported in ml/min per 1.73 m^2^. Incident CKD is then defined as the first time eGFRcreat drops <60 ml/min per 1.73 m^2^, a time point which is calculated using baseline eGFRcreat and the eGFRcreat slope. Urine albumin-to-creatinine ratio (ACR) was calculated by dividing urine albumin by urine creatinine (mg/g). Information on data collection of covariates (including age, sex, Rotterdam Study Cohort, body mass index, smoking status, alcohol use, serum cholesterol, lipid-lowering drugs, prevalent cardiovascular disease, hypertension, and diabetes) can be found in the Methods S3 (see online supplementary material).

### Genome-wide association studies for circulating metabolites and kidney function

Genome-wide significant variants were selected as instrumental variables for circulating metabolites and kidney function from the largest available genome-wide association studies (GWAS) including participants from European ancestry, if available. Further details can be found in the Methods S4–S6 (see online supplementary material).

### Statistical analyses

All analyses were performed for participants with data available from the Nightingale and Metabolon platforms separately, after which results were shown together. Circulating metabolites were transformed using a natural log-transformation (after adding one unit to the non-transformed values) and were scaled to a mean of zero and a standard deviation (SD) of 1. The urine ACR was naturally log-transformed after adding 1 mg/g to all ACR values before transformation to account for zero values. A false discovery rate (FDR)-corrected *P*-value < 0.05 was set as the significance threshold in this study. Linear regression analyses were conducted to study the cross-sectional associations between circulating metabolites and eGFRcreat, eGFRcys, and urine ACR, separately per batch or Rotterdam Study cohort, after which results were meta-analysed using the inverse variance weighted method. Predefined stratification by sex were performed with (i) the circulating metabolites that were significantly associated with all three kidney function assessments at baseline; and (ii) the sex-hormone related circulating metabolites. Longitudinal analyses were conducted with metabolites that were significantly associated with all three kidney function assessments at baseline. Linear mixed models were used to study the associations between circulating metabolites and repeated assessments of eGFRcreat over time. Cox proportional-hazards models were used to study the associations between metabolites and time to incident CKD. In analyses with CKD as the outcome, we excluded participants with prevalent CKD. All cross-sectional and longitudinal analyses were adjusted for (i) age, sex, and Rotterdam Study sub-cohort; (ii) model 1 + the potential confounders (which were selected based on available literature) body mass index (BMI), smoking status, alcohol use, serum cholesterol, lipid-lowering drugs, and prevalent cardiovascular disease; and (iii) model 2 + hypertension and type 2 diabetes. Analyses with eGFRcys as the outcome were additionally adjusted for the time difference between the cystatin C measurement and the metabolite measurements. When investigating the association between circulating metabolites and CKD longitudinally, we additionally adjusted the third model for baseline eGFR as an extra analysis. As sensitivity analyses, we investigated the association between circulating metabolites and the four alternative CKD definitions. Further details on the used statistical methods can be found in the Methods S7 (see online supplementary material). The Mendelian randomization (MR) analyses together with the assumptions and sensitivity analyses are described in detail in the Methods S8 (see online supplementary material).

## RESULTS

In total, we included 3337 and 1540 participants with data on kidney function and circulating metabolites available from the Nightingale and Metabolon platform, respectively (Table 1). These samples show partial overlap, meaning that a participant could have data on circulating metabolites from both platforms. The mean ± SD age of the 3337 participants included for analyses regarding metabolites from the Nightingale platform was 68.6 ± 8.8 years and 58.2% were women. The mean (SD) age of the 1540 participants included for analyses regarding metabolites from the Metabolon platform was 66.9 ± 8.5 years and 56.8% were women. The mean ± SD values of eGFRcreat were 74 ± 16 and 76 ± 16 ml/min per 1.73 m^2^ and mean values for eGFRcys were 79 ± 17 and 82 ± 17 ml/min per 1.73 m^2^, for the Nightingale and Metabolon populations, respectively. The median (interquartile range (IQR)) of ACR was 3.61 (1.97;8.43) mg/g for the Nightingale population versus 3.40 (1.97;7.20) mg/g for the Metabolon population.

**Table 1:** Baseline: characteristics of the total study population.

	Total population Nightingale platform (*n* = 3337)	Total population Metabolon platform (*n* = 1540)
Age, years (*n* = 3337; *n* = 1540)	68.6 ± 8.8	66.9 ± 8.5
Female sex, *n* (%) (*n* = 3337; *n* = 1540)	1942 (58.2)	874 (56.8)
Body mass index, kg/m^2^ (*n* = 3283; *n* = 1539)	27.5 ± 4.4	27.5 ± 4.4
Systolic blood pressure, mmHg (*n* = 3324; *n* = 1537)	143 ± 22	140 ± 22
Diastolic blood pressure, mmHg (*n* = 3324; *n* = 1537)	80 ± 11	81 ± 11
Hypertension, *n* (valid%) (*n* = 3326; *n* = 1537)	2350 (70.7)	1028 (66.9)
Diabetes, *n* (%) (*n* = 3211; *n* = 1508)	453 (14.1)	194 (12.9)
History of CVD, n (valid%) (*n* = 3288; *n* = 1524)	415 (12.6)	135 (8.9)
Smoking (*n* = 3336; *n* = 1540)		
Current smoking, *n* (valid%)	423 (12.7)	204 (13.2)
Past smoking, *n* (valid%)	1781 (53.4)	810 (52.6)
Never smoking, *n* (valid%)	1132 (33.9)	526 (34.2)
Alcohol use, g/day (*n* = 3085; *n* = 1413)	6.4 (1.4;15.0)	7.4 (1.6;15.0)
eGFRcreat ml/min per 1.73 m^2^ (*n* = 3337; 1908)	74 ± 16	76 ± 16
eGFRcys ml/min per 1.73 m^2^ (*n* = 3091; 1452)	79 ± 17	82 ± 17
ACR, mg/g (*n* = 2510; *n* = 1290)	3.61 (1.97;8.43)	3.40 (1.97;7.20)
Serum cholesterol, mmol/L (*n* = 3333; *n* = 1539)	5.56 ± 1.04	5.57 ± 1.08
Use of lipid-lowering drugs, *n* (valid %) (*n* = 3331; *n* = 1908)	857 (25.7)	385 (25.0)

The table shows baseline characteristics of the study population with the two metabolomics platforms. Data are presented as number (%), number (valid%), mean ± standard deviation, or median (interquartile range). Values are shown for non-imputed data. For variables with missing data, valid % is given. Of the 1540 participants with Metabolon data, 1513 also have data from the Nightingale platform. Abbreviations: ACR: albumin-to-creatinine ratio; CVD: cardiovascular disease; eGFRcreat: estimated glomerular filtration rate (eGFR) based on serum creatinine; eGFRcys: eGFR based on serum cystatin C; *n*: number.

### Cross-sectional analyses

**Figure 1:**
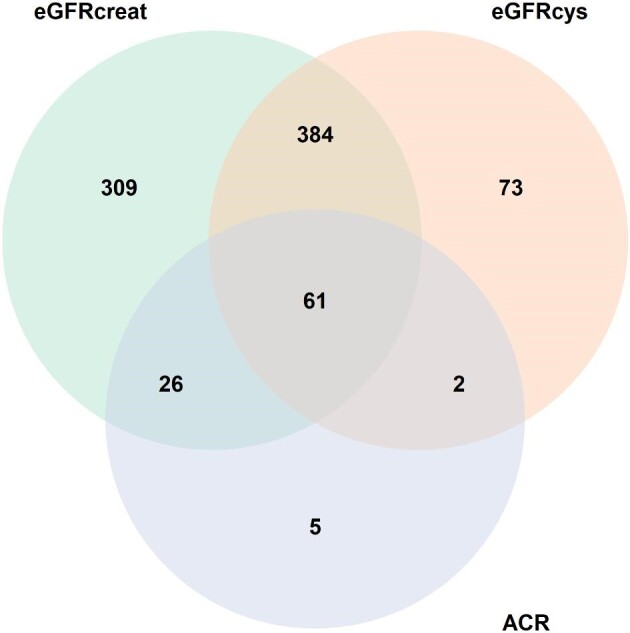
Venn diagram representing the number of metabolites significantly associated with kidney function assessments. This Venn diagram indicates the number of circulating metabolites significantly associated with eGFRcreat, eGFRcys, and ACR.P-values were FDR-corrected. ACR: albumin-to-creatinine ratio; eGFRcreat: estimated glomerular filtration rate (eGFR) based on serum creatinine; eGFRcys: eGFR based on serum cystatin C.

**Figure 2:**
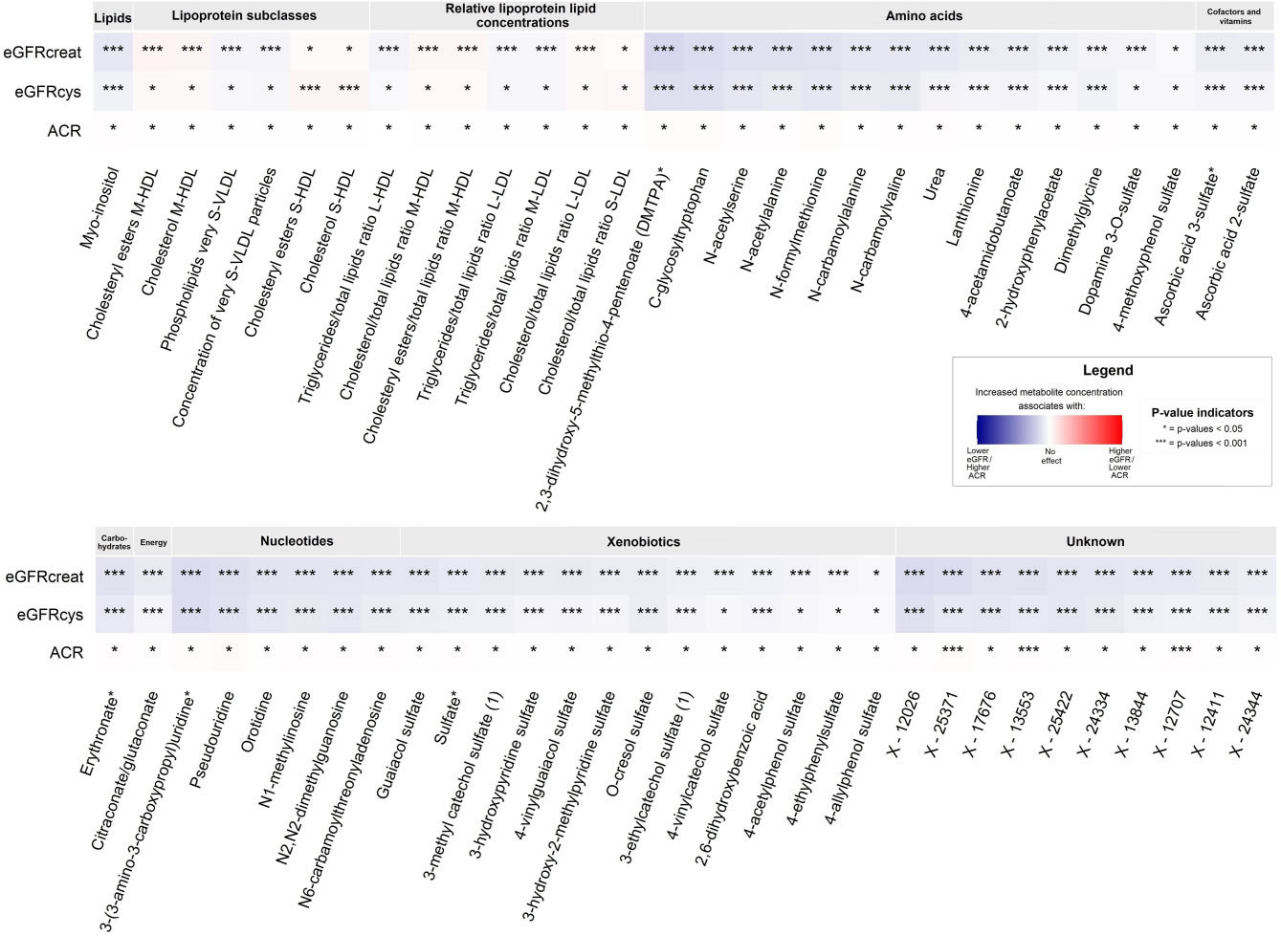
Overview of the metabolites significantly associated with all three kidney function assessments in cross-sectional analysis. The figure shows metabolites that are significantly associated with all three kidney function assessments. Adjusted for age, sex, Rotterdam Study Cohort, body mass index, smoking status, alcohol use, serum cholesterol, lipid-lowering drugs, prevalent cardiovascular disease, hypertension and diabetes. *P*-values are FDR-corrected. Abbreviations: ACR: albumin-to-creatinine ratio; eGFRcreat: estimated glomerular filtration rate (eGFR) based on serum creatinine; eGFRcys: eGFR based on serum cystatin C.

In the cross-sectional analyses, 780 circulating metabolites from both platforms were significantly associated with eGFRcreat (Tables S1 and S2, see online supplementary material) and 520 circulating metabolites were significantly associated with eGFRcys (Tables S3 and S4). In addition, 94 circulating metabolites were significantly associated with ACR (Tables S5 and S6). The Venn diagram on the overlap between the significantly associated metabolites and the three kidney function assessments is shown in Fig. 1. Of these, 61 metabolites were associated with all three kidney function assessments and were therefore considered as the most robust kidney function-associated metabolites (Figs 1 and 2). For eGFRcreat, the two strongest associated metabolites based on the effect estimate were 2,3-dihydroxy-5-methylthio-4-pentenoate (DMTPA) (beta −6.48, 95% CI −7.11; −5.86) and X-12026 (beta −5.89, 95% CI −6.54; −5.24). For eGFRcys, the two strongest associated metabolites were 3-(3-amino-3-carboxypropyl)uridine (beta −5.48, 95% CI −6.11; −4.85) and C-glycosyltryptophan (beta −4.93, 95% CI −5.57; −4.29). For ACR, the two strongest associated metabolites were two yet unknown circulating metabolites: X-12707 (beta 0.28, 95% CI −0.17;0.38) and X-13553 (beta 0.35, 95% CI 0.21;0.49). Among the known circulating metabolites, the first two that were most strongly associated with ACR were cholesterol to total lipids ratio in large LDL (beta −0.10, 95% CI −0.15; −0.05) and 3-(3-amino-3-carboxypropyl)uridine (beta 0.38, 95% CI 0.21;0.56).

### Longitudinal analyses

**Figure 3:**
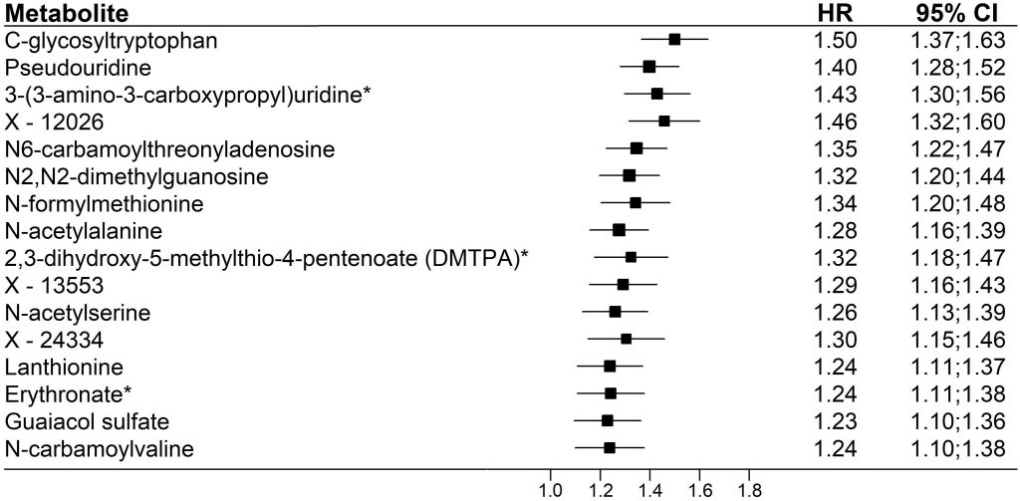
Overview of the 16 metabolites significantly associated with incident CKD. This figure shows metabolites that are significantly associated with incident CKD (N Nightingale = 779; N Metabolon = 287). Participants with prevalent CKD (N Nightingale = 187; N Metabolon = 60) were excluded. Adjusted for baseline eGFRcreat, age, sex, Rotterdam Study Cohort, body mass index, smoking status, alcohol use, serum cholesterol, lipid-lowering drugs, prevalent cardiovascular disease, hypertension and diabetes. Abbreviations: CKD: chronic kidney disease; eGFRcreat: estimated glomerular filtration rate (eGFR) based on serum creatinine; HR: hazard ratio.

Longitudinal assessments of eGFRcreat were collected during a median follow-up time of 2.0 years (interquartile range (IQR) 0;8.7). In the longitudinal analysis, metabolites most strongly associated with baseline eGFRcreat (Table S8) were also strongly associated in analyses of repeated measures of eGFRcreat over time (Table S7). In total, 779 individuals (24.7%) from the Nightingale population and 287 individuals (19.4%) from the Metabolon population were identified as having incident CKD, defined as the first follow-up eGFR assessment <60 ml/min per 1.73 m^2^. Median time to incident CKD was 5.4 years (IQR 3.9;8.2) in the Nightingale population and 5.2 years (IQR 4.0;7.6) in the Metabolon population. Cox proportional hazard regression analysis with CKD as the outcome revealed that 58 of the 61 metabolites that were robustly associated with all three kidney function assessments (eGFRcreat, eGFRcys, and ACR) at baseline were also significantly associated with CKD (Fig. S1, see online supplementary material). The three metabolites that were significantly associated with eGFRcreat, eGFRcys, and ACR at baseline, but not with CKD included cholesterol to total lipids ratio in small LDL, 4-methoxyphenol sulfate, and 4-allylphenol sulfate. Among the metabolites associated with CKD, the highest hazard ratio was shown for DMPTA (HR 2.07, 95% CI 1.84;2.33), followed by X-12026 (HR 2.02, 95% CI 1.78;2.28). (Fig. S1, see online supplementary material). When further adjusting the analyses for baseline eGFRcreat, only 16 metabolites remained significantly associated with CKD, with C- glycosyltryptophan (HR 1.50, 95% CI 1.31;1.71) and X-12026 (HR 1.46, 95% CI 1.26;1.68) having the highest HRs (Fig. 3). In total, 27 metabolites were associated with at least one of the CKD-related outcomes, with similar HRs for all outcomes, showing that the associations remain with sustained low eGFR (Table S9, see online supplementary material).

### Stratification analyses

**Figure 4:**
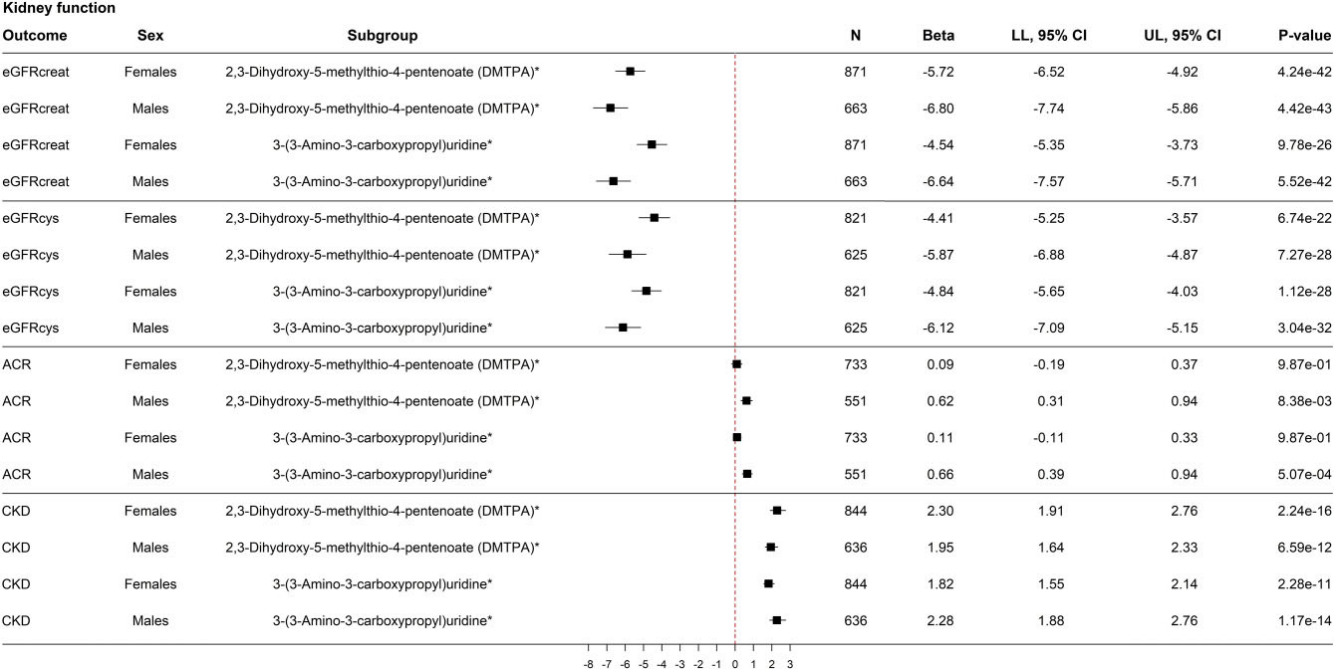
Stratified analyses for men and women, for the two metabolites most significantly associated with the three kidney function measurements. Adjusted for age, sex, Rotterdam Study Cohort, body mass index, smoking status, alcohol use, serum cholesterol, lipid-lowering drugs, prevalent cardiovascular disease, hypertension, and diabetes. *P*-values are FDR-corrected. Abbreviations: ACR: albumin-to-creatinine ratio; CI: confidence interval; eGFRcreat: estimated glomerular filtration rate (eGFR) based on serum creatinine; eGFRcys: eGFR based on serum cystatin C; CKD: chronic kidney disease; LL: lower limit; N: number; UL: upper limit.

Stratification analyses by sex were performed in two steps. First, stratification analyses by sex were performed for the previously identified 61 metabolites significantly associated with all three kidney function assessments. When stratifying these analyses by sex, a similar pattern in the associations between circulating metabolites and the three kidney function assessments was shown (Fig. S2, see online supplementary material). Generally, a stronger negative association between the serum metabolite and eGFRcreat or eGFRcys was shown in men compared to women. Similarly, a stronger positive association between the serum metabolite and ACR was shown in men compared to women, while the sample size was lower in men compared to women. Results of the stratification analyses for the two most significant metabolites were shown in Fig. 4, for which the patterns were most pronounced. When stratifying the analyses by sex with CKD as the outcome, no specific patterns could be identified.

**Figure 5:**
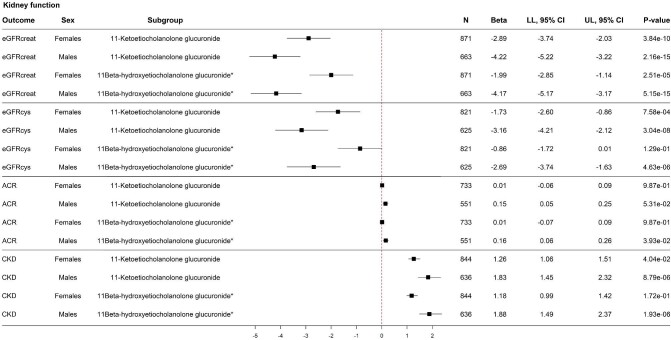
Stratified analyses for men and women, for the two most significantly associated sex hormone-related metabolites. Adjusted for age, sex, Rotterdam Study Cohort, body mass index, smoking status, alcohol use, serum cholesterol, lipid-lowering drugs, prevalent cardiovascular disease, hypertension, and diabetes. P-values are FDR-corrected. Abbreviations: ACR: albumin-to-creatinine ratio; CI: confidence interval; CKD: chronic kidney disease; eGFRcreat: estimated glomerular filtration rate (eGFR) based on serum creatinine; eGFRcys: eGFR based on serum cystatin C; LL: lower limit; N: number; UL: upper limit.

Second, stratification analyses by sex were performed for the sex-hormone related metabolites. When stratifying these analyses by sex, a similar pattern was shown (Fig. S3, see online supplementary material), with a stronger negative association between the serum metabolites and eGFRcreat or eGFRcys and a stronger positive association between the serum metabolites and ACR, both in men compared to women. Contrasting, a similar pattern was identified with CKD as the outcome, where higher HRs and more significant results were reported in men compared to women. Results of the stratification analyses for the two most significant metabolites were shown in Fig. 5.

### Mendelian randomization analyses

For the 16 metabolites that were significantly associated with eGFRcreat, eGFRcys, and ACR at baseline and with CKD, we performed MR analyses to assess the directionality and causality of the results. The number of selected genome-wide significant variants for all metabolites is shown in Fig. S4 (see online supplementary material). As the number of genome-wide significant variants was too low when using a GWAS *P*-value threshold of <5e-8, a less stringent *P*-value threshold of <1e-5 was included as the final threshold. All selected genetic variants had a F-statistic >10.

We found evidence for an association between genetically determined N-carbamoylvaline and N-formylmethionine with genetically determined eGFRcreat when using the IVW method, but not when using the WME and/or MR-Egger method (Fig. S5, see online supplementary material). In addition, we found evidence for an association between genetically determined acetylalanine, C-glycosyltryptophan, pseudouridine, and X-24334 with genetically determined eGFRcreat when using the IVW method, but only with the genetic variants selected from the GWAS by Yin *et al*. Similarly, no association was found when using the WME and/or the MR-Egger methods (Fig. S5, see online supplementary material).

As the number of identified genetic variants for eGFRcys was insufficient for MR analysis when using the GWAS by Li *et al.* (Table S4, see online supplementary material), we selected the GWAS by Gorski *et al.* for the main analysis. In the analyses with genetically determined eGFRcys, ACR, and CKD as outcomes, we found evidence for associations between several genetically determined metabolites using one of the MR methods, but none of the associations were consistently reported with all three MR methods (Figs S6–S8, see online supplementary material).

## DISCUSSION

In this prospective population-based cohort study, we investigated the association between circulating metabolites and kidney function and identified 61 common metabolites significantly associated with eGFRcreat, eGFRcys, and ACR at baseline. Of these, 16 metabolites were also longitudinally associated with CKD after adjustment for baseline eGFRcreat. Our findings did not support a causal association between any of the investigated metabolites and kidney function with the currently available genetic data, but we showed the potential of circulating metabolites as biomarkers of kidney function decline. Furthermore, we observed sex-differences in the associations between serum metabolite levels and the different kidney outcomes, which may highlight the importance of sex when assessing metabolites in CKD and may also suggest a potential explanatory role of metabolites on the sex differences in CKD.

Several previous studies have investigated the link between metabolites and kidney function [24–30]. However, these studies were heterogeneous in their study design, the included study population, the included kidney function assessments, and also the number of metabolites, complicating the comparison of their findings with one another and with the current study. More specifically, previous studies did not include ACR as an outcome. According to the ‘Kidney Disease: Improving Global Outcomes’ (KDIGO) 2012 guideline, CKD should be defined and classified by using eGFR as a marker of glomerular filtration and ACR as a marker of glomerular damage [31, 32]. In the current study, we included ACR as a marker of kidney function next to the assessments of eGFR and showed that fewer metabolites were significantly associated with ACR compared to the number of metabolites significantly associated with eGFR. The explanation for these findings might be found within the nature of the different kidney function assessments, e.g. marker of glomerular filtration vs. marker of glomerular damage. This highlights the need for combining the findings with both eGFR and ACR as outcomes in order to get a more comprehensive view on the association between circulating metabolites and CKD. In the current study, we also included metabolites from the two most frequently used metabolomics platforms, i.e. Nightingale and Metabolon, generating a total of nearly 1400 metabolites. This resulted in a wide range of metabolites covering different metabolic pathways which were not all included in the previous studies and could therefore provide a more comprehensive picture of metabolic pathways associated with kidney function.

When investigating the association between circulating metabolites and kidney function, the potential bidirectional nature of their association could introduce the important issue of reverse causation. One approach to test the direction of associations is MR analysis. This approach has been used in a recent study exploring the association between circulating metabolites of the tryptophan pathway and kidney function [33]. Several other studies have also shown that circulating metabolites of the tryptophan pathway were associated with kidney function and disease [24, 26, 29], which is in line with our findings demonstrating a strong association of C-glycosyltryptophan with eGFRcys and CKD. Notably, C-glycosyltryptophan is a post-translational modification product of tryptophan and such products could be endogenous toxins in chronic conditions such kidney failure [34]. However, the previous MR study [33] was not able to prove a causal association between higher levels of theses metabolites and lower eGFR. Conversely, they did show a causal association between lower eGFR and higher levels of these metabolites, suggesting that eGFR might be involved in the clearance of these metabolites instead of the metabolites having an effect on kidney function. Here, our MR analyses for the metabolites associated with kidney function also did not support a causal association between any of the investigated metabolites and kidney function, which might be explained by several reasons. It is well-known that the kidney can regulate the levels of circulating metabolite in order to maintain homeostasis. Thus, it is more likely that, based on our findings, changes in kidney function affect levels of circulating metabolites instead of the circulating metabolites affecting kidney function. Therefore, the metabolites that were identified as being significantly associated with eGFRcreat, eGFRcys, ACR, and CKD in the regression analyses, especially C-glycosyltryptophan and X-12026, might be considered more as potential biomarkers of kidney function rather than molecules involved in the pathogenesis of CKD. However, it might also be that eGFR does not completely represent true kidney function and that other results will be found when using measured kidney function as an outcome. Moreover, it should be acknowledged that the available WES/GWAS on metabolites derived from the Metabolon platform were limited and have their specific limitations. The WES study by Bomba *et al.* has a limited sample size and not all metabolites included in our study were available. The GWAS by Yin *et al.* however only included Finnish men, limiting its generalizability to other populations. It should also be noted that we only tested the causality for subset of 61 metabolites that were associated with all kidney function traits. Therefore, the results of our MR analyses should be interpreted with caution and should be explored further by other comprehensive studies before any final conclusions can be drawn.

Previous literature has described sex-differences in the prevalence and prognosis of CKD, which is often referred to as the ‘CKD paradox’. This paradox describes the phenomenon that women have a higher prevalence of CKD, while men with CKD have a worse prognosis due to a more rapid progression to kidney failure [35, 36]. Sex hormones such as serum testosterone might play an important role within these sex differences [36, 37]. In general, we observed a stronger association between the circulating metabolites and both eGFRcreat and eGFRcys in men compared to women, even though the sample size of men was smaller than women. In addition, we did identify sex differences in the sex-related metabolites 11-ketoetiocholanolone glucuronide and 11-beta-glucuronide. Both of these metabolites are etiocholanolones, which is a catabolic product of testosterone formed in the liver [38]. Later in the process of testosterone catabolism in the liver, glucuronidation of the etiocholanolones will occur in order to increase their hydrophilic character in such a way that it can be transported through the circulation to the kidney, where it can be excreted via urine [38]. This gives rise to the concern of reverse causation of our observed association. We observed that one unit increase in 11-ketoetiocholanolone glucuronide and 11-beta-glucuronide was associated with 2.89 and 1.99 ml/min per 1.73 m^2^ lower eGFRcreat levels in women, but with 4.22 and 4.17 ml/min per 1.73 m^2^ lower eGFRcreat levels in men, respectively. Baseline values of the circulating metabolites were, however, not different between men and women, which raises the question whether the identified associations could be fully explained by the filtration of these metabolites by the kidney. Thus, more causal investigation is needed to ascertain the direction of the association and to unravel whether the circulating metabolites 11-ketoetiocholanolone glucuronide and 11-beta-glucuronide could have a role as biomarker or even risk factor for kidney function decline.

The main strength of our study is that we analysed a high number of circulating metabolites from two different metabolomics platforms in a relatively large sample from the general population, enabling detailed investigation of metabolite-kidney associations with sufficient statistical power. Other strengths include the availability of different assessments of kidney function, the availability of repeated assessments of eGFRcreat, the longitudinal and prospective study design, the information on a wide variety of confounders, and the investigation of causality of the observed associations using a MR approach. However, some limitations should also be acknowledged. First, measurements of serum cystatin C and urine albumin and creatinine were not performed at the same time point as measurements of the circulating metabolites were performed. However, by adjusting all analyses with eGFRcys and ACR as the outcome for the time difference between the two different measurement time points, we could still provide valid effect estimates. Second, no repeated assessments of eGFRcys and ACR were available and therefore, only eGFRcreat could be used in the definition of CKD. Third, no information on acute kidney injury was available, however inspection of participants’ individual eGFR declines did not reveal any sharp declines in eGFR pointing towards episodes of acute kidney injury. Fourth, the included population comprises mainly individuals from European descent aged above 45 years, which might limit the generalizability of our findings to younger individuals and individuals from other ethnicities. Fifth, some limitations related to the MR analyses should be acknowledged, including the potential presence of unobserved horizontal pleiotropy, the possibility of weak instrument bias, the limited generalizability to ancestries other than European ancestry, and the relatively low number of genetic variants available for some of the metabolites. However, we attempted to address part of these limitations by current best practices using different statistical methods to identify horizontal pleiotropy and weak instruments, including the WME, MR-Egger, MR-PRESSO, and the F-statistic.

In summary, we report an association of several circulating metabolites with markers of kidney function (eGFRcreat, eGFRcys, and ACR) both in cross-sectional and longitudinal assessments and with incident CKD. Notably, all the identified metabolites were associated with an increased risk of CKD. We did not find evidence for a causal association between the identified metabolites and kidney function, however, our results showed that the identified metabolites have the potential to be used as biomarkers of kidney function and its decline. Future studies should confirm the potential of the identified metabolites as new biomarkers for kidney function and their potential role in the pathophysiology of CKD.

## Supplementary Material

sfad286_Supplemental_File

## Data Availability

Data can be obtained upon request. Requests should be directed towards the management team of the Rotterdam Study (datamanagement.ergo@erasmusmc.nl), which has a protocol for approving data requests. Because of restrictions based on privacy regulations and informed consent of the participants, data cannot be made freely available in a public repository.
