# Supplementary material for: Circulating metabolites associated with kidney function decline and incident CKD: a multi-platform population-based study
Source: Clin Kidney J. 2023 Nov 16;17(1):sfad286. doi: 10.1093/ckj/sfad286 (PMC10783258; doi:10.1093/ckj/sfad286)
Supplement: sfad286_Supplemental_File [file sfad286_supplemental_file.docx]

**Supplemental Materials**

**Supplemental Methods 1.** Study design and population

**Supplemental Methods 2.** Assessment of plasma metabolites and kidney function

S**upplemental Methods 3.** Assessment of covariates

**Supplemental Methods 4.** Genome-wide association studies for circulating metabolites and kidney function

**Supplemental Methods 5.** Overview of the included metabolite GWAS/WES

**Supplemental Methods 6.** Overview of the included kidney function GWAS

**Supplemental Methods 7.** Statistical analyses

**Supplemental Methods 8.** Mendelian randomization analyses

**Supplemental Table 1.** Association between serum metabolites from the Nightingale platform and eGFRcreat at baseline (n = 3,337)

**Supplemental Table 2.** Association between serum metabolites from the Metabolon platform and eGFRcreat at baseline (n = 1,540)

**Supplemental Table 3.** Association between serum metabolites from the Nightingale platform and eGFRcys at baseline (n = 3,091)

**Supplemental Table 4.** Association between serum metabolites from the Metabolon platform and eGFRcys at baseline (n = 1,452)

**Supplemental Table 5.** Association between serum metabolites from the Nightingale platform and urine albumin-to-creatinine ratio at baseline (n = 2,510)

**Supplemental Table 6.** Association between serum metabolites from the Metabolon platform and urine albumin-to-creatinine ratio at baseline (n = 1,290)

**Supplemental Table 7.** Association between serum metabolites from the Nightingale and Metabolon platform and eGFRcreat over time

**Supplemental Table 8.** Association between serum metabolites from the Nightingale and Metabolon platform and eGFRcreat at baseline and over time, ordered on P-values from the baseline analyses

**Supplemental Table 9.** Association between significantly associated circulating metabolites from the Nightingale and Metabolon platform and different CKD-related definitions

**Supplemental Figure 1.** Overview of the metabolites significantly associated with CKD

**Supplemental Figure 3.** Stratification analyses for the metabolites significantly associated with all three kidney function assessments

**Supplemental Figure 3.** Stratification analyses for the metabolites associated with sex hormones only

**Supplemental Figure 4.** Overview of selection of genome-wide significant variants for Mendelian Randomization analyses

**Supplemental Figure 5.** Mendelian Randomization analyses between metabolites and kidney function assessed with eGFRcreat

**Supplemental Figure 6.** Sensitivity Mendelian Randomization analyses between metabolites and kidney function assessed with eGFRcys

**Supplemental Figure 7.** Mendelian Randomization analyses between metabolites and kidney function assessed with ACR

**Supplemental Figure 8.** Mendelian Randomization analyses between metabolites and CKD

**Supplemental Methods 1.** Study design and population

This study was embedded within the Rotterdam Study, an ongoing prospective population-based cohort study designed to investigate the occurrence and determinants of age-related diseases in the general population. The study started in 1990 and included individuals aged 45 years and older from Ommoord, a district of Rotterdam, the Netherlands. Further details regarding the objectives and design of the study have been reported previously (1). Currently, the Rotterdam Study consists of four independent cohorts (RS-I, RS-II, RS-III, and RS-IV) and comprises approximately 18,000 participants in total. The average response rate is equal to 72%. For the current study, baseline was defined as the second visit of the first cohort (RS-I-4, 2002-2004) and the third visit of the third cohort (RS-III-2, 2012-2014). We included participants for whom data on circulating metabolites and eGFR based on serum creatinine (eGFRcreat) were available at baseline. The Rotterdam Study complies with the declaration of Helsinki and has been approved by the Medical Ethics Committee of the Erasmus MC (registration number MEC 02.1015) and by the Dutch Ministry of Health, Welfare and Sport (Population Screening Act WBO, license number 1071272-159521-PG). The Rotterdam Study Personal Registration Data collection is filed with the Erasmus MC Data Protection Officer under registration number EMC1712001. The Rotterdam Study has been entered into the Netherlands National Trial Register (NTR; www.trialregister.nl) and into the WHO International Clinical Trials Registry Platform (ICTRP; www.who.int/ictrp/network/primary/en/) under shared catalogue number NTR6831. All participants provided written informed consent.

**Supplemental Methods 2.** Assessment of plasma metabolites and kidney function

In the Rotterdam Study, metabolomics profiling was conducted by using nuclear magnetic resonance (NMR) spectroscopy and mass spectrometry (MS), the two most commonly employed techniques in the field of metabolomics. NMR-based metabolomics is a non-invasive and non-destructive technique with high reproducibility, making it a powerful tool for searching novel biomarkers. On the other hand, MS-based metabolomics offers quantitative analyses with high selectivity and sensitivity and the potential to identify novel metabolites. These techniques (NMR and MS) are integrated in the Nightingale and Metabolon platforms as explained below.

The Nightingale platform enables simultaneous quantification of 249 lipoprotein subclasses and metabolites including amino acids, ketone bodies, and gluconeogenesis-related metabolites. The metabolites present in this platform were quantified using fasted ethylenediaminetetraacetic acid (EDTA) plasma samples by high-throughput proton NMR metabolomics (Nightingale Health Ltd, Helsinki, Finland). The Nightingale Quantification Library 2020 was used for quantification of metabolites. Of the 249 metabolites, five were excluded because of >10% missingness in one or more of the Rotterdam Study cohorts. As pyruvate measurements are not reliable in EDTA plasma due to interference with the EDTA anticoagulant, they were excluded from the dataset. Therefore, our final dataset included 243 metabolites, of which 65 were relative lipoprotein lipid concentrations. All included metabolites were measured as concentrations (mmol/L or g/L), except for the relative lipoprotein lipid concentrations and other ratios (given in % and as ratios) and three metabolite derivatives measuring lipid particle volume in nanometer.

In addition, 1,387 circulating metabolites were available from the Metabolon platform. These metabolites are from different biochemical pathways, including lipids, amino acids, xenobiotics, nucleotides, cofactors and vitamins, peptides, carbohydrates, energy-related metabolites, and uncharacterized metabolites. The metabolites present in this platform were quantified using mass spectrometry (MS) technique (developed by Metabolon, Inc.). Pre-processing of the data was performed before data-analyses. First, 14 participants with missingness greater than 5 times the standard deviation (SD) of the mean missingness in all participants were excluded. Then all metabolites with missingness greater than 5 times SD of the mean missingness in all metabolites and with a coefficient of variance (CV) greater than 30% in internal control samples (NIST Standard Reference Material) were excluded. The remaining metabolites were log-transformed using a log2 transformation. Finally, we removed metabolites with missingness greater than 30 percent and then imputed the missing data in the remaining metabolites (n = 1,138) with the lowest limit of detection.

As both platforms include serum creatinine as a metabolite, we removed it as exposure for the current study as serum creatinine is also used in defining our outcome.

Serum creatinine measurements were performed using an enzymatic assay method and expressed in µmol/L (2). Measurements of serum creatinine from the Rotterdam Study were supplemented with measurements from the Star-MDC database, which is a database from a center for medical diagnostics for outpatients in the city of Rotterdam (3), providing multiple serum creatinine measurements over time. Serum cystatin C measurements were performed using particle-enhanced immunonephelometric assay and expressed in mg/L. As measurements of serum cystatin C were not performed at RS-I-4 and RS-III-2, cystatin C measurements from the third visit of RS-I (RS-I-3,1997-1999) and the first visit of RS-III (RS-III-1, 2006-2008) were used. Both eGFRcreat and eGFRcys were calculated according to the Chronic Kidney Disease Epidemiology Collaboration (CKD-EPI) formula (4, 5), without incorporation of race (6). Incident CKD was defined as the first follow-up eGFR assessment <60 ml/min per 1.73 m^2^. Four alternative definitions of incident CKD and/or CKD progression were considered as well, including: 1) two consecutive eGFRcreat measurements <60 ml/min per 1.73m^2^, 2) a single eGFRcreat < 45 ml/min/1.73m^2^, 3) eGFRcreat <60 ml/min per 1.73m^2^ determined with eGFRcreat slope , and 4) the composite outcome of 40% loss of eGFRcreat or kidney failure. Urine albumin and creatinine were measured using a turbidimetric and enzymatic method and measured by a Hitachi Modular P analyzer (Roche/Hitachi Diagnostics, Mannheim, Germany). Urine albumin-to-creatinine ratio (ACR) was calculated by dividing urine albumin by urine creatinine (mg/g). As measurements of urine ACR were not performed at RS-I-4, urine ACR measurements from the fifth visit of RS-I (RS-I-5, 2009-2011) were used.

**Supplemental Methods 3.** Assessment of covariates

Information on demographics, alcohol use, tobacco smoking, and lipid-lowering medication use was derived during home interviews and questionnaires at baseline. Participants reported on the number of alcoholic beverages consumed weekly and from this, total alcohol use was converted to grams per day. Tobacco smoking was categorized into never, former, and current smoking. Lipid-lowering medication use was defined according to the WHO's Anatomical Therapeutic Chemical (ATC) codes and included lipid modifying agents, ATC C10. Height and weight were measured at the research center, and body mass index (BMI) was computed by dividing weight in kilograms by squared height in meters. Serum cholesterol was measured by the Department of Clinical Chemistry of the Erasmus University Medical Center using standard laboratory techniques. Systolic and diastolic blood pressure were measured twice on the right brachial artery in sitting position using a random-zero sphygmomanometer. The average of two consecutive measurements was taken as the final measurement. Hypertension was defined as a systolic blood pressure >140 mm Hg and a diastolic blood pressure >90 mm Hg, or as the use of antihypertensive drugs prescribed for hypertension. Diabetes was defined as a fasting serum glucose concentration ≥7.0 mmol/L, a non-fasting glucose concentration ≥11.1 mmol/L (if fasting serum glucose was not available), or the use of glucose lowering medication. History of cardiovascular disease (CVD) was defined as a history of myocardial infarction, stroke, and coronary or other arterial revascularization (7, 8), and assessed during interviews and verified by clinical data from medical records (7).

**Supplemental Methods 4.** Genome-wide association studies for circulating metabolites and kidney function

Genome-wide significant variants were selected as instrumental variables for circulating metabolites and kidney function from the largest available genome-wide association studies (GWAS) including participants from European ancestry, if available.

Instrumental variants for circulating metabolites were selected from a whole-exome sequencing (WES) study and a genome-wide association study (GWAS). First, we selected the WES study by Bomba et al., which analyzed 995 metabolites from the Metabolon platform in 3,924 individuals of European ancestry (9). Second, we selected the GWAS by Yin et al., which analyzed 1,391 metabolites from the Metabolon platform in 6,136 Finnish men (10). Five different GWAS were included from which instrumental variants for the different kidney function assessments were selected. The first large-scale GWAS was a trans-ethnic meta-analysis by Stanzick et al. including 1,201,909 participants, of which 1,004,040 (83.5%) were of European ancestry (11). European-ancestry specific meta-analyses were conducted with log-transformed eGFRcreat as the outcome. The second and third GWAS were the European GWAS meta-analyses by by Li et al. (12) and Gorski et al.(13), including 32,861 and 24,063 participants, respectively. Both GWAS investigated continuous assessments of log-transformed eGFRcys. The fourth GWAS was a trans-ethnic GWAS meta-analysis including 564,257 participants by Teumer et al. (14), of which 547,361 (97.0%) were from European ancestry. European-ancestry specific meta-analyses were conducted with log-transformed ACR as the outcome. The fifth GWAS was a trans-ethnic GWAS by Wuttke et al. (15) including 765,348 participants, of which 567,460 (74.1%) were of European ancestry. In this study, we only selected the summary statistics from participants from European ancestry. This GWAS investigated CKD as outcome, defined as eGFRcreat <60 ml/min per 1.73 m^2^.

**Supplemental Methods 5.** Overview of the included metabolite GWAS/WES

| **GWAS** | **Phenotype(s)** | **N of studies** | **N of participants** | **Ethnicity** | **Summary statistic of age** | **N of women (%)** | **Phenotype definition** |
| --- | --- | --- | --- | --- | --- | --- | --- |
| Bomba et al. * | Plasma metabolites | NA | 3,924 | European ancestry | Median (SD): 44.5 (14.30) | 1,939 (50.50) | A total of 995 plasma metabolites measured with a non-targeted Metabolon HD4 metabolomics platform |
| Yin et al. | Plasma metabolites | NA | 6,136 | European ancestry (Finnish population) | Mean (SD): 58.1 (7.0) | 0 (0) | A total of 1,391 plasma metabolites measured using a non-targeted Metabolon DiscoveryHD4 mass spectrometry platform |

* Whole exome sequencing study.

**Supplemental Methods 6.** Overview of the included kidney function GWAS

| **GWAS** | **Phenotype(s)** | **N of studies** | **N of participants** | **Ethnicity** | **Summary statistic of age^$^** | **N of men and women** | **Summary statistic of kidney function assessment^$^** | **Phenotype definition** |
| --- | --- | --- | --- | --- | --- | --- | --- | --- |
| Stanzick et al. | eGFRcreat | 2 (UK Biobank and CKDgen, available from Wuttke et al.) | 1,201,909 | Trans-ethnic, mainly European Ancestry (n = 1,004,040) | UK Biobank: mean (SD): 56.76 (8.03) years** | UK Biobank: men: 54% women ** | UK Biobank mean (SD): 90.52 (13.27) ml/min/1.73m² ** | eGFRcreat was estimated  using the Chronic Kidney Disease Epidemiology Collaboration (CKD-EPI) formula. Log-transformed eGFRcreat, with eGFRcreat winsorized at 15 or 200 ml/min/1.73 m^2^. |
| Wuttke et al. | eGFRcreat  and CKD | 121 for eGFRcreat  60 for CKD | eGFRcreat: 765,348  CKD:  625,219 | Trans-ethnic, mainly European ancestry (n=567,460, based on total population) | Median: 54.0 years | Women: 50% | Median of the  study-specific mean eGFRcreat: 89 ml/min/1.73m²  (IQR: 81, 94)  N CKD cases: 64,164 | eGFRcreat was estimated  using the Chronic Kidney Disease Epidemiology Collaboration (CKD-EPI) formula. Log-transformed eGFRcreat, with eGFRcreat winsorized at 15 or 200 ml/min/1.73 m^2^.  CKD was defined as eGFRcreat below 60 ml/min/1.73m². |
| Li et al. | eGFRcys | 10  (N total = 27) | 32,861  (N total = 111,666) | European ancestry | Median of the  study-specific mean of Age: 53.0 years  (total: 54.7 years) | Women: 67.6% | Median of the  study-specific mean eGFRcys: 92.0 ml/min/1.73 m^2^ | eGFRcys was estimated as 76.73 multiplied by serum cystatin C). Log-transformed eGFRcys, with eGFRcys winsorized at 15 or 200 ml/min/1.73 m^2^. |
| Gorski et al. | eGFRcys | 11  (N total = 33) | 24,063  (N total = 110,517) | European ancestry | Median of the  study-specific mean of Age: 56.2 years  (total: 55.6 years) | Women: 67.6% | Median of the  study-specific mean eGFRcys: 86.3 ml/min/1.73 m^2^ | eGFRcys was estimated as 76.73 multiplied by serum cystatin C). Log-transformed eGFRcys, with eGFRcys winsorized at 15 or 200 ml/min/1.73 m^2^. |
| Teumer et al. | ACR | 54 | 564,257 | Trans-ethnic, mainly European ancestry (n= 547,361, based on total population) | Median of the  study-specific mean of Age: 58.6 years | Women: 52.4% | Median of the  study-specific mean ACR: 7.4 mg/g | Urinary albumin  values below the detection limit of the used assays were set to the lower limit of  detection. ACR was assessed in mg/g and calculated as urinary albumin (mg/l)/urinary creatinine (mg/dl) × 100. ACR was log transformed. |
| * In case of a trans-ethnic GWAS, data on sub-analyses performed in individuals from European ancestry only were selected, if available.  ** For the second study, see charateristics of Wuttke et al.  ^$^ Given for the specific EA population (where possible) in which data on the phenotype of interest was available.  Abbreviations: CKD = chronic kidney disease; eGFR = estimated glomerular filtration rate; eGFRcreat = eGFR based on serum creatinine; eGFRcys = eGFR based on serum cystatin C; IQR = interquartile range; N = number; SD = standard deviation. | | | | | | | | |

**Supplemental Methods 7.** Statistical analyses

All analyses were performed for participants with data available from the Nightingale and Metabolon platforms separately, after which results were shown together. Circulating metabolites were transformed using a natural log-transformation and 1 unit was added to the non-transformed values to account for zero values of the metabolites. Before analysis, the circulating metabolites were scaled to a mean of zero and a standard deviation (SD) of 1. When the denominator for calculating the ratios from the Nightingale platform was equal to zero, half of the minimum value in the raw value was used instead of zero in order to be able to calculate the ratio. To normalize the distribution of the urine ACR, it was naturally log-transformed after adding 1 mg/g to all ACR values before transformation to account for zero values. Missing values in covariates (missingness for all variables <4%, except for alcohol use (8%)) were handled by multiple imputation using the Multivariate Imputation by Chained Equations (MICE) package in R. All statistical analyses were performed using R statistical software (R-project, R Foundation for Statistical Computing (2020), 3.6.3).To correct for multiple testing in the analyses, the false discovery rate (FDR) by Benjamini-Hochberg (BH) was used (24). A FDR-corrected p-value <0.05 was set as the significance threshold in this study.

*Cross-sectional analyses*

Linear regression analyses were conducted to study the cross-sectional associations between circulating metabolites and eGFRcreat, eGFRcys, and urine ACR. Analyses with metabolites from the Nightingale platform were conducted separately per batch and results were meta-analyzed using the inverse variance weighted method. Analyses with metabolites from the Metabolon platform were conducted separately by Rotterdam Study cohort and results were meta-analyzed using the inverse variance weighted method as well. All primary analyses were adjusted for age, sex, and Rotterdam Study sub-cohort. In a second model, we additionally adjusted for the potential confounders BMI, smoking status, alcohol use, serum cholesterol, lipid-lowering drugs, and prevalent cardiovascular disease. In a third model, we additionally adjusted for hypertension and type 2 diabetes. As measurements of serum cystatin C were not performed at the same time as the measurements of metabolites, analyses with eGFRcys as the outcome were additionally adjusted for the time difference between the serum cystatin C measurement and the serum metabolite measurements. Results were reported as betas with their 95% confidence intervals (CIs) and heatmaps were used to graphically display the results. A Venn diagram was created to investigate the overlap in circulating metabolites significantly associated with all three kidney function outcomes. Predefined stratification by sex was performed in view of the “CKD paradox” (25, 26). To investigate the sex differences in more detail, stratified analyses were performed with 1) the circulating metabolites that were significantly associated with all three kidney function assessments at baseline, and 2) the sex-hormone related circulating metabolites, defined as androgenic steroids, pregnenolone steroids, and progestin steroids.

*Longitudinal analyses*

Longitudinal analyses were conducted with metabolites that were significantly associated with all three kidney function assessments at baseline. Linear mixed models were used to study the associations between circulating metabolites and repeated assessments of eGFRcreat over time only, as no repeated assessments of eGFRcys and ACR were available. In these analyses, time of kidney function assessment was used as the time variable. The random effects part of the model included a random intercept and a linear random slope. Cox proportional-hazards models were used to obtain hazard ratios (HRs) with their 95% CIs for the associations between circulating metabolites and CKD. The proportional-hazards assumption was checked using the Schoenfeld test and plot. Participants with prevalent reduced kidney function were excluded for these analyses. The longitudinal analyses were adjusted for the same covariates as described with the cross-sectional analyses. In an additional analysis, the third model that was used to investigate the association between circulating metabolites and CKD, was additionally adjusted for baseline eGFR. Heatmaps were used to show the results of the third model only. As sensitivity analyses, we investigated the association between circulating metabolites and the four alternative CKD definitions.

**Supplemental Methods 8.** Mendelian randomization analyses

Two-sample Mendelian randomization (MR) analyses were conducted to examine the potential causal association between circulatory metabolites and different assessments of kidney function, including estimated glomerular filtration rate (eGFR) based on serum creatinine (eGFRcreat), eGFR based on serum cystatin C (eGFRcys), albumin-to-creatinine ratio (ACR), and chronic kidney disease (CKD). The MR analyses were conducted given three assumptions: 1) the genetic variant is strongly associated with the exposure, 2) the genetic variant only affects the outcome through its effect on the exposure, and 3) the genetic variant is not associated with any confounders of the exposure-outcome relationship. For the metabolites that were significantly associated with eGFRcreat, eGFRcys, ACR, and CKD in the regression analyses, we selected genetic instruments that were genome-wide significantly (p<5e-08) associated with these metabolites from the exposure genome-wide association studies (GWAS). As a sensitivity analysis, we applied the less stringent threshold of p<1e-05 to include more variants for our MR analysis. To avoid weak instrument bias, we only selected the genetic instruments with a F-statistic >10 (16). Then, to remove correlated single nucleotide polymorphisms (SNPs), SNPs were clumped using a linkage disequilibrium threshold of r^2^ < 0.001. Genetic instruments for the kidney function assessments (eGFRcreat, eGFRcys, ACR, and CKD) were selected from one out of the five included GWAS. We performed harmonization of the exposure and outcome SNPs. The MRCIEU/TwoSampleMR package in R was used to perform the clumping and harmonization and to perform subsequent MR analyses (36). For the main analyses, we used the inverse-variance weighted (IVW) method (17, 18), which provides a weighted mean estimate of the metabolite-kidney function association. As MR estimates from the IVW method could be biased in case of horizontal pleiotropy, we conducted sensitivity analyses by using the weighted median estimator (WME) (19) and the MR-Egger methods (20). Similarity between the effect estimates obtained from the IVW, WME, and MR-Egger methods is indicative of robustness of the MR results (19). Furthermore, we used MR-PRESSO to identify and remove horizontal pleiotropic outliers in order to provide an outlier-corrected estimate (21). For the analyses with eGFRcreat as the outcome, the GWAS by Stanzick et al. (11) was included as main analysis. For the analyses with eGFRcys as the outcome, the GWAS by Li et al. (12) was included as main analysis, while the GWAS by Gorski et al. (13) was included as sensitivity analysis. For the analyses with ACR and CKD as the outcome, the GWAS by Teumer et al. (14) and the GWAS by Wuttke et al. (15) were included in the main analyses, respectively. Genetic variants for metabolites were selected from the WES study by Bomba et al. (9) and the GWAS by Yin et al. (10), and results were compared.

**Supplemental Table 1.** Association between serum metabolites from the Nightingale platform and eGFRcreat at baseline (n = 3,337)

|  | **Model 1** |  | **Model 2** |  | **Model 3** |  |
| --- | --- | --- | --- | --- | --- | --- |
| **Metabolite** | **Beta (95% CI)** | **P-value** | **Beta (95% CI)** | **P-value** | **Beta (95% CI)** | **P-value** |
| Citrate | -2.61 (-3.07;-2.16) | 7.01E-27 | -2.54 (-3.01;-2.08) | 6.13E-25 | -2.60 (-3.06;-2.14) | 4.39E-26 |
| Phenylalanine | -2.23 (-2.69;-1.78) | 6.28E-20 | -2.23 (-2.69;-1.76) | 7.51E-19 | -2.26 (-2.73;-1.79) | 1.94E-19 |
| Glycine | -1.94 (-2.41;-1.47) | 3.62E-14 | -2.11 (-2.59;-1.63) | 6.60E-16 | -2.10 (-2.59;-1.62) | 1.20E-15 |
| Triglycerides To Total Lipids Ratio In Large HDL | -1.60 (-2.05;-1.15) | 1.76E-10 | -1.65 (-2.12;-1.19) | 1.71E-10 | -1.75 (-2.22;-1.29) | 8.29E-12 |
| Cholesterol To Total Lipids Ratio In IDL | 1.25 (0.79;1.70) | 2.72E-07 | 1.66 (1.15;2.17) | 3.23E-09 | 1.82 (1.30;2.33) | 1.29E-10 |
| Cholesteryl Esters To Total Lipids Ratio In IDL | 1.31 (0.85;1.76) | 8.19E-08 | 1.65 (1.16;2.15) | 1.98E-09 | 1.76 (1.26;2.25) | 1.29E-10 |
| Cholesteryl Esters In Medium HDL | 1.72 (1.23;2.21) | 3.15E-10 | 1.74 (1.22;2.26) | 1.98E-09 | 1.82 (1.30;2.34) | 2.49E-10 |
| Triglycerides In Medium LDL | -1.44 (-1.89;-0.99) | 4.49E-09 | -1.69 (-2.22;-1.16) | 5.92E-09 | -1.84 (-2.37;-1.31) | 3.27E-10 |
| Glycoprotein Acetyls | -1.54 (-1.99;-1.09) | 4.52E-10 | -1.58 (-2.06;-1.11) | 1.98E-09 | -1.64 (-2.11;-1.16) | 3.39E-10 |
| Triglycerides To Total Lipids Ratio In Small HDL | -1.51 (-1.96;-1.05) | 1.55E-09 | -1.58 (-2.06;-1.09) | 3.23E-09 | -1.66 (-2.14;-1.18) | 3.39E-10 |
| Cholesterol In Medium HDL | 1.68 (1.18;2.17) | 7.65E-10 | 1.73 (1.20;2.26) | 3.23E-09 | 1.80 (1.27;2.33) | 4.33E-10 |
| Concentration Of Small VLDL Particles | -1.53 (-1.98;-1.09) | 5.09E-10 | -1.71 (-2.24;-1.18) | 5.18E-09 | -1.82 (-2.35;-1.28) | 5.24E-10 |
| Cholesteryl Esters In HDL | 1.54 (1.05;2.04) | 9.50E-09 | 1.73 (1.19;2.27) | 4.59E-09 | 1.82 (1.28;2.36) | 5.53E-10 |
| Phospholipids In Small VLDL | -1.42 (-1.86;-0.97) | 7.24E-09 | -2.03 (-2.66;-1.40) | 4.59E-09 | -2.13 (-2.76;-1.50) | 5.53E-10 |
| Triglycerides To Total Lipids Ratio In IDL | -1.09 (-1.54;-0.63) | 7.06E-06 | -1.64 (-2.17;-1.11) | 1.21E-08 | -1.79 (-2.32;-1.26) | 6.20E-10 |
| Cholesterol In Small VLDL | -1.40 (-1.84;-0.95) | 1.00E-08 | -2.09 (-2.74;-1.45) | 3.28E-09 | -2.15 (-2.79;-1.51) | 6.70E-10 |
| Cholesteryl Esters In Small VLDL | -1.44 (-1.89;-0.99) | 4.33E-09 | -1.97 (-2.58;-1.37) | 3.23E-09 | -2.02 (-2.62;-1.42) | 6.70E-10 |
| Total Lipids In Small VLDL | -1.49 (-1.94;-1.05) | 1.28E-09 | -1.74 (-2.28;-1.19) | 8.01E-09 | -1.85 (-2.40;-1.29) | 6.70E-10 |
| Triglycerides In LDL | -1.39 (-1.84;-0.94) | 1.37E-08 | -1.66 (-2.19;-1.12) | 1.31E-08 | -1.79 (-2.33;-1.26) | 7.48E-10 |
| Triglycerides In Very Small VLDL | -1.47 (-1.91;-1.02) | 2.48E-09 | -1.54 (-2.03;-1.05) | 8.01E-09 | -1.63 (-2.12;-1.14) | 7.48E-10 |
| HDL Cholesterol | 1.49 (1.00;1.99) | 2.76E-08 | 1.72 (1.17;2.27) | 8.41E-09 | 1.81 (1.26;2.35) | 1.00E-09 |
| Concentration Of VLDL Particles | -1.44 (-1.88;-0.99) | 4.49E-09 | -1.81 (-2.38;-1.23) | 1.00E-08 | -1.91 (-2.49;-1.33) | 1.00E-09 |
| Cholesteryl Esters To Total Lipids Ratio In Very Small VLDL | 1.13 (0.68;1.59) | 3.18E-06 | 1.52 (1.00;2.04) | 7.43E-08 | 1.73 (1.20;2.26) | 1.59E-09 |
| Cholesterol To Total Lipids Ratio In Very Small VLDL | 1.06 (0.61;1.52) | 1.17E-05 | 1.55 (1.01;2.08) | 9.36E-08 | 1.77 (1.23;2.31) | 1.68E-09 |
| Free Cholesterol In Small VLDL | -1.29 (-1.74;-0.84) | 8.41E-08 | -2.23 (-2.95;-1.52) | 1.02E-08 | -2.31 (-3.03;-1.60) | 2.16E-09 |
| Cholesterol To Total Lipids Ratio In Medium HDL | 1.37 (0.92;1.82) | 2.62E-08 | 1.43 (0.96;1.91) | 2.88E-08 | 1.54 (1.06;2.01) | 2.30E-09 |
| Triglycerides In Small LDL | -1.39 (-1.83;-0.94) | 1.22E-08 | -1.48 (-1.99;-0.98) | 6.66E-08 | -1.63 (-2.14;-1.12) | 3.45E-09 |
| Triglycerides To Total Lipids Ratio In Very Large HDL | -1.34 (-1.79;-0.88) | 4.75E-08 | -1.41 (-1.90;-0.93) | 7.40E-08 | -1.56 (-2.05;-1.07) | 3.45E-09 |
| Total Lipids In Large HDL | 1.45 (0.96;1.93) | 4.13E-08 | 1.55 (1.02;2.07) | 5.02E-08 | 1.66 (1.14;2.19) | 3.88E-09 |
| Free Cholesterol In VLDL | -1.40 (-1.84;-0.95) | 9.89E-09 | -1.69 (-2.26;-1.13) | 4.29E-08 | -1.81 (-2.38;-1.24) | 4.27E-09 |
| VLDL Cholesterol | -1.35 (-1.79;-0.90) | 2.78E-08 | -1.90 (-2.52;-1.27) | 2.66E-08 | -1.98 (-2.61;-1.35) | 4.29E-09 |
| Ratio Of Apolipoprotein B To Apolipoprotein A1 | -1.54 (-1.99;-1.09) | 7.59E-10 | -1.70 (-2.26;-1.15) | 1.96E-08 | -1.75 (-2.31;-1.20) | 4.52E-09 |
| Phospholipids In VLDL | -1.41 (-1.85;-0.96) | 8.02E-09 | -1.62 (-2.16;-1.07) | 5.40E-08 | -1.73 (-2.28;-1.18) | 4.74E-09 |
| Triglycerides In Large LDL | -1.32 (-1.78;-0.87) | 6.02E-08 | -1.60 (-2.14;-1.05) | 5.75E-08 | -1.71 (-2.25;-1.17) | 4.82E-09 |
| Cholesteryl Esters To Total Lipids Ratio In Medium HDL | 1.35 (0.90;1.80) | 2.76E-08 | 1.37 (0.90;1.84) | 7.35E-08 | 1.47 (1.00;1.94) | 6.25E-09 |
| Triglycerides To Total Lipids Ratio In Medium HDL | -1.36 (-1.81;-0.91) | 2.68E-08 | -1.39 (-1.86;-0.93) | 4.32E-08 | -1.46 (-1.93;-0.99) | 6.90E-09 |
| Free Cholesterol In Medium HDL | 1.47 (0.96;1.97) | 6.84E-08 | 1.67 (1.11;2.22) | 3.31E-08 | 1.72 (1.17;2.27) | 6.99E-09 |
| Triglycerides In IDL | -1.35 (-1.80;-0.90) | 3.76E-08 | -1.50 (-2.02;-0.99) | 7.33E-08 | -1.58 (-2.10;-1.07) | 1.06E-08 |
| Free Cholesterol In Medium VLDL | -1.27 (-1.72;-0.82) | 1.21E-07 | -1.97 (-2.64;-1.29) | 7.40E-08 | -2.05 (-2.73;-1.38) | 1.63E-08 |
| Ratio Of Triglycerides To Phosphoglycerides | -1.38 (-1.84;-0.92) | 3.54E-08 | -1.39 (-1.89;-0.90) | 1.59E-07 | -1.50 (-2.00;-1.01) | 1.80E-08 |
| Concentration Of Medium HDL Particles | 1.51 (1.01;2.01) | 3.30E-08 | 1.57 (1.03;2.11) | 7.35E-08 | 1.63 (1.09;2.17) | 1.93E-08 |
| Cholesteryl Esters In Large HDL | 1.31 (0.85;1.77) | 1.53E-07 | 1.35 (0.86;1.83) | 2.29E-07 | 1.46 (0.98;1.95) | 1.94E-08 |
| Cholesteryl Esters In VLDL | -1.26 (-1.70;-0.81) | 1.75E-07 | -2.01 (-2.70;-1.33) | 5.63E-08 | -2.05 (-2.74;-1.37) | 1.94E-08 |
| Phospholipids In Medium VLDL | -1.30 (-1.74;-0.85) | 6.92E-08 | -1.80 (-2.44;-1.17) | 1.28E-07 | -1.90 (-2.54;-1.27) | 2.16E-08 |
| Concentration Of Medium VLDL Particles | -1.32 (-1.77;-0.88) | 4.21E-08 | -1.72 (-2.33;-1.11) | 1.59E-07 | -1.82 (-2.43;-1.21) | 2.53E-08 |
| Free Cholesterol To Total Lipids Ratio In Large LDL | 1.13 (0.68;1.59) | 3.22E-06 | 1.31 (0.81;1.81) | 9.24E-07 | 1.51 (1.00;2.02) | 2.75E-08 |
| Ratio Of Monounsaturated Fatty Acids To Total Fatty Acids | -1.21 (-1.66;-0.76) | 5.75E-07 | -1.34 (-1.82;-0.86) | 2.07E-07 | -1.43 (-1.91;-0.95) | 2.75E-08 |
| Total Lipids In Medium HDL | 1.52 (1.02;2.03) | 2.76E-08 | 1.54 (1.00;2.07) | 1.05E-07 | 1.59 (1.05;2.12) | 2.93E-08 |
| Total Lipids In HDL | 1.42 (0.91;1.92) | 1.72E-07 | 1.58 (1.03;2.13) | 1.28E-07 | 1.64 (1.09;2.19) | 3.02E-08 |
| Cholesteryl Esters In Large VLDL | -1.41 (-1.86;-0.97) | 8.02E-09 | -1.46 (-1.98;-0.94) | 2.13E-07 | -1.54 (-2.06;-1.01) | 4.09E-08 |
| Phospholipids In HDL | 1.46 (0.96;1.97) | 7.87E-08 | 1.55 (1.00;2.09) | 1.59E-07 | 1.60 (1.06;2.15) | 4.35E-08 |
| Concentration Of Large HDL Particles | 1.28 (0.80;1.76) | 7.14E-07 | 1.40 (0.88;1.91) | 4.38E-07 | 1.51 (0.99;2.02) | 4.45E-08 |
| Triglycerides In Small HDL | -1.25 (-1.70;-0.81) | 1.93E-07 | -1.31 (-1.80;-0.83) | 3.91E-07 | -1.41 (-1.89;-0.92) | 5.58E-08 |
| Cholesterol In Large VLDL | -1.39 (-1.83;-0.94) | 1.46E-08 | -1.40 (-1.91;-0.89) | 3.84E-07 | -1.49 (-2.00;-0.97) | 6.19E-08 |
| Free Cholesterol In HDL | 1.24 (0.74;1.74) | 3.85E-06 | 1.60 (1.02;2.17) | 2.66E-07 | 1.67 (1.09;2.24) | 6.47E-08 |
| Triglycerides In Small VLDL | -1.33 (-1.78;-0.88) | 4.21E-08 | -1.30 (-1.79;-0.82) | 5.73E-07 | -1.40 (-1.89;-0.92) | 7.24E-08 |
| Apolipoprotein B | -1.06 (-1.51;-0.61) | 1.01E-05 | -2.49 (-3.39;-1.60) | 2.24E-07 | -2.56 (-3.45;-1.67) | 7.79E-08 |
| Total Lipids In VLDL | -1.32 (-1.77;-0.87) | 4.75E-08 | -1.39 (-1.91;-0.87) | 6.88E-07 | -1.51 (-2.03;-0.98) | 7.95E-08 |
| Isoleucine | -1.34 (-1.83;-0.84) | 4.49E-07 | -1.27 (-1.80;-0.75) | 5.05E-06 | -1.53 (-2.06;-0.99) | 9.14E-08 |
| Concentration Of LDL Particles | -1.07 (-1.51;-0.62) | 9.15E-06 | -2.43 (-3.31;-1.55) | 3.12E-07 | -2.51 (-3.39;-1.63) | 9.66E-08 |
| Phospholipids To Total Lipids Ratio In Very Small VLDL | -1.24 (-1.69;-0.78) | 3.27E-07 | -1.27 (-1.74;-0.79) | 5.80E-07 | -1.34 (-1.81;-0.87) | 1.11E-07 |
| Total Lipids In Medium VLDL | -1.30 (-1.75;-0.85) | 6.73E-08 | -1.51 (-2.08;-0.94) | 7.82E-07 | -1.62 (-2.19;-1.04) | 1.18E-07 |
| Free Cholesterol In Large VLDL | -1.34 (-1.79;-0.89) | 3.88E-08 | -1.33 (-1.83;-0.83) | 8.18E-07 | -1.43 (-1.93;-0.92) | 1.23E-07 |
| Concentration Of Medium LDL Particles | -1.14 (-1.59;-0.69) | 1.96E-06 | -1.93 (-2.64;-1.21) | 5.82E-07 | -2.03 (-2.75;-1.31) | 1.23E-07 |
| Phospholipids In Medium HDL | 1.48 (0.98;1.98) | 5.44E-08 | 1.46 (0.93;1.99) | 3.68E-07 | 1.50 (0.97;2.03) | 1.25E-07 |
| Phospholipids In Very Small VLDL | -1.27 (-1.73;-0.82) | 1.81E-07 | -1.66 (-2.26;-1.06) | 2.47E-07 | -1.68 (-2.28;-1.09) | 1.25E-07 |
| Concentration Of Small LDL Particles | -1.12 (-1.57;-0.67) | 2.81E-06 | -2.08 (-2.85;-1.30) | 6.46E-07 | -2.19 (-2.97;-1.41) | 1.25E-07 |
| Triglycerides To Total Lipids Ratio In Very Small VLDL | -0.85 (-1.30;-0.39) | 5.45E-04 | -1.37 (-1.92;-0.83) | 2.46E-06 | -1.52 (-2.07;-0.98) | 1.79E-07 |
| Free Cholesterol To Total Lipids Ratio In Medium HDL | 0.89 (0.41;1.38) | 6.19E-04 | 1.63 (1.03;2.24) | 5.73E-07 | 1.69 (1.08;2.30) | 1.86E-07 |
| Triglycerides To Total Lipids Ratio In Large LDL | -0.82 (-1.27;-0.36) | 7.42E-04 | -1.33 (-1.85;-0.81) | 1.95E-06 | -1.43 (-1.95;-0.91) | 2.41E-07 |
| Cholesterol In Large HDL | 1.19 (0.72;1.66) | 1.93E-06 | 1.24 (0.75;1.73) | 2.42E-06 | 1.36 (0.86;1.85) | 2.46E-07 |
| Concentration Of Large VLDL Particles | -1.29 (-1.74;-0.83) | 1.13E-07 | -1.26 (-1.76;-0.76) | 2.36E-06 | -1.36 (-1.86;-0.86) | 3.48E-07 |
| Phospholipids In Large HDL | 1.25 (0.77;1.72) | 8.53E-07 | 1.24 (0.75;1.73) | 2.34E-06 | 1.33 (0.84;1.82) | 3.56E-07 |
| Triglycerides To Total Lipids Ratio In Small LDL | -0.95 (-1.40;-0.50) | 7.97E-05 | -1.22 (-1.71;-0.73) | 3.61E-06 | -1.34 (-1.84;-0.85) | 3.79E-07 |
| Apolipoprotein A1 | 1.29 (0.78;1.79) | 1.91E-06 | 1.46 (0.90;2.02) | 1.08E-06 | 1.50 (0.95;2.06) | 4.06E-07 |
| Free Cholesterol To Total Lipids Ratio In Medium LDL | 1.06 (0.61;1.51) | 1.01E-05 | 1.19 (0.71;1.67) | 3.51E-06 | 1.30 (0.82;1.78) | 4.08E-07 |
| Total Triglycerides | -1.22 (-1.67;-0.77) | 3.89E-07 | -1.22 (-1.72;-0.73) | 3.60E-06 | -1.34 (-1.84;-0.84) | 4.08E-07 |
| Concentration Of Very Large VLDL Particles | -1.26 (-1.71;-0.81) | 1.89E-07 | -1.24 (-1.74;-0.75) | 2.60E-06 | -1.34 (-1.84;-0.84) | 4.08E-07 |
| Free Cholesterol In Very Small VLDL | -1.18 (-1.63;-0.72) | 1.36E-06 | -1.78 (-2.44;-1.12) | 5.40E-07 | -1.78 (-2.44;-1.12) | 4.08E-07 |
| Free Cholesterol In Large HDL | 1.14 (0.67;1.61) | 5.49E-06 | 1.21 (0.71;1.70) | 4.69E-06 | 1.32 (0.83;1.82) | 5.14E-07 |
| Triglycerides In Very Large HDL | -1.21 (-1.67;-0.76) | 5.98E-07 | -1.25 (-1.75;-0.75) | 2.84E-06 | -1.33 (-1.83;-0.83) | 5.34E-07 |
| Total Lipids In Very Small VLDL | -1.17 (-1.63;-0.72) | 1.57E-06 | -1.71 (-2.36;-1.07) | 8.00E-07 | -1.72 (-2.37;-1.08) | 5.35E-07 |
| Cholesteryl Esters In Small LDL | -1.04 (-1.49;-0.59) | 1.48E-05 | -2.04 (-2.86;-1.23) | 2.83E-06 | -2.16 (-2.97;-1.34) | 6.33E-07 |
| Cholesterol In Very Large VLDL | -1.28 (-1.73;-0.83) | 1.21E-07 | -1.27 (-1.79;-0.76) | 3.51E-06 | -1.36 (-1.88;-0.85) | 6.33E-07 |
| Average Diameter For HDL Particles | 1.27 (0.79;1.75) | 7.61E-07 | 1.26 (0.75;1.77) | 3.83E-06 | 1.35 (0.84;1.86) | 7.09E-07 |
| Free Cholesterol In Very Large VLDL | -1.25 (-1.70;-0.80) | 1.96E-07 | -1.22 (-1.72;-0.72) | 4.35E-06 | -1.31 (-1.81;-0.81) | 8.56E-07 |
| Total Lipids In Large VLDL | -1.24 (-1.69;-0.79) | 2.69E-07 | -1.20 (-1.70;-0.70) | 6.26E-06 | -1.30 (-1.81;-0.80) | 9.85E-07 |
| Monounsaturated Fatty Acids | -1.13 (-1.58;-0.68) | 2.71E-06 | -1.26 (-1.79;-0.73) | 9.73E-06 | -1.40 (-1.94;-0.86) | 9.85E-07 |
| Concentration Of Very Small VLDL Particles | -1.14 (-1.60;-0.68) | 3.18E-06 | -1.74 (-2.40;-1.07) | 1.27E-06 | -1.73 (-2.40;-1.07) | 9.85E-07 |
| Cholesteryl Esters In Very Large VLDL | -1.27 (-1.72;-0.82) | 1.41E-07 | -1.29 (-1.82;-0.76) | 4.57E-06 | -1.37 (-1.90;-0.84) | 9.91E-07 |
| Phospholipids In Large VLDL | -1.25 (-1.70;-0.80) | 2.05E-07 | -1.20 (-1.68;-0.71) | 4.20E-06 | -1.27 (-1.75;-0.78) | 1.03E-06 |
| Triglycerides To Total Lipids Ratio In Medium LDL | -0.76 (-1.21;-0.31) | 1.78E-03 | -1.22 (-1.73;-0.70) | 9.32E-06 | -1.33 (-1.84;-0.81) | 1.27E-06 |
| Triglycerides In Medium VLDL | -1.22 (-1.67;-0.77) | 3.85E-07 | -1.17 (-1.67;-0.67) | 1.13E-05 | -1.28 (-1.78;-0.77) | 1.78E-06 |
| Cholesterol In Medium VLDL | -1.04 (-1.49;-0.59) | 1.42E-05 | -2.12 (-2.95;-1.29) | 1.99E-06 | -2.10 (-2.93;-1.27) | 1.86E-06 |
| Ratio Of Polyunsaturated Fatty Acids To Monounsaturated Fatty Acids | 0.94 (0.49;1.39) | 9.57E-05 | 1.12 (0.64;1.60) | 1.29E-05 | 1.22 (0.74;1.70) | 1.96E-06 |
| Total Lipids In Very Large VLDL | -1.19 (-1.64;-0.74) | 8.53E-07 | -1.15 (-1.65;-0.66) | 1.24E-05 | -1.25 (-1.75;-0.75) | 2.21E-06 |
| Total Lipids In Small LDL | -0.99 (-1.44;-0.54) | 4.02E-05 | -1.99 (-2.83;-1.14) | 1.12E-05 | -2.12 (-2.97;-1.28) | 2.30E-06 |
| Triglycerides In VLDL | -1.16 (-1.61;-0.71) | 1.35E-06 | -1.13 (-1.62;-0.64) | 1.61E-05 | -1.24 (-1.74;-0.75) | 2.34E-06 |
| Free Cholesterol To Total Lipids Ratio In Small LDL | 0.99 (0.55;1.44) | 3.26E-05 | 1.07 (0.61;1.53) | 1.39E-05 | 1.15 (0.69;1.61) | 2.95E-06 |
| Cholesteryl Esters In Medium LDL | -1.04 (-1.49;-0.58) | 1.74E-05 | -1.70 (-2.44;-0.97) | 1.27E-05 | -1.81 (-2.55;-1.08) | 3.00E-06 |
| Concentration Of HDL Particles | 1.03 (0.53;1.53) | 1.29E-04 | 1.39 (0.81;1.97) | 7.28E-06 | 1.41 (0.84;1.99) | 4.11E-06 |
| Phospholipids To Total Lipids Ratio In Medium HDL | -0.95 (-1.41;-0.49) | 1.10E-04 | -1.11 (-1.60;-0.61) | 2.60E-05 | -1.19 (-1.69;-0.70) | 5.94E-06 |
| Triglycerides In HDL | -1.01 (-1.46;-0.56) | 2.96E-05 | -1.06 (-1.54;-0.59) | 2.59E-05 | -1.13 (-1.60;-0.65) | 7.89E-06 |
| Phospholipids In Very Large VLDL | -1.17 (-1.62;-0.72) | 1.28E-06 | -1.09 (-1.58;-0.60) | 2.59E-05 | -1.15 (-1.64;-0.67) | 7.91E-06 |
| Triglycerides In Very Large VLDL | -1.11 (-1.56;-0.66) | 4.03E-06 | -1.06 (-1.55;-0.58) | 3.81E-05 | -1.15 (-1.63;-0.66) | 9.06E-06 |
| Free Cholesterol To Total Lipids Ratio In Small VLDL | 0.70 (0.24;1.15) | 4.87E-03 | 1.15 (0.60;1.70) | 8.04E-05 | 1.29 (0.74;1.84) | 1.07E-05 |
| Remnant Cholesterol (Non-HDL. Non-LDL -Cholesterol) | -0.90 (-1.36;-0.45) | 2.21E-04 | -2.19 (-3.15;-1.24) | 1.55E-05 | -2.21 (-3.16;-1.26) | 1.19E-05 |
| Total Lipids In Medium LDL | -0.95 (-1.40;-0.50) | 8.49E-05 | -1.76 (-2.56;-0.95) | 4.68E-05 | -1.87 (-2.68;-1.06) | 1.26E-05 |
| Triglycerides In Large VLDL | -1.09 (-1.54;-0.64) | 5.77E-06 | -1.02 (-1.52;-0.53) | 9.85E-05 | -1.12 (-1.62;-0.63) | 1.95E-05 |
| Cholesterol In Small LDL | -0.93 (-1.38;-0.48) | 1.17E-04 | -1.86 (-2.73;-0.99) | 5.71E-05 | -1.96 (-2.83;-1.10) | 1.95E-05 |
| Concentration Of Large LDL Particles | -0.94 (-1.39;-0.49) | 9.57E-05 | -1.96 (-2.85;-1.08) | 3.25E-05 | -1.99 (-2.88;-1.11) | 2.12E-05 |
| Linoleic Acid | -1.08 (-1.53;-0.62) | 1.01E-05 | -1.46 (-2.14;-0.78) | 6.28E-05 | -1.52 (-2.21;-0.84) | 2.71E-05 |
| Phospholipids To Total Lipids Ratio In Small VLDL | 0.66 (0.21;1.12) | 7.23E-03 | 1.08 (0.55;1.62) | 1.53E-04 | 1.20 (0.66;1.74) | 2.99E-05 |
| Phospholipids To Total Lipids Ratio In Medium LDL | 0.91 (0.46;1.36) | 1.60E-04 | 0.99 (0.52;1.45) | 6.65E-05 | 1.03 (0.56;1.49) | 3.25E-05 |
| Total Concentration Of Lipoprotein Particles | 0.77 (0.26;1.27) | 4.86E-03 | 1.37 (0.74;2.01) | 5.07E-05 | 1.39 (0.76;2.03) | 3.43E-05 |
| Cholesterol In Chylomicrons And Extremely Large VLDL | -1.02 (-1.47;-0.57) | 2.12E-05 | -0.98 (-1.46;-0.50) | 1.23E-04 | -1.06 (-1.54;-0.58) | 3.43E-05 |
| Cholesterol In Medium LDL | -0.93 (-1.38;-0.48) | 1.29E-04 | -1.66 (-2.46;-0.85) | 1.11E-04 | -1.76 (-2.56;-0.95) | 3.75E-05 |
| Cholesteryl Esters In Chylomicrons And Extremely Large VLDL | -1.03 (-1.48;-0.58) | 1.85E-05 | -0.97 (-1.45;-0.49) | 1.50E-04 | -1.05 (-1.53;-0.57) | 4.27E-05 |
| Free Cholesterol In Chylomicrons And Extremely Large VLDL | -0.95 (-1.40;-0.50) | 7.97E-05 | -0.93 (-1.40;-0.45) | 2.67E-04 | -0.99 (-1.47;-0.52) | 9.34E-05 |
| Total Fatty Acids | -0.93 (-1.39;-0.47) | 1.61E-04 | -1.13 (-1.75;-0.51) | 6.75E-04 | -1.30 (-1.92;-0.67) | 1.01E-04 |
| Concentration Of Chylomicrons And Extremely Large VLDL Particles | -0.95 (-1.40;-0.50) | 7.87E-05 | -0.93 (-1.41;-0.45) | 3.15E-04 | -1.00 (-1.48;-0.51) | 1.03E-04 |
| Leucine | -0.95 (-1.45;-0.44) | 4.65E-04 | -0.81 (-1.33;-0.29) | 4.16E-03 | -1.10 (-1.63;-0.56) | 1.25E-04 |
| Cholesterol In Very Large HDL | 0.72 (0.25;1.19) | 4.52E-03 | 0.95 (0.43;1.48) | 7.34E-04 | 1.06 (0.54;1.59) | 1.49E-04 |
| Phospholipids In Small LDL | -0.87 (-1.32;-0.42) | 2.96E-04 | -1.57 (-2.42;-0.73) | 5.24E-04 | -1.69 (-2.54;-0.85) | 1.63E-04 |
| Cholesteryl Esters To Total Lipids Ratio In Medium LDL | -0.89 (-1.35;-0.44) | 2.21E-04 | -0.89 (-1.36;-0.42) | 4.11E-04 | -0.94 (-1.41;-0.47) | 1.90E-04 |
| Cholesterol To Total Lipids Ratio In Large LDL | 0.46 (0.01;0.91) | 5.69E-02 | 1.00 (0.46;1.54) | 5.78E-04 | 1.07 (0.53;1.61) | 1.97E-04 |
| Phospholipids To Total Lipids Ratio In Small HDL | 1.19 (0.72;1.65) | 1.58E-06 | 0.93 (0.44;1.42) | 4.39E-04 | 0.97 (0.48;1.46) | 2.02E-04 |
| Average Diameter For VLDL Particles | -0.90 (-1.36;-0.44) | 2.69E-04 | -0.83 (-1.31;-0.34) | 1.48E-03 | -0.94 (-1.43;-0.45) | 3.16E-04 |
| Total Cholesterol Minus HDL-C | -0.78 (-1.23;-0.32) | 1.59E-03 | -1.99 (-3.06;-0.93) | 4.93E-04 | -2.03 (-3.09;-0.96) | 3.58E-04 |
| Total Concentration Of Branched-Chain Amino Acids (Leucine + Isoleucine + Valine) | -0.91 (-1.40;-0.42) | 5.45E-04 | -0.75 (-1.27;-0.23) | 7.56E-03 | -1.02 (-1.55;-0.48) | 3.61E-04 |
| Cholesteryl Esters To Total Lipids Ratio In Large HDL | 0.78 (0.34;1.23) | 1.10E-03 | 0.78 (0.33;1.24) | 1.31E-03 | 0.86 (0.41;1.31) | 3.72E-04 |
| Phospholipids In Chylomicrons And Extremely Large VLDL | -0.87 (-1.33;-0.42) | 3.13E-04 | -0.85 (-1.32;-0.37) | 9.39E-04 | -0.90 (-1.38;-0.43) | 3.98E-04 |
| Cholesteryl Esters To Total Lipids Ratio In Medium VLDL | 0.65 (0.19;1.11) | 8.31E-03 | 0.80 (0.30;1.31) | 3.11E-03 | 0.95 (0.44;1.46) | 4.98E-04 |
| Phospholipids In Medium LDL | -0.83 (-1.28;-0.38) | 6.30E-04 | -1.44 (-2.28;-0.61) | 1.39E-03 | -1.55 (-2.39;-0.71) | 5.25E-04 |
| Total Lipids In Very Large HDL | 0.68 (0.21;1.16) | 7.56E-03 | 0.84 (0.33;1.36) | 2.51E-03 | 0.95 (0.43;1.47) | 6.51E-04 |
| Total Lipids In Chylomicrons And Extremely Large VLDL | -0.84 (-1.29;-0.39) | 4.95E-04 | -0.79 (-1.27;-0.32) | 1.98E-03 | -0.87 (-1.35;-0.39) | 7.00E-04 |
| Alanine | -0.84 (-1.29;-0.39) | 4.77E-04 | -0.68 (-1.13;-0.22) | 6.23E-03 | -0.84 (-1.30;-0.37) | 7.10E-04 |
| Histidine | -0.82 (-1.27;-0.37) | 6.88E-04 | -0.78 (-1.23;-0.33) | 1.27E-03 | -0.81 (-1.26;-0.36) | 7.60E-04 |
| Degree Of Unsaturation | 0.55 (0.09;1.00) | 2.61E-02 | 0.80 (0.33;1.27) | 1.66E-03 | 0.84 (0.37;1.31) | 7.82E-04 |
| Cholesteryl Esters To Total Lipids Ratio In Very Large VLDL | 0.65 (0.20;1.10) | 8.11E-03 | 0.78 (0.29;1.27) | 3.39E-03 | 0.88 (0.38;1.37) | 9.50E-04 |
| Cholesterol To Total Lipids Ratio In Medium VLDL | 0.56 (0.10;1.02) | 2.41E-02 | 0.78 (0.26;1.30) | 5.71E-03 | 0.93 (0.40;1.45) | 9.94E-04 |
| Albumin | -0.85 (-1.31;-0.39) | 5.83E-04 | -0.80 (-1.27;-0.32) | 1.79E-03 | -0.82 (-1.29;-0.35) | 1.11E-03 |
| Cholesteryl Esters In IDL | 0.03 (-0.44;0.50) | 9.13E-01 | 1.43 (0.48;2.38) | 5.36E-03 | 1.65 (0.70;2.61) | 1.20E-03 |
| Triglycerides In Medium HDL | -0.71 (-1.17;-0.26) | 3.57E-03 | -0.77 (-1.24;-0.30) | 2.35E-03 | -0.81 (-1.28;-0.34) | 1.20E-03 |
| Total Lipids In Lipoprotein Particles | -0.75 (-1.21;-0.28) | 2.91E-03 | -1.37 (-2.25;-0.49) | 3.88E-03 | -1.52 (-2.40;-0.64) | 1.20E-03 |
| Ratio Of Polyunsaturated Fatty Acids To Total Fatty Acids | 0.55 (0.10;1.00) | 2.42E-02 | 0.73 (0.25;1.21) | 4.45E-03 | 0.83 (0.35;1.31) | 1.20E-03 |
| Sphingomyelins | 0.16 (-0.33;0.64) | 5.78E-01 | 1.41 (0.52;2.29) | 3.39E-03 | 1.47 (0.58;2.36) | 1.95E-03 |
| Free Cholesterol In Very Large HDL | 0.57 (0.11;1.03) | 2.33E-02 | 0.74 (0.24;1.25) | 6.55E-03 | 0.83 (0.32;1.34) | 2.22E-03 |
| Free Cholesterol To Total Lipids Ratio In Very Large HDL | -0.70 (-1.19;-0.22) | 7.43E-03 | -0.75 (-1.27;-0.24) | 6.53E-03 | -0.84 (-1.36;-0.33) | 2.23E-03 |
| Cholesteryl Esters In Very Large HDL | 0.57 (0.10;1.03) | 2.41E-02 | 0.73 (0.22;1.23) | 7.43E-03 | 0.83 (0.32;1.33) | 2.28E-03 |
| Cholesteryl Esters In LDL | -0.74 (-1.19;-0.28) | 2.68E-03 | -1.48 (-2.44;-0.51) | 4.39E-03 | -1.55 (-2.51;-0.59) | 2.55E-03 |
| Phospholipids To Total Lipids Ratio In IDL | -0.75 (-1.20;-0.30) | 2.00E-03 | -0.74 (-1.20;-0.29) | 2.46E-03 | -0.73 (-1.18;-0.27) | 2.74E-03 |
| Phospholipids In Very Large HDL | 0.56 (0.10;1.02) | 2.50E-02 | 0.65 (0.17;1.13) | 1.25E-02 | 0.78 (0.29;1.26) | 2.74E-03 |
| Phospholipids To Total Lipids Ratio In Small LDL | 0.75 (0.30;1.21) | 2.00E-03 | 0.74 (0.26;1.22) | 4.13E-03 | 0.76 (0.29;1.24) | 2.77E-03 |
| Omega-6 Fatty Acids | -0.86 (-1.33;-0.40) | 5.57E-04 | -1.08 (-1.81;-0.35) | 6.27E-03 | -1.17 (-1.90;-0.44) | 2.79E-03 |
| Cholesteryl Esters To Total Lipids Ratio In Small HDL | 0.59 (0.14;1.04) | 1.57E-02 | 0.68 (0.22;1.14) | 5.87E-03 | 0.73 (0.27;1.19) | 2.79E-03 |
| Phospholipids To Total Lipids Ratio In Large VLDL | -0.76 (-1.21;-0.32) | 1.54E-03 | -0.70 (-1.15;-0.25) | 3.98E-03 | -0.71 (-1.16;-0.26) | 3.21E-03 |
| Free Cholesterol To Total Lipids Ratio In Large HDL | 0.60 (0.15;1.05) | 1.36E-02 | 0.63 (0.17;1.09) | 1.03E-02 | 0.72 (0.26;1.18) | 3.35E-03 |
| Saturated Fatty Acids | -0.69 (-1.15;-0.23) | 5.75E-03 | -0.79 (-1.39;-0.19) | 1.36E-02 | -0.94 (-1.55;-0.34) | 3.48E-03 |
| Cholesterol In IDL | -0.02 (-0.49;0.46) | 9.54E-01 | 1.25 (0.29;2.22) | 1.49E-02 | 1.49 (0.53;2.46) | 3.80E-03 |
| Cholesterol To Total Lipids Ratio In Very Large VLDL | 0.55 (0.10;1.01) | 2.41E-02 | 0.68 (0.18;1.17) | 1.05E-02 | 0.76 (0.27;1.26) | 3.80E-03 |
| Ratio Of Omega-6 Fatty Acids To Total Fatty Acids | 0.48 (0.03;0.93) | 4.85E-02 | 0.63 (0.14;1.12) | 1.58E-02 | 0.76 (0.26;1.25) | 4.16E-03 |
| Total Lipids In LDL | -0.70 (-1.16;-0.25) | 4.25E-03 | -1.47 (-2.48;-0.46) | 6.69E-03 | -1.54 (-2.54;-0.53) | 4.26E-03 |
| Triglycerides To Total Lipids Ratio In Medium VLDL | -0.48 (-0.93;-0.02) | 5.52E-02 | -0.70 (-1.22;-0.18) | 1.20E-02 | -0.80 (-1.32;-0.27) | 4.26E-03 |
| Cholesterol To Total Lipids Ratio In Large HDL | 0.61 (0.16;1.06) | 1.14E-02 | 0.61 (0.16;1.07) | 1.18E-02 | 0.69 (0.24;1.14) | 4.31E-03 |
| Cholesteryl Esters To Total Lipids Ratio In Small LDL | -0.76 (-1.21;-0.30) | 1.91E-03 | -0.74 (-1.22;-0.26) | 4.38E-03 | -0.72 (-1.20;-0.25) | 4.47E-03 |
| Total Free Cholesterol | -0.65 (-1.11;-0.18) | 1.02E-02 | -1.85 (-3.11;-0.58) | 6.69E-03 | -1.87 (-3.13;-0.61) | 5.43E-03 |
| Polyunsaturated Fatty Acids | -0.82 (-1.29;-0.35) | 1.05E-03 | -0.94 (-1.65;-0.22) | 1.52E-02 | -1.06 (-1.78;-0.34) | 5.72E-03 |
| Triglycerides To Total Lipids Ratio In Small VLDL | -0.38 (-0.83;0.07) | 1.23E-01 | -0.68 (-1.20;-0.15) | 1.64E-02 | -0.76 (-1.29;-0.23) | 7.32E-03 |
| Cholesterol To Total Lipids Ratio In Small HDL | 0.42 (-0.02;0.87) | 8.05E-02 | 0.62 (0.15;1.08) | 1.43E-02 | 0.66 (0.19;1.13) | 8.05E-03 |
| Concentration Of Very Large HDL Particles | 0.46 (-0.01;0.94) | 7.19E-02 | 0.68 (0.14;1.22) | 1.80E-02 | 0.76 (0.22;1.30) | 8.18E-03 |
| Cholesteryl Esters In Small HDL | 0.51 (0.03;0.98) | 4.85E-02 | 0.73 (0.21;1.26) | 9.66E-03 | 0.73 (0.20;1.25) | 9.41E-03 |
| Tyrosine | 0.59 (0.14;1.04) | 1.51E-02 | 0.65 (0.19;1.10) | 8.98E-03 | 0.64 (0.18;1.10) | 9.76E-03 |
| Phospholipids To Total Lipids Ratio In Very Large HDL | 0.46 (0.01;0.91) | 5.99E-02 | 0.50 (0.04;0.96) | 4.32E-02 | 0.63 (0.17;1.09) | 1.06E-02 |
| Phospholipids To Total Lipids Ratio In Large LDL | 0.67 (0.22;1.12) | 5.42E-03 | 0.56 (0.08;1.05) | 3.17E-02 | 0.66 (0.17;1.14) | 1.13E-02 |
| Triglycerides In Large HDL | -0.55 (-1.03;-0.08) | 3.00E-02 | -0.63 (-1.11;-0.14) | 1.56E-02 | -0.64 (-1.12;-0.16) | 1.28E-02 |
| Ratio Of Docosahexaenoic Acid To Total Fatty Acids | 0.58 (0.13;1.03) | 1.66E-02 | 0.62 (0.16;1.08) | 1.27E-02 | 0.60 (0.14;1.06) | 1.43E-02 |
| Valine | -0.66 (-1.13;-0.19) | 9.89E-03 | -0.45 (-0.96;0.06) | 1.03E-01 | -0.67 (-1.19;-0.15) | 1.54E-02 |
| Cholesterol In Very Small VLDL | -0.72 (-1.18;-0.26) | 4.14E-03 | -1.08 (-1.84;-0.32) | 8.54E-03 | -0.97 (-1.73;-0.21) | 1.74E-02 |
| Cholesterol To Total Lipids Ratio In Small VLDL | 0.32 (-0.13;0.77) | 1.99E-01 | 0.54 (0.02;1.06) | 5.73E-02 | 0.66 (0.13;1.18) | 1.95E-02 |
| Cholesterol In Small HDL | 0.40 (-0.07;0.88) | 1.20E-01 | 0.69 (0.14;1.25) | 1.95E-02 | 0.68 (0.12;1.23) | 2.22E-02 |
| Free Cholesterol To Total Lipids Ratio In Large VLDL | -0.61 (-1.05;-0.16) | 1.19E-02 | -0.56 (-1.02;-0.11) | 2.07E-02 | -0.55 (-1.00;-0.09) | 2.41E-02 |
| Phospholipids In LDL | -0.64 (-1.10;-0.19) | 9.09E-03 | -1.16 (-2.16;-0.15) | 3.27E-02 | -1.19 (-2.20;-0.19) | 2.66E-02 |
| Glutamine | -0.62 (-1.07;-0.16) | 1.17E-02 | -0.65 (-1.11;-0.19) | 8.19E-03 | -0.55 (-1.01;-0.09) | 2.67E-02 |
| LDL Cholesterol | -0.64 (-1.09;-0.18) | 9.69E-03 | -1.14 (-2.15;-0.14) | 3.50E-02 | -1.18 (-2.18;-0.17) | 2.87E-02 |
| Phospholipids To Total Lipids Ratio In Large HDL | 0.55 (0.11;1.00) | 2.19E-02 | 0.49 (0.04;0.94) | 4.21E-02 | 0.51 (0.07;0.96) | 3.10E-02 |
| Triglycerides To Total Lipids Ratio In Large VLDL | 0.62 (0.17;1.06) | 1.02E-02 | 0.54 (0.08;1.00) | 2.85E-02 | 0.52 (0.06;0.98) | 3.59E-02 |
| Free Cholesterol To Total Lipids Ratio In Medium VLDL | 0.26 (-0.20;0.72) | 2.98E-01 | 0.49 (-0.05;1.04) | 9.47E-02 | 0.61 (0.06;1.15) | 3.83E-02 |
| Cholesterol To Total Lipids Ratio In Small LDL | 0.20 (-0.26;0.66) | 4.44E-01 | 0.46 (-0.08;0.99) | 1.16E-01 | 0.56 (0.03;1.10) | 4.99E-02 |
| Acetone | -0.38 (-0.83;0.08) | 1.27E-01 | -0.44 (-0.90;0.01) | 7.36E-02 | -0.47 (-0.93;-0.01) | 5.69E-02 |
| Average Diameter For LDL Particles | 0.29 (-0.17;0.74) | 2.49E-01 | 0.43 (-0.05;0.90) | 1.03E-01 | 0.49 (0.01;0.97) | 5.88E-02 |
| Triglycerides To Total Lipids Ratio In Very Large VLDL | -0.37 (-0.82;0.08) | 1.27E-01 | -0.43 (-0.89;0.04) | 9.16E-02 | -0.47 (-0.94;-0.01) | 6.01E-02 |
| Total Esterified Cholesterol | -0.20 (-0.67;0.28) | 4.70E-01 | 1.13 (-0.11;2.37) | 9.36E-02 | 1.24 (0.00;2.48) | 6.35E-02 |
| Total Cholines | 0.08 (-0.42;0.58) | 8.10E-01 | 0.81 (0.02;1.61) | 5.83E-02 | 0.78 (-0.01;1.57) | 6.91E-02 |
| Phospholipids In Small HDL | 0.45 (-0.03;0.94) | 8.49E-02 | 0.55 (0.01;1.08) | 5.94E-02 | 0.52 (-0.01;1.06) | 7.16E-02 |
| Free Cholesterol To Total Lipids Ratio In Small HDL | -0.59 (-1.05;-0.13) | 1.67E-02 | -0.49 (-1.05;0.07) | 1.06E-01 | -0.51 (-1.07;0.04) | 8.75E-02 |
| Cholesteryl Esters To Total Lipids Ratio In Very Large HDL | 0.32 (-0.12;0.77) | 1.86E-01 | 0.36 (-0.09;0.82) | 1.38E-01 | 0.41 (-0.04;0.86) | 9.17E-02 |
| Free Cholesterol In Large LDL | -0.19 (-0.65;0.27) | 4.72E-01 | 0.73 (-0.25;1.70) | 1.74E-01 | 0.88 (-0.09;1.86) | 9.56E-02 |
| Cholesteryl Esters In Medium VLDL | -0.66 (-1.11;-0.20) | 7.56E-03 | -0.80 (-1.55;-0.04) | 5.21E-02 | -0.68 (-1.44;0.08) | 9.83E-02 |
| Free Cholesterol In IDL | -0.15 (-0.62;0.32) | 5.73E-01 | 0.58 (-0.32;1.49) | 2.46E-01 | 0.80 (-0.11;1.72) | 1.03E-01 |
| Phospholipids To Total Lipids Ratio In Very Large VLDL | -0.50 (-0.94;-0.05) | 4.02E-02 | -0.38 (-0.84;0.07) | 1.19E-01 | -0.38 (-0.83;0.07) | 1.20E-01 |
| Glucose | 0.61 (0.16;1.06) | 1.20E-02 | 0.80 (0.32;1.28) | 1.98E-03 | 0.47 (-0.10;1.04) | 1.29E-01 |
| Lactate | -0.39 (-0.84;0.05) | 1.05E-01 | -0.26 (-0.72;0.19) | 2.95E-01 | -0.37 (-0.83;0.08) | 1.31E-01 |
| Phospholipids To Total Lipids Ratio In Medium VLDL | 0.14 (-0.32;0.60) | 5.85E-01 | 0.34 (-0.20;0.88) | 2.56E-01 | 0.44 (-0.11;0.98) | 1.42E-01 |
| Phosphoglycerides | 0.07 (-0.43;0.57) | 8.19E-01 | 0.63 (-0.11;1.36) | 1.16E-01 | 0.56 (-0.17;1.30) | 1.58E-01 |
| Phosphatidylcholines | 0.07 (-0.43;0.56) | 8.32E-01 | 0.58 (-0.14;1.31) | 1.38E-01 | 0.54 (-0.19;1.26) | 1.75E-01 |
| Free Cholesterol To Total Lipids Ratio In IDL | 0.22 (-0.24;0.67) | 3.97E-01 | 0.24 (-0.23;0.72) | 3.51E-01 | 0.34 (-0.13;0.82) | 1.90E-01 |
| Total Lipids In Small HDL | 0.26 (-0.22;0.75) | 3.24E-01 | 0.41 (-0.14;0.96) | 1.69E-01 | 0.38 (-0.17;0.92) | 2.10E-01 |
| Total Lipids In IDL | -0.22 (-0.70;0.25) | 3.97E-01 | 0.51 (-0.51;1.52) | 3.68E-01 | 0.70 (-0.32;1.72) | 2.11E-01 |
| Concentration Of Small HDL Particles | 0.16 (-0.31;0.64) | 5.49E-01 | 0.41 (-0.15;0.98) | 1.77E-01 | 0.38 (-0.18;0.94) | 2.15E-01 |
| Concentration Of IDL Particles | -0.55 (-1.01;-0.09) | 2.74E-02 | -0.68 (-1.55;0.19) | 1.51E-01 | -0.58 (-1.45;0.29) | 2.21E-01 |
| Cholesteryl Esters In Large LDL | -0.53 (-0.98;-0.07) | 3.32E-02 | -0.65 (-1.67;0.37) | 2.48E-01 | -0.67 (-1.68;0.35) | 2.31E-01 |
| Cholesteryl Esters To Total Lipids Ratio In Large VLDL | 0.12 (-0.33;0.57) | 6.54E-01 | 0.23 (-0.26;0.72) | 3.81E-01 | 0.32 (-0.17;0.82) | 2.31E-01 |
| Total Lipids In Large LDL | -0.52 (-0.98;-0.06) | 3.73E-02 | -0.69 (-1.77;0.38) | 2.44E-01 | -0.68 (-1.75;0.39) | 2.43E-01 |
| Ratio Of Saturated Fatty Acids To Total Fatty Acids | 0.51 (0.06;0.96) | 3.51E-02 | 0.33 (-0.13;0.80) | 1.89E-01 | 0.30 (-0.17;0.76) | 2.43E-01 |
| Free Cholesterol In Small LDL | -0.55 (-1.00;-0.10) | 2.41E-02 | -0.48 (-1.30;0.34) | 2.88E-01 | -0.51 (-1.33;0.31) | 2.52E-01 |
| Triglycerides In Chylomicrons And Extremely Large VLDL | -0.33 (-0.78;0.11) | 1.73E-01 | -0.25 (-0.71;0.21) | 3.32E-01 | -0.27 (-0.73;0.19) | 2.79E-01 |
| Cholesterol To Total Lipids Ratio In Very Large HDL | -0.29 (-0.76;0.18) | 2.68E-01 | -0.22 (-0.70;0.25) | 3.88E-01 | -0.26 (-0.74;0.21) | 3.17E-01 |
| Free Cholesterol To Total Lipids Ratio In Very Small VLDL | -0.48 (-0.93;-0.03) | 4.85E-02 | -0.33 (-0.85;0.19) | 2.54E-01 | -0.26 (-0.79;0.26) | 3.65E-01 |
| Free Cholesterol In Medium LDL | -0.51 (-0.96;-0.05) | 3.92E-02 | -0.42 (-1.30;0.45) | 3.81E-01 | -0.43 (-1.30;0.45) | 3.77E-01 |
| Clinical LDL Cholesterol | -0.50 (-0.96;-0.04) | 4.19E-02 | -0.56 (-1.59;0.48) | 3.32E-01 | -0.50 (-1.53;0.53) | 3.82E-01 |
| Free Cholesterol To Total Lipids Ratio In Very Large VLDL | 0.08 (-0.37;0.53) | 7.61E-01 | 0.17 (-0.31;0.65) | 5.15E-01 | 0.23 (-0.25;0.71) | 3.87E-01 |
| Cholesteryl Esters To Total Lipids Ratio In Small VLDL | 0.03 (-0.42;0.49) | 9.05E-01 | 0.13 (-0.37;0.62) | 6.44E-01 | 0.22 (-0.28;0.72) | 4.16E-01 |
| Acetoacetate | 0.40 (-0.05;0.85) | 1.00E-01 | 0.26 (-0.19;0.71) | 3.03E-01 | 0.20 (-0.25;0.65) | 4.24E-01 |
| Free Cholesterol In Small HDL | 0.02 (-0.47;0.51) | 9.50E-01 | 0.32 (-0.32;0.96) | 3.70E-01 | 0.26 (-0.38;0.90) | 4.67E-01 |
| Cholesteryl Esters In Very Small VLDL | -0.47 (-0.93;0.00) | 6.41E-02 | -0.46 (-1.22;0.31) | 2.79E-01 | -0.30 (-1.06;0.46) | 4.79E-01 |
| Cholesterol To Total Lipids Ratio In Medium LDL | -0.05 (-0.50;0.40) | 8.57E-01 | 0.12 (-0.37;0.62) | 6.54E-01 | 0.19 (-0.30;0.69) | 4.82E-01 |
| Ratio Of Omega-3 Fatty Acids To Total Fatty Acids | 0.09 (-0.36;0.54) | 7.45E-01 | 0.23 (-0.25;0.71) | 3.81E-01 | 0.18 (-0.29;0.66) | 4.82E-01 |
| Cholesteryl Esters To Total Lipids Ratio In Large LDL | -0.39 (-0.84;0.07) | 1.16E-01 | -0.13 (-0.63;0.37) | 6.33E-01 | -0.19 (-0.69;0.31) | 4.82E-01 |
| 3-Hydroxybutyrate | 0.06 (-0.41;0.53) | 8.32E-01 | -0.10 (-0.57;0.38) | 7.04E-01 | -0.16 (-0.63;0.31) | 5.43E-01 |
| Docosahexaenoic Acid | 0.03 (-0.43;0.49) | 9.22E-01 | 0.25 (-0.27;0.76) | 3.81E-01 | 0.17 (-0.34;0.69) | 5.43E-01 |
| Free Cholesterol In LDL | -0.32 (-0.78;0.14) | 2.04E-01 | 0.24 (-0.73;1.20) | 6.55E-01 | 0.32 (-0.64;1.28) | 5.43E-01 |
| Total Phospholipids In Lipoprotein Particles | -0.19 (-0.68;0.30) | 4.86E-01 | 0.34 (-0.57;1.25) | 5.02E-01 | 0.30 (-0.61;1.20) | 5.47E-01 |
| Total Cholesterol | -0.33 (-0.80;0.14) | 2.03E-01 | 0.34 (-0.97;1.65) | 6.41E-01 | 0.41 (-0.89;1.72) | 5.55E-01 |
| Omega-3 Fatty Acids | -0.27 (-0.73;0.19) | 2.88E-01 | -0.08 (-0.59;0.44) | 7.85E-01 | -0.16 (-0.68;0.36) | 5.75E-01 |
| Phospholipids In Large LDL | -0.45 (-0.91;0.01) | 6.84E-02 | -0.36 (-1.42;0.69) | 5.32E-01 | -0.30 (-1.36;0.75) | 5.92E-01 |
| Cholesterol In Large LDL | -0.45 (-0.90;0.01) | 7.21E-02 | -0.30 (-1.34;0.74) | 6.07E-01 | -0.27 (-1.31;0.77) | 6.26E-01 |
| Cholesterol To Total Lipids Ratio In Large VLDL | -0.07 (-0.52;0.38) | 8.11E-01 | 0.01 (-0.46;0.49) | 9.56E-01 | 0.08 (-0.40;0.56) | 7.70E-01 |
| Phospholipids In IDL | -0.35 (-0.82;0.12) | 1.70E-01 | -0.04 (-1.02;0.93) | 9.37E-01 | 0.14 (-0.84;1.12) | 7.90E-01 |
| Ratio Of Linoleic Acid To Total Fatty Acids | -0.31 (-0.76;0.14) | 2.05E-01 | -0.12 (-0.63;0.39) | 6.66E-01 | -0.03 (-0.54;0.48) | 9.27E-01 |
| Acetate | -0.01 (-0.46;0.43) | 9.54E-01 | 0.00 (-0.45;0.45) | 9.98E-01 | -0.02 (-0.47;0.43) | 9.42E-01 |
| Ratio Of Omega-6 Fatty Acids To Omega-3 Fatty Acids | 0.02 (-0.44;0.47) | 9.54E-01 | -0.09 (-0.57;0.40) | 7.42E-01 | -0.02 (-0.51;0.47) | 9.42E-01 |

Model 1 is adjusted for age, sex, and Rotterdam Study Cohort

Model 2 is additionally adjusted for body mass index, smoking status, alcohol use, serum cholesterol, lipid-lowering drugs, and prevalent cardiovascular disease

Model 3 is additionally adjusted for hypertension and diabetes

All serum metabolites are log-transformed.

* P-values are FDR-corrected.

Abbreviations: CI = confidence interval; eGFRcreat = estimated glomerular filtration rate based on serum creatinine; HDL = high-density lipoprotein; IDL = Intermediate-density lipoprotein; LDL = low-density lipoprotein; VLDL = very low-density lipoprotein.

**Supplemental Table 2.** Association between serum metabolites from the Metabolon platform and eGFRcreat at baseline (n = 1,540)

|  | **Model 1** |  | **Model 2** |  | **Model 3** |  |
| --- | --- | --- | --- | --- | --- | --- |
| **Metabolite** | **Beta (95% CI)** | **P-value** | **Beta (95% CI)** | **P-value** | **Beta (95% CI)** | **P-value** |
| 2,3-Dihydroxy-5-Methylthio-4-Pentenoate (DMTPA)* | -6.27 (-6.88;-5.66) | 1.30E-86 | -6.48 (-7.11;-5.85) | 3.66E-88 | -6.48 (-7.11;-5.86) | 3.30E-88 |
| N,N,N-Trimethyl-Alanylproline Betaine (TMAP) | -6.19 (-6.80;-5.57) | 2.19E-84 | -6.21 (-6.83;-5.60) | 2.08E-84 | -6.18 (-6.79;-5.56) | 3.87E-83 |
| X-12026 | -5.57 (-6.18;-4.95) | 8.22E-68 | -5.86 (-6.50;-5.21) | 1.57E-68 | -5.89 (-6.54;-5.24) | 3.39E-68 |
| 3-(3-Amino-3-Carboxypropyl)Uridine* | -5.64 (-6.26;-5.02) | 4.24E-69 | -5.62 (-6.25;-5.00) | 2.46E-67 | -5.64 (-6.27;-5.02) | 7.73E-68 |
| X-25371 | -5.34 (-5.94;-4.73) | 2.95E-65 | -5.51 (-6.13;-4.88) | 9.19E-65 | -5.48 (-6.10;-4.85) | 6.25E-64 |
| Hydroxyasparagine** | -5.35 (-5.96;-4.74) | 2.10E-64 | -5.36 (-5.98;-4.74) | 4.71E-63 | -5.34 (-5.96;-4.73) | 1.44E-62 |
| C-Glycosyltryptophan | -5.43 (-6.05;-4.81) | 1.19E-63 | -5.40 (-6.02;-4.77) | 3.49E-62 | -5.39 (-6.01;-4.76) | 6.30E-62 |
| Pseudouridine | -5.22 (-5.83;-4.61) | 2.70E-60 | -5.21 (-5.82;-4.59) | 1.06E-59 | -5.23 (-5.85;-4.61) | 8.57E-60 |
| X-25790 | -5.35 (-6.00;-4.71) | 3.18E-57 | -5.47 (-6.12;-4.81) | 3.75E-58 | -5.46 (-6.11;-4.80) | 1.03E-57 |
| N,N-Dimethyl-Pro-Pro | -5.04 (-5.66;-4.42) | 1.35E-54 | -5.05 (-5.67;-4.42) | 1.05E-53 | -5.03 (-5.66;-4.40) | 2.00E-53 |
| X-15503 | -4.88 (-5.51;-4.26) | 2.13E-50 | -5.29 (-5.96;-4.62) | 2.70E-52 | -5.29 (-5.96;-4.62) | 3.74E-52 |
| N-Acetylcarnosine | -5.66 (-6.39;-4.94) | 3.69E-51 | -5.71 (-6.45;-4.98) | 1.51E-50 | -5.70 (-6.44;-4.96) | 8.99E-50 |
| N-Acetylserine | -4.80 (-5.42;-4.18) | 5.53E-50 | -4.77 (-5.39;-4.14) | 1.43E-48 | -4.76 (-5.38;-4.13) | 2.42E-48 |
| X-12906 | -4.60 (-5.21;-3.98) | 1.79E-46 | -4.72 (-5.34;-4.10) | 3.19E-48 | -4.73 (-5.35;-4.10) | 2.58E-48 |
| N-Acetylalanine | -4.75 (-5.37;-4.14) | 8.38E-50 | -4.72 (-5.35;-4.10) | 2.55E-48 | -4.70 (-5.33;-4.08) | 7.89E-48 |
| S-Adenosylhomocysteine (SAH) | -4.91 (-5.55;-4.26) | 3.26E-48 | -4.88 (-5.54;-4.22) | 5.24E-46 | -4.95 (-5.61;-4.29) | 3.62E-47 |
| O-Sulfo-L-Tyrosine | -4.60 (-5.21;-3.99) | 3.30E-47 | -4.57 (-5.19;-3.95) | 7.48E-46 | -4.58 (-5.20;-3.96) | 4.60E-46 |
| N-Acetylneuraminate | -4.47 (-5.09;-3.85) | 3.94E-44 | -4.61 (-5.23;-3.98) | 4.91E-46 | -4.59 (-5.21;-3.97) | 9.45E-46 |
| Erythronate* | -4.58 (-5.19;-3.96) | 3.33E-46 | -4.54 (-5.16;-3.91) | 3.57E-44 | -4.62 (-5.25;-3.99) | 1.97E-45 |
| N-Formylmethionine | -4.69 (-5.32;-4.06) | 1.68E-46 | -4.66 (-5.29;-4.03) | 1.58E-45 | -4.66 (-5.29;-4.02) | 2.05E-45 |
| X-17676 | -4.63 (-5.25;-4.00) | 6.76E-46 | -4.62 (-5.25;-3.98) | 1.27E-44 | -4.61 (-5.24;-3.98) | 1.64E-44 |
| X-13553 | -4.67 (-5.30;-4.03) | 1.17E-44 | -4.61 (-5.26;-3.96) | 8.02E-43 | -4.63 (-5.28;-3.99) | 3.05E-43 |
| Kynurenate | -4.37 (-5.00;-3.73) | 8.60E-40 | -4.59 (-5.24;-3.93) | 1.81E-41 | -4.58 (-5.24;-3.93) | 2.07E-41 |
| Homocitrulline | -4.37 (-4.99;-3.75) | 2.23E-41 | -4.40 (-5.04;-3.77) | 9.44E-41 | -4.40 (-5.03;-3.77) | 1.18E-40 |
| 5,6-Dihydrouridine | -4.40 (-5.03;-3.77) | 2.51E-41 | -4.41 (-5.04;-3.78) | 9.44E-41 | -4.39 (-5.02;-3.75) | 2.87E-40 |
| Orotidine | -4.37 (-5.01;-3.73) | 4.06E-39 | -4.39 (-5.04;-3.75) | 9.29E-39 | -4.43 (-5.08;-3.79) | 2.11E-39 |
| N-Acetylvaline | -4.27 (-4.90;-3.64) | 6.25E-39 | -4.35 (-4.99;-3.70) | 1.82E-38 | -4.36 (-5.00;-3.71) | 1.66E-38 |
| Erythritol | -4.22 (-4.85;-3.59) | 5.58E-38 | -4.22 (-4.86;-3.59) | 1.45E-37 | -4.26 (-4.89;-3.62) | 3.89E-38 |
| X-12117 | -4.21 (-4.85;-3.58) | 3.31E-37 | -4.37 (-5.03;-3.72) | 1.73E-37 | -4.42 (-5.07;-3.76) | 3.89E-38 |
| Arabitol/Xylitol | -4.20 (-4.83;-3.57) | 1.49E-37 | -4.22 (-4.85;-3.59) | 1.35E-37 | -4.23 (-4.86;-3.60) | 1.13E-37 |
| 2S,3R-Dihydroxybutyrate | -4.74 (-5.45;-4.03) | 2.21E-37 | -4.81 (-5.53;-4.09) | 1.05E-37 | -4.82 (-5.54;-4.10) | 1.33E-37 |
| Hydroxy-N6,N6,N6-Trimethyllysine* | -4.25 (-4.88;-3.62) | 2.75E-38 | -4.26 (-4.90;-3.62) | 1.12E-37 | -4.26 (-4.89;-3.62) | 1.57E-37 |
| N-Acetylthreonine | -4.25 (-4.88;-3.62) | 2.34E-38 | -4.23 (-4.86;-3.60) | 9.97E-38 | -4.22 (-4.85;-3.58) | 1.58E-37 |
| N1-Methylinosine | -4.23 (-4.88;-3.57) | 2.11E-35 | -4.38 (-5.05;-3.72) | 5.12E-37 | -4.38 (-5.04;-3.72) | 7.77E-37 |
| N-Acetylmethionine | -4.21 (-4.84;-3.57) | 4.51E-37 | -4.21 (-4.85;-3.57) | 9.94E-37 | -4.19 (-4.83;-3.55) | 3.54E-36 |
| N2,N2-Dimethylguanosine | -4.14 (-4.78;-3.51) | 3.48E-36 | -4.19 (-4.84;-3.55) | 4.14E-36 | -4.17 (-4.81;-3.53) | 1.38E-35 |
| Vanillactate | -4.20 (-4.83;-3.56) | 6.35E-37 | -4.16 (-4.79;-3.52) | 6.79E-36 | -4.13 (-4.77;-3.49) | 3.06E-35 |
| X-25422 | -4.05 (-4.69;-3.41) | 3.21E-34 | -4.11 (-4.75;-3.47) | 8.71E-35 | -4.10 (-4.74;-3.45) | 1.67E-34 |
| X-23659 | -4.11 (-4.75;-3.47) | 3.21E-35 | -4.07 (-4.72;-3.43) | 1.14E-33 | -4.12 (-4.77;-3.47) | 2.42E-34 |
| Guaiacol Sulfate | -4.09 (-4.73;-3.45) | 7.52E-35 | -4.07 (-4.72;-3.42) | 2.37E-33 | -4.11 (-4.76;-3.46) | 4.75E-34 |
| X-24728 | -6.72 (-7.82;-5.62) | 5.63E-32 | -6.97 (-8.08;-5.86) | 1.41E-33 | -7.09 (-8.21;-5.98) | 5.12E-34 |
| Methionine Sulfone | -4.04 (-4.68;-3.40) | 1.70E-33 | -4.08 (-4.72;-3.43) | 1.40E-33 | -4.08 (-4.73;-3.44) | 1.09E-33 |
| Picolinoylglycine | -4.13 (-4.78;-3.48) | 4.11E-34 | -4.28 (-4.96;-3.61) | 9.31E-34 | -4.28 (-4.96;-3.60) | 1.37E-33 |
| Vanillylmandelate (VMA) | -4.15 (-4.80;-3.50) | 1.79E-34 | -4.16 (-4.82;-3.50) | 1.64E-33 | -4.14 (-4.81;-3.48) | 8.39E-33 |
| 1-Ribosyl-Imidazoleacetate* | -4.01 (-4.65;-3.37) | 3.17E-33 | -3.98 (-4.62;-3.34) | 1.99E-32 | -3.97 (-4.61;-3.33) | 2.85E-32 |
| N-Carbamoylalanine | -3.85 (-4.48;-3.22) | 9.78E-32 | -3.90 (-4.54;-3.27) | 7.28E-32 | -3.90 (-4.54;-3.27) | 7.32E-32 |
| X-12822 | -3.97 (-4.61;-3.32) | 2.43E-32 | -3.98 (-4.62;-3.33) | 8.03E-32 | -3.97 (-4.62;-3.32) | 1.05E-31 |
| Kynurenine | -3.92 (-4.55;-3.28) | 4.04E-32 | -4.01 (-4.66;-3.35) | 4.25E-32 | -3.98 (-4.63;-3.33) | 1.23E-31 |
| N6-Carbamoylthreonyladenosine | -4.04 (-4.68;-3.40) | 9.41E-34 | -3.97 (-4.62;-3.33) | 4.22E-32 | -3.94 (-4.58;-3.29) | 1.93E-31 |
| X-12100 | -3.92 (-4.56;-3.29) | 3.57E-32 | -3.93 (-4.57;-3.29) | 1.29E-31 | -3.92 (-4.56;-3.27) | 1.93E-31 |
| N2,N5-Diacetylornithine | -3.72 (-4.35;-3.08) | 3.81E-29 | -3.90 (-4.54;-3.25) | 8.38E-31 | -3.91 (-4.55;-3.26) | 5.80E-31 |
| N-Carbamoylvaline | -4.02 (-4.68;-3.37) | 2.28E-32 | -3.98 (-4.63;-3.32) | 2.59E-31 | -3.95 (-4.61;-3.30) | 7.10E-31 |
| Arabonate/Xylonate | -3.78 (-4.40;-3.15) | 8.35E-31 | -3.78 (-4.41;-3.14) | 2.04E-30 | -3.80 (-4.43;-3.16) | 9.97E-31 |
| X-24334 | -3.92 (-4.59;-3.25) | 2.25E-29 | -3.94 (-4.62;-3.26) | 9.98E-29 | -4.13 (-4.82;-3.44) | 1.26E-30 |
| 1-Methylhistidine | -4.45 (-5.19;-3.71) | 8.35E-31 | -4.45 (-5.19;-3.71) | 2.18E-30 | -4.44 (-5.19;-3.70) | 2.10E-30 |
| Methylsuccinoylcarnitine | -3.79 (-4.42;-3.16) | 4.61E-31 | -3.83 (-4.46;-3.19) | 5.97E-31 | -3.79 (-4.43;-3.16) | 3.44E-30 |
| Myo-Inositol | -3.77 (-4.39;-3.14) | 8.13E-31 | -3.77 (-4.40;-3.14) | 2.25E-30 | -3.74 (-4.37;-3.11) | 6.68E-30 |
| X-13507 | -3.69 (-4.32;-3.06) | 3.63E-29 | -3.72 (-4.35;-3.08) | 5.46E-29 | -3.75 (-4.38;-3.11) | 1.79E-29 |
| Glutarylcarnitine (C5-DC) | -3.85 (-4.49;-3.22) | 2.77E-31 | -3.80 (-4.44;-3.16) | 3.41E-30 | -3.76 (-4.40;-3.12) | 3.98E-29 |
| Glutamine_Degradant* | -4.35 (-5.10;-3.60) | 1.33E-28 | -4.46 (-5.22;-3.70) | 2.13E-29 | -4.43 (-5.19;-3.66) | 1.22E-28 |
| X-18886 | -3.75 (-4.43;-3.06) | 6.75E-26 | -3.97 (-4.66;-3.28) | 5.64E-28 | -4.03 (-4.73;-3.34) | 1.43E-28 |
| X-13844 | -3.71 (-4.35;-3.07) | 6.39E-29 | -3.75 (-4.40;-3.11) | 8.01E-29 | -3.73 (-4.37;-3.08) | 2.09E-28 |
| 3-Methylglutarylcarnitine (2) | -3.71 (-4.34;-3.07) | 6.48E-29 | -3.87 (-4.53;-3.20) | 1.20E-28 | -3.84 (-4.51;-3.17) | 3.58E-28 |
| X-12680 | -3.66 (-4.28;-3.03) | 7.80E-29 | -3.66 (-4.30;-3.02) | 6.17E-28 | -3.68 (-4.31;-3.04) | 3.58E-28 |
| 1-Methyl-5-Imidazoleacetate | -3.72 (-4.36;-3.08) | 1.42E-28 | -3.70 (-4.34;-3.05) | 4.33E-28 | -3.70 (-4.34;-3.05) | 4.52E-28 |
| Urea | -3.78 (-4.41;-3.14) | 7.88E-30 | -3.71 (-4.36;-3.06) | 4.33E-28 | -3.70 (-4.35;-3.06) | 6.14E-28 |
| Quinolinate | -3.70 (-4.34;-3.05) | 2.57E-28 | -3.81 (-4.48;-3.15) | 5.43E-28 | -3.79 (-4.46;-3.12) | 1.08E-27 |
| Tiglylcarnitine (C5:1-DC) | -3.63 (-4.26;-2.99) | 4.29E-28 | -3.62 (-4.26;-2.98) | 1.46E-27 | -3.64 (-4.28;-3.00) | 1.37E-27 |
| X-23666 | -6.36 (-7.47;-5.25) | 4.61E-28 | -6.34 (-7.46;-5.23) | 1.67E-27 | -6.35 (-7.47;-5.23) | 1.76E-27 |
| N-Acetylglucosaminylasparagine | -3.57 (-4.22;-2.93) | 1.36E-26 | -3.69 (-4.33;-3.04) | 1.43E-27 | -3.67 (-4.32;-3.02) | 2.29E-27 |
| 3-Methylglutaconate | -3.42 (-4.06;-2.78) | 7.25E-25 | -3.78 (-4.46;-3.09) | 3.02E-26 | -3.80 (-4.48;-3.12) | 1.64E-26 |
| Gamma-Glutamylisoleucine* | -3.65 (-4.32;-2.99) | 5.53E-26 | -3.76 (-4.45;-3.08) | 5.08E-26 | -3.81 (-4.50;-3.13) | 1.80E-26 |
| 5-(Galactosylhydroxy)-L-Lysine | -3.47 (-4.11;-2.84) | 1.29E-25 | -3.57 (-4.21;-2.92) | 4.13E-26 | -3.57 (-4.21;-2.92) | 4.04E-26 |
| Succinylcarnitine (C4-DC) | -3.63 (-4.27;-2.99) | 1.08E-27 | -3.62 (-4.27;-2.97) | 1.69E-26 | -3.60 (-4.25;-2.95) | 4.09E-26 |
| 4-Hydroxyphenylacetylglutamine | -3.63 (-4.28;-2.97) | 1.58E-26 | -3.64 (-4.30;-2.98) | 3.92E-26 | -3.64 (-4.30;-2.98) | 4.34E-26 |
| Sulfate* | -3.52 (-4.14;-2.89) | 3.05E-27 | -3.46 (-4.09;-2.84) | 3.31E-26 | -3.45 (-4.08;-2.82) | 5.01E-26 |
| X-12707 | -3.51 (-4.14;-2.87) | 7.12E-26 | -3.55 (-4.20;-2.91) | 8.12E-26 | -3.56 (-4.21;-2.91) | 6.15E-26 |
| 3-Hydroxyphenylacetoylglutamine | -3.52 (-4.15;-2.89) | 8.72E-27 | -3.48 (-4.11;-2.84) | 8.26E-26 | -3.46 (-4.09;-2.83) | 1.48E-25 |
| Indolelactate | -3.63 (-4.29;-2.97) | 5.91E-26 | -3.63 (-4.29;-2.96) | 1.23E-25 | -3.62 (-4.28;-2.96) | 1.55E-25 |
| 11-Ketoetiocholanolone Glucuronide | -3.44 (-4.07;-2.80) | 4.01E-25 | -3.42 (-4.06;-2.77) | 3.47E-24 | -3.45 (-4.10;-2.80) | 1.78E-24 |
| Gamma-CEHC Glucuronide* | -6.00 (-7.08;-4.91) | 3.55E-26 | -5.88 (-6.99;-4.77) | 2.98E-24 | -5.93 (-7.04;-4.82) | 1.95E-24 |
| X-24556 | -3.47 (-4.11;-2.84) | 1.33E-25 | -3.46 (-4.10;-2.82) | 7.91E-25 | -3.43 (-4.08;-2.79) | 2.24E-24 |
| 3-Methyl Catechol Sulfate (1) | -3.19 (-3.83;-2.54) | 2.99E-21 | -3.47 (-4.14;-2.81) | 1.63E-23 | -3.52 (-4.19;-2.86) | 3.40E-24 |
| X-17685 | -3.32 (-3.96;-2.68) | 1.79E-23 | -3.34 (-3.97;-2.70) | 1.86E-23 | -3.36 (-4.00;-2.72) | 7.54E-24 |
| X-12411 | -3.46 (-4.11;-2.80) | 4.75E-24 | -3.45 (-4.11;-2.79) | 8.04E-24 | -3.42 (-4.08;-2.76) | 2.30E-23 |
| Citraconate/Glutaconate | -3.31 (-3.95;-2.67) | 5.79E-23 | -3.36 (-4.01;-2.71) | 6.99E-23 | -3.39 (-4.04;-2.74) | 2.97E-23 |
| X-16580 | -3.21 (-3.85;-2.57) | 8.53E-22 | -3.33 (-3.97;-2.68) | 7.39E-23 | -3.38 (-4.03;-2.73) | 3.08E-23 |
| 6-Hydroxyindole Sulfate | -3.31 (-3.95;-2.67) | 4.69E-23 | -3.32 (-3.96;-2.67) | 1.80E-22 | -3.34 (-3.99;-2.69) | 9.21E-23 |
| 3,4-Dihydroxybutyrate | -3.28 (-3.92;-2.63) | 2.16E-22 | -3.33 (-3.99;-2.68) | 2.08E-22 | -3.36 (-4.02;-2.71) | 1.24E-22 |
| Gamma-Glutamylphenylalanine | -3.43 (-4.09;-2.78) | 1.06E-23 | -3.45 (-4.11;-2.78) | 4.52E-23 | -3.41 (-4.08;-2.74) | 1.63E-22 |
| N-Acetyl-1-Methylhistidine* | -3.21 (-3.85;-2.58) | 5.95E-22 | -3.27 (-3.92;-2.63) | 3.67E-22 | -3.25 (-3.900;-2.61) | 6.22E-22 |
| X-12101 | -3.11 (-3.75;-2.47) | 1.97E-20 | -3.20 (-3.85;-2.56) | 2.97E-21 | -3.27 (-3.92;-2.62) | 6.32E-22 |
| 1-Methyl-5-Imidazolelactate | -3.19 (-3.84;-2.54) | 5.10E-21 | -3.30 (-3.96;-2.64) | 8.02E-22 | -3.30 (-3.96;-2.64) | 8.07E-22 |
| N-Acetyltaurine | -5.74 (-6.91;-4.58) | 4.69E-21 | -5.81 (-6.97;-4.64) | 2.57E-21 | -5.88 (-7.05;-4.70) | 1.16E-21 |
| 3-Methoxycatechol Sulfate (1) | -3.13 (-3.77;-2.50) | 7.45E-21 | -3.14 (-3.78;-2.50) | 7.41E-21 | -3.19 (-3.83;-2.55) | 1.79E-21 |
| X-17346 | -5.86 (-7.00;-4.72) | 1.23E-22 | -5.78 (-6.94;-4.63) | 1.53E-21 | -5.80 (-6.96;-4.64) | 1.85E-21 |
| 3-Methoxycatechol Sulfate (2) | -3.21 (-3.85;-2.57) | 1.93E-21 | -3.17 (-3.82;-2.53) | 8.27E-21 | -3.17 (-3.82;-2.53) | 7.71E-21 |
| 3-Hydroxypyridine Sulfate | -3.03 (-3.68;-2.38) | 4.20E-19 | -3.17 (-3.84;-2.51) | 6.74E-20 | -3.24 (-3.91;-2.58) | 1.07E-20 |
| N-Acetylhistidine | -3.10 (-3.74;-2.45) | 4.90E-20 | -3.19 (-3.85;-2.54) | 1.24E-20 | -3.18 (-3.84;-2.53) | 1.65E-20 |
| Gulonate* | -5.60 (-6.71;-4.49) | 6.80E-22 | -5.43 (-6.56;-4.30) | 4.10E-20 | -5.49 (-6.63;-4.36) | 2.61E-20 |
| X-12713 | -5.64 (-6.81;-4.47) | 4.42E-20 | -5.61 (-6.79;-4.44) | 9.08E-20 | -5.72 (-6.90;-4.54) | 2.77E-20 |
| 2-Methylbutyrylcarnitine (C5) | -3.25 (-3.90;-2.59) | 2.56E-21 | -3.24 (-3.91;-2.57) | 3.86E-20 | -3.26 (-3.94;-2.58) | 3.42E-20 |
| 5-Hydroxyindole Sulfate | -3.10 (-3.74;-2.46) | 1.92E-20 | -3.11 (-3.76;-2.47) | 5.71E-20 | -3.14 (-3.79;-2.49) | 3.93E-20 |
| 2R,3R-Dihydroxybutyrate | -3.13 (-3.76;-2.50) | 4.68E-21 | -3.09 (-3.74;-2.45) | 4.49E-20 | -3.09 (-3.73;-2.45) | 4.71E-20 |
| 8-Methoxykynurenate | -3.18 (-3.83;-2.52) | 2.73E-20 | -3.26 (-3.94;-2.57) | 9.26E-20 | -3.28 (-3.96;-2.59) | 6.40E-20 |
| 4-Vinylguaiacol Sulfate | -2.79 (-3.45;-2.13) | 7.02E-16 | -3.28 (-3.98;-2.57) | 6.25E-19 | -3.38 (-4.08;-2.67) | 6.89E-20 |
| Ascorbic Acid 3-Sulfate* | -3.11 (-3.76;-2.45) | 1.26E-19 | -3.09 (-3.75;-2.43) | 2.76E-19 | -3.13 (-3.79;-2.48) | 9.01E-20 |
| Imidazole Lactate | -3.04 (-3.68;-2.39) | 3.29E-19 | -3.10 (-3.74;-2.45) | 1.01E-19 | -3.10 (-3.75;-2.45) | 9.22E-20 |
| X-21821 | -3.07 (-3.70;-2.43) | 3.85E-20 | -3.11 (-3.75;-2.46) | 6.21E-20 | -3.09 (-3.74;-2.44) | 1.06E-19 |
| 3-Methyl Catechol Sulfate (2) | -2.90 (-3.55;-2.25) | 1.47E-17 | -3.13 (-3.80;-2.46) | 4.43E-19 | -3.18 (-3.85;-2.51) | 1.16E-19 |
| X-12283 | -3.07 (-3.71;-2.43) | 4.42E-20 | -3.12 (-3.77;-2.47) | 7.27E-20 | -3.10 (-3.76;-2.45) | 1.32E-19 |
| 5-Methylthioadenosine (MTA) | -3.03 (-3.67;-2.39) | 1.62E-19 | -3.13 (-3.79;-2.47) | 1.48E-19 | -3.13 (-3.79;-2.47) | 1.50E-19 |
| X-12096 | -5.47 (-6.60;-4.35) | 1.20E-20 | -5.50 (-6.67;-4.32) | 4.23E-19 | -5.58 (-6.76;-4.40) | 1.97E-19 |
| X-22162 | -3.07 (-3.70;-2.43) | 4.84E-20 | -3.05 (-3.69;-2.41) | 9.08E-20 | -3.03 (-3.67;-2.39) | 2.11E-19 |
| Pentose Acid* | -2.95 (-3.59;-2.31) | 9.24E-19 | -3.00 (-3.64;-2.35) | 5.74E-19 | -3.03 (-3.68;-2.39) | 2.26E-19 |
| 3-Amino-2-Piperidone | -3.15 (-3.80;-2.50) | 3.44E-20 | -3.12 (-3.77;-2.46) | 1.30E-19 | -3.10 (-3.76;-2.45) | 2.26E-19 |
| 1-Methylurate | -2.98 (-3.63;-2.33) | 2.23E-18 | -3.07 (-3.72;-2.42) | 2.22E-19 | -3.06 (-3.72;-2.41) | 2.58E-19 |
| X-23593 | -3.00 (-3.65;-2.36) | 6.23E-19 | -3.04 (-3.69;-2.39) | 4.15E-19 | -3.04 (-3.69;-2.39) | 4.03E-19 |
| Adipoylcarnitine (C6-DC) | -3.04 (-3.69;-2.39) | 3.59E-19 | -3.09 (-3.75;-2.44) | 2.76E-19 | -3.07 (-3.73;-2.41) | 5.75E-19 |
| Phenyllactate (PLA) | -3.20 (-3.89;-2.51) | 7.00E-19 | -3.25 (-3.95;-2.56) | 4.43E-19 | -3.24 (-3.94;-2.55) | 6.05E-19 |
| Pimeloylcarnitine/3-Methyladipoylcarnitine (C7-DC) | -3.01 (-3.65;-2.36) | 4.20E-19 | -3.05 (-3.69;-2.40) | 3.03E-19 | -3.04 (-3.69;-2.38) | 6.54E-19 |
| 3-Methoxytyramine Sulfate | -2.87 (-3.51;-2.23) | 9.10E-18 | -2.97 (-3.61;-2.33) | 1.03E-18 | -2.97 (-3.62;-2.33) | 8.16E-19 |
| 1-Methyl-4-Imidazoleacetate | -2.92 (-3.57;-2.28) | 5.43E-18 | -3.03 (-3.68;-2.38) | 5.86E-19 | -3.01 (-3.66;-2.36) | 1.01E-18 |
| Lanthionine | -3.00 (-3.64;-2.36) | 3.80E-19 | -2.95 (-3.59;-2.31) | 2.17E-18 | -2.97 (-3.61;-2.33) | 1.21E-18 |
| 3-Indoxyl Sulfate | -2.98 (-3.63;-2.34) | 8.70E-19 | -2.99 (-3.64;-2.34) | 2.46E-18 | -3.01 (-3.66;-2.36) | 1.51E-18 |
| 5-Hydroxyindoleacetate | -5.47 (-6.64;-4.31) | 2.78E-19 | -5.43 (-6.61;-4.25) | 1.46E-18 | -5.43 (-6.61;-4.25) | 2.08E-18 |
| 3-Methylxanthine | -2.89 (-3.53;-2.26) | 4.64E-18 | -2.91 (-3.55;-2.27) | 3.99E-18 | -2.92 (-3.56;-2.28) | 2.78E-18 |
| N-Acetyl-2-Aminoadipate | -2.90 (-3.54;-2.26) | 3.77E-18 | -3.05 (-3.72;-2.39) | 1.34E-18 | -3.03 (-3.69;-2.37) | 2.87E-18 |
| 3-Hydroxy-3-Methylglutarate | -2.93 (-3.57;-2.29) | 2.23E-18 | -3.08 (-3.76;-2.40) | 3.77E-18 | -3.10 (-3.77;-2.42) | 2.87E-18 |
| 2-O-Methylascorbic Acid | -2.91 (-3.55;-2.28) | 2.95E-18 | -2.96 (-3.61;-2.31) | 4.27E-18 | -2.97 (-3.62;-2.31) | 3.51E-18 |
| Dimethylarginine (SDMA + ADMA) | -2.92 (-3.57;-2.28) | 5.37E-18 | -2.97 (-3.62;-2.32) | 3.27E-18 | -2.96 (-3.61;-2.31) | 5.05E-18 |
| N1-Methyl-2-Pyridone-5-Carboxamide | -2.96 (-3.60;-2.33) | 6.94E-19 | -2.98 (-3.63;-2.33) | 2.06E-18 | -2.94 (-3.59;-2.29) | 6.05E-18 |
| Gluconate | -2.95 (-3.59;-2.31) | 1.25E-18 | -2.88 (-3.52;-2.23) | 1.82E-17 | -2.93 (-3.58;-2.28) | 6.46E-18 |
| 11beta-Hydroxyetiocholanolone Glucuronide* | -2.99 (-3.63;-2.35) | 4.20E-19 | -2.92 (-3.57;-2.27) | 8.06E-18 | -2.93 (-3.58;-2.28) | 7.86E-18 |
| Indoleacetylglutamine | -3.01 (-3.67;-2.35) | 2.23E-18 | -3.00 (-3.66;-2.33) | 8.33E-18 | -3.00 (-3.66;-2.33) | 8.37E-18 |
| 3-Hydroxy-2-Methylpyridine Sulfate | -2.80 (-3.44;-2.15) | 1.27E-16 | -2.94 (-3.61;-2.27) | 4.72E-17 | -3.01 (-3.67;-2.34) | 1.09E-17 |
| Isobutyrylcarnitine (C4) | -3.00 (-3.63;-2.37) | 1.85E-19 | -2.92 (-3.56;-2.27) | 8.75E-18 | -2.90 (-3.55;-2.25) | 1.66E-17 |
| 5-Acetylamino-6-Amino-3-Methyluracil | -2.83 (-3.47;-2.19) | 3.85E-17 | -2.89 (-3.53;-2.24) | 1.31E-17 | -2.87 (-3.52;-2.23) | 1.87E-17 |
| Gamma-Carboxyglutamate | -2.75 (-3.39;-2.11) | 2.10E-16 | -2.84 (-3.48;-2.20) | 2.56E-17 | -2.85 (-3.49;-2.21) | 2.02E-17 |
| X-24337 | -2.89 (-3.55;-2.23) | 4.82E-17 | -2.98 (-3.65;-2.30) | 2.79E-17 | -2.99 (-3.66;-2.32) | 2.13E-17 |
| X-19141 | -2.81 (-3.45;-2.17) | 5.15E-17 | -2.90 (-3.54;-2.25) | 1.11E-17 | -2.87 (-3.51;-2.22) | 2.76E-17 |
| 7-Methylguanine | -2.84 (-3.50;-2.19) | 1.33E-16 | -2.93 (-3.59;-2.27) | 2.55E-17 | -2.92 (-3.58;-2.26) | 3.12E-17 |
| Glutamine Conjugate Of C6H10O2 (1)* | -2.79 (-3.45;-2.13) | 7.56E-16 | -2.87 (-3.53;-2.21) | 1.66E-16 | -2.95 (-3.62;-2.28) | 3.49E-17 |
| 4-Acetamidobutanoate | -2.84 (-3.48;-2.20) | 3.51E-17 | -2.83 (-3.48;-2.19) | 4.45E-17 | -2.83 (-3.48;-2.19) | 4.27E-17 |
| 3-Carboxy-4-Methyl-5-Pentyl-2-Furanpropionate (3-CMPFP)** | -2.93 (-3.57;-2.29) | 3.03E-18 | -2.92 (-3.58;-2.27) | 1.62E-17 | -2.88 (-3.54;-2.23) | 5.44E-17 |
| 3-Acetylphenol Sulfate | -2.74 (-3.38;-2.09) | 7.02E-16 | -2.86 (-3.52;-2.20) | 1.53E-16 | -2.90 (-3.56;-2.24) | 6.17E-17 |
| X-11979 | -5.25 (-6.42;-4.07) | 1.47E-17 | -5.22 (-6.42;-4.03) | 7.63E-17 | -5.24 (-6.43;-4.04) | 7.95E-17 |
| N-(2-Furoyl)Glycine | -2.81 (-3.47;-2.16) | 3.02E-16 | -2.88 (-3.55;-2.21) | 3.90E-16 | -2.95 (-3.63;-2.28) | 8.09E-17 |
| Pantoate | -2.78 (-3.42;-2.14) | 1.64E-16 | -2.82 (-3.46;-2.18) | 7.06E-17 | -2.80 (-3.44;-2.16) | 1.13E-16 |
| Cis-3,4-Methyleneheptanoylglycine | -2.76 (-3.41;-2.11) | 4.34E-16 | -2.84 (-3.49;-2.18) | 1.14E-16 | -2.83 (-3.49;-2.18) | 1.18E-16 |
| Glycine Conjugate Of C10H12O2* | -2.76 (-3.40;-2.12) | 1.71E-16 | -2.78 (-3.42;-2.13) | 1.88E-16 | -2.80 (-3.44;-2.16) | 1.18E-16 |
| X-12221 | -2.83 (-3.48;-2.18) | 9.80E-17 | -2.82 (-3.47;-2.16) | 2.50E-16 | -2.84 (-3.50;-2.19) | 1.28E-16 |
| X-17357 | -2.86 (-3.51;-2.22) | 2.39E-17 | -2.83 (-3.47;-2.18) | 9.39E-17 | -2.82 (-3.47;-2.17) | 1.28E-16 |
| X-16397 | -2.82 (-3.46;-2.19) | 3.04E-17 | -2.87 (-3.51;-2.23) | 1.87E-17 | -2.81 (-3.46;-2.16) | 1.28E-16 |
| 2,3-Dihydroxyisovalerate | -2.55 (-3.19;-1.91) | 4.84E-14 | -2.87 (-3.53;-2.21) | 1.66E-16 | -2.87 (-3.53;-2.21) | 1.66E-16 |
| Catechol Sulfate | -2.75 (-3.39;-2.11) | 2.59E-16 | -2.75 (-3.40;-2.10) | 8.72E-16 | -2.78 (-3.43;-2.13) | 4.01E-16 |
| Mannonate* | -4.74 (-5.88;-3.61) | 1.87E-15 | -4.87 (-6.03;-3.71) | 1.36E-15 | -5.12 (-6.32;-3.92) | 4.34E-16 |
| Ascorbic Acid 2-Sulfate | -2.71 (-3.35;-2.07) | 5.94E-16 | -2.74 (-3.38;-2.10) | 4.87E-16 | -2.74 (-3.39;-2.10) | 4.98E-16 |
| Xanthurenate | -2.91 (-3.57;-2.25) | 5.58E-17 | -2.90 (-3.58;-2.22) | 3.47E-16 | -2.89 (-3.56;-2.21) | 4.99E-16 |
| Hydantoin-5-Propionate | -2.85 (-3.50;-2.21) | 2.94E-17 | -2.86 (-3.53;-2.19) | 4.03E-16 | -2.85 (-3.52;-2.18) | 5.54E-16 |
| Cystathionine | -2.75 (-3.40;-2.11) | 5.21E-16 | -2.75 (-3.41;-2.09) | 2.27E-15 | -2.79 (-3.45;-2.13) | 9.15E-16 |
| 3-(4-Hydroxyphenyl)Lactate | -2.92 (-3.61;-2.23) | 7.40E-16 | -2.96 (-3.66;-2.26) | 8.72E-16 | -2.95 (-3.65;-2.24) | 1.24E-15 |
| Hippurate | -2.82 (-3.47;-2.18) | 6.90E-17 | -2.78 (-3.44;-2.12) | 7.72E-16 | -2.75 (-3.41;-2.10) | 1.49E-15 |
| X-24328 | -3.20 (-3.97;-2.43) | 3.34E-15 | -3.31 (-4.09;-2.52) | 1.04E-15 | -3.27 (-4.06;-2.48) | 2.39E-15 |
| 1,3,7-Trimethylurate | -2.65 (-3.29;-2.00) | 5.84E-15 | -2.77 (-3.44;-2.10) | 4.10E-15 | -2.80 (-3.47;-2.12) | 2.46E-15 |
| 3-Hydroxyhexanoylcarnitine (1) | -2.63 (-3.29;-1.98) | 2.84E-14 | -2.72 (-3.38;-2.05) | 1.00E-14 | -2.78 (-3.46;-2.11) | 2.75E-15 |
| Heptenedioate (C7:1-DC)* | -2.79 (-3.45;-2.14) | 4.10E-16 | -2.78 (-3.44;-2.12) | 8.04E-16 | -2.73 (-3.39;-2.07) | 3.30E-15 |
| X-24422 | -2.53 (-3.18;-1.88) | 1.13E-13 | -2.67 (-3.33;-2.02) | 6.23E-15 | -2.68 (-3.34;-2.03) | 4.74E-15 |
| Isobutyrylglycine | -2.69 (-3.32;-2.05) | 1.05E-15 | -2.67 (-3.31;-2.03) | 2.54E-15 | -2.66 (-3.30;-2.01) | 4.76E-15 |
| X-12216 | -2.67 (-3.31;-2.02) | 4.34E-15 | -2.70 (-3.36;-2.04) | 7.55E-15 | -2.72 (-3.38;-2.06) | 5.13E-15 |
| X-15486 | -2.57 (-3.22;-1.93) | 3.79E-14 | -2.65 (-3.30;-2.00) | 1.31E-14 | -2.67 (-3.33;-2.01) | 9.45E-15 |
| Aconitate [Cis Or Trans] | -2.55 (-3.20;-1.90) | 8.24E-14 | -2.63 (-3.29;-1.97) | 4.42E-14 | -2.70 (-3.37;-2.04) | 1.06E-14 |
| X-24462 | -2.68 (-3.33;-2.04) | 2.17E-15 | -2.65 (-3.29;-2.00) | 6.98E-15 | -2.63 (-3.27;-1.98) | 1.21E-14 |
| Ribonate | -4.68 (-5.82;-3.54) | 5.76E-15 | -4.64 (-5.80;-3.47) | 4.02E-14 | -4.82 (-6.01;-3.63) | 1.39E-14 |
| X-12007 | -4.81 (-5.98;-3.65) | 3.20E-15 | -4.65 (-5.81;-3.49) | 3.12E-14 | -4.75 (-5.92;-3.57) | 1.44E-14 |
| Glycine Conjugate Of C10H14O2 (1)* | -2.54 (-3.18;-1.90) | 4.39E-14 | -2.58 (-3.23;-1.93) | 3.10E-14 | -2.61 (-3.25;-1.96) | 1.90E-14 |
| X-23655 | -2.52 (-3.17;-1.87) | 2.12E-13 | -2.63 (-3.29;-1.96) | 8.81E-14 | -2.69 (-3.36;-2.02) | 2.07E-14 |
| Citrulline | -2.64 (-3.29;-2.00) | 3.90E-15 | -2.65 (-3.30;-1.99) | 1.11E-14 | -2.60 (-3.26;-1.95) | 3.69E-14 |
| O-Cresol Sulfate | -1.99 (-2.64;-1.33) | 1.13E-08 | -2.99 (-3.76;-2.23) | 1.22E-13 | -3.05 (-3.82;-2.29) | 4.02E-14 |
| Phenylacetylglutamate | -2.70 (-3.35;-2.05) | 2.37E-15 | -2.64 (-3.31;-1.97) | 5.58E-14 | -2.65 (-3.32;-1.98) | 5.02E-14 |
| X-25810 | -2.57 (-3.22;-1.93) | 3.36E-14 | -2.59 (-3.24;-1.94) | 4.07E-14 | -2.58 (-3.23;-1.93) | 5.34E-14 |
| 3-Aminoisobutyrate | -2.63 (-3.29;-1.98) | 2.33E-14 | -2.63 (-3.29;-1.97) | 3.42E-14 | -2.61 (-3.27;-1.95) | 5.60E-14 |
| X-23739 | -2.57 (-3.22;-1.92) | 4.96E-14 | -2.58 (-3.23;-1.92) | 7.21E-14 | -2.58 (-3.24;-1.93) | 6.36E-14 |
| Gamma-Glutamylleucine | -2.79 (-3.48;-2.10) | 1.16E-14 | -2.78 (-3.48;-2.08) | 5.61E-14 | -2.77 (-3.47;-2.06) | 7.39E-14 |
| Glucuronate | -2.52 (-3.17;-1.88) | 1.35E-13 | -2.54 (-3.19;-1.89) | 1.39E-13 | -2.56 (-3.21;-1.90) | 1.20E-13 |
| 3-Hydroxy-2-Ethylpropionate | -2.51 (-3.16;-1.86) | 2.61E-13 | -2.55 (-3.21;-1.89) | 1.68E-13 | -2.57 (-3.23;-1.91) | 1.64E-13 |
| 1,7-Dimethylurate | -2.52 (-3.17;-1.87) | 1.57E-13 | -2.58 (-3.24;-1.92) | 1.57E-13 | -2.58 (-3.24;-1.91) | 1.87E-13 |
| X-21383 | -2.49 (-3.13;-1.85) | 1.58E-13 | -2.52 (-3.17;-1.88) | 1.15E-13 | -2.50 (-3.15;-1.86) | 1.93E-13 |
| N-Acetylisoleucine | -2.62 (-3.27;-1.96) | 3.12E-14 | -2.60 (-3.28;-1.92) | 2.85E-13 | -2.60 (-3.28;-1.92) | 3.24E-13 |
| 3-Hydroxyoctanoylcarnitine (1) | -2.45 (-3.11;-1.78) | 2.70E-12 | -2.54 (-3.21;-1.87) | 5.97E-13 | -2.55 (-3.22;-1.88) | 5.03E-13 |
| 2-Aminophenol Sulfate | -2.50 (-3.14;-1.85) | 2.88E-13 | -2.49 (-3.14;-1.83) | 4.92E-13 | -2.48 (-3.13;-1.82) | 6.24E-13 |
| Beta-Hydroxyisovaleroylcarnitine | -2.54 (-3.19;-1.90) | 5.88E-14 | -2.47 (-3.12;-1.82) | 4.09E-13 | -2.45 (-3.10;-1.81) | 6.71E-13 |
| Pyridoxate | -2.45 (-3.09;-1.81) | 4.46E-13 | -2.52 (-3.17;-1.86) | 2.36E-13 | -2.48 (-3.13;-1.82) | 7.08E-13 |
| Trigonelline (N'-Methylnicotinate) | -2.37 (-3.01;-1.72) | 3.19E-12 | -2.48 (-3.13;-1.83) | 5.72E-13 | -2.46 (-3.11;-1.81) | 8.27E-13 |
| Gamma-Glutamylvaline | -2.51 (-3.16;-1.86) | 2.46E-13 | -2.50 (-3.16;-1.83) | 8.76E-13 | -2.50 (-3.16;-1.84) | 9.01E-13 |
| 3-Formylindole | -2.63 (-3.30;-1.96) | 1.18E-13 | -2.59 (-3.27;-1.91) | 4.25E-13 | -2.56 (-3.24;-1.88) | 9.01E-13 |
| Retinol (Vitamin A) | -2.46 (-3.10;-1.81) | 3.68E-13 | -2.47 (-3.12;-1.82) | 7.62E-13 | -2.47 (-3.12;-1.81) | 9.29E-13 |
| N6,N6,N6-Trimethyllysine | -2.61 (-3.27;-1.96) | 3.74E-14 | -2.54 (-3.19;-1.88) | 2.34E-13 | -2.50 (-3.16;-1.83) | 9.92E-13 |
| X-24344 | -2.04 (-2.69;-1.38) | 4.87E-09 | -2.67 (-3.39;-1.94) | 3.47E-12 | -2.73 (-3.46;-2.00) | 1.06E-12 |
| 3-Ethylcatechol Sulfate (1) | -2.12 (-2.77;-1.48) | 6.12E-10 | -2.55 (-3.23;-1.86) | 2.16E-12 | -2.58 (-3.27;-1.89) | 1.10E-12 |
| N-Acetyl-Isoputreanine | -2.40 (-3.04;-1.75) | 1.70E-12 | -2.42 (-3.07;-1.77) | 1.55E-12 | -2.43 (-3.08;-1.78) | 1.14E-12 |
| Xanthosine | -4.42 (-5.59;-3.25) | 6.06E-13 | -4.41 (-5.59;-3.22) | 1.89E-12 | -4.44 (-5.63;-3.24) | 1.80E-12 |
| Cinnamoylglycine | -2.39 (-3.04;-1.75) | 2.28E-12 | -2.43 (-3.09;-1.77) | 2.48E-12 | -2.44 (-3.10;-1.78) | 2.22E-12 |
| X-23680 | -2.49 (-3.13;-1.84) | 2.37E-13 | -2.42 (-3.07;-1.77) | 1.80E-12 | -2.41 (-3.06;-1.76) | 2.29E-12 |
| Prolylglycine | -4.29 (-5.44;-3.14) | 1.48E-12 | -4.29 (-5.45;-3.13) | 2.46E-12 | -4.30 (-5.47;-3.14) | 2.33E-12 |
| X-12407 | -2.42 (-3.07;-1.78) | 8.34E-13 | -2.40 (-3.04;-1.75) | 2.42E-12 | -2.40 (-3.05;-1.75) | 2.69E-12 |
| Urate | -2.51 (-3.19;-1.83) | 3.26E-12 | -2.65 (-3.36;-1.94) | 1.58E-12 | -2.62 (-3.33;-1.91) | 2.83E-12 |
| Argininate* | -2.49 (-3.14;-1.84) | 3.98E-13 | -2.45 (-3.11;-1.78) | 2.72E-12 | -2.44 (-3.10;-1.78) | 3.36E-12 |
| 4-Ethylcatechol Sulfate | -2.31 (-2.96;-1.66) | 1.68E-11 | -2.41 (-3.08;-1.75) | 6.12E-12 | -2.44 (-3.10;-1.77) | 3.39E-12 |
| Cysteinylglycine Disulfide* | -2.45 (-3.10;-1.81) | 3.98E-13 | -2.39 (-3.03;-1.74) | 2.14E-12 | -2.35 (-2.99;-1.70) | 5.95E-12 |
| Sucrose | -4.64 (-5.85;-3.42) | 4.09E-13 | -4.46 (-5.72;-3.20) | 1.98E-11 | -4.58 (-5.85;-3.31) | 8.08E-12 |
| N4-Acetylcytidine | -2.37 (-3.01;-1.73) | 3.03E-12 | -2.36 (-3.02;-1.71) | 9.03E-12 | -2.36 (-3.02;-1.71) | 9.50E-12 |
| X-24518 | -2.33 (-2.99;-1.67) | 1.83E-11 | -2.43 (-3.12;-1.75) | 1.90E-11 | -2.46 (-3.15;-1.77) | 1.11E-11 |
| X-24812 | -1.77 (-2.41;-1.12) | 3.11E-07 | -2.74 (-3.51;-1.97) | 1.22E-11 | -2.75 (-3.52;-1.98) | 1.13E-11 |
| Deoxycarnitine | -2.42 (-3.09;-1.75) | 8.33E-12 | -2.48 (-3.15;-1.80) | 3.40E-12 | -2.43 (-3.11;-1.75) | 1.16E-11 |
| Adenosine 3',5'-Cyclic Monophosphate (Camp) | -2.36 (-3.02;-1.71) | 6.49E-12 | -2.35 (-3.00;-1.69) | 9.50E-12 | -2.33 (-2.99;-1.68) | 1.27E-11 |
| Phenylacetylglutamine | -2.45 (-3.10;-1.80) | 1.04E-12 | -2.39 (-3.05;-1.72) | 1.03E-11 | -2.38 (-3.04;-1.71) | 1.30E-11 |
| 2-Hydroxyphenylacetate | -2.42 (-3.07;-1.76) | 2.53E-12 | -2.37 (-3.03;-1.71) | 8.75E-12 | -2.36 (-3.02;-1.70) | 1.30E-11 |
| X-24811 | -2.17 (-2.82;-1.51) | 3.34E-10 | -2.38 (-3.05;-1.71) | 1.67E-11 | -2.39 (-3.06;-1.72) | 1.33E-11 |
| Decadienedioic Acid (C10:2-DC)** | -2.22 (-2.87;-1.57) | 8.07E-11 | -2.32 (-2.97;-1.67) | 1.57E-11 | -2.32 (-2.97;-1.67) | 1.45E-11 |
| N-Delta-Acetylornithine | -2.30 (-2.94;-1.66) | 1.24E-11 | -2.33 (-2.98;-1.68) | 1.02E-11 | -2.31 (-2.96;-1.66) | 1.58E-11 |
| X-24588 | -2.32 (-2.98;-1.66) | 2.53E-11 | -2.42 (-3.09;-1.75) | 9.93E-12 | -2.39 (-3.07;-1.72) | 1.78E-11 |
| Ectoine | -2.38 (-3.04;-1.72) | 6.48E-12 | -2.37 (-3.03;-1.72) | 7.59E-12 | -2.33 (-2.99;-1.67) | 1.96E-11 |
| 11beta-Hydroxyandrosterone Glucuronide | -2.25 (-2.91;-1.60) | 7.84E-11 | -2.24 (-2.90;-1.57) | 1.75E-10 | -2.36 (-3.03;-1.70) | 2.11E-11 |
| Trimethylamine N-Oxide | -2.31 (-2.95;-1.66) | 1.32E-11 | -2.25 (-2.90;-1.60) | 5.08E-11 | -2.29 (-2.94;-1.64) | 2.32E-11 |
| N-Acetyltryptophan | -2.37 (-3.06;-1.68) | 7.57E-11 | -2.47 (-3.17;-1.76) | 2.97E-11 | -2.47 (-3.18;-1.77) | 2.84E-11 |
| 7-Methylxanthine | -2.19 (-2.83;-1.54) | 1.41E-10 | -2.26 (-2.91;-1.62) | 3.82E-11 | -2.28 (-2.92;-1.63) | 2.89E-11 |
| 4-Vinylcatechol Sulfate | -2.05 (-2.71;-1.39) | 4.48E-09 | -2.39 (-3.09;-1.70) | 6.01E-11 | -2.43 (-3.12;-1.73) | 3.19E-11 |
| X-12410 | -2.38 (-3.03;-1.73) | 3.02E-12 | -2.31 (-2.96;-1.66) | 1.88E-11 | -2.28 (-2.94;-1.63) | 3.48E-11 |
| Etiocholanolone Glucuronide | -2.18 (-2.84;-1.52) | 3.80E-10 | -2.32 (-2.99;-1.65) | 6.52E-11 | -2.33 (-3.00;-1.66) | 5.01E-11 |
| N-Acetylleucine | -2.38 (-3.05;-1.71) | 1.26E-11 | -2.41 (-3.11;-1.71) | 6.81E-11 | -2.43 (-3.13;-1.73) | 5.41E-11 |
| 4-Guanidinobutanoate | -2.33 (-3.03;-1.62) | 3.98E-10 | -2.40 (-3.10;-1.69) | 1.10E-10 | -2.42 (-3.13;-1.72) | 7.76E-11 |
| Hydroquinone Sulfate | -2.24 (-2.88;-1.59) | 6.86E-11 | -2.21 (-2.86;-1.55) | 1.76E-10 | -2.25 (-2.90;-1.59) | 8.39E-11 |
| Gamma-Glutamylcitrulline* | -2.23 (-2.87;-1.59) | 5.33E-11 | -2.31 (-2.97;-1.66) | 2.93E-11 | -2.27 (-2.94;-1.61) | 9.64E-11 |
| N-Acetylphenylalanine | -2.33 (-3.01;-1.65) | 9.11E-11 | -2.41 (-3.11;-1.71) | 7.82E-11 | -2.40 (-3.10;-1.70) | 1.01E-10 |
| Methyl Indole-3-Acetate | -2.32 (-2.97;-1.67) | 1.55E-11 | -2.30 (-2.96;-1.64) | 4.44E-11 | -2.26 (-2.93;-1.60) | 1.06E-10 |
| 3,7-Dimethylurate | -2.23 (-2.88;-1.59) | 5.52E-11 | -2.18 (-2.83;-1.54) | 1.61E-10 | -2.19 (-2.84;-1.54) | 1.47E-10 |
| 2,6-Dihydroxybenzoic Acid | -2.19 (-2.83;-1.54) | 1.33E-10 | -2.16 (-2.82;-1.51) | 3.62E-10 | -2.19 (-2.84;-1.53) | 2.41E-10 |
| X-17367 | -2.27 (-2.91;-1.63) | 2.46E-11 | -2.19 (-2.84;-1.54) | 1.97E-10 | -2.17 (-2.82;-1.52) | 2.75E-10 |
| 4-Hydroxyhippurate | -2.23 (-2.88;-1.59) | 4.95E-11 | -2.19 (-2.84;-1.53) | 2.52E-10 | -2.17 (-2.82;-1.51) | 4.11E-10 |
| X-13728 | -2.16 (-2.81;-1.52) | 2.18E-10 | -2.14 (-2.79;-1.50) | 3.37E-10 | -2.13 (-2.78;-1.49) | 4.35E-10 |
| N-Acetylglucosamine/N-Acetylgalactosamine | -2.18 (-2.82;-1.53) | 2.11E-10 | -2.19 (-2.85;-1.54) | 2.48E-10 | -2.16 (-2.81;-1.50) | 5.52E-10 |
| Imidazole Propionate | -2.14 (-2.82;-1.47) | 2.49E-09 | -2.22 (-2.92;-1.53) | 1.37E-09 | -2.28 (-2.98;-1.59) | 5.62E-10 |
| 1,3-Dimethylurate | -2.06 (-2.71;-1.42) | 1.75E-09 | -2.12 (-2.77;-1.47) | 6.84E-10 | -2.12 (-2.77;-1.47) | 7.69E-10 |
| Tetrahydrocortisone Glucuronide (5) | -2.18 (-2.85;-1.52) | 6.50E-10 | -2.23 (-2.91;-1.54) | 9.48E-10 | -2.25 (-2.94;-1.56) | 7.85E-10 |
| Vanillic Acid Glycine | -2.15 (-2.79;-1.50) | 3.25E-10 | -2.11 (-2.76;-1.46) | 1.06E-09 | -2.12 (-2.77;-1.47) | 9.02E-10 |
| Gamma-Glutamylglycine | -2.00 (-2.65;-1.35) | 7.48E-09 | -2.21 (-2.88;-1.54) | 6.27E-10 | -2.20 (-2.88;-1.52) | 9.54E-10 |
| N-Acetyl-2-Aminooctanoate* | -2.17 (-2.84;-1.51) | 7.83E-10 | -2.20 (-2.87;-1.53) | 6.99E-10 | -2.19 (-2.86;-1.51) | 9.71E-10 |
| 3-Methylhistidine | -2.06 (-2.71;-1.41) | 1.88E-09 | -2.09 (-2.74;-1.44) | 1.22E-09 | -2.09 (-2.74;-1.44) | 1.12E-09 |
| Cytidine | -2.14 (-2.78;-1.49) | 4.33E-10 | -2.09 (-2.74;-1.44) | 1.25E-09 | -2.10 (-2.75;-1.45) | 1.17E-09 |
| Glutamine Conjugate Of C6H10O2 (2)* | -1.99 (-2.65;-1.32) | 1.79E-08 | -2.11 (-2.78;-1.43) | 3.71E-09 | -2.18 (-2.85;-1.50) | 1.23E-09 |
| Cytosine | -2.05 (-2.69;-1.40) | 2.45E-09 | -2.06 (-2.70;-1.41) | 2.39E-09 | -2.08 (-2.73;-1.43) | 1.45E-09 |
| 3-Hydroxydecanoylcarnitine | -2.09 (-2.75;-1.42) | 4.30E-09 | -2.15 (-2.82;-1.48) | 1.85E-09 | -2.16 (-2.83;-1.49) | 1.52E-09 |
| (S)-3-Hydroxybutyrylcarnitine | -1.91 (-2.57;-1.25) | 5.27E-08 | -2.06 (-2.74;-1.37) | 1.43E-08 | -2.22 (-2.91;-1.53) | 1.55E-09 |
| Succinoyltaurine | -3.98 (-5.14;-2.83) | 6.34E-11 | -3.77 (-4.94;-2.59) | 1.32E-09 | -3.76 (-4.93;-2.58) | 1.59E-09 |
| Gamma-CEHC | -2.15 (-2.79;-1.51) | 2.80E-10 | -2.10 (-2.75;-1.44) | 1.59E-09 | -2.09 (-2.75;-1.44) | 1.79E-09 |
| 1H-Indole-7-Acetic Acid | -2.14 (-2.78;-1.49) | 4.32E-10 | -2.11 (-2.76;-1.46) | 8.30E-10 | -2.07 (-2.73;-1.42) | 1.94E-09 |
| N-Acetylkynurenine (2) | -2.16 (-2.85;-1.47) | 3.20E-09 | -2.26 (-2.96;-1.55) | 1.42E-09 | -2.23 (-2.94;-1.53) | 2.21E-09 |
| X-23648 | -3.82 (-4.97;-2.66) | 5.08E-10 | -3.73 (-4.90;-2.55) | 2.38E-09 | -3.74 (-4.92;-2.56) | 2.21E-09 |
| 3-Hydroxybutyroylglycine** | -1.98 (-2.62;-1.33) | 9.92E-09 | -2.04 (-2.70;-1.39) | 4.13E-09 | -2.06 (-2.72;-1.41) | 2.96E-09 |
| Mannitol/Sorbitol | -1.99 (-2.64;-1.35) | 5.63E-09 | -2.01 (-2.66;-1.36) | 5.99E-09 | -2.05 (-2.70;-1.40) | 3.08E-09 |
| P-Cresol Sulfate | -2.18 (-2.83;-1.53) | 2.78E-10 | -2.09 (-2.76;-1.43) | 2.57E-09 | -2.08 (-2.75;-1.42) | 3.14E-09 |
| Quinate | -1.93 (-2.58;-1.28) | 1.90E-08 | -2.03 (-2.68;-1.37) | 5.97E-09 | -2.05 (-2.71;-1.39) | 3.86E-09 |
| Isovalerylglycine | -2.01 (-2.66;-1.37) | 3.55E-09 | -2.01 (-2.65;-1.36) | 4.54E-09 | -2.01 (-2.66;-1.37) | 3.93E-09 |
| Androsterone Glucuronide | -2.23 (-2.95;-1.52) | 3.52E-09 | -2.19 (-2.91;-1.48) | 8.16E-09 | -2.24 (-2.95;-1.52) | 4.01E-09 |
| Serine | 2.02 (1.37;2.66) | 3.81E-09 | 2.02 (1.36;2.67) | 6.27E-09 | 2.04 (1.38;2.70) | 4.45E-09 |
| Lyxonate | -2.01 (-2.65;-1.37) | 3.92E-09 | -2.00 (-2.65;-1.36) | 5.27E-09 | -2.01 (-2.65;-1.36) | 5.19E-09 |
| 3-Hydroxyoctanoylcarnitine (2) | -1.98 (-2.65;-1.32) | 2.34E-08 | -2.08 (-2.75;-1.40) | 6.32E-09 | -2.09 (-2.76;-1.41) | 5.85E-09 |
| N-Acetylproline | -2.08 (-2.73;-1.43) | 1.97E-09 | -2.05 (-2.72;-1.39) | 7.11E-09 | -2.06 (-2.73;-1.40) | 6.25E-09 |
| Glutamine Conjugate Of C7H12O2* | -1.95 (-2.62;-1.29) | 3.71E-08 | -2.04 (-2.72;-1.37) | 1.01E-08 | -2.07 (-2.75;-1.40) | 6.46E-09 |
| Homovanillate (HVA) | -1.99 (-2.64;-1.34) | 8.47E-09 | -2.02 (-2.67;-1.37) | 4.89E-09 | -2.00 (-2.66;-1.35) | 6.60E-09 |
| X-13729 | -3.66 (-4.85;-2.46) | 9.00E-09 | -3.72 (-4.93;-2.52) | 6.27E-09 | -3.71 (-4.92;-2.50) | 7.95E-09 |
| X-21829 | -2.23 (-3.02;-1.45) | 7.97E-08 | -2.41 (-3.22;-1.60) | 2.03E-08 | -2.46 (-3.27;-1.66) | 9.22E-09 |
| 2-Hydroxyoctanoate | -1.94 (-2.60;-1.29) | 2.12E-08 | -2.01 (-2.67;-1.36) | 6.38E-09 | -2.00 (-2.65;-1.34) | 9.56E-09 |
| 3-Indoleglyoxylic Acid | -3.71 (-4.90;-2.53) | 3.55E-09 | -3.61 (-4.80;-2.41) | 1.35E-08 | -3.65 (-4.85;-2.45) | 9.77E-09 |
| Orotate | -2.04 (-2.69;-1.39) | 3.83E-09 | -2.00 (-2.66;-1.35) | 8.51E-09 | -1.99 (-2.65;-1.34) | 9.95E-09 |
| X-17354 | -2.01 (-2.66;-1.36) | 4.45E-09 | -2.02 (-2.68;-1.35) | 1.03E-08 | -2.01 (-2.68;-1.35) | 1.06E-08 |
| X-17351 | -3.62 (-4.80;-2.44) | 6.76E-09 | -3.64 (-4.85;-2.42) | 1.74E-08 | -3.69 (-4.91;-2.47) | 1.18E-08 |
| 2,3-Dihydroxy-2-Methylbutyrate | -3.70 (-4.88;-2.52) | 3.30E-09 | -3.59 (-4.77;-2.40) | 1.22E-08 | -3.58 (-4.77;-2.39) | 1.42E-08 |
| Galactonate | -3.72 (-4.89;-2.54) | 2.33E-09 | -3.58 (-4.77;-2.39) | 1.42E-08 | -3.58 (-4.77;-2.39) | 1.61E-08 |
| X-17325 | -2.05 (-2.70;-1.41) | 1.97E-09 | -1.97 (-2.62;-1.32) | 1.23E-08 | -1.96 (-2.61;-1.30) | 1.66E-08 |
| 3-Hydroxypyridine Glucuronide | -3.75 (-4.92;-2.58) | 1.56E-09 | -3.60 (-4.81;-2.40) | 1.81E-08 | -3.63 (-4.84;-2.42) | 1.67E-08 |
| X-24757 | -2.01 (-2.65;-1.36) | 4.30E-09 | -1.93 (-2.58;-1.28) | 2.38E-08 | -1.92 (-2.57;-1.27) | 2.93E-08 |
| X-16087 | -1.92 (-2.58;-1.27) | 3.26E-08 | -1.96 (-2.62;-1.29) | 3.31E-08 | -1.96 (-2.63;-1.29) | 3.38E-08 |
| X-18887 | -1.90 (-2.56;-1.24) | 5.40E-08 | -1.98 (-2.65;-1.32) | 1.96E-08 | -1.97 (-2.64;-1.30) | 3.51E-08 |
| X-12830 | -1.88 (-2.53;-1.24) | 4.26E-08 | -1.94 (-2.59;-1.29) | 2.03E-08 | -1.90 (-2.55;-1.25) | 4.41E-08 |
| Cortolone Glucuronide (1) | -1.86 (-2.51;-1.21) | 8.20E-08 | -2.01 (-2.70;-1.31) | 5.07E-08 | -2.02 (-2.71;-1.33) | 4.50E-08 |
| Cys-Gly, Oxidized | -1.96 (-2.61;-1.30) | 1.60E-08 | -1.96 (-2.61;-1.30) | 1.83E-08 | -1.91 (-2.57;-1.25) | 4.58E-08 |
| X-12738 | -3.66 (-4.86;-2.46) | 9.25E-09 | -3.57 (-4.80;-2.35) | 4.32E-08 | -3.58 (-4.81;-2.35) | 4.61E-08 |
| 3-(3-Hydroxyphenyl)Propionate Sulfate | -1.93 (-2.57;-1.28) | 2.09E-08 | -1.91 (-2.56;-1.26) | 3.36E-08 | -1.89 (-2.54;-1.24) | 4.91E-08 |
| X-22509 | -1.93 (-2.57;-1.28) | 2.01E-08 | -1.92 (-2.58;-1.27) | 4.07E-08 | -1.91 (-2.57;-1.25) | 4.91E-08 |
| X-26106 | -1.85 (-2.51;-1.18) | 1.80E-07 | -1.93 (-2.60;-1.27) | 5.46E-08 | -1.94 (-2.61;-1.27) | 5.59E-08 |
| 3-Hydroxyhippurate | -1.93 (-2.58;-1.29) | 1.85E-08 | -1.91 (-2.56;-1.26) | 3.81E-08 | -1.89 (-2.54;-1.23) | 5.60E-08 |
| 4-Acetylphenol Sulfate | -1.89 (-2.55;-1.24) | 5.25E-08 | -1.91 (-2.57;-1.24) | 6.57E-08 | -1.91 (-2.57;-1.25) | 6.23E-08 |
| Dihydroorotate | -1.98 (-2.63;-1.32) | 1.24E-08 | -1.93 (-2.59;-1.26) | 4.54E-08 | -1.91 (-2.57;-1.24) | 6.51E-08 |
| Creatine | 2.02 (1.30;2.75) | 1.67E-07 | 2.14 (1.40;2.87) | 4.54E-08 | 2.10 (1.37;2.84) | 8.10E-08 |
| X-12015 | -3.40 (-4.60;-2.21) | 8.64E-08 | -3.44 (-4.64;-2.23) | 8.37E-08 | -3.45 (-4.66;-2.24) | 8.18E-08 |
| Dimethylglycine | -1.86 (-2.52;-1.19) | 1.59E-07 | -1.91 (-2.58;-1.24) | 9.17E-08 | -1.91 (-2.58;-1.24) | 8.25E-08 |
| 3-Methoxytyrosine | -1.95 (-2.60;-1.30) | 1.75E-08 | -1.94 (-2.60;-1.28) | 2.75E-08 | -1.88 (-2.54;-1.22) | 8.55E-08 |
| N1-Methyladenosine | -1.90 (-2.54;-1.25) | 3.70E-08 | -1.89 (-2.54;-1.24) | 4.72E-08 | -1.85 (-2.50;-1.20) | 9.11E-08 |
| X-14082 | -3.60 (-4.81;-2.40) | 1.91E-08 | -3.45 (-4.66;-2.23) | 9.30E-08 | -3.47 (-4.69;-2.25) | 9.35E-08 |
| Beta-Hydroxyisovalerate | -1.86 (-2.52;-1.19) | 1.56E-07 | -1.90 (-2.57;-1.23) | 9.70E-08 | -1.90 (-2.57;-1.23) | 9.37E-08 |
| Formiminoglutamate | -1.95 (-2.64;-1.26) | 1.04E-07 | -2.06 (-2.78;-1.33) | 1.06E-07 | -2.07 (-2.80;-1.34) | 9.48E-08 |
| Tetrahydrocortisol Glucuronide | -1.90 (-2.58;-1.23) | 1.20E-07 | -1.95 (-2.64;-1.26) | 1.27E-07 | -1.97 (-2.67;-1.28) | 1.03E-07 |
| 4-Vinylphenol Sulfate | -1.65 (-2.33;-0.98) | 4.63E-06 | -1.93 (-2.63;-1.24) | 1.76E-07 | -1.97 (-2.66;-1.27) | 1.03E-07 |
| Indolin-2-One | -1.92 (-2.57;-1.27) | 2.60E-08 | -1.87 (-2.53;-1.21) | 1.01E-07 | -1.87 (-2.53;-1.21) | 1.03E-07 |
| 3-Hydroxyhippurate Sulfate | -3.54 (-4.73;-2.35) | 2.36E-08 | -3.37 (-4.57;-2.17) | 1.41E-07 | -3.39 (-4.60;-2.19) | 1.21E-07 |
| Cortisone | 1.75 (1.11;2.40) | 4.02E-07 | 1.78 (1.13;2.44) | 3.64E-07 | 1.85 (1.19;2.51) | 1.29E-07 |
| Delta-CEHC | -3.20 (-4.37;-2.03) | 2.84E-07 | -3.23 (-4.40;-2.06) | 2.26E-07 | -3.28 (-4.46;-2.11) | 1.62E-07 |
| Cis-3,4-Methyleneheptanoylcarnitine | -1.82 (-2.46;-1.17) | 1.28E-07 | -1.84 (-2.50;-1.18) | 1.67E-07 | -1.84 (-2.50;-1.18) | 1.62E-07 |
| X-24456 | -1.87 (-2.52;-1.22) | 7.30E-08 | -1.84 (-2.49;-1.18) | 1.59E-07 | -1.84 (-2.49;-1.18) | 1.67E-07 |
| Methylsuccinate | -1.85 (-2.49;-1.20) | 8.30E-08 | -1.83 (-2.48;-1.18) | 1.17E-07 | -1.81 (-2.46;-1.16) | 1.73E-07 |
| 3-Hydroxysebacate | -1.66 (-2.34;-0.98) | 5.83E-06 | -1.82 (-2.51;-1.13) | 9.24E-07 | -1.94 (-2.64;-1.24) | 1.91E-07 |
| N-Acetylcitrulline | -1.90 (-2.56;-1.25) | 4.42E-08 | -1.85 (-2.52;-1.18) | 1.92E-07 | -1.85 (-2.52;-1.18) | 1.91E-07 |
| X-12193 | -1.74 (-2.39;-1.09) | 5.00E-07 | -1.80 (-2.45;-1.15) | 2.22E-07 | -1.80 (-2.45;-1.15) | 2.16E-07 |
| N-Methylpipecolate | -1.83 (-2.48;-1.18) | 1.14E-07 | -1.81 (-2.46;-1.16) | 1.55E-07 | -1.79 (-2.44;-1.14) | 2.18E-07 |
| 3-Methylcrotonylglycine | -2.15 (-2.92;-1.39) | 1.21E-07 | -2.13 (-2.90;-1.36) | 2.09E-07 | -2.12 (-2.89;-1.35) | 2.39E-07 |
| X-13695 | -1.84 (-2.50;-1.18) | 1.59E-07 | -1.79 (-2.46;-1.12) | 5.59E-07 | -1.83 (-2.50;-1.16) | 2.93E-07 |
| Undecenoylcarnitine (C11:1) | -1.90 (-2.56;-1.25) | 5.25E-08 | -1.85 (-2.51;-1.19) | 1.47E-07 | -1.81 (-2.47;-1.15) | 3.01E-07 |
| X-25519 | -3.28 (-4.45;-2.12) | 1.26E-07 | -3.19 (-4.39;-2.00) | 5.73E-07 | -3.42 (-4.67;-2.16) | 3.34E-07 |
| X-24494 | -1.65 (-2.30;-1.00) | 2.30E-06 | -1.77 (-2.43;-1.11) | 5.71E-07 | -1.79 (-2.45;-1.13) | 4.29E-07 |
| X-17335 | -1.69 (-2.34;-1.03) | 1.65E-06 | -1.77 (-2.44;-1.11) | 5.77E-07 | -1.79 (-2.46;-1.13) | 4.50E-07 |
| Ethyl Alpha-Glucopyranoside | -1.25 (-1.92;-0.59) | 6.04E-04 | -2.01 (-2.77;-1.26) | 5.59E-07 | -2.02 (-2.78;-1.27) | 4.86E-07 |
| 2-Hydroxy-4-(Methylthio)Butanoic Acid | -1.80 (-2.46;-1.13) | 4.80E-07 | -1.78 (-2.46;-1.11) | 7.56E-07 | -1.81 (-2.48;-1.13) | 5.26E-07 |
| 2-Pyrrolidinone | -1.71 (-2.36;-1.07) | 7.33E-07 | -1.74 (-2.39;-1.09) | 5.26E-07 | -1.74 (-2.39;-1.09) | 5.59E-07 |
| Acisoga | -1.74 (-2.39;-1.09) | 5.09E-07 | -1.70 (-2.35;-1.04) | 1.28E-06 | -1.74 (-2.39;-1.08) | 6.93E-07 |
| Phenol Sulfate | -1.69 (-2.34;-1.04) | 1.06E-06 | -1.70 (-2.35;-1.05) | 1.10E-06 | -1.72 (-2.37;-1.07) | 7.81E-07 |
| X-23641 | -3.32 (-4.58;-2.05) | 8.82E-07 | -3.35 (-4.61;-2.08) | 7.17E-07 | -3.34 (-4.60;-2.07) | 7.93E-07 |
| N-Acetylputrescine | -1.58 (-2.23;-0.93) | 6.12E-06 | -1.74 (-2.40;-1.08) | 7.98E-07 | -1.73 (-2.39;-1.07) | 9.47E-07 |
| Dodecanedioate (C12-DC) | -1.58 (-2.24;-0.91) | 1.01E-05 | -1.71 (-2.39;-1.04) | 2.27E-06 | -1.76 (-2.43;-1.08) | 1.21E-06 |
| 2,3-Dihydroxypyridine | -1.70 (-2.35;-1.04) | 1.22E-06 | -1.73 (-2.39;-1.06) | 1.31E-06 | -1.72 (-2.39;-1.05) | 1.42E-06 |
| Suberoylcarnitine (C8-DC) | -3.16 (-4.39;-1.92) | 1.89E-06 | -3.20 (-4.44;-1.96) | 1.54E-06 | -3.20 (-4.45;-1.96) | 1.68E-06 |
| X-11444 | -1.79 (-2.45;-1.13) | 4.02E-07 | -1.69 (-2.36;-1.03) | 2.26E-06 | -1.71 (-2.38;-1.04) | 1.90E-06 |
| (2,40 Or 2,50)-Dimethylphenol Sulfate | -2.27 (-3.48;-1.06) | 6.34E-04 | -3.74 (-5.21;-2.27) | 2.12E-06 | -3.76 (-5.23;-2.28) | 2.09E-06 |
| X-25948 | -3.20 (-4.37;-2.03) | 2.84E-07 | -3.03 (-4.21;-1.84) | 1.78E-06 | -3.01 (-4.20;-1.82) | 2.29E-06 |
| Ethylmalonate | -1.72 (-2.37;-1.07) | 8.09E-07 | -1.69 (-2.34;-1.04) | 1.31E-06 | -1.65 (-2.30;-1.00) | 2.34E-06 |
| 3-Ureidopropionate | -1.78 (-2.48;-1.08) | 2.32E-06 | -1.78 (-2.49;-1.07) | 2.74E-06 | -1.79 (-2.49;-1.08) | 2.60E-06 |
| X-11787 | -1.71 (-2.36;-1.06) | 9.87E-07 | -1.67 (-2.33;-1.01) | 2.19E-06 | -1.65 (-2.31;-0.99) | 3.04E-06 |
| Dopamine 3-O-Sulfate | -1.63 (-2.28;-0.98) | 3.00E-06 | -1.60 (-2.26;-0.94) | 5.69E-06 | -1.64 (-2.30;-0.99) | 3.08E-06 |
| 4-Methylhexanoylglutamine | -1.58 (-2.25;-0.91) | 1.23E-05 | -1.69 (-2.37;-1.02) | 2.99E-06 | -1.69 (-2.37;-1.01) | 3.25E-06 |
| 4-Hydroxyphenylacetate | -1.62 (-2.28;-0.97) | 4.39E-06 | -1.66 (-2.32;-1.00) | 2.94E-06 | -1.65 (-2.31;-0.99) | 3.61E-06 |
| Allantoin | -1.70 (-2.35;-1.04) | 1.17E-06 | -1.64 (-2.29;-0.98) | 3.31E-06 | -1.62 (-2.28;-0.97) | 4.30E-06 |
| X-24418 | -1.59 (-2.25;-0.93) | 7.58E-06 | -1.67 (-2.34;-1.00) | 3.50E-06 | -1.66 (-2.33;-0.98) | 4.32E-06 |
| 2-Hydroxysebacate | -1.55 (-2.22;-0.89) | 1.43E-05 | -1.62 (-2.29;-0.95) | 6.65E-06 | -1.65 (-2.32;-0.98) | 4.45E-06 |
| (N(1) + N(8))-Acetylspermidine | -1.66 (-2.33;-1.00) | 2.91E-06 | -1.70 (-2.37;-1.04) | 1.80E-06 | -1.65 (-2.32;-0.98) | 4.67E-06 |
| Alpha-CEHC Sulfate | -3.09 (-4.27;-1.91) | 9.10E-07 | -2.91 (-4.11;-1.71) | 6.40E-06 | -2.96 (-4.17;-1.75) | 5.47E-06 |
| N,N-Dimethyl-5-Aminovalerate | -2.87 (-4.04;-1.69) | 5.84E-06 | -2.82 (-4.00;-1.64) | 8.79E-06 | -2.85 (-4.03;-1.67) | 7.80E-06 |
| X-23587 | -1.41 (-2.06;-0.75) | 6.89E-05 | -1.53 (-2.19;-0.87) | 1.72E-05 | -1.59 (-2.25;-0.93) | 8.12E-06 |
| 1-Methylxanthine | -1.53 (-2.18;-0.88) | 1.42E-05 | -1.58 (-2.24;-0.93) | 7.26E-06 | -1.57 (-2.23;-0.92) | 8.28E-06 |
| N-Acetyl-Aspartyl-Glutamate (NAAG) | -1.54 (-2.20;-0.88) | 1.35E-05 | -1.59 (-2.24;-0.93) | 7.76E-06 | -1.58 (-2.24;-0.92) | 8.58E-06 |
| Decanoylcarnitine (C10) | -1.57 (-2.22;-0.91) | 9.39E-06 | -1.57 (-2.24;-0.91) | 1.01E-05 | -1.58 (-2.24;-0.91) | 1.00E-05 |
| 4-Ethylphenylsulfate | -1.49 (-2.14;-0.84) | 2.26E-05 | -1.60 (-2.26;-0.93) | 7.66E-06 | -1.58 (-2.24;-0.91) | 1.03E-05 |
| N6,N6-Dimethyllysine | -1.56 (-2.21;-0.91) | 7.58E-06 | -1.56 (-2.21;-0.91) | 8.23E-06 | -1.55 (-2.20;-0.90) | 1.06E-05 |
| Dopamine 4-Sulfate | -2.92 (-4.11;-1.73) | 5.03E-06 | -2.84 (-4.04;-1.65) | 1.03E-05 | -2.86 (-4.06;-1.65) | 1.08E-05 |
| X-12729 | -1.60 (-2.25;-0.95) | 4.39E-06 | -1.55 (-2.21;-0.90) | 9.73E-06 | -1.54 (-2.19;-0.89) | 1.17E-05 |
| Octadecanedioylcarnitine (C18-DC)* | -1.64 (-2.31;-0.96) | 6.13E-06 | -1.63 (-2.31;-0.95) | 8.45E-06 | -1.60 (-2.28;-0.92) | 1.29E-05 |
| 1-Carboxyethylphenylalanine | -1.62 (-2.42;-0.83) | 1.72E-04 | -1.75 (-2.59;-0.90) | 1.42E-04 | -2.02 (-2.87;-1.16) | 1.29E-05 |
| 2-Hydroxyhippurate (Salicylurate) | -1.67 (-2.33;-1.01) | 2.16E-06 | -1.78 (-2.52;-1.03) | 9.38E-06 | -1.75 (-2.50;-1.01) | 1.30E-05 |
| X-13431 | -1.63 (-2.29;-0.97) | 3.88E-06 | -1.61 (-2.27;-0.95) | 5.77E-06 | -1.56 (-2.22;-0.89) | 1.32E-05 |
| X-12726 | -2.93 (-4.12;-1.73) | 5.17E-06 | -2.79 (-4.00;-1.59) | 1.61E-05 | -2.83 (-4.03;-1.62) | 1.44E-05 |
| X-11470 | -1.55 (-2.21;-0.90) | 1.12E-05 | -1.54 (-2.20;-0.88) | 1.36E-05 | -1.54 (-2.20;-0.88) | 1.45E-05 |
| N-Acetylglutamine | -1.53 (-2.19;-0.88) | 1.44E-05 | -1.54 (-2.20;-0.88) | 1.46E-05 | -1.54 (-2.20;-0.88) | 1.47E-05 |
| X-23639 | -1.37 (-2.01;-0.72) | 1.06E-04 | -1.55 (-2.22;-0.89) | 1.61E-05 | -1.56 (-2.23;-0.89) | 1.63E-05 |
| Umbelliferone Sulfate | -1.56 (-2.22;-0.90) | 1.17E-05 | -1.53 (-2.20;-0.87) | 1.93E-05 | -1.54 (-2.21;-0.88) | 1.70E-05 |
| 2-Methoxyhydroquinone Sulfate (1) | -1.52 (-2.18;-0.86) | 1.80E-05 | -1.51 (-2.17;-0.85) | 2.59E-05 | -1.54 (-2.20;-0.87) | 1.81E-05 |
| S-Carboxyethylcysteine | -1.23 (-1.88;-0.57) | 6.53E-04 | -1.59 (-2.30;-0.88) | 3.40E-05 | -1.63 (-2.34;-0.92) | 1.98E-05 |
| 2-Naphthol Sulfate | -0.94 (-1.60;-0.29) | 1.09E-02 | -1.94 (-2.78;-1.10) | 1.89E-05 | -1.94 (-2.78;-1.10) | 2.00E-05 |
| X-26054 | -2.50 (-3.68;-1.31) | 1.02E-04 | -2.71 (-3.89;-1.53) | 2.15E-05 | -2.72 (-3.90;-1.53) | 2.14E-05 |
| Pregnanediol-3-Glucuronide | -1.53 (-2.19;-0.88) | 1.49E-05 | -1.55 (-2.21;-0.88) | 1.75E-05 | -1.53 (-2.20;-0.86) | 2.16E-05 |
| 2-Acetamidophenol Sulfate | -1.54 (-2.20;-0.89) | 1.12E-05 | -1.53 (-2.19;-0.87) | 1.92E-05 | -1.52 (-2.18;-0.86) | 2.19E-05 |
| Nonanoylcarnitine (C9) | -1.54 (-2.20;-0.88) | 1.66E-05 | -1.53 (-2.20;-0.86) | 2.30E-05 | -1.52 (-2.19;-0.85) | 2.74E-05 |
| 4-Hydroxyphenylacetoylcarnitine | -2.71 (-3.89;-1.52) | 2.28E-05 | -2.67 (-3.85;-1.48) | 3.22E-05 | -2.71 (-3.90;-1.51) | 2.82E-05 |
| 2-Ketocaprylate | -1.40 (-2.05;-0.75) | 7.70E-05 | -1.51 (-2.17;-0.86) | 1.95E-05 | -1.49 (-2.14;-0.83) | 3.03E-05 |
| X-13866 | -1.56 (-2.21;-0.91) | 9.21E-06 | -1.47 (-2.13;-0.81) | 3.61E-05 | -1.48 (-2.14;-0.82) | 3.12E-05 |
| N-Acetylglycine | -1.30 (-1.95;-0.64) | 3.18E-04 | -1.57 (-2.26;-0.88) | 2.71E-05 | -1.56 (-2.25;-0.86) | 3.27E-05 |
| Cis-4-Decenoylcarnitine (C10:1) | -1.51 (-2.16;-0.86) | 1.76E-05 | -1.46 (-2.12;-0.81) | 3.93E-05 | -1.48 (-2.14;-0.82) | 3.27E-05 |
| X-17010 | 1.43 (0.78;2.08) | 4.53E-05 | 1.47 (0.82;2.12) | 2.82E-05 | 1.46 (0.81;2.11) | 3.32E-05 |
| Citramalate | -1.44 (-2.09;-0.79) | 3.93E-05 | -1.49 (-2.15;-0.84) | 2.61E-05 | -1.48 (-2.14;-0.82) | 3.34E-05 |
| 17alpha-Hydroxypregnanolone Glucuronide | -1.84 (-2.65;-1.02) | 3.14E-05 | -1.86 (-2.69;-1.03) | 3.40E-05 | -1.86 (-2.69;-1.03) | 3.44E-05 |
| X-21736 | -1.39 (-2.04;-0.74) | 8.89E-05 | -1.49 (-2.16;-0.82) | 3.43E-05 | -1.49 (-2.16;-0.82) | 3.58E-05 |
| 4-Hydroxycoumarin | -1.61 (-2.25;-0.96) | 3.91E-06 | -1.51 (-2.17;-0.85) | 2.29E-05 | -1.47 (-2.13;-0.81) | 3.68E-05 |
| X-21364 | -1.43 (-2.11;-0.76) | 9.47E-05 | -1.48 (-2.16;-0.79) | 6.87E-05 | -1.53 (-2.22;-0.84) | 3.76E-05 |
| Hexanoylglutamine | -1.34 (-2.01;-0.67) | 2.42E-04 | -1.46 (-2.14;-0.79) | 6.26E-05 | -1.50 (-2.17;-0.82) | 4.16E-05 |
| X-15728 | -1.38 (-2.03;-0.73) | 8.99E-05 | -1.44 (-2.10;-0.78) | 5.29E-05 | -1.46 (-2.11;-0.80) | 4.21E-05 |
| Tridecenedioate (C13:1-DC)* | -1.47 (-2.14;-0.80) | 5.15E-05 | -1.52 (-2.20;-0.84) | 3.45E-05 | -1.50 (-2.18;-0.82) | 4.34E-05 |
| Paraxanthine | -1.43 (-2.08;-0.78) | 5.03E-05 | -1.44 (-2.11;-0.78) | 6.43E-05 | -1.44 (-2.10;-0.77) | 6.83E-05 |
| X-18888 | -1.30 (-1.97;-0.62) | 4.39E-04 | -1.40 (-2.08;-0.72) | 1.59E-04 | -1.48 (-2.17;-0.80) | 6.83E-05 |
| 1-Carboxyethylisoleucine | -1.44 (-2.24;-0.64) | 1.08E-03 | -1.54 (-2.39;-0.70) | 9.16E-04 | -1.87 (-2.74;-1.00) | 6.89E-05 |
| Indole-3-Carboxylate | -1.54 (-2.19;-0.88) | 1.46E-05 | -1.47 (-2.13;-0.80) | 4.24E-05 | -1.42 (-2.09;-0.76) | 7.63E-05 |
| N-Acetyltyrosine | -1.45 (-2.11;-0.79) | 4.56E-05 | -1.48 (-2.16;-0.81) | 4.85E-05 | -1.44 (-2.11;-0.76) | 8.57E-05 |
| S-1-Pyrroline-5-Carboxylate | -2.54 (-3.75;-1.32) | 1.26E-04 | -2.61 (-3.85;-1.37) | 1.05E-04 | -2.65 (-3.90;-1.41) | 8.59E-05 |
| 5-Hydroxy-2-Methylpyridine Sulfate | -2.81 (-4.03;-1.59) | 1.89E-05 | -2.69 (-3.95;-1.42) | 8.91E-05 | -2.69 (-3.95;-1.42) | 9.15E-05 |
| X-23787 | -1.34 (-2.01;-0.68) | 1.96E-04 | -1.41 (-2.08;-0.74) | 1.05E-04 | -1.42 (-2.10;-0.75) | 9.19E-05 |
| 4-Methylguaiacol Sulfate | -1.52 (-2.17;-0.87) | 1.50E-05 | -1.41 (-2.06;-0.75) | 7.33E-05 | -1.39 (-2.04;-0.73) | 9.84E-05 |
| N-Acetyl-Beta-Alanine | -1.35 (-2.01;-0.69) | 1.67E-04 | -1.42 (-2.08;-0.75) | 9.80E-05 | -1.41 (-2.08;-0.74) | 1.02E-04 |
| 1-Palmitoyl-2-Linoleoyl-GPE (16:0/18:2) | -1.35 (-2.00;-0.70) | 1.20E-04 | -1.35 (-2.00;-0.69) | 1.59E-04 | -1.38 (-2.04;-0.72) | 1.12E-04 |
| 5-Hydroxyhexanoate | -1.37 (-2.03;-0.72) | 1.02E-04 | -1.36 (-2.01;-0.70) | 1.42E-04 | -1.36 (-2.02;-0.71) | 1.29E-04 |
| Malate | -1.32 (-1.98;-0.67) | 2.17E-04 | -1.36 (-2.03;-0.70) | 1.69E-04 | -1.39 (-2.06;-0.72) | 1.34E-04 |
| X-12127 | -2.64 (-3.83;-1.45) | 4.20E-05 | -2.52 (-3.73;-1.31) | 1.34E-04 | -2.52 (-3.74;-1.31) | 1.36E-04 |
| Choline | -1.48 (-2.14;-0.82) | 3.57E-05 | -1.40 (-2.07;-0.73) | 1.12E-04 | -1.38 (-2.05;-0.72) | 1.36E-04 |
| Indolebutyrate | -1.45 (-2.11;-0.80) | 4.14E-05 | -1.40 (-2.06;-0.74) | 8.74E-05 | -1.36 (-2.02;-0.71) | 1.39E-04 |
| Anthranilate | -1.52 (-2.18;-0.86) | 2.00E-05 | -1.44 (-2.12;-0.76) | 9.56E-05 | -1.41 (-2.09;-0.73) | 1.40E-04 |
| X-23890 | -2.81 (-4.03;-1.60) | 1.74E-05 | -2.57 (-3.80;-1.34) | 1.22E-04 | -2.55 (-3.79;-1.32) | 1.47E-04 |
| Octanoylcarnitine (C8) | -1.37 (-2.03;-0.71) | 1.35E-04 | -1.37 (-2.04;-0.71) | 1.53E-04 | -1.37 (-2.04;-0.71) | 1.48E-04 |
| X-15461 | -1.31 (-1.96;-0.65) | 2.33E-04 | -1.36 (-2.02;-0.70) | 1.56E-04 | -1.36 (-2.02;-0.70) | 1.52E-04 |
| Gamma-Glutamyltryptophan | -1.44 (-2.09;-0.78) | 5.41E-05 | -1.40 (-2.06;-0.73) | 1.17E-04 | -1.37 (-2.04;-0.70) | 1.64E-04 |
| X-24531 | -2.63 (-3.84;-1.42) | 6.07E-05 | -2.51 (-3.72;-1.30) | 1.34E-04 | -2.50 (-3.72;-1.28) | 1.64E-04 |
| X-11850 | -1.52 (-2.17;-0.87) | 1.65E-05 | -1.41 (-2.07;-0.74) | 9.46E-05 | -1.36 (-2.03;-0.69) | 1.72E-04 |
| X-12844 | -1.33 (-1.98;-0.67) | 1.97E-04 | -1.37 (-2.03;-0.71) | 1.47E-04 | -1.35 (-2.02;-0.69) | 1.88E-04 |
| Octadecenedioylcarnitine (C18:1-DC)* | -1.35 (-2.02;-0.68) | 2.33E-04 | -1.38 (-2.05;-0.70) | 1.79E-04 | -1.37 (-2.05;-0.70) | 1.93E-04 |
| Alpha-Ketobutyrate | 1.27 (0.62;1.93) | 3.40E-04 | 1.39 (0.70;2.07) | 1.89E-04 | 1.40 (0.71;2.08) | 1.93E-04 |
| X-26119 | -1.43 (-2.10;-0.77) | 6.42E-05 | -1.35 (-2.02;-0.68) | 1.94E-04 | -1.34 (-2.01;-0.67) | 2.21E-04 |
| 1-Carboxyethylvaline | -1.37 (-2.16;-0.58) | 1.60E-03 | -1.39 (-2.21;-0.57) | 2.20E-03 | -1.68 (-2.51;-0.84) | 2.36E-04 |
| P-Cresol Glucuronide* | -1.46 (-2.12;-0.81) | 3.65E-05 | -1.36 (-2.02;-0.69) | 1.70E-04 | -1.33 (-2.00;-0.67) | 2.41E-04 |
| 4-Methylcatechol Sulfate | -1.44 (-2.09;-0.79) | 3.92E-05 | -1.33 (-1.98;-0.68) | 1.74E-04 | -1.30 (-1.95;-0.65) | 2.51E-04 |
| Glucuronide Of C10H18O2 (7)* | -1.44 (-2.09;-0.79) | 4.22E-05 | -1.32 (-1.97;-0.66) | 2.32E-04 | -1.31 (-1.96;-0.65) | 2.69E-04 |
| Laurylcarnitine (C12) | -1.28 (-1.95;-0.61) | 4.59E-04 | -1.34 (-2.01;-0.66) | 2.78E-04 | -1.33 (-2.01;-0.66) | 2.82E-04 |
| Dihydrocaffeate Sulfate (2) | -2.43 (-3.64;-1.23) | 2.07E-04 | -2.35 (-3.56;-1.14) | 3.74E-04 | -2.41 (-3.62;-1.19) | 2.82E-04 |
| Succinate | -1.32 (-1.97;-0.67) | 1.97E-04 | -1.33 (-1.99;-0.67) | 2.04E-04 | -1.30 (-1.96;-0.64) | 2.99E-04 |
| 2-Aminobutyrate | 1.25 (0.60;1.90) | 4.59E-04 | 1.28 (0.62;1.94) | 3.81E-04 | 1.30 (0.63;1.96) | 3.34E-04 |
| N6-Methyladenosine | -1.31 (-1.96;-0.66) | 2.04E-04 | -1.29 (-1.94;-0.64) | 2.75E-04 | -1.27 (-1.93;-0.62) | 3.37E-04 |
| 5alpha-Androstan-3beta,17alpha-Diol Disulfate | -1.42 (-2.11;-0.73) | 1.60E-04 | -1.35 (-2.05;-0.65) | 4.32E-04 | -1.37 (-2.08;-0.67) | 3.43E-04 |
| Indoleacetate | -1.40 (-2.05;-0.74) | 8.34E-05 | -1.34 (-2.00;-0.67) | 2.03E-04 | -1.29 (-1.95;-0.63) | 3.69E-04 |
| X-11843 | -1.47 (-2.13;-0.82) | 3.15E-05 | -1.34 (-2.00;-0.67) | 2.25E-04 | -1.28 (-1.95;-0.62) | 4.34E-04 |
| X-18935 | -2.36 (-3.56;-1.16) | 2.96E-04 | -2.36 (-3.58;-1.13) | 4.34E-04 | -2.35 (-3.58;-1.12) | 4.70E-04 |
| 1-Carboxyethyltyrosine | -1.29 (-2.08;-0.50) | 3.51E-03 | -1.35 (-2.19;-0.52) | 3.64E-03 | -1.63 (-2.48;-0.78) | 4.77E-04 |
| X-24544 | -1.32 (-2.02;-0.63) | 4.95E-04 | -1.33 (-2.02;-0.63) | 5.16E-04 | -1.33 (-2.02;-0.63) | 5.14E-04 |
| N-Acetylarginine | -1.32 (-1.97;-0.67) | 1.99E-04 | -1.26 (-1.91;-0.60) | 4.69E-04 | -1.24 (-1.90;-0.59) | 5.41E-04 |
| Homostachydrine* | -1.18 (-1.83;-0.53) | 9.82E-04 | -1.27 (-1.94;-0.61) | 4.77E-04 | -1.25 (-1.92;-0.59) | 5.82E-04 |
| 2-Oxoarginine* | -1.33 (-1.99;-0.67) | 2.19E-04 | -1.27 (-1.95;-0.59) | 6.87E-04 | -1.28 (-1.97;-0.60) | 6.05E-04 |
| Taurolithocholate 3-Sulfate | -1.21 (-1.86;-0.56) | 7.04E-04 | -1.22 (-1.88;-0.57) | 6.76E-04 | -1.23 (-1.89;-0.58) | 6.06E-04 |
| Tyramine O-Sulfate | -1.10 (-1.76;-0.44) | 2.74E-03 | -1.22 (-1.89;-0.56) | 8.40E-04 | -1.25 (-1.92;-0.59) | 6.19E-04 |
| X-18345 | -2.36 (-3.56;-1.16) | 3.27E-04 | -2.26 (-3.47;-1.04) | 6.95E-04 | -2.28 (-3.50;-1.06) | 6.20E-04 |
| 5-Dodecenoylcarnitine (C12:1) | -1.20 (-1.87;-0.53) | 1.16E-03 | -1.29 (-1.97;-0.61) | 5.38E-04 | -1.27 (-1.95;-0.59) | 6.68E-04 |
| X-24411 | -2.26 (-3.46;-1.06) | 5.72E-04 | -2.26 (-3.47;-1.05) | 6.60E-04 | -2.25 (-3.46;-1.04) | 6.96E-04 |
| Epiandrosterone Sulfate | -1.24 (-1.94;-0.54) | 1.30E-03 | -1.29 (-2.00;-0.57) | 1.02E-03 | -1.32 (-2.04;-0.61) | 7.00E-04 |
| X-12111 | -1.30 (-1.95;-0.65) | 2.44E-04 | -1.24 (-1.90;-0.57) | 6.70E-04 | -1.23 (-1.89;-0.57) | 7.13E-04 |
| X-25420 | -1.18 (-1.87;-0.49) | 1.91E-03 | -1.35 (-2.06;-0.64) | 4.95E-04 | -1.32 (-2.03;-0.61) | 7.13E-04 |
| Dihydroferulic Acid Sulfate | -2.28 (-3.49;-1.06) | 6.21E-04 | -2.22 (-3.45;-1.00) | 9.55E-04 | -2.27 (-3.50;-1.04) | 7.65E-04 |
| X-12816 | -1.19 (-1.84;-0.53) | 9.27E-04 | -1.22 (-1.87;-0.56) | 7.63E-04 | -1.21 (-1.86;-0.55) | 8.41E-04 |
| Ferulic Acid 4-Sulfate | -2.38 (-3.60;-1.16) | 3.68E-04 | -2.23 (-3.46;-1.00) | 9.76E-04 | -2.27 (-3.50;-1.03) | 8.47E-04 |
| Beta-Alanine | -1.26 (-1.91;-0.61) | 4.26E-04 | -1.23 (-1.88;-0.57) | 6.76E-04 | -1.20 (-1.86;-0.54) | 8.72E-04 |
| Catechol Glucuronide | -2.69 (-3.90;-1.48) | 4.02E-05 | -2.28 (-3.53;-1.03) | 9.10E-04 | -2.29 (-3.55;-1.04) | 8.73E-04 |
| X-24473 | -1.10 (-1.75;-0.44) | 2.45E-03 | -1.22 (-1.89;-0.56) | 8.49E-04 | -1.20 (-1.87;-0.53) | 1.08E-03 |
| 3b-Hydroxy-5-Cholenoic Acid | 1.22 (0.57;1.87) | 6.21E-04 | 1.16 (0.50;1.83) | 1.47E-03 | 1.19 (0.52;1.85) | 1.19E-03 |
| Ribitol | -1.19 (-1.84;-0.54) | 8.34E-04 | -1.15 (-1.81;-0.50) | 1.38E-03 | -1.16 (-1.82;-0.51) | 1.31E-03 |
| Propionylcarnitine (C3) | -1.26 (-1.92;-0.60) | 4.67E-04 | -1.18 (-1.85;-0.51) | 1.41E-03 | -1.19 (-1.86;-0.52) | 1.35E-03 |
| X-26058 | -2.44 (-3.63;-1.26) | 1.52E-04 | -2.13 (-3.33;-0.93) | 1.33E-03 | -2.14 (-3.35;-0.93) | 1.38E-03 |
| X-25520 | -1.19 (-1.84;-0.53) | 9.13E-04 | -1.13 (-1.79;-0.48) | 1.82E-03 | -1.16 (-1.81;-0.50) | 1.39E-03 |
| 1-Palmitoyl-2-Docosahexaenoyl-GPE (16:0/22:6)* | -1.22 (-1.88;-0.56) | 7.64E-04 | -1.16 (-1.85;-0.47) | 2.43E-03 | -1.21 (-1.91;-0.52) | 1.45E-03 |
| X-13684 | -1.39 (-2.19;-0.59) | 1.64E-03 | -1.40 (-2.21;-0.58) | 2.06E-03 | -1.43 (-2.25;-0.61) | 1.52E-03 |
| X-12126 | -2.21 (-3.40;-1.01) | 7.69E-04 | -2.12 (-3.34;-0.90) | 1.67E-03 | -2.13 (-3.36;-0.90) | 1.65E-03 |
| X-12306 | -1.17 (-1.82;-0.52) | 1.08E-03 | -1.13 (-1.79;-0.47) | 2.05E-03 | -1.13 (-1.79;-0.47) | 2.03E-03 |
| N,N-Dimethylalanine | -1.27 (-1.92;-0.62) | 3.41E-04 | -1.19 (-1.85;-0.54) | 9.26E-04 | -1.13 (-1.78;-0.47) | 2.04E-03 |
| 2'-O-Methylcytidine | -1.05 (-1.71;-0.40) | 3.69E-03 | -1.13 (-1.79;-0.47) | 2.06E-03 | -1.13 (-1.79;-0.47) | 2.06E-03 |
| Glycerol 3-Phosphate | -1.19 (-1.84;-0.54) | 8.56E-04 | -1.08 (-1.73;-0.42) | 3.19E-03 | -1.10 (-1.76;-0.44) | 2.57E-03 |
| 4-Allylphenol Sulfate | -1.16 (-1.81;-0.51) | 1.14E-03 | -1.09 (-1.75;-0.43) | 3.19E-03 | -1.10 (-1.76;-0.44) | 2.79E-03 |
| Tartronate (Hydroxymalonate) | 0.96 (0.29;1.62) | 1.07E-02 | 1.09 (0.40;1.77) | 4.39E-03 | 1.14 (0.45;1.82) | 2.96E-03 |
| Fumarate | -1.07 (-1.72;-0.42) | 3.05E-03 | -1.07 (-1.73;-0.42) | 3.32E-03 | -1.07 (-1.72;-0.41) | 3.51E-03 |
| X-11852 | -1.00 (-1.65;-0.34) | 6.62E-03 | -1.05 (-1.71;-0.39) | 4.26E-03 | -1.07 (-1.73;-0.41) | 3.54E-03 |
| Theophylline | -1.05 (-1.70;-0.40) | 3.78E-03 | -1.10 (-1.77;-0.43) | 3.33E-03 | -1.09 (-1.76;-0.42) | 3.57E-03 |
| Dimethyl Sulfone | -1.86 (-3.05;-0.66) | 5.51E-03 | -1.95 (-3.13;-0.76) | 3.29E-03 | -1.94 (-3.13;-0.74) | 3.62E-03 |
| Carboxyethyl-GABA | -1.12 (-1.77;-0.47) | 1.84E-03 | -1.08 (-1.74;-0.43) | 2.96E-03 | -1.06 (-1.72;-0.40) | 3.75E-03 |
| Gamma-Glutamylhistidine | -1.88 (-3.08;-0.68) | 5.11E-03 | -1.95 (-3.15;-0.74) | 3.87E-03 | -1.96 (-3.17;-0.75) | 3.75E-03 |
| Ursodeoxycholate | 1.07 (0.41;1.72) | 3.42E-03 | 1.05 (0.39;1.71) | 4.58E-03 | 1.07 (0.41;1.73) | 3.79E-03 |
| 1-Stearoyl-2-Docosahexaenoyl-GPE (18:0/22:6)* | -1.11 (-1.76;-0.45) | 2.45E-03 | -1.09 (-1.80;-0.37) | 6.54E-03 | -1.16 (-1.87;-0.44) | 3.87E-03 |
| Sebacate (C10-DC) | -0.95 (-1.62;-0.28) | 1.24E-02 | -1.05 (-1.73;-0.38) | 5.43E-03 | -1.09 (-1.76;-0.41) | 4.01E-03 |
| Phenol Glucuronide | -1.99 (-3.18;-0.79) | 2.88E-03 | -1.95 (-3.14;-0.76) | 3.34E-03 | -1.93 (-3.13;-0.73) | 4.01E-03 |
| Pro-Hydroxy-Pro | -1.00 (-1.66;-0.35) | 6.43E-03 | -1.08 (-1.74;-0.42) | 3.29E-03 | -1.06 (-1.73;-0.40) | 4.08E-03 |
| Dodecenedioate (C12:1-DC)* | -0.94 (-1.60;-0.27) | 1.26E-02 | -1.02 (-1.69;-0.35) | 6.74E-03 | -1.08 (-1.75;-0.40) | 4.21E-03 |
| X-26107 | -1.05 (-1.73;-0.37) | 5.87E-03 | -1.06 (-1.75;-0.37) | 5.82E-03 | -1.09 (-1.78;-0.40) | 4.49E-03 |
| Saccharin | -1.16 (-1.94;-0.39) | 7.21E-03 | -1.22 (-2.00;-0.45) | 4.64E-03 | -1.22 (-1.99;-0.45) | 4.65E-03 |
| 12-Hete | 1.06 (0.40;1.71) | 3.65E-03 | 1.09 (0.43;1.74) | 2.94E-03 | 1.04 (0.38;1.69) | 4.70E-03 |
| 3-Carboxy-4-Methyl-5-Propyl-2-Furanpropanoate (CMPF) | -1.07 (-1.72;-0.42) | 3.01E-03 | -1.06 (-1.73;-0.40) | 3.91E-03 | -1.04 (-1.71;-0.38) | 4.74E-03 |
| Sulfate Of Piperine Metabolite C16H19NO3 (3)* | -1.05 (-1.71;-0.39) | 4.56E-03 | -1.09 (-1.77;-0.40) | 4.37E-03 | -1.07 (-1.75;-0.39) | 4.87E-03 |
| Tetradecadienedioate (C14:2-DC)* | -0.89 (-1.55;-0.23) | 1.80E-02 | -1.04 (-1.70;-0.37) | 5.33E-03 | -1.05 (-1.71;-0.38) | 4.87E-03 |
| Eugenol Sulfate | -1.03 (-1.69;-0.37) | 5.50E-03 | -1.04 (-1.70;-0.37) | 5.28E-03 | -1.04 (-1.70;-0.38) | 5.03E-03 |
| N,N,N-Trimethyl-5-Aminovalerate | -1.09 (-1.75;-0.44) | 2.45E-03 | -1.01 (-1.68;-0.35) | 6.66E-03 | -1.05 (-1.72;-0.38) | 5.24E-03 |
| Gentisate | -1.85 (-3.06;-0.65) | 5.87E-03 | -1.89 (-3.10;-0.68) | 5.20E-03 | -1.89 (-3.10;-0.68) | 5.29E-03 |
| Retinal | -1.07 (-1.72;-0.42) | 3.14E-03 | -1.02 (-1.68;-0.37) | 5.38E-03 | -1.02 (-1.67;-0.36) | 5.41E-03 |
| X-07765 | -1.03 (-1.68;-0.38) | 4.43E-03 | -1.03 (-1.70;-0.37) | 5.51E-03 | -1.03 (-1.70;-0.37) | 5.49E-03 |
| X-18899 | 0.91 (0.25;1.57) | 1.47E-02 | 1.01 (0.34;1.67) | 6.77E-03 | 1.03 (0.37;1.70) | 5.52E-03 |
| 3-Hydroxyhexanoate | -0.92 (-1.57;-0.26) | 1.39E-02 | -1.02 (-1.68;-0.36) | 6.14E-03 | -1.03 (-1.69;-0.36) | 5.89E-03 |
| Glycochenodeoxycholate Glucuronide (1) | 1.09 (0.40;1.78) | 4.59E-03 | 1.07 (0.37;1.77) | 6.15E-03 | 1.08 (0.38;1.77) | 5.92E-03 |
| Acetylcarnitine (C2) | -0.95 (-1.60;-0.30) | 9.79E-03 | -1.01 (-1.67;-0.35) | 6.34E-03 | -1.01 (-1.67;-0.35) | 6.18E-03 |
| X-25271 | -1.07 (-1.73;-0.41) | 3.42E-03 | -1.03 (-1.70;-0.37) | 5.40E-03 | -1.01 (-1.68;-0.35) | 6.56E-03 |
| Taurocholenate Sulfate* | -0.96 (-1.61;-0.31) | 9.16E-03 | -1.02 (-1.68;-0.35) | 6.39E-03 | -1.01 (-1.67;-0.34) | 7.03E-03 |
| Androsterone Sulfate | -0.99 (-1.67;-0.32) | 8.97E-03 | -0.99 (-1.67;-0.31) | 9.60E-03 | -1.03 (-1.70;-0.35) | 7.20E-03 |
| X-21834 | -0.96 (-1.61;-0.31) | 8.90E-03 | -0.98 (-1.63;-0.32) | 7.65E-03 | -0.99 (-1.64;-0.33) | 7.20E-03 |
| X-16938 | 1.17 (0.40;1.95) | 7.03E-03 | 1.20 (0.42;1.97) | 5.82E-03 | 1.17 (0.39;1.94) | 7.33E-03 |
| Isovalerylcarnitine (C5) | -1.11 (-1.78;-0.45) | 2.61E-03 | -1.06 (-1.74;-0.38) | 5.20E-03 | -1.03 (-1.71;-0.34) | 7.41E-03 |
| Aspartate | 0.83 (0.17;1.50) | 2.92E-02 | 1.01 (0.31;1.70) | 9.99E-03 | 1.05 (0.35;1.75) | 7.44E-03 |
| Glycerophosphorylcholine (GPC) | 0.81 (0.16;1.47) | 3.04E-02 | 1.02 (0.32;1.72) | 9.79E-03 | 1.05 (0.35;1.76) | 7.50E-03 |
| Caffeine | -0.99 (-1.65;-0.34) | 6.70E-03 | -1.01 (-1.70;-0.32) | 9.40E-03 | -1.04 (-1.73;-0.34) | 7.63E-03 |
| 6-Oxopiperidine-2-Carboxylate | -1.05 (-1.70;-0.40) | 3.85E-03 | -1.00 (-1.66;-0.33) | 7.74E-03 | -0.99 (-1.66;-0.32) | 8.26E-03 |
| Cysteinylglycine | -1.01 (-1.66;-0.35) | 6.21E-03 | -1.01 (-1.67;-0.35) | 6.02E-03 | -0.98 (-1.63;-0.32) | 8.27E-03 |
| 2-Aminoheptanoate | -1.00 (-1.66;-0.34) | 6.66E-03 | -0.99 (-1.66;-0.32) | 8.37E-03 | -0.99 (-1.66;-0.32) | 8.32E-03 |
| 1-Carboxyethylleucine | -1.03 (-1.83;-0.23) | 2.38E-02 | -1.06 (-1.90;-0.23) | 2.61E-02 | -1.25 (-2.09;-0.41) | 8.36E-03 |
| Citrate | -0.99 (-1.64;-0.33) | 7.43E-03 | -0.99 (-1.65;-0.33) | 7.79E-03 | -0.98 (-1.65;-0.32) | 8.39E-03 |
| 1-Stearoyl-2-Linoleoyl-GPE (18:0/18:2)* | -1.02 (-1.67;-0.37) | 5.12E-03 | -0.96 (-1.63;-0.30) | 1.04E-02 | -0.99 (-1.66;-0.32) | 8.76E-03 |
| Butyrylglycine | -0.94 (-1.59;-0.28) | 1.13E-02 | -0.99 (-1.65;-0.34) | 6.94E-03 | -0.97 (-1.62;-0.31) | 9.13E-03 |
| (16 Or 17)-Methylstearate (A19:0 Or I19:0) | 1.02 (0.35;1.69) | 6.87E-03 | 1.03 (0.35;1.71) | 7.01E-03 | 1.00 (0.32;1.68) | 9.14E-03 |
| 1-Palmitoyl-2-Arachidonoyl-GPE (16:0/20:4)* | -0.99 (-1.64;-0.34) | 6.73E-03 | -0.95 (-1.62;-0.27) | 1.28E-02 | -0.99 (-1.66;-0.31) | 9.15E-03 |
| Theobromine | -1.01 (-1.66;-0.36) | 5.42E-03 | -0.96 (-1.61;-0.30) | 9.77E-03 | -0.96 (-1.62;-0.30) | 9.35E-03 |
| Docosadienoate (22:2n6) | 1.05 (0.39;1.70) | 4.06E-03 | 1.03 (0.36;1.69) | 5.85E-03 | 0.97 (0.31;1.64) | 9.46E-03 |
| N-Acetylglutamate | -0.95 (-1.60;-0.30) | 9.64E-03 | -0.94 (-1.60;-0.28) | 1.15E-02 | -0.96 (-1.62;-0.30) | 9.66E-03 |
| Xanthine | -1.08 (-1.73;-0.43) | 2.81E-03 | -1.00 (-1.66;-0.34) | 6.64E-03 | -0.96 (-1.62;-0.30) | 9.74E-03 |
| Nonadecanoate (19:0) | 0.96 (0.30;1.62) | 9.58E-03 | 1.01 (0.34;1.68) | 6.94E-03 | 0.97 (0.30;1.64) | 9.89E-03 |
| 1-Palmitoyl-2-Oleoyl-GPE (16:0/18:1) | -0.99 (-1.64;-0.34) | 6.37E-03 | -0.96 (-1.64;-0.28) | 1.20E-02 | -0.98 (-1.66;-0.30) | 1.01E-02 |
| Sulfate Of Piperine Metabolite C16H19NO3 (2)* | -0.96 (-1.62;-0.30) | 9.93E-03 | -0.99 (-1.67;-0.31) | 9.82E-03 | -0.98 (-1.66;-0.30) | 1.06E-02 |
| 4-Methoxyphenol Sulfate | -0.87 (-1.52;-0.22) | 1.85E-02 | -0.90 (-1.55;-0.25) | 1.53E-02 | -0.93 (-1.59;-0.28) | 1.16E-02 |
| Indolepropionate | -1.02 (-1.67;-0.37) | 5.19E-03 | -1.00 (-1.67;-0.33) | 7.91E-03 | -0.96 (-1.63;-0.28) | 1.21E-02 |
| Eicosenoate (20:1) | 1.02 (0.36;1.67) | 5.60E-03 | 0.99 (0.33;1.66) | 7.75E-03 | 0.94 (0.28;1.61) | 1.23E-02 |
| 2'-Deoxyuridine | 0.86 (0.21;1.52) | 2.10E-02 | 0.88 (0.22;1.54) | 2.00E-02 | 0.94 (0.28;1.61) | 1.23E-02 |
| X-23780 | -0.89 (-1.55;-0.24) | 1.63E-02 | -0.93 (-1.59;-0.27) | 1.23E-02 | -0.93 (-1.59;-0.27) | 1.23E-02 |
| 1-Arachidonoyl-GPC (20:4n6)* | 0.74 (0.09;1.39) | 5.07E-02 | 0.94 (0.26;1.62) | 1.47E-02 | 0.95 (0.27;1.63) | 1.34E-02 |
| Histidine | 0.88 (0.22;1.53) | 1.84E-02 | 0.89 (0.23;1.56) | 1.77E-02 | 0.92 (0.26;1.59) | 1.41E-02 |
| Myristoleoylcarnitine (C14:1)* | -0.88 (-1.54;-0.22) | 2.00E-02 | -0.94 (-1.60;-0.27) | 1.31E-02 | -0.93 (-1.59;-0.26) | 1.43E-02 |
| 1,2,3-Benzenetriol Sulfate (2) | -1.58 (-2.78;-0.38) | 2.06E-02 | -1.66 (-2.85;-0.46) | 1.46E-02 | -1.67 (-2.87;-0.47) | 1.43E-02 |
| 5alpha-Pregnan-3beta,20alpha-Diol Disulfate | -0.98 (-1.65;-0.30) | 1.03E-02 | -0.91 (-1.59;-0.24) | 1.77E-02 | -0.94 (-1.62;-0.26) | 1.43E-02 |
| Trans-2-Hexenoylglycine | -1.48 (-2.74;-0.21) | 4.28E-02 | -1.78 (-3.07;-0.48) | 1.58E-02 | -1.81 (-3.11;-0.50) | 1.43E-02 |
| 4-Hydroxychlorothalonil | 0.74 (0.08;1.41) | 5.38E-02 | 0.87 (0.19;1.54) | 2.52E-02 | 0.94 (0.26;1.62) | 1.47E-02 |
| X-23997 | -1.70 (-2.91;-0.50) | 1.26E-02 | -1.68 (-2.90;-0.47) | 1.42E-02 | -1.67 (-2.88;-0.45) | 1.56E-02 |
| Cysteine Sulfinic Acid | -0.83 (-1.48;-0.18) | 2.55E-02 | -0.94 (-1.61;-0.26) | 1.42E-02 | -0.92 (-1.59;-0.25) | 1.61E-02 |
| Pantothenate | -0.94 (-1.59;-0.29) | 1.09E-02 | -0.94 (-1.60;-0.28) | 1.18E-02 | -0.91 (-1.57;-0.24) | 1.62E-02 |
| 4-Allylcatechol Sulfate | -0.85 (-1.50;-0.19) | 2.32E-02 | -0.86 (-1.51;-0.20) | 2.21E-02 | -0.90 (-1.55;-0.24) | 1.63E-02 |
| X-12812 | -1.04 (-1.70;-0.39) | 4.06E-03 | -0.94 (-1.60;-0.27) | 1.22E-02 | -0.90 (-1.56;-0.24) | 1.63E-02 |
| Isoursodeoxycholate | 0.91 (0.26;1.56) | 1.27E-02 | 0.91 (0.25;1.56) | 1.47E-02 | 0.89 (0.23;1.55) | 1.67E-02 |
| Stearidonate (18:4n3) | 0.93 (0.25;1.61) | 1.59E-02 | 0.97 (0.28;1.66) | 1.29E-02 | 0.94 (0.25;1.63) | 1.68E-02 |
| Methyl Glucopyranoside (Alpha + Beta) | -0.94 (-1.60;-0.28) | 1.14E-02 | -0.96 (-1.63;-0.29) | 1.16E-02 | -0.92 (-1.60;-0.24) | 1.68E-02 |
| Taurochenodeoxycholic Acid 3-Sulfate | -0.89 (-1.54;-0.23) | 1.73E-02 | -0.94 (-1.61;-0.28) | 1.21E-02 | -0.90 (-1.57;-0.24) | 1.69E-02 |
| 2-Hydroxyfluorene Sulfate | -0.85 (-2.06;0.37) | 2.58E-01 | -2.16 (-3.77;-0.54) | 1.93E-02 | -2.19 (-3.81;-0.57) | 1.72E-02 |
| 5alpha-Pregnan-3beta,20alpha-Diol Monosulfate (2) | -0.94 (-1.60;-0.28) | 1.18E-02 | -0.87 (-1.54;-0.21) | 2.11E-02 | -0.88 (-1.55;-0.22) | 1.95E-02 |
| 1-Stearoyl-2-Oleoyl-GPE (18:0/18:1) | -0.96 (-1.60;-0.31) | 8.91E-03 | -0.87 (-1.55;-0.19) | 2.49E-02 | -0.91 (-1.59;-0.22) | 2.01E-02 |
| Glycoursodeoxycholate | 0.88 (0.23;1.53) | 1.75E-02 | 0.87 (0.21;1.52) | 2.04E-02 | 0.87 (0.21;1.52) | 2.05E-02 |
| 3-Methyl-2-Oxobutyrate | 0.81 (0.16;1.47) | 3.08E-02 | 0.86 (0.19;1.53) | 2.44E-02 | 0.87 (0.20;1.55) | 2.28E-02 |
| Suberate (C8-DC) | -1.64 (-2.86;-0.43) | 1.74E-02 | -1.60 (-2.82;-0.37) | 2.23E-02 | -1.61 (-2.85;-0.37) | 2.30E-02 |
| X-21842 | -0.91 (-1.56;-0.26) | 1.39E-02 | -0.87 (-1.54;-0.21) | 2.06E-02 | -0.86 (-1.52;-0.20) | 2.31E-02 |
| 3-Hydroxyoctanoate | -0.71 (-1.37;-0.04) | 7.00E-02 | -0.80 (-1.48;-0.12) | 4.12E-02 | -0.88 (-1.57;-0.20) | 2.39E-02 |
| Glcnac Sulfate Conjugate Of C21H34O2 Steroid** | 1.67 (0.41;2.93) | 2.02E-02 | 1.65 (0.38;2.92) | 2.31E-02 | 1.64 (0.37;2.92) | 2.40E-02 |
| Trans-4-Hydroxyproline | -0.79 (-1.44;-0.13) | 3.81E-02 | -0.83 (-1.50;-0.17) | 2.94E-02 | -0.85 (-1.52;-0.19) | 2.53E-02 |
| 5-Methyluridine (Ribothymidine) | -0.93 (-1.59;-0.28) | 1.17E-02 | -0.88 (-1.54;-0.21) | 2.06E-02 | -0.85 (-1.51;-0.18) | 2.61E-02 |
| X-16124 | -1.56 (-2.77;-0.34) | 2.49E-02 | -1.56 (-2.78;-0.34) | 2.48E-02 | -1.55 (-2.77;-0.33) | 2.64E-02 |
| Glycine | -0.79 (-1.46;-0.13) | 3.83E-02 | -0.90 (-1.60;-0.21) | 2.23E-02 | -0.88 (-1.58;-0.18) | 2.75E-02 |
| Phenylalanine | -0.94 (-1.60;-0.28) | 1.13E-02 | -0.88 (-1.54;-0.21) | 2.06E-02 | -0.84 (-1.50;-0.17) | 2.83E-02 |
| Cysteine S-Sulfate | -1.68 (-2.88;-0.49) | 1.27E-02 | -1.54 (-2.75;-0.33) | 2.59E-02 | -1.52 (-2.73;-0.31) | 2.83E-02 |
| X-24475 | -0.87 (-1.54;-0.21) | 2.17E-02 | -0.88 (-1.55;-0.20) | 2.23E-02 | -0.85 (-1.53;-0.17) | 2.84E-02 |
| 3-Phenylpropionate (Hydrocinnamate) | -0.90 (-1.55;-0.25) | 1.44E-02 | -0.85 (-1.51;-0.19) | 2.48E-02 | -0.83 (-1.49;-0.17) | 2.87E-02 |
| N2-Acetyl,N6,N6-Dimethyllysine | -0.79 (-1.44;-0.14) | 3.39E-02 | -0.82 (-1.47;-0.16) | 2.92E-02 | -0.82 (-1.47;-0.17) | 2.87E-02 |
| Lithocholate | 1.42 (0.22;2.63) | 3.99E-02 | 1.52 (0.30;2.73) | 2.94E-02 | 1.53 (0.31;2.74) | 2.87E-02 |
| Deoxycholate | 0.79 (0.13;1.44) | 3.57E-02 | 0.83 (0.16;1.49) | 2.97E-02 | 0.84 (0.17;1.50) | 2.88E-02 |
| Glycerophosphoethanolamine | 0.69 (0.04;1.34) | 7.10E-02 | 0.84 (0.15;1.52) | 3.29E-02 | 0.86 (0.17;1.54) | 2.88E-02 |
| Cis-3,4-Methyleneheptanoate | -0.82 (-1.47;-0.17) | 2.73E-02 | -0.86 (-1.52;-0.20) | 2.15E-02 | -0.83 (-1.48;-0.17) | 2.89E-02 |
| Androstenediol (3beta,17beta) Disulfate (2) | -0.84 (-1.57;-0.10) | 5.09E-02 | -0.88 (-1.63;-0.14) | 4.05E-02 | -0.93 (-1.68;-0.18) | 2.98E-02 |
| Bilirubin Degradation Product, C16H18N2O5 (3)** | 1.55 (0.33;2.76) | 2.64E-02 | 1.55 (0.32;2.79) | 2.84E-02 | 1.56 (0.31;2.80) | 3.00E-02 |
| X-21310 | -0.82 (-1.47;-0.16) | 2.86E-02 | -0.83 (-1.49;-0.17) | 2.76E-02 | -0.82 (-1.48;-0.16) | 3.00E-02 |
| N-Methylhydroxyproline** | -0.92 (-1.57;-0.27) | 1.21E-02 | -0.81 (-1.47;-0.15) | 3.14E-02 | -0.82 (-1.47;-0.16) | 3.12E-02 |
| Dihomo-Linoleate (20:2n6) | 0.88 (0.22;1.54) | 1.84E-02 | 0.88 (0.22;1.55) | 2.03E-02 | 0.82 (0.16;1.49) | 3.15E-02 |
| Corticosterone | -0.75 (-1.41;-0.10) | 4.57E-02 | -0.85 (-1.52;-0.19) | 2.47E-02 | -0.82 (-1.49;-0.16) | 3.17E-02 |
| Glutamate | 0.65 (-0.01;1.31) | 9.64E-02 | 0.87 (0.16;1.58) | 3.31E-02 | 0.88 (0.17;1.59) | 3.17E-02 |
| Caprate (10:0) | 0.95 (0.27;1.62) | 1.33E-02 | 0.87 (0.18;1.56) | 2.85E-02 | 0.85 (0.16;1.54) | 3.21E-02 |
| Gamma-Glutamylthreonine | -0.84 (-1.49;-0.18) | 2.46E-02 | -0.84 (-1.49;-0.18) | 2.56E-02 | -0.81 (-1.46;-0.15) | 3.22E-02 |
| Bilirubin Degradation Product, C16H18N2O5 (2)** | 1.56 (0.35;2.77) | 2.40E-02 | 1.52 (0.30;2.75) | 3.03E-02 | 1.52 (0.28;2.76) | 3.24E-02 |
| Isoeugenol Sulfate | -1.34 (-2.55;-0.12) | 5.76E-02 | -1.46 (-2.69;-0.23) | 3.87E-02 | -1.52 (-2.76;-0.28) | 3.26E-02 |
| X-25172 | 0.79 (0.13;1.44) | 3.64E-02 | 0.77 (0.11;1.42) | 4.41E-02 | 0.81 (0.15;1.46) | 3.33E-02 |
| X-12740 | -0.83 (-1.48;-0.17) | 2.65E-02 | -0.81 (-1.46;-0.15) | 3.14E-02 | -0.80 (-1.45;-0.14) | 3.41E-02 |
| Bilirubin Degradation Product, C16H18N2O5 (4)** | 1.56 (0.34;2.77) | 2.46E-02 | 1.52 (0.28;2.75) | 3.21E-02 | 1.51 (0.27;2.76) | 3.47E-02 |
| Glycoursodeoxycholic Acid Sulfate (1) | 0.82 (0.15;1.49) | 3.16E-02 | 0.83 (0.15;1.51) | 3.26E-02 | 0.82 (0.14;1.50) | 3.53E-02 |
| X-23636 | 0.76 (0.10;1.41) | 4.53E-02 | 0.76 (0.11;1.42) | 4.42E-02 | 0.79 (0.14;1.45) | 3.60E-02 |
| Ethyl Beta-Glucopyranoside | -0.76 (-1.41;-0.11) | 4.35E-02 | -0.81 (-1.48;-0.14) | 3.44E-02 | -0.81 (-1.48;-0.14) | 3.62E-02 |
| N-Formylanthranilic Acid | -1.51 (-2.71;-0.31) | 2.73E-02 | -1.48 (-2.69;-0.27) | 3.26E-02 | -1.45 (-2.67;-0.24) | 3.74E-02 |
| Bilirubin Degradation Product, C16H18N2O5 (1)** | 1.48 (0.27;2.69) | 3.34E-02 | 1.49 (0.27;2.71) | 3.29E-02 | 1.48 (0.24;2.71) | 3.77E-02 |
| N-Oleoyltaurine | -0.72 (-1.37;-0.07) | 5.69E-02 | -0.74 (-1.39;-0.09) | 5.12E-02 | -0.78 (-1.44;-0.13) | 3.77E-02 |
| X-25828 | 0.77 (-0.05;1.60) | 1.15E-01 | 0.84 (-0.03;1.70) | 9.97E-02 | 1.05 (0.17;1.93) | 3.77E-02 |
| Myristoylcarnitine (C14) | -0.82 (-1.50;-0.15) | 3.34E-02 | -0.84 (-1.52;-0.16) | 3.05E-02 | -0.81 (-1.49;-0.13) | 3.79E-02 |
| Glycerate | 0.65 (-0.01;1.31) | 9.37E-02 | 0.78 (0.10;1.45) | 4.63E-02 | 0.80 (0.13;1.48) | 3.84E-02 |
| X-23644 | -0.76 (-1.42;-0.10) | 4.69E-02 | -0.79 (-1.46;-0.13) | 3.83E-02 | -0.79 (-1.45;-0.12) | 4.06E-02 |
| 1-Linolenoyl-GPC (18:3)* | 0.74 (0.08;1.39) | 5.23E-02 | 0.78 (0.10;1.45) | 4.63E-02 | 0.80 (0.12;1.47) | 4.07E-02 |
| Pyrraline | -0.79 (-1.44;-0.14) | 3.45E-02 | -0.75 (-1.40;-0.09) | 4.83E-02 | -0.77 (-1.42;-0.11) | 4.25E-02 |
| 5alpha-Androstan-3beta,17beta-Diol Monosulfate (2) | -0.78 (-1.50;-0.07) | 5.90E-02 | -0.80 (-1.52;-0.08) | 5.74E-02 | -0.84 (-1.57;-0.12) | 4.32E-02 |
| Tyrosine | 0.72 (0.07;1.37) | 5.80E-02 | 0.72 (0.07;1.38) | 5.83E-02 | 0.77 (0.11;1.42) | 4.38E-02 |
| 1-Methylnicotinamide | -1.30 (-2.50;-0.10) | 6.26E-02 | -1.42 (-2.61;-0.22) | 3.98E-02 | -1.39 (-2.59;-0.19) | 4.55E-02 |
| 1-Stearoyl-2-Arachidonoyl-GPC (18:0/20:4) | 0.56 (-0.11;1.22) | 1.66E-01 | 0.79 (0.09;1.48) | 5.05E-02 | 0.80 (0.10;1.49) | 4.74E-02 |
| 1,5-Anhydroglucitol (1,5-AG) | 0.52 (-0.14;1.17) | 1.95E-01 | 0.61 (-0.06;1.27) | 1.23E-01 | 0.78 (0.10;1.47) | 4.75E-02 |
| Gamma-Glutamyltyrosine | -0.84 (-1.49;-0.18) | 2.52E-02 | -0.81 (-1.47;-0.14) | 3.44E-02 | -0.76 (-1.43;-0.10) | 4.76E-02 |
| 2-Oleoylglycerol (18:1) | -1.46 (-2.66;-0.26) | 3.34E-02 | -1.40 (-2.61;-0.18) | 4.65E-02 | -1.39 (-2.61;-0.18) | 4.78E-02 |
| Caprylate (8:0) | 0.82 (0.16;1.47) | 2.95E-02 | 0.76 (0.10;1.43) | 4.63E-02 | 0.76 (0.10;1.42) | 4.78E-02 |
| Arachidate (20:0) | 0.83 (0.18;1.48) | 2.58E-02 | 0.81 (0.15;1.48) | 3.25E-02 | 0.76 (0.10;1.43) | 4.80E-02 |
| Linolenate [Alpha Or Gamma; (18:3n3 Or 6)] | 0.82 (0.15;1.49) | 3.26E-02 | 0.82 (0.14;1.49) | 3.60E-02 | 0.77 (0.09;1.45) | 4.94E-02 |
| Oleate/Vaccenate (18:1) | 0.84 (0.17;1.50) | 2.86E-02 | 0.80 (0.12;1.47) | 4.06E-02 | 0.77 (0.09;1.44) | 4.95E-02 |
| Cystine | -1.44 (-2.64;-0.24) | 3.73E-02 | -1.34 (-2.54;-0.13) | 5.61E-02 | -1.36 (-2.57;-0.16) | 5.15E-02 |
| Erucate (22:1n9) | 0.84 (0.19;1.49) | 2.41E-02 | 0.82 (0.16;1.49) | 3.05E-02 | 0.75 (0.09;1.42) | 5.20E-02 |
| 1-Stearoyl-2-Arachidonoyl-GPE (18:0/20:4) | -0.84 (-1.50;-0.19) | 2.41E-02 | -0.75 (-1.43;-0.06) | 6.14E-02 | -0.78 (-1.47;-0.09) | 5.23E-02 |
| Picolinate | -0.92 (-1.59;-0.25) | 1.62E-02 | -0.79 (-1.47;-0.10) | 4.63E-02 | -0.77 (-1.45;-0.09) | 5.23E-02 |
| 1-Myristoyl-2-Arachidonoyl-GPC (14:0/20:4)* | 0.62 (-0.07;1.30) | 1.35E-01 | 0.80 (0.08;1.52) | 5.51E-02 | 0.81 (0.09;1.53) | 5.26E-02 |
| Oxalate (Ethanedioate) | 0.66 (-0.01;1.33) | 9.63E-02 | 0.74 (0.05;1.42) | 6.58E-02 | 0.77 (0.08;1.46) | 5.31E-02 |
| Tetrahydrocortisol Sulfate (1) | -1.60 (-2.81;-0.40) | 1.90E-02 | -1.38 (-2.64;-0.12) | 5.98E-02 | -1.43 (-2.71;-0.15) | 5.51E-02 |
| Isoleucine | -0.86 (-1.56;-0.16) | 3.12E-02 | -0.79 (-1.51;-0.08) | 5.71E-02 | -0.80 (-1.52;-0.08) | 5.59E-02 |
| Stearate (18:0) | 0.80 (0.14;1.46) | 3.42E-02 | 0.80 (0.13;1.46) | 3.73E-02 | 0.74 (0.07;1.41) | 5.66E-02 |
| X-26097 | -0.77 (-1.43;-0.12) | 4.05E-02 | -0.73 (-1.39;-0.07) | 5.55E-02 | -0.73 (-1.39;-0.07) | 5.69E-02 |
| Hexanoylcarnitine (C6) | -0.73 (-1.39;-0.06) | 5.88E-02 | -0.74 (-1.41;-0.07) | 5.90E-02 | -0.74 (-1.41;-0.07) | 5.75E-02 |
| Hexadecasphingosine (D16:1)* | 0.64 (-0.01;1.30) | 9.37E-02 | 0.74 (0.07;1.42) | 5.92E-02 | 0.75 (0.07;1.42) | 5.77E-02 |
| 2-Hydroxyarachidate* | 0.80 (0.15;1.45) | 3.28E-02 | 0.77 (0.10;1.44) | 4.61E-02 | 0.74 (0.07;1.41) | 5.77E-02 |
| 10-Nonadecenoate (19:1n9) | 0.81 (0.13;1.48) | 3.75E-02 | 0.78 (0.09;1.46) | 4.95E-02 | 0.75 (0.07;1.43) | 5.89E-02 |
| Nervonoylcarnitine (C24:1)* | 1.20 (0.00;2.41) | 8.94E-02 | 1.36 (0.11;2.61) | 6.15E-02 | 1.38 (0.12;2.63) | 5.94E-02 |
| X-11632 | -0.66 (-1.31;0.00) | 8.70E-02 | -0.71 (-1.37;-0.05) | 6.64E-02 | -0.73 (-1.39;-0.06) | 5.94E-02 |
| Eicosapentaenoate (EPA; 20:5n3) | 0.67 (0.01;1.33) | 8.56E-02 | 0.78 (0.09;1.48) | 4.97E-02 | 0.76 (0.06;1.45) | 6.00E-02 |
| N6-Acetyllysine | -0.84 (-1.50;-0.19) | 2.32E-02 | -0.74 (-1.40;-0.07) | 5.55E-02 | -0.72 (-1.38;-0.06) | 6.09E-02 |
| 3-Hydroxystachydrine* | -0.82 (-1.48;-0.17) | 2.71E-02 | -0.71 (-1.37;-0.04) | 6.73E-02 | -0.72 (-1.39;-0.06) | 6.09E-02 |
| X-13007 | 1.34 (0.14;2.54) | 5.37E-02 | 1.32 (0.13;2.51) | 5.68E-02 | 1.30 (0.11;2.50) | 6.13E-02 |
| Dibutyl Sulfosuccinate | -0.87 (-1.65;-0.09) | 5.27E-02 | -0.83 (-1.61;-0.05) | 6.65E-02 | -0.84 (-1.62;-0.06) | 6.31E-02 |
| Cyclo(Gly-Pro) | -1.47 (-2.67;-0.27) | 3.30E-02 | -1.31 (-2.52;-0.09) | 6.43E-02 | -1.29 (-2.51;-0.07) | 6.98E-02 |
| 1-Oleoylglycerol (18:1) | -1.53 (-2.74;-0.33) | 2.60E-02 | -1.33 (-2.58;-0.09) | 6.65E-02 | -1.32 (-2.58;-0.07) | 7.09E-02 |
| Biliverdin | 0.58 (-0.09;1.24) | 1.49E-01 | 0.67 (-0.01;1.35) | 9.20E-02 | 0.72 (0.04;1.40) | 7.09E-02 |
| Ceramide (D18:2/24:1, D18:1/24:2)* | -0.86 (-1.52;-0.20) | 2.25E-02 | -0.78 (-1.46;-0.10) | 4.89E-02 | -0.72 (-1.41;-0.04) | 7.13E-02 |
| X-22776 | 0.64 (-0.01;1.29) | 9.38E-02 | 0.70 (0.04;1.35) | 6.83E-02 | 0.69 (0.03;1.35) | 7.27E-02 |
| Betaine | 0.62 (-0.05;1.29) | 1.17E-01 | 0.67 (-0.01;1.35) | 9.66E-02 | 0.71 (0.03;1.40) | 7.33E-02 |
| Maleate | -0.63 (-1.28;0.03) | 1.05E-01 | -0.64 (-1.30;0.02) | 1.00E-01 | -0.70 (-1.37;-0.03) | 7.36E-02 |
| 1-Stearoyl-GPC (18:0) | 0.46 (-0.19;1.12) | 2.52E-01 | 0.71 (-0.02;1.44) | 9.69E-02 | 0.77 (0.03;1.50) | 7.39E-02 |
| Gamma-Glutamylglutamate | -0.82 (-1.47;-0.16) | 3.00E-02 | -0.76 (-1.44;-0.07) | 5.74E-02 | -0.72 (-1.40;-0.03) | 7.39E-02 |
| X-12456 | 0.55 (-0.10;1.21) | 1.63E-01 | 0.73 (0.02;1.44) | 7.79E-02 | 0.74 (0.03;1.46) | 7.39E-02 |
| X-17690 | -0.69 (-1.35;-0.03) | 7.21E-02 | -0.72 (-1.39;-0.06) | 6.22E-02 | -0.70 (-1.36;-0.03) | 7.39E-02 |
| 1-Oleoyl-2-Linoleoyl-GPE (18:1/18:2)* | -0.69 (-1.34;-0.04) | 6.93E-02 | -0.66 (-1.32;0.00) | 8.71E-02 | -0.68 (-1.34;-0.03) | 7.58E-02 |
| 2-Aminoadipate | -0.86 (-1.64;-0.07) | 6.16E-02 | -0.86 (-1.68;-0.04) | 7.19E-02 | -0.85 (-1.67;-0.03) | 7.60E-02 |
| 1-Oleoyl-2-Docosahexaenoyl-GPC (18:1/22:6)* | 0.46 (-0.21;1.13) | 2.65E-01 | 0.67 (-0.02;1.36) | 9.97E-02 | 0.72 (0.03;1.41) | 7.65E-02 |
| Beta-Citrylglutamate | -0.74 (-1.39;-0.09) | 4.82E-02 | -0.69 (-1.35;-0.03) | 7.47E-02 | -0.68 (-1.35;-0.02) | 7.79E-02 |
| Behenoyl Dihydrosphingomyelin (D18:0/22:0)* | 0.53 (-0.14;1.19) | 1.90E-01 | 0.71 (-0.01;1.42) | 9.35E-02 | 0.74 (0.02;1.45) | 7.79E-02 |
| Palmitoyl Dihydrosphingomyelin (D18:0/16:0)* | 0.54 (-0.12;1.20) | 1.79E-01 | 0.67 (-0.06;1.39) | 1.20E-01 | 0.75 (0.02;1.48) | 7.82E-02 |
| X-18913 | -0.71 (-1.38;-0.04) | 7.03E-02 | -0.71 (-1.38;-0.04) | 6.87E-02 | -0.69 (-1.37;-0.02) | 7.82E-02 |
| X-24947 | -0.66 (-1.33;0.00) | 8.79E-02 | -0.66 (-1.33;0.00) | 9.08E-02 | -0.69 (-1.35;-0.02) | 7.82E-02 |
| 1-Palmitoyl-2-Stearoyl-GPC (16:0/18:0) | 0.50 (-0.15;1.15) | 2.03E-01 | 0.67 (-0.03;1.36) | 1.02E-01 | 0.72 (0.02;1.41) | 7.86E-02 |
| 1-Linolenoylglycerol (18:3) | -1.36 (-2.57;-0.15) | 5.31E-02 | -1.27 (-2.50;-0.05) | 7.40E-02 | -1.26 (-2.49;-0.03) | 7.88E-02 |
| Isoleucylglycine | -0.62 (-1.27;0.04) | 1.12E-01 | -0.70 (-1.37;-0.03) | 7.40E-02 | -0.70 (-1.37;-0.02) | 7.88E-02 |
| 10-Undecenoate (11:1n1) | 0.71 (0.05;1.36) | 6.52E-02 | 0.64 (-0.02;1.31) | 1.01E-01 | 0.68 (0.02;1.35) | 7.96E-02 |
| Margarate (17:0) | 0.70 (0.04;1.37) | 7.10E-02 | 0.74 (0.07;1.42) | 5.68E-02 | 0.69 (0.02;1.36) | 8.02E-02 |
| Hexadecadienoate (16:2n6) | 0.69 (0.00;1.38) | 8.70E-02 | 0.73 (0.04;1.43) | 7.09E-02 | 0.71 (0.02;1.41) | 8.08E-02 |
| Deoxycholic Acid Glucuronide | 0.62 (-0.05;1.29) | 1.19E-01 | 0.61 (-0.07;1.28) | 1.29E-01 | 0.69 (0.01;1.37) | 8.12E-02 |
| Tetradecadienoate (14:2)* | 0.72 (0.06;1.39) | 6.20E-02 | 0.72 (0.05;1.39) | 6.64E-02 | 0.69 (0.01;1.36) | 8.28E-02 |
| Caproate (6:0) | 0.68 (0.03;1.33) | 7.51E-02 | 0.67 (0.01;1.33) | 8.16E-02 | 0.66 (0.01;1.32) | 8.33E-02 |
| X-10458 | -0.66 (-1.31;-0.01) | 8.44E-02 | -0.66 (-1.32;-0.01) | 8.36E-02 | -0.66 (-1.32;-0.01) | 8.35E-02 |
| 2-Hydroxy-3-Methylvalerate | -0.60 (-1.28;0.08) | 1.42E-01 | -0.67 (-1.36;0.03) | 1.02E-01 | -0.70 (-1.40;-0.01) | 8.38E-02 |
| X-11880 | 0.52 (-0.14;1.17) | 1.95E-01 | 0.67 (0.00;1.35) | 8.76E-02 | 0.68 (0.01;1.35) | 8.44E-02 |
| Stachydrine | -0.76 (-1.41;-0.11) | 4.38E-02 | -0.67 (-1.34;-0.01) | 8.36E-02 | -0.67 (-1.33;0.00) | 8.53E-02 |
| Linolenoylcarnitine (C18:3)* | -0.77 (-1.43;-0.11) | 4.43E-02 | -0.71 (-1.37;-0.04) | 6.83E-02 | -0.67 (-1.34;0.00) | 8.53E-02 |
| X-15245 | 0.64 (-0.01;1.29) | 9.65E-02 | 0.66 (0.00;1.33) | 9.02E-02 | 0.67 (0.00;1.34) | 8.57E-02 |
| Glycosyl Ceramide (D18:1/23:1, D17:1/24:1)* | -0.96 (-1.74;-0.18) | 3.20E-02 | -0.96 (-1.79;-0.13) | 4.57E-02 | -0.84 (-1.68;0.00) | 8.62E-02 |
| Gamma-Glutamylmethionine | -0.71 (-1.38;-0.04) | 7.04E-02 | -0.70 (-1.37;-0.02) | 7.63E-02 | -0.67 (-1.35;0.00) | 8.66E-02 |
| 3-Hydroxydodecanedioate* | -0.44 (-1.11;0.23) | 2.93E-01 | -0.59 (-1.27;0.09) | 1.45E-01 | -0.69 (-1.37;0.00) | 8.76E-02 |
| Bilirubin Degradation Product, C17H18N2O4 (3)** | 0.52 (-0.14;1.18) | 1.95E-01 | 0.62 (-0.05;1.29) | 1.21E-01 | 0.67 (0.00;1.35) | 8.76E-02 |
| 5alpha-Androstan-3beta,17beta-Diol Disulfate | -0.65 (-1.44;0.14) | 1.71E-01 | -0.73 (-1.53;0.08) | 1.30E-01 | -0.81 (-1.62;0.00) | 8.83E-02 |
| 1-Arachidonoyl-GPI (20:4)* | 0.51 (-0.14;1.16) | 1.97E-01 | 0.67 (-0.01;1.35) | 9.38E-02 | 0.68 (0.00;1.35) | 8.85E-02 |
| Indoleacetoylcarnitine* | 0.57 (-0.09;1.23) | 1.52E-01 | 0.62 (-0.04;1.29) | 1.14E-01 | 0.67 (0.00;1.34) | 8.95E-02 |
| Sphingosine | 0.56 (-0.09;1.22) | 1.49E-01 | 0.66 (-0.01;1.32) | 9.26E-02 | 0.66 (0.00;1.33) | 8.96E-02 |
| Androstenediol (3beta,17beta) Monosulfate (2) | -0.50 (-1.17;0.16) | 2.08E-01 | -0.62 (-1.30;0.06) | 1.22E-01 | -0.67 (-1.35;0.01) | 9.14E-02 |
| 10-Heptadecenoate (17:1n7) | 0.74 (0.05;1.43) | 6.41E-02 | 0.71 (0.01;1.40) | 8.25E-02 | 0.69 (-0.01;1.38) | 9.17E-02 |
| Lactosyl-N-Behenoyl-Sphingosine (D18:1/22:0)* | 0.66 (0.00;1.33) | 8.83E-02 | 0.69 (-0.02;1.39) | 9.76E-02 | 0.70 (-0.01;1.40) | 9.26E-02 |
| Arachidonate (20:4n6) | 0.60 (-0.07;1.26) | 1.33E-01 | 0.71 (0.03;1.38) | 7.24E-02 | 0.66 (-0.01;1.34) | 9.26E-02 |
| Proline | -0.73 (-1.40;-0.07) | 5.71E-02 | -0.65 (-1.32;0.03) | 1.04E-01 | -0.66 (-1.33;0.02) | 9.68E-02 |
| Oleoyl Ethanolamide | 0.66 (-0.01;1.32) | 9.29E-02 | 0.67 (0.00;1.35) | 9.09E-02 | 0.66 (-0.02;1.34) | 9.74E-02 |
| X-21471 | 0.53 (-0.13;1.19) | 1.87E-01 | 0.71 (0.01;1.42) | 8.36E-02 | 0.68 (-0.02;1.39) | 1.00E-01 |
| Palmitoleate (16:1n7) | 0.79 (0.09;1.48) | 5.15E-02 | 0.70 (-0.02;1.41) | 9.75E-02 | 0.69 (-0.02;1.41) | 1.00E-01 |
| 1-Palmitoyl-GPE (16:0) | -0.73 (-1.39;-0.07) | 5.47E-02 | -0.66 (-1.33;0.02) | 9.79E-02 | -0.65 (-1.33;0.02) | 1.01E-01 |
| Androstenediol (3beta,17beta) Disulfate (1) | -0.46 (-1.19;0.26) | 3.03E-01 | -0.67 (-1.43;0.09) | 1.40E-01 | -0.73 (-1.50;0.03) | 1.02E-01 |
| Hypotaurine | -0.63 (-1.30;0.03) | 1.07E-01 | -0.69 (-1.37;-0.01) | 8.29E-02 | -0.66 (-1.34;0.03) | 1.02E-01 |
| Docosapentaenoate (N3 DPA; 22:5n3) | 0.64 (-0.04;1.31) | 1.10E-01 | 0.71 (0.02;1.40) | 7.78E-02 | 0.66 (-0.03;1.35) | 1.03E-01 |
| X-21286 | -0.76 (-1.41;-0.10) | 4.59E-02 | -0.66 (-1.34;0.02) | 9.84E-02 | -0.65 (-1.33;0.03) | 1.04E-01 |
| 5alpha-Androstan-3alpha,17beta-Diol Monosulfate (1) | -0.63 (-1.31;0.05) | 1.19E-01 | -0.61 (-1.29;0.08) | 1.35E-01 | -0.65 (-1.33;0.03) | 1.05E-01 |
| Salicylate | -0.86 (-1.52;-0.20) | 2.27E-02 | -0.74 (-1.48;0.01) | 9.20E-02 | -0.71 (-1.45;0.04) | 1.06E-01 |
| X-21470 | -0.55 (-1.24;0.13) | 1.79E-01 | -0.59 (-1.29;0.10) | 1.53E-01 | -0.66 (-1.36;0.03) | 1.06E-01 |
| 1-Stearoyl-2-Docosahexaenoyl-GPC (18:0/22:6) | 0.41 (-0.25;1.06) | 3.18E-01 | 0.61 (-0.07;1.28) | 1.31E-01 | 0.64 (-0.03;1.32) | 1.06E-01 |
| Linoleate (18:2n6) | 0.65 (-0.01;1.31) | 9.44E-02 | 0.68 (0.01;1.35) | 8.36E-02 | 0.63 (-0.04;1.30) | 1.09E-01 |
| 1-Linoleoyl-2-Arachidonoyl-GPC (18:2/20:4n6)* | 0.52 (-0.15;1.19) | 1.99E-01 | 0.63 (-0.08;1.35) | 1.36E-01 | 0.67 (-0.04;1.39) | 1.11E-01 |
| Hydroxy-CMPF* | -0.72 (-1.37;-0.06) | 5.81E-02 | -0.66 (-1.32;0.01) | 9.20E-02 | -0.62 (-1.28;0.05) | 1.17E-01 |
| Nicotinamide | 0.58 (-0.08;1.24) | 1.39E-01 | 0.62 (-0.05;1.28) | 1.17E-01 | 0.61 (-0.05;1.28) | 1.18E-01 |
| 1-Oleoyl-GPC (18:1) | 0.54 (-0.12;1.19) | 1.75E-01 | 0.60 (-0.09;1.29) | 1.45E-01 | 0.64 (-0.05;1.34) | 1.18E-01 |
| X-23782 | 0.67 (0.00;1.34) | 8.77E-02 | 0.65 (-0.02;1.33) | 9.97E-02 | 0.62 (-0.06;1.30) | 1.22E-01 |
| Dihomo-Linolenate (20:3n3 Or N6) | 0.57 (-0.10;1.24) | 1.57E-01 | 0.66 (-0.02;1.34) | 9.74E-02 | 0.62 (-0.06;1.30) | 1.23E-01 |
| Dihydroferulate | -1.11 (-2.32;0.10) | 1.23E-01 | -1.07 (-2.28;0.14) | 1.38E-01 | -1.10 (-2.32;0.11) | 1.26E-01 |
| Edta | 0.48 (-0.18;1.14) | 2.30E-01 | 0.53 (-0.13;1.19) | 1.81E-01 | 0.60 (-0.06;1.26) | 1.28E-01 |
| Carotene Diol (3) | -0.65 (-1.30;0.01) | 9.52E-02 | -0.65 (-1.33;0.03) | 1.04E-01 | -0.61 (-1.29;0.07) | 1.31E-01 |
| Taurodeoxycholic Acid 3-Sulfate | -0.58 (-1.23;0.07) | 1.35E-01 | -0.60 (-1.26;0.05) | 1.21E-01 | -0.59 (-1.24;0.07) | 1.33E-01 |
| Bilirubin Degradation Product, C17H18N2O4 (2)** | 0.44 (-0.22;1.10) | 2.83E-01 | 0.54 (-0.14;1.21) | 1.87E-01 | 0.60 (-0.07;1.27) | 1.35E-01 |
| 4-Hydroxyglutamate | -0.61 (-1.28;0.05) | 1.20E-01 | -0.61 (-1.33;0.12) | 1.63E-01 | -0.65 (-1.38;0.08) | 1.35E-01 |
| Hyocholate | 0.53 (-0.13;1.19) | 1.87E-01 | 0.51 (-0.15;1.17) | 2.05E-01 | 0.59 (-0.08;1.26) | 1.37E-01 |
| Glucuronide Of Piperine Metabolite C17H21NO3 (4)* | -0.56 (-1.22;0.10) | 1.59E-01 | -0.63 (-1.32;0.06) | 1.24E-01 | -0.61 (-1.30;0.08) | 1.38E-01 |
| 2-Hydroxybehenate | 0.59 (-0.06;1.24) | 1.31E-01 | 0.67 (0.00;1.33) | 8.81E-02 | 0.59 (-0.08;1.26) | 1.39E-01 |
| X-12112 | -0.49 (-1.15;0.16) | 2.11E-01 | -0.57 (-1.23;0.09) | 1.46E-01 | -0.58 (-1.24;0.08) | 1.39E-01 |
| 1-Stearoyl-2-Oleoyl-GPC (18:0/18:1) | 0.53 (-0.14;1.19) | 1.89E-01 | 0.57 (-0.12;1.25) | 1.69E-01 | 0.60 (-0.08;1.28) | 1.41E-01 |
| Bilirubin (E,E)* | 0.49 (-0.17;1.14) | 2.21E-01 | 0.55 (-0.12;1.22) | 1.71E-01 | 0.58 (-0.09;1.25) | 1.49E-01 |
| 2-Aminooctanoate | -0.55 (-1.21;0.10) | 1.62E-01 | -0.61 (-1.29;0.06) | 1.24E-01 | -0.58 (-1.26;0.09) | 1.49E-01 |
| 1-Palmitoleoyl-GPC (16:1)* | 0.51 (-0.16;1.17) | 2.07E-01 | 0.55 (-0.13;1.23) | 1.83E-01 | 0.59 (-0.10;1.27) | 1.51E-01 |
| X-11849 | -0.65 (-1.30;0.00) | 9.17E-02 | -0.61 (-1.27;0.05) | 1.19E-01 | -0.57 (-1.23;0.09) | 1.52E-01 |
| Thyroxine | 0.53 (-0.13;1.19) | 1.88E-01 | 0.56 (-0.11;1.23) | 1.65E-01 | 0.58 (-0.10;1.25) | 1.52E-01 |
| (R)-3-Hydroxybutyrylcarnitine | -0.96 (-2.17;0.26) | 1.93E-01 | -1.09 (-2.34;0.17) | 1.46E-01 | -1.11 (-2.41;0.19) | 1.52E-01 |
| Glyco-Beta-Muricholate** | 0.53 (-0.12;1.19) | 1.79E-01 | 0.51 (-0.15;1.16) | 2.06E-01 | 0.56 (-0.10;1.22) | 1.53E-01 |
| Sphingomyelin (D18:0/18:0, D19:0/17:0)* | 0.45 (-0.22;1.12) | 2.80E-01 | 0.57 (-0.16;1.30) | 2.00E-01 | 0.62 (-0.11;1.36) | 1.56E-01 |
| Bilirubin Degradation Product, C17H18N2O4 (1)** | 0.42 (-0.24;1.08) | 3.09E-01 | 0.51 (-0.16;1.18) | 2.15E-01 | 0.57 (-0.10;1.25) | 1.57E-01 |
| Gamma-Glutamyl-Alpha-Lysine | -0.67 (-1.32;-0.02) | 7.86E-02 | -0.58 (-1.23;0.07) | 1.36E-01 | -0.55 (-1.20;0.10) | 1.58E-01 |
| Hypoxanthine | -1.04 (-2.24;0.16) | 1.47E-01 | -1 (-2.20;0.19) | 1.64E-01 | -1.02 (-2.22;0.19) | 1.60E-01 |
| 3-Hydroxybutyrate (BHBA) | 0.71 (0.05;1.37) | 6.27E-02 | 0.60 (-0.06;1.27) | 1.28E-01 | 0.56 (-0.11;1.23) | 1.62E-01 |
| Gamma-Glutamyl-Epsilon-Lysine | -0.69 (-1.46;0.08) | 1.36E-01 | -0.69 (-1.47;0.08) | 1.34E-01 | -0.65 (-1.42;0.13) | 1.63E-01 |
| Phosphoethanolamine | 0.46 (-0.20;1.11) | 2.58E-01 | 0.54 (-0.13;1.21) | 1.81E-01 | 0.56 (-0.11;1.23) | 1.64E-01 |
| X-12798 | 0.38 (-0.27;1.04) | 3.53E-01 | 0.48 (-0.17;1.14) | 2.29E-01 | 0.55 (-0.11;1.21) | 1.66E-01 |
| Sphingomyelin (D18:2/24:2)* | -0.69 (-1.38;0.00) | 8.89E-02 | -0.69 (-1.41;0.04) | 1.09E-01 | -0.62 (-1.36;0.12) | 1.66E-01 |
| Lignoceroylcarnitine (C24)* | 0.41 (-0.24;1.07) | 3.10E-01 | 0.55 (-0.13;1.22) | 1.83E-01 | 0.56 (-0.11;1.24) | 1.67E-01 |
| Docosahexaenoate (DHA; 22:6n3) | 0.48 (-0.19;1.14) | 2.41E-01 | 0.60 (-0.09;1.28) | 1.43E-01 | 0.57 (-0.12;1.25) | 1.67E-01 |
| X-24306 | -0.64 (-1.30;0.01) | 9.64E-02 | -0.57 (-1.23;0.08) | 1.44E-01 | -0.55 (-1.21;0.11) | 1.68E-01 |
| 5-Dodecenoate (12:1n7) | 0.66 (-0.04;1.37) | 1.13E-01 | 0.58 (-0.13;1.30) | 1.78E-01 | 0.60 (-0.12;1.31) | 1.68E-01 |
| X-16576 | 0.54 (-0.12;1.19) | 1.74E-01 | 0.47 (-0.19;1.13) | 2.48E-01 | 0.55 (-0.12;1.22) | 1.70E-01 |
| Linoleoyl-Linolenoyl-Glycerol (18:2/18:3) [2]* | -0.71 (-1.49;0.06) | 1.22E-01 | -0.63 (-1.43;0.17) | 1.92E-01 | -0.65 (-1.45;0.14) | 1.72E-01 |
| X-12013 | -1.26 (-2.47;-0.05) | 7.43E-02 | -1.03 (-2.25;0.19) | 1.59E-01 | -1 (-2.23;0.22) | 1.73E-01 |
| Iminodiacetate (IDA) | 0.89 (-0.32;2.10) | 2.25E-01 | 1.00 (-0.21;2.20) | 1.69E-01 | 0.99 (-0.22;2.19) | 1.73E-01 |
| X-21796 | -0.64 (-1.32;0.03) | 1.08E-01 | -0.60 (-1.28;0.08) | 1.39E-01 | -0.56 (-1.24;0.12) | 1.73E-01 |
| Tryptophan Betaine | 0.59 (-0.08;1.26) | 1.42E-01 | 0.56 (-0.12;1.24) | 1.70E-01 | 0.55 (-0.13;1.23) | 1.76E-01 |
| Palmitate (16:0) | 0.62 (-0.04;1.28) | 1.16E-01 | 0.59 (-0.08;1.27) | 1.38E-01 | 0.55 (-0.13;1.22) | 1.77E-01 |
| Sulfate Of Piperine Metabolite C18H21NO3 (1)* | -0.52 (-1.19;0.14) | 1.93E-01 | -0.57 (-1.25;0.10) | 1.57E-01 | -0.55 (-1.22;0.13) | 1.78E-01 |
| X-17612 | -0.48 (-1.14;0.19) | 2.45E-01 | -0.56 (-1.23;0.11) | 1.67E-01 | -0.55 (-1.22;0.13) | 1.78E-01 |
| Cis-4-Decenoate (10:1n6)* | 0.50 (-0.15;1.16) | 2.05E-01 | 0.53 (-0.13;1.19) | 1.81E-01 | 0.53 (-0.13;1.19) | 1.86E-01 |
| X-12849 | -0.64 (-1.42;0.14) | 1.76E-01 | -0.69 (-1.48;0.09) | 1.38E-01 | -0.62 (-1.41;0.16) | 1.90E-01 |
| 5-Acetylamino-6-Formylamino-3-Methyluracil | -0.95 (-2.15;0.25) | 1.92E-01 | -0.94 (-2.14;0.26) | 1.98E-01 | -0.95 (-2.16;0.25) | 1.92E-01 |
| X-25265 | 0.50 (-0.15;1.15) | 2.03E-01 | 0.52 (-0.13;1.17) | 1.89E-01 | 0.52 (-0.14;1.17) | 1.92E-01 |
| 2-Hydroxyglutarate | -0.62 (-1.40;0.16) | 1.89E-01 | -0.61 (-1.40;0.17) | 1.98E-01 | -0.62 (-1.41;0.17) | 1.92E-01 |
| Docosatrienoate (22:3n6)* | 0.96 (-0.25;2.18) | 1.90E-01 | 0.97 (-0.28;2.22) | 2.00E-01 | 0.98 (-0.27;2.23) | 1.94E-01 |
| 3-(3-Hydroxyphenyl)Propionate | -0.54 (-1.19;0.11) | 1.71E-01 | -0.52 (-1.18;0.14) | 1.90E-01 | -0.52 (-1.18;0.14) | 1.97E-01 |
| Pregnenetriol Disulfate* | -0.49 (-1.21;0.23) | 2.68E-01 | -0.55 (-1.28;0.18) | 2.20E-01 | -0.57 (-1.30;0.16) | 1.98E-01 |
| X-12261 | -1.24 (-2.46;-0.01) | 8.55E-02 | -1.00 (-2.23;0.24) | 1.81E-01 | -0.97 (-2.21;0.27) | 1.99E-01 |
| X-18922 | -0.44 (-1.09;0.22) | 2.79E-01 | -0.48 (-1.14;0.18) | 2.36E-01 | -0.51 (-1.18;0.15) | 2.03E-01 |
| X-21312 | -1.19 (-2.40;0.01) | 9.34E-02 | -0.91 (-2.13;0.30) | 2.19E-01 | -0.94 (-2.16;0.28) | 2.03E-01 |
| (2-Butoxyethoxy)Acetic Acid | -0.59 (-1.37;0.18) | 2.05E-01 | -0.61 (-1.39;0.17) | 1.94E-01 | -0.60 (-1.37;0.18) | 2.05E-01 |
| X-14939 | 0.49 (-0.17;1.14) | 2.21E-01 | 0.49 (-0.17;1.15) | 2.27E-01 | 0.51 (-0.16;1.17) | 2.09E-01 |
| Phenylpyruvate | -0.58 (-1.24;0.08) | 1.43E-01 | -0.53 (-1.19;0.13) | 1.88E-01 | -0.51 (-1.17;0.16) | 2.10E-01 |
| Carotene Diol (1) | -0.57 (-1.23;0.09) | 1.53E-01 | -0.53 (-1.23;0.17) | 2.12E-01 | -0.53 (-1.23;0.17) | 2.11E-01 |
| Myristoleate (14:1n5) | 0.62 (-0.09;1.34) | 1.46E-01 | 0.53 (-0.19;1.26) | 2.28E-01 | 0.55 (-0.18;1.28) | 2.11E-01 |
| Homoarginine | 0.39 (-0.28;1.07) | 3.57E-01 | 0.47 (-0.21;1.15) | 2.56E-01 | 0.51 (-0.16;1.19) | 2.13E-01 |
| Spermidine | -0.81 (-2.03;0.41) | 2.84E-01 | -0.88 (-2.10;0.34) | 2.37E-01 | -0.93 (-2.15;0.30) | 2.14E-01 |
| X-11372 | 0.32 (-0.34;0.98) | 4.41E-01 | 0.48 (-0.20;1.15) | 2.48E-01 | 0.51 (-0.16;1.18) | 2.14E-01 |
| Lignoceroyl Sphingomyelin (D18:1/24:0) | 0.41 (-0.25;1.07) | 3.19E-01 | 0.52 (-0.22;1.26) | 2.51E-01 | 0.55 (-0.18;1.29) | 2.18E-01 |
| X-12462 | 0.39 (-0.27;1.04) | 3.44E-01 | 0.46 (-0.20;1.12) | 2.51E-01 | 0.49 (-0.17;1.14) | 2.24E-01 |
| 1-Stearoyl-2-Arachidonoyl-GPS (18:0/20:4) | 0.53 (-0.24;1.30) | 2.68E-01 | 0.57 (-0.21;1.35) | 2.32E-01 | 0.58 (-0.20;1.35) | 2.24E-01 |
| Propionylglycine | -0.74 (-1.95;0.47) | 3.26E-01 | -0.87 (-2.10;0.36) | 2.47E-01 | -0.91 (-2.15;0.33) | 2.29E-01 |
| X-17654 | 0.38 (-0.28;1.03) | 3.60E-01 | 0.47 (-0.20;1.13) | 2.50E-01 | 0.49 (-0.18;1.15) | 2.30E-01 |
| Sphingomyelin (D18:0/20:0, D16:0/22:0)* | 0.35 (-0.32;1.02) | 4.09E-01 | 0.48 (-0.23;1.20) | 2.75E-01 | 0.53 (-0.19;1.24) | 2.30E-01 |
| 1-Palmitoyl-GPG (16:0)* | -0.54 (-1.20;0.11) | 1.69E-01 | -0.49 (-1.16;0.18) | 2.28E-01 | -0.49 (-1.16;0.18) | 2.32E-01 |
| 1-Linoleoyl-GPC (18:2) | 0.41 (-0.24;1.07) | 3.10E-01 | 0.48 (-0.23;1.19) | 2.68E-01 | 0.52 (-0.19;1.23) | 2.34E-01 |
| Glucuronide Of Piperine Metabolite C17H21NO3 (5)* | -0.83 (-2.04;0.39) | 2.71E-01 | -0.92 (-2.17;0.33) | 2.27E-01 | -0.91 (-2.16;0.34) | 2.37E-01 |
| 1,2-Dipalmitoyl-GPC (16:0/16:0) | 0.41 (-0.24;1.06) | 3.15E-01 | 0.46 (-0.22;1.14) | 2.75E-01 | 0.50 (-0.19;1.18) | 2.37E-01 |
| Choline Phosphate | 0.38 (-0.28;1.04) | 3.60E-01 | 0.52 (-0.16;1.21) | 2.11E-01 | 0.50 (-0.19;1.19) | 2.37E-01 |
| Phenylacetylcarnitine | -0.62 (-1.28;0.03) | 1.07E-01 | -0.50 (-1.16;0.17) | 2.21E-01 | -0.48 (-1.15;0.19) | 2.39E-01 |
| Palmitoyl Ethanolamide | 0.86 (-0.38;2.09) | 2.59E-01 | 0.95 (-0.32;2.22) | 2.21E-01 | 0.92 (-0.36;2.20) | 2.39E-01 |
| Cholesterol Sulfate | -0.43 (-1.11;0.26) | 3.15E-01 | -0.48 (-1.22;0.27) | 2.99E-01 | -0.54 (-1.28;0.21) | 2.40E-01 |
| Sphinganine | 0.41 (-0.25;1.06) | 3.17E-01 | 0.47 (-0.20;1.14) | 2.50E-01 | 0.48 (-0.19;1.15) | 2.41E-01 |
| X-21607 | 0.44 (-0.21;1.10) | 2.72E-01 | 0.47 (-0.19;1.13) | 2.43E-01 | 0.47 (-0.19;1.13) | 2.41E-01 |
| Sphingomyelin (D18:1/22:2, D18:2/22:1, D16:1/24:2)* | -0.64 (-1.33;0.06) | 1.24E-01 | -0.60 (-1.33;0.13) | 1.71E-01 | -0.53 (-1.27;0.21) | 2.41E-01 |
| 3-Phosphoglycerate | 0.33 (-0.32;0.98) | 4.28E-01 | 0.47 (-0.19;1.13) | 2.44E-01 | 0.47 (-0.19;1.13) | 2.43E-01 |
| 1-Arachidonoyl-GPE (20:4n6)* | 0.40 (-0.25;1.05) | 3.27E-01 | 0.49 (-0.18;1.15) | 2.29E-01 | 0.47 (-0.19;1.14) | 2.43E-01 |
| 1-Palmitoleoyl-2-Linolenoyl-GPC (16:1/18:3)* | 0.45 (-0.22;1.12) | 2.76E-01 | 0.46 (-0.23;1.14) | 2.77E-01 | 0.49 (-0.20;1.17) | 2.43E-01 |
| X-23583 | -0.98 (-2.18;0.23) | 1.79E-01 | -0.87 (-2.07;0.34) | 2.39E-01 | -0.86 (-2.07;0.34) | 2.43E-01 |
| Tetradecanedioate (C14-DC) | -0.34 (-1.01;0.33) | 4.22E-01 | -0.45 (-1.13;0.23) | 2.79E-01 | -0.48 (-1.16;0.19) | 2.44E-01 |
| 2-Hydroxybutyrate/2-Hydroxyisobutyrate | 0.48 (-0.18;1.13) | 2.34E-01 | 0.53 (-0.15;1.22) | 1.97E-01 | 0.49 (-0.20;1.18) | 2.47E-01 |
| Adrenate (22:4n6) | 0.91 (-0.33;2.15) | 2.30E-01 | 0.90 (-0.35;2.15) | 2.40E-01 | 0.89 (-0.37;2.14) | 2.48E-01 |
| (14 Or 15)-Methylpalmitate (A17:0 Or I17:0) | 0.53 (-0.15;1.21) | 1.95E-01 | 0.48 (-0.21;1.18) | 2.53E-01 | 0.49 (-0.21;1.18) | 2.53E-01 |
| Heneicosapentaenoate (21:5n3) | 0.45 (-0.21;1.11) | 2.72E-01 | 0.51 (-0.17;1.18) | 2.19E-01 | 0.47 (-0.20;1.15) | 2.56E-01 |
| 2,2'-Methylenebis(6-Tert-Butyl-P-Cresol) | 0.89 (-0.32;2.09) | 2.25E-01 | 0.83 (-0.37;2.03) | 2.61E-01 | 0.84 (-0.36;2.04) | 2.56E-01 |
| Trans-Urocanate | 0.38 (-0.27;1.03) | 3.55E-01 | 0.40 (-0.26;1.06) | 3.26E-01 | 0.46 (-0.20;1.12) | 2.56E-01 |
| Docosapentaenoate (N6 DPA; 22:5n6) | 0.52 (-0.13;1.17) | 1.89E-01 | 0.50 (-0.15;1.16) | 2.07E-01 | 0.46 (-0.20;1.12) | 2.56E-01 |
| Lysine | 0.34 (-0.31;0.99) | 4.13E-01 | 0.41 (-0.24;1.07) | 3.04E-01 | 0.45 (-0.20;1.11) | 2.59E-01 |
| Leucine | -0.58 (-1.27;0.12) | 1.71E-01 | -0.49 (-1.19;0.22) | 2.61E-01 | -0.49 (-1.20;0.22) | 2.59E-01 |
| 1-Oleoyl-2-Arachidonoyl-GPE (18:1/20:4)* | -0.52 (-1.18;0.13) | 1.89E-01 | -0.42 (-1.10;0.26) | 3.18E-01 | -0.47 (-1.15;0.21) | 2.60E-01 |
| Bilirubin (E,Z Or Z,E)* | 0.34 (-0.32;1.00) | 4.17E-01 | 0.42 (-0.26;1.09) | 3.20E-01 | 0.47 (-0.21;1.14) | 2.62E-01 |
| Arachidonoylcarnitine (C20:4) | -0.61 (-1.28;0.07) | 1.33E-01 | -0.50 (-1.19;0.18) | 2.28E-01 | -0.47 (-1.16;0.22) | 2.63E-01 |
| X-12839 | -0.69 (-1.93;0.56) | 3.82E-01 | -0.85 (-2.10;0.40) | 2.67E-01 | -0.86 (-2.12;0.39) | 2.63E-01 |
| Alpha-Hydroxyisocaproate | -0.39 (-1.06;0.29) | 3.60E-01 | -0.47 (-1.15;0.21) | 2.62E-01 | -0.47 (-1.16;0.22) | 2.65E-01 |
| Tryptophan | 0.36 (-0.30;1.03) | 3.87E-01 | 0.41 (-0.26;1.08) | 3.20E-01 | 0.46 (-0.21;1.12) | 2.67E-01 |
| 2-Palmitoleoyl-GPC (16:1)* | 0.40 (-0.25;1.05) | 3.24E-01 | 0.39 (-0.27;1.05) | 3.43E-01 | 0.45 (-0.21;1.11) | 2.67E-01 |
| Myristate (14:0) | 0.54 (-0.14;1.21) | 1.93E-01 | 0.50 (-0.19;1.18) | 2.39E-01 | 0.47 (-0.22;1.15) | 2.72E-01 |
| X-25267 | 0.38 (-0.27;1.03) | 3.46E-01 | 0.41 (-0.25;1.06) | 3.16E-01 | 0.44 (-0.21;1.09) | 2.73E-01 |
| X-18901 | -0.55 (-1.20;0.11) | 1.66E-01 | -0.47 (-1.14;0.21) | 2.61E-01 | -0.45 (-1.13;0.22) | 2.75E-01 |
| X-12104 | -0.98 (-2.20;0.23) | 1.82E-01 | -0.81 (-2.05;0.43) | 2.89E-01 | -0.83 (-2.07;0.41) | 2.78E-01 |
| Glycochenodeoxycholate | 0.51 (-0.15;1.16) | 2.03E-01 | 0.47 (-0.19;1.14) | 2.41E-01 | 0.44 (-0.22;1.10) | 2.80E-01 |
| Sulfate Of Piperine Metabolite C18H21NO3 (3)* | -0.41 (-1.08;0.25) | 3.17E-01 | -0.46 (-1.14;0.21) | 2.62E-01 | -0.45 (-1.12;0.23) | 2.81E-01 |
| 1-Palmitoyl-2-Arachidonoyl-GPC (16:0/20:4n6) | 0.32 (-0.34;0.97) | 4.54E-01 | 0.45 (-0.24;1.14) | 2.89E-01 | 0.45 (-0.24;1.14) | 2.88E-01 |
| X-11795 | -0.35 (-1.01;0.31) | 3.97E-01 | -0.43 (-1.11;0.24) | 3.01E-01 | -0.44 (-1.12;0.23) | 2.88E-01 |
| Uridine | 0.29 (-0.36;0.94) | 4.87E-01 | 0.39 (-0.28;1.05) | 3.53E-01 | 0.44 (-0.23;1.10) | 2.89E-01 |
| X-24307 | -0.55 (-1.21;0.10) | 1.63E-01 | -0.49 (-1.15;0.17) | 2.20E-01 | -0.43 (-1.10;0.23) | 2.90E-01 |
| Sphingomyelin (D18:1/20:2, D18:2/20:1, D16:1/22:2)* | -0.65 (-1.35;0.05) | 1.17E-01 | -0.55 (-1.27;0.16) | 1.99E-01 | -0.47 (-1.19;0.25) | 2.93E-01 |
| 2-Hydroxynervonate* | 0.48 (-0.17;1.14) | 2.23E-01 | 0.48 (-0.18;1.15) | 2.37E-01 | 0.44 (-0.23;1.10) | 2.93E-01 |
| X-11308 | 0.29 (-0.38;0.95) | 5.04E-01 | 0.45 (-0.23;1.14) | 2.79E-01 | 0.44 (-0.24;1.12) | 2.93E-01 |
| Oleoyl-Linoleoyl-Glycerol (18:1/18:2) [1] | -0.62 (-1.27;0.03) | 1.09E-01 | -0.45 (-1.13;0.22) | 2.75E-01 | -0.44 (-1.11;0.24) | 2.94E-01 |
| N-Acetylasparagine | -0.36 (-1.01;0.29) | 3.78E-01 | -0.40 (-1.06;0.26) | 3.27E-01 | -0.42 (-1.08;0.23) | 2.98E-01 |
| Tricosanoyl Sphingomyelin (D18:1/23:0)* | 0.19 (-0.50;0.88) | 6.72E-01 | 0.44 (-0.34;1.21) | 3.66E-01 | 0.50 (-0.28;1.28) | 2.99E-01 |
| Sphingomyelin (D18:2/14:0, D18:1/14:1)* | -0.73 (-1.50;0.05) | 1.15E-01 | -0.64 (-1.50;0.21) | 2.19E-01 | -0.56 (-1.43;0.31) | 3.02E-01 |
| Andro Steroid Monosulfate C19H28O6S (1)* | -0.37 (-1.05;0.31) | 3.84E-01 | -0.41 (-1.10;0.28) | 3.41E-01 | -0.44 (-1.13;0.25) | 3.07E-01 |
| Sarcosine | -0.43 (-1.09;0.22) | 2.85E-01 | -0.46 (-1.12;0.20) | 2.54E-01 | -0.42 (-1.08;0.24) | 3.07E-01 |
| Palmitoylcholine | -0.43 (-1.64;0.78) | 5.80E-01 | -0.68 (-1.98;0.63) | 4.10E-01 | -0.86 (-2.22;0.50) | 3.09E-01 |
| Bilirubin (Z,Z) | 0.28 (-0.38;0.94) | 5.11E-01 | 0.36 (-0.31;1.04) | 3.85E-01 | 0.42 (-0.25;1.10) | 3.11E-01 |
| Linoleoyl Ethanolamide | 0.40 (-0.26;1.05) | 3.27E-01 | 0.43 (-0.24;1.09) | 2.96E-01 | 0.42 (-0.25;1.08) | 3.13E-01 |
| Glycochenodeoxycholate 3-Sulfate | -0.41 (-1.08;0.25) | 3.19E-01 | -0.43 (-1.11;0.24) | 2.97E-01 | -0.42 (-1.10;0.25) | 3.14E-01 |
| Sphingomyelin (D18:1/25:0, D19:0/24:1, D20:1/23:0, D19:1/24:0)* | 0.23 (-0.42;0.89) | 5.86E-01 | 0.35 (-0.34;1.04) | 4.22E-01 | 0.43 (-0.26;1.13) | 3.20E-01 |
| Myristoyl Dihydrosphingomyelin (D18:0/14:0)* | 0.31 (-0.37;0.98) | 4.76E-01 | 0.37 (-0.36;1.09) | 4.23E-01 | 0.45 (-0.28;1.18) | 3.21E-01 |
| Alpha-Hydroxyisovalerate | -0.27 (-0.95;0.41) | 5.40E-01 | -0.38 (-1.08;0.32) | 3.89E-01 | -0.43 (-1.13;0.27) | 3.23E-01 |
| X-24949 | 0.44 (-0.21;1.10) | 2.74E-01 | 0.45 (-0.21;1.12) | 2.69E-01 | 0.41 (-0.26;1.08) | 3.23E-01 |
| 1-Lignoceroyl-GPC (24:0) | 0.25 (-0.40;0.91) | 5.48E-01 | 0.42 (-0.35;1.20) | 3.82E-01 | 0.48 (-0.30;1.25) | 3.23E-01 |
| 16-Hydroxypalmitate | 0.56 (-0.10;1.23) | 1.57E-01 | 0.45 (-0.23;1.12) | 2.81E-01 | 0.41 (-0.26;1.09) | 3.23E-01 |
| X-12847 | -0.35 (-1.01;0.32) | 4.13E-01 | -0.42 (-1.10;0.26) | 3.22E-01 | -0.42 (-1.10;0.26) | 3.23E-01 |
| X-25419 | -0.41 (-1.08;0.25) | 3.14E-01 | -0.47 (-1.14;0.21) | 2.58E-01 | -0.41 (-1.09;0.26) | 3.26E-01 |
| Ceramide (D18:1/14:0, D16:1/16:0)* | -0.48 (-1.14;0.18) | 2.30E-01 | -0.45 (-1.13;0.22) | 2.78E-01 | -0.41 (-1.09;0.26) | 3.26E-01 |
| Serotonin | 0.38 (-0.39;1.16) | 4.35E-01 | 0.46 (-0.33;1.24) | 3.51E-01 | 0.48 (-0.30;1.26) | 3.26E-01 |
| X-21339 | 0.21 (-0.45;0.87) | 6.26E-01 | 0.42 (-0.26;1.09) | 3.22E-01 | 0.41 (-0.26;1.09) | 3.26E-01 |
| N1-Methyl-4-Pyridone-3-Carboxamide | -0.60 (-1.38;0.18) | 2.01E-01 | -0.51 (-1.30;0.27) | 2.89E-01 | -0.48 (-1.26;0.31) | 3.27E-01 |
| Pregnenediol Disulfate (C21H34O8S2)* | -0.39 (-1.15;0.38) | 4.24E-01 | -0.44 (-1.22;0.34) | 3.63E-01 | -0.47 (-1.25;0.30) | 3.27E-01 |
| X-11483 | -0.44 (-1.09;0.21) | 2.74E-01 | -0.44 (-1.09;0.22) | 2.80E-01 | -0.40 (-1.06;0.26) | 3.27E-01 |
| 1-Palmitoyl-2-Dihomo-Linolenoyl-GPC (16:0/20:3n3 Or 6)* | 0.28 (-0.39;0.94) | 5.18E-01 | 0.37 (-0.31;1.05) | 3.89E-01 | 0.41 (-0.27;1.10) | 3.29E-01 |
| S-Methylmethionine | -0.62 (-1.83;0.59) | 4.17E-01 | -0.72 (-1.94;0.49) | 3.41E-01 | -0.74 (-1.96;0.48) | 3.30E-01 |
| 1-Oleoyl-GPG (18:1)* | -0.95 (-2.16;0.26) | 1.95E-01 | -0.73 (-1.98;0.51) | 3.43E-01 | -0.75 (-1.99;0.50) | 3.34E-01 |
| X-21319 | -0.35 (-1.00;0.30) | 3.96E-01 | -0.42 (-1.08;0.23) | 2.96E-01 | -0.39 (-1.05;0.26) | 3.37E-01 |
| 1-(1-Enyl-Palmitoyl)-2-Oleoyl-GPE (P-16:0/18:1)* | 0.37 (-0.29;1.02) | 3.73E-01 | 0.37 (-0.29;1.03) | 3.69E-01 | 0.39 (-0.27;1.05) | 3.42E-01 |
| Histidine Betaine (Hercynine)* | -0.81 (-2.01;0.39) | 2.73E-01 | -0.74 (-1.95;0.48) | 3.31E-01 | -0.72 (-1.94;0.50) | 3.45E-01 |
| Behenoylcarnitine (C22)* | 0.69 (-0.51;1.89) | 3.62E-01 | 0.71 (-0.51;1.93) | 3.53E-01 | 0.72 (-0.51;1.95) | 3.47E-01 |
| 2-Palmitoyl-GPC (16:0)* | 0.26 (-0.39;0.91) | 5.35E-01 | 0.40 (-0.30;1.09) | 3.60E-01 | 0.41 (-0.29;1.10) | 3.49E-01 |
| Alpha-Ketoglutarate | -0.43 (-1.08;0.22) | 2.89E-01 | -0.39 (-1.06;0.27) | 3.41E-01 | -0.39 (-1.05;0.28) | 3.49E-01 |
| X-11381 | -0.56 (-1.21;0.10) | 1.60E-01 | -0.47 (-1.13;0.19) | 2.45E-01 | -0.39 (-1.05;0.28) | 3.49E-01 |
| X-21959 | 0.61 (-0.59;1.82) | 4.22E-01 | 0.71 (-0.49;1.91) | 3.41E-01 | 0.70 (-0.50;1.90) | 3.49E-01 |
| X-17438 | -0.30 (-0.96;0.36) | 4.81E-01 | -0.38 (-1.05;0.30) | 3.72E-01 | -0.39 (-1.06;0.28) | 3.50E-01 |
| 13-Hode + 9-Hode | 0.42 (-0.24;1.07) | 3.05E-01 | 0.43 (-0.24;1.09) | 2.96E-01 | 0.39 (-0.28;1.05) | 3.52E-01 |
| 1-Stearoyl-GPI (18:0) | 0.22 (-0.45;0.88) | 6.17E-01 | 0.37 (-0.33;1.07) | 3.99E-01 | 0.41 (-0.30;1.11) | 3.52E-01 |
| X-21442 | -0.39 (-1.04;0.27) | 3.44E-01 | -0.43 (-1.09;0.23) | 2.89E-01 | -0.38 (-1.04;0.28) | 3.54E-01 |
| Ceramide (D18:1/17:0, D17:1/18:0)* | -0.62 (-1.29;0.05) | 1.20E-01 | -0.46 (-1.16;0.25) | 2.94E-01 | -0.41 (-1.12;0.30) | 3.57E-01 |
| Phenylacetate | -0.49 (-1.15;0.16) | 2.15E-01 | -0.40 (-1.08;0.27) | 3.38E-01 | -0.39 (-1.06;0.29) | 3.59E-01 |
| Oleoyl-Linoleoyl-Glycerol (18:1/18:2) [2] | -0.58 (-1.23;0.07) | 1.37E-01 | -0.39 (-1.07;0.29) | 3.56E-01 | -0.39 (-1.07;0.29) | 3.63E-01 |
| Ceramide (D16:1/24:1, D18:1/22:1)* | -0.51 (-1.16;0.15) | 1.98E-01 | -0.42 (-1.08;0.25) | 3.15E-01 | -0.38 (-1.05;0.29) | 3.63E-01 |
| X-23276 | 0.57 (-0.64;1.78) | 4.59E-01 | 0.68 (-0.52;1.88) | 3.63E-01 | 0.68 (-0.52;1.89) | 3.63E-01 |
| Phytanate | 0.40 (-0.26;1.05) | 3.37E-01 | 0.37 (-0.30;1.04) | 3.73E-01 | 0.38 (-0.29;1.05) | 3.63E-01 |
| X-24951 | 0.66 (-0.55;1.87) | 3.88E-01 | 0.68 (-0.54;1.91) | 3.72E-01 | 0.70 (-0.53;1.93) | 3.64E-01 |
| Bilirubin Degradation Product, C17H20N2O5 (2)** | 0.84 (-0.37;2.05) | 2.62E-01 | 0.71 (-0.51;1.93) | 3.50E-01 | 0.69 (-0.54;1.92) | 3.68E-01 |
| Hexadecanedioate (C16-DC) | -0.24 (-0.92;0.43) | 5.80E-01 | -0.37 (-1.05;0.31) | 3.90E-01 | -0.38 (-1.06;0.30) | 3.71E-01 |
| Stearoylcholine* | -0.44 (-1.65;0.77) | 5.77E-01 | -0.58 (-1.88;0.71) | 4.76E-01 | -0.75 (-2.11;0.60) | 3.75E-01 |
| 2-Hydroxydecanoate | 0.36 (-0.30;1.02) | 3.88E-01 | 0.33 (-0.33;0.99) | 4.27E-01 | 0.37 (-0.29;1.03) | 3.76E-01 |
| Threonine | 0.34 (-0.31;1.00) | 4.09E-01 | 0.35 (-0.31;1.01) | 3.92E-01 | 0.37 (-0.29;1.03) | 3.76E-01 |
| 3-Hydroxyoleoylcarnitine | -0.33 (-1.01;0.34) | 4.35E-01 | -0.36 (-1.04;0.32) | 3.99E-01 | -0.37 (-1.05;0.31) | 3.83E-01 |
| Bilirubin Degradation Product, C17H20N2O5 (1)** | 0.82 (-0.39;2.03) | 2.74E-01 | 0.69 (-0.53;1.90) | 3.66E-01 | 0.67 (-0.56;1.89) | 3.88E-01 |
| Adenosine 5'-Monophosphate (AMP) | 0.31 (-0.34;0.97) | 4.54E-01 | 0.37 (-0.29;1.03) | 3.65E-01 | 0.36 (-0.30;1.02) | 3.88E-01 |
| X-21258 | -0.31 (-0.97;0.35) | 4.57E-01 | -0.37 (-1.03;0.29) | 3.69E-01 | -0.36 (-1.02;0.30) | 3.91E-01 |
| 2-Hydroxypalmitate | 0.27 (-0.39;0.92) | 5.24E-01 | 0.37 (-0.30;1.05) | 3.73E-01 | 0.36 (-0.31;1.03) | 3.93E-01 |
| 5alpha-Pregnan-3beta,20beta-Diol Monosulfate (1) | -0.38 (-1.04;0.28) | 3.64E-01 | -0.33 (-0.99;0.33) | 4.30E-01 | -0.36 (-1.02;0.31) | 3.93E-01 |
| S-Methylcysteine | 0.34 (-0.31;0.99) | 4.13E-01 | 0.32 (-0.34;0.98) | 4.36E-01 | 0.35 (-0.31;1.02) | 3.96E-01 |
| 1-Palmitoyl-2-Docosahexaenoyl-GPC (16:0/22:6) | 0.19 (-0.46;0.85) | 6.51E-01 | 0.33 (-0.35;1.02) | 4.35E-01 | 0.36 (-0.32;1.05) | 3.96E-01 |
| X-23665 | -0.35 (-1.01;0.30) | 3.96E-01 | -0.36 (-1.02;0.31) | 3.92E-01 | -0.36 (-1.02;0.31) | 3.97E-01 |
| Guanidinoacetate | 0.31 (-0.36;0.99) | 4.68E-01 | 0.29 (-0.40;0.98) | 5.07E-01 | 0.37 (-0.33;1.07) | 3.97E-01 |
| Sphingomyelin (D18:1/14:0, D16:1/16:0)* | -0.44 (-1.11;0.23) | 2.91E-01 | -0.43 (-1.16;0.29) | 3.41E-01 | -0.39 (-1.12;0.34) | 3.99E-01 |
| Glycosyl-N-Palmitoyl-Sphingosine (D18:1/16:0) | 0.29 (-0.37;0.94) | 4.93E-01 | 0.30 (-0.41;1.02) | 5.05E-01 | 0.38 (-0.34;1.10) | 3.99E-01 |
| X-22771 | 0.35 (-0.43;1.12) | 4.84E-01 | 0.38 (-0.40;1.16) | 4.41E-01 | 0.41 (-0.37;1.20) | 4.01E-01 |
| Androstenediol (3alpha, 17alpha) Monosulfate (3) | -0.39 (-1.14;0.36) | 4.15E-01 | -0.37 (-1.12;0.39) | 4.41E-01 | -0.40 (-1.16;0.36) | 4.04E-01 |
| N-Palmitoyl-Heptadecasphingosine (D17:1/16:0)* | -0.52 (-1.19;0.14) | 1.90E-01 | -0.42 (-1.12;0.28) | 3.39E-01 | -0.37 (-1.07;0.33) | 4.04E-01 |
| Hexadecenedioate (C16:1-DC)* | -0.26 (-0.92;0.41) | 5.51E-01 | -0.35 (-1.02;0.32) | 4.02E-01 | -0.35 (-1.02;0.32) | 4.04E-01 |
| Pentadecanoate (15:0) | 0.38 (-0.29;1.06) | 3.67E-01 | 0.39 (-0.29;1.07) | 3.62E-01 | 0.36 (-0.33;1.04) | 4.05E-01 |
| Stearoyl-Arachidonoyl-Glycerol (18:0/20:4) [2]* | 0.38 (-0.40;1.16) | 4.40E-01 | 0.46 (-0.34;1.26) | 3.55E-01 | 0.42 (-0.38;1.22) | 4.05E-01 |
| Branched-Chain, Straight-Chain, Or Cyclopropyl 10:1 Fatty Acid (1)* | 0.49 (-0.17;1.15) | 2.25E-01 | 0.36 (-0.32;1.03) | 3.99E-01 | 0.36 (-0.32;1.04) | 4.05E-01 |
| Dihomo-Linolenoyl-Choline | -0.40 (-1.61;0.82) | 6.19E-01 | -0.52 (-1.78;0.74) | 5.18E-01 | -0.69 (-2.02;0.63) | 4.05E-01 |
| Isovalerate (I5:0) | 0.35 (-0.31;1.02) | 3.99E-01 | 0.33 (-0.34;0.99) | 4.35E-01 | 0.35 (-0.32;1.02) | 4.06E-01 |
| 1-Myristoyl-2-Palmitoyl-GPC (14:0/16:0) | 0.35 (-0.32;1.01) | 4.09E-01 | 0.34 (-0.34;1.02) | 4.22E-01 | 0.35 (-0.33;1.03) | 4.06E-01 |
| Sphingomyelin (D18:2/23:1)* | -0.58 (-1.30;0.14) | 1.86E-01 | -0.49 (-1.27;0.28) | 3.04E-01 | -0.41 (-1.20;0.38) | 4.08E-01 |
| 6-Bromotryptophan | 0.18 (-0.47;0.84) | 6.69E-01 | 0.24 (-0.42;0.90) | 5.62E-01 | 0.34 (-0.32;1.01) | 4.08E-01 |
| Palmitoyl-Oleoyl-Glycerol (16:0/18:1) [1]* | 0.35 (-0.43;1.13) | 4.84E-01 | 0.42 (-0.42;1.26) | 4.27E-01 | 0.44 (-0.41;1.28) | 4.09E-01 |
| Linoleoyl-Linoleoyl-Glycerol (18:2/18:2) [2]* | 0.35 (-0.43;1.12) | 4.86E-01 | 0.40 (-0.38;1.19) | 4.12E-01 | 0.40 (-0.38;1.19) | 4.11E-01 |
| Linoleoylcholine* | -0.25 (-1.45;0.96) | 7.69E-01 | -0.54 (-1.87;0.79) | 5.21E-01 | -0.71 (-2.10;0.67) | 4.12E-01 |
| X-21410 | -0.67 (-1.93;0.60) | 4.05E-01 | -0.68 (-1.97;0.61) | 4.00E-01 | -0.67 (-1.96;0.63) | 4.12E-01 |
| 1-Palmitoyl-2-Oleoyl-GPI (16:0/18:1)* | -0.28 (-0.94;0.38) | 5.12E-01 | -0.40 (-1.09;0.30) | 3.62E-01 | -0.35 (-1.05;0.34) | 4.14E-01 |
| Linoleoylcarnitine (C18:2)* | -0.50 (-1.16;0.17) | 2.19E-01 | -0.38 (-1.05;0.30) | 3.72E-01 | -0.35 (-1.02;0.33) | 4.15E-01 |
| 12,13-Dihome | 0.27 (-0.39;0.93) | 5.28E-01 | 0.35 (-0.33;1.02) | 4.12E-01 | 0.35 (-0.33;1.02) | 4.15E-01 |
| 3-Bromo-5-Chloro-2,6-Dihydroxybenzoic Acid* | 0.25 (-0.40;0.90) | 5.49E-01 | 0.29 (-0.37;0.95) | 4.84E-01 | 0.33 (-0.33;0.99) | 4.21E-01 |
| 1-Palmitoyl-GPC (16:0) | 0.20 (-0.45;0.85) | 6.35E-01 | 0.31 (-0.39;1.00) | 4.84E-01 | 0.35 (-0.35;1.05) | 4.23E-01 |
| Methyl-4-Hydroxybenzoate Sulfate | -0.52 (-1.78;0.74) | 5.23E-01 | -0.65 (-1.93;0.62) | 4.15E-01 | -0.64 (-1.92;0.64) | 4.25E-01 |
| Carnitine | -0.42 (-1.07;0.24) | 3.03E-01 | -0.39 (-1.05;0.26) | 3.40E-01 | -0.33 (-0.99;0.33) | 4.25E-01 |
| Palmitoyl-Docosahexaenoyl-Glycerol (16:0/22:6) [1]* | 0.38 (-0.40;1.16) | 4.42E-01 | 0.43 (-0.38;1.24) | 3.99E-01 | 0.41 (-0.41;1.22) | 4.26E-01 |
| Cortisol | -0.30 (-0.96;0.36) | 4.84E-01 | -0.34 (-1.00;0.33) | 4.19E-01 | -0.33 (-1.00;0.33) | 4.26E-01 |
| Trans-3,4-Methyleneheptanoate | -0.36 (-1.56;0.85) | 6.53E-01 | -0.58 (-1.79;0.64) | 4.50E-01 | -0.61 (-1.83;0.61) | 4.27E-01 |
| X-25343 | 0.27 (-0.39;0.92) | 5.24E-01 | 0.30 (-0.36;0.96) | 4.78E-01 | 0.33 (-0.33;0.99) | 4.27E-01 |
| 1-(1-Enyl-Oleoyl)-GPE (P-18:1)* | 0.18 (-0.48;0.84) | 6.71E-01 | 0.31 (-0.38;1.00) | 4.76E-01 | 0.34 (-0.35;1.04) | 4.27E-01 |
| Cholate | -0.46 (-1.68;0.76) | 5.57E-01 | -0.58 (-1.79;0.64) | 4.52E-01 | -0.60 (-1.82;0.62) | 4.31E-01 |
| 1-Stearoyl-2-Arachidonoyl-GPI (18:0/20:4) | 0.16 (-0.50;0.83) | 7.13E-01 | 0.31 (-0.39;1.01) | 4.80E-01 | 0.34 (-0.35;1.04) | 4.31E-01 |
| Eicosenedioate (C20:1-DC)* | 0.17 (-0.49;0.83) | 7.01E-01 | 0.28 (-0.39;0.95) | 5.10E-01 | 0.32 (-0.34;0.99) | 4.40E-01 |
| Alanine | -0.40 (-1.06;0.25) | 3.19E-01 | -0.32 (-0.98;0.34) | 4.46E-01 | -0.32 (-0.98;0.34) | 4.40E-01 |
| Carotene Diol (2) | -0.37 (-1.03;0.29) | 3.73E-01 | -0.36 (-1.06;0.34) | 4.15E-01 | -0.34 (-1.04;0.36) | 4.42E-01 |
| N-Palmitoyl-Sphingadienine (D18:2/16:0)* | -0.42 (-1.09;0.24) | 3.03E-01 | -0.39 (-1.07;0.29) | 3.62E-01 | -0.33 (-1.01;0.36) | 4.48E-01 |
| N2-Acetyl,N6-Methyllysine | -0.31 (-0.96;0.34) | 4.59E-01 | -0.33 (-0.98;0.33) | 4.25E-01 | -0.31 (-0.97;0.34) | 4.50E-01 |
| Malonylcarnitine | -0.41 (-1.62;0.79) | 5.97E-01 | -0.57 (-1.77;0.63) | 4.52E-01 | -0.57 (-1.78;0.63) | 4.50E-01 |
| Fructosyllysine | -0.31 (-0.96;0.35) | 4.64E-01 | -0.27 (-0.94;0.41) | 5.32E-01 | -0.33 (-1.02;0.36) | 4.51E-01 |
| X-24546 | -0.27 (-0.97;0.44) | 5.62E-01 | -0.35 (-1.08;0.37) | 4.37E-01 | -0.34 (-1.07;0.38) | 4.51E-01 |
| X-11847 | -0.39 (-1.05;0.26) | 3.36E-01 | -0.34 (-1.00;0.32) | 4.07E-01 | -0.31 (-0.97;0.35) | 4.56E-01 |
| Glutarate (C5-DC) | -0.30 (-0.95;0.36) | 4.80E-01 | -0.30 (-0.96;0.36) | 4.69E-01 | -0.31 (-0.96;0.35) | 4.61E-01 |
| N-Palmitoylglycine | -0.27 (-0.93;0.39) | 5.33E-01 | -0.29 (-0.95;0.38) | 4.95E-01 | -0.31 (-0.98;0.36) | 4.64E-01 |
| 1-Palmitoylglycerol (16:0) | -0.60 (-1.80;0.61) | 4.35E-01 | -0.56 (-1.76;0.64) | 4.60E-01 | -0.55 (-1.76;0.65) | 4.65E-01 |
| 1-(1-Enyl-Palmitoyl)-GPE (P-16:0)* | 0.19 (-0.46;0.84) | 6.58E-01 | 0.28 (-0.40;0.96) | 5.21E-01 | 0.31 (-0.37;1.00) | 4.66E-01 |
| 2-Hydroxystearate | 0.22 (-0.44;0.88) | 6.07E-01 | 0.36 (-0.31;1.03) | 3.92E-01 | 0.31 (-0.36;0.98) | 4.67E-01 |
| Glutamine | 0.27 (-0.39;0.92) | 5.28E-01 | 0.27 (-0.40;0.93) | 5.30E-01 | 0.31 (-0.36;0.98) | 4.67E-01 |
| 5,6-Dihydrothymine | -0.42 (-1.07;0.24) | 3.10E-01 | -0.38 (-1.04;0.28) | 3.61E-01 | -0.30 (-0.97;0.36) | 4.73E-01 |
| Inosine | -0.48 (-1.68;0.72) | 5.36E-01 | -0.55 (-1.74;0.65) | 4.69E-01 | -0.55 (-1.75;0.66) | 4.73E-01 |
| X-11299 | -0.37 (-1.02;0.29) | 3.73E-01 | -0.34 (-1.00;0.32) | 4.12E-01 | -0.30 (-0.96;0.36) | 4.73E-01 |
| 1-(1-Enyl-Palmitoyl)-2-Linoleoyl-GPC (P-16:0/18:2)* | -0.28 (-0.94;0.38) | 5.04E-01 | -0.40 (-1.12;0.32) | 3.74E-01 | -0.33 (-1.05;0.40) | 4.75E-01 |
| 2-Linoleoylglycerol (18:2) | -0.79 (-2.00;0.42) | 2.91E-01 | -0.58 (-1.80;0.65) | 4.53E-01 | -0.55 (-1.78;0.68) | 4.77E-01 |
| Glycocholenate Sulfate* | 0.22 (-0.45;0.89) | 6.17E-01 | 0.24 (-0.44;0.92) | 5.75E-01 | 0.30 (-0.38;0.98) | 4.77E-01 |
| Sphingomyelin (D17:1/16:0, D18:1/15:0, D16:1/17:0)* | -0.42 (-1.09;0.25) | 3.14E-01 | -0.39 (-1.12;0.34) | 3.97E-01 | -0.33 (-1.07;0.41) | 4.77E-01 |
| Oleoylcholine | -0.25 (-1.46;0.97) | 7.69E-01 | -0.45 (-1.74;0.85) | 5.91E-01 | -0.61 (-1.95;0.74) | 4.77E-01 |
| (2 Or 3)-Decenoate (10:1n7 Or N8) | 0.33 (-0.33;0.98) | 4.27E-01 | 0.32 (-0.34;0.98) | 4.35E-01 | 0.30 (-0.36;0.96) | 4.77E-01 |
| Taurochenodeoxycholate | -0.25 (-0.90;0.40) | 5.50E-01 | -0.24 (-0.89;0.42) | 5.76E-01 | -0.30 (-0.96;0.36) | 4.77E-01 |
| X-26111 | 0.29 (-0.36;0.95) | 4.85E-01 | 0.28 (-0.38;0.94) | 5.04E-01 | 0.29 (-0.37;0.96) | 4.79E-01 |
| Sphingomyelin (D17:2/16:0, D18:2/15:0)* | -0.63 (-1.38;0.11) | 1.57E-01 | -0.47 (-1.31;0.37) | 3.73E-01 | -0.38 (-1.24;0.47) | 4.80E-01 |
| X-21733 | 0.10 (-0.56;0.76) | 8.32E-01 | 0.29 (-0.41;0.98) | 5.18E-01 | 0.31 (-0.39;1.01) | 4.80E-01 |
| X-19438 | 0.30 (-0.36;0.96) | 4.76E-01 | 0.31 (-0.36;0.97) | 4.63E-01 | 0.29 (-0.37;0.95) | 4.85E-01 |
| Hydroxypalmitoyl Sphingomyelin (D18:1/16:0(OH))** | -0.39 (-1.05;0.27) | 3.40E-01 | -0.36 (-1.08;0.35) | 4.20E-01 | -0.31 (-1.03;0.41) | 4.89E-01 |
| X-26108 | 0.30 (-0.38;0.97) | 4.90E-01 | 0.37 (-0.32;1.05) | 3.97E-01 | 0.30 (-0.39;0.99) | 4.89E-01 |
| Taurodeoxycholate | -0.24 (-0.89;0.41) | 5.67E-01 | -0.25 (-0.91;0.41) | 5.51E-01 | -0.29 (-0.95;0.37) | 4.90E-01 |
| 1-Palmitoyl-2-Palmitoleoyl-GPC (16:0/16:1)* | 0.37 (-0.30;1.04) | 3.82E-01 | 0.30 (-0.41;1.00) | 5.05E-01 | 0.30 (-0.40;1.01) | 4.95E-01 |
| N-Stearoyltaurine | -0.29 (-0.95;0.36) | 4.86E-01 | -0.26 (-0.93;0.41) | 5.46E-01 | -0.29 (-0.96;0.38) | 4.96E-01 |
| 3-Hydroxyisobutyrate | -0.27 (-0.93;0.38) | 5.19E-01 | -0.27 (-0.94;0.39) | 5.18E-01 | -0.29 (-0.96;0.38) | 4.96E-01 |
| Sphingomyelin (D18:1/24:1, D18:2/24:0)* | 0.21 (-0.46;0.87) | 6.32E-01 | 0.24 (-0.48;0.97) | 6.01E-01 | 0.31 (-0.42;1.04) | 4.98E-01 |
| Dodecadienoate (12:2)* | 0.24 (-0.42;0.89) | 5.72E-01 | 0.31 (-0.35;0.98) | 4.59E-01 | 0.28 (-0.38;0.95) | 4.99E-01 |
| 1-(1-Enyl-Stearoyl)-2-Arachidonoyl-GPE (P-18:0/20:4)* | 0.20 (-0.45;0.86) | 6.34E-01 | 0.28 (-0.39;0.95) | 5.10E-01 | 0.28 (-0.39;0.95) | 5.04E-01 |
| 3-Hydroxylaurate | 0.43 (-0.24;1.10) | 3.02E-01 | 0.35 (-0.33;1.03) | 4.14E-01 | 0.29 (-0.39;0.97) | 5.06E-01 |
| Sphingomyelin (D18:2/21:0, D16:2/23:0)* | -0.59 (-1.32;0.14) | 1.85E-01 | -0.41 (-1.19;0.38) | 4.12E-01 | -0.33 (-1.13;0.46) | 5.09E-01 |
| Oxindolylalanine | -0.48 (-1.68;0.73) | 5.41E-01 | -0.47 (-1.69;0.74) | 5.42E-01 | -0.51 (-1.73;0.71) | 5.09E-01 |
| 5alpha-Androstan-3alpha,17alpha-Diol Monosulfate | -0.45 (-1.78;0.89) | 6.07E-01 | -0.53 (-1.86;0.80) | 5.32E-01 | -0.56 (-1.89;0.78) | 5.10E-01 |
| 1-(1-Enyl-Stearoyl)-2-Oleoyl-GPE (P-18:0/18:1) | 0.26 (-0.40;0.91) | 5.45E-01 | 0.27 (-0.40;0.93) | 5.27E-01 | 0.28 (-0.39;0.94) | 5.10E-01 |
| 1-Linoleoyl-GPG (18:2)* | 0.16 (-0.49;0.82) | 7.09E-01 | 0.28 (-0.38;0.95) | 5.04E-01 | 0.28 (-0.39;0.95) | 5.10E-01 |
| Glucose | 0.27 (-0.38;0.93) | 5.21E-01 | 0.32 (-0.35;1.00) | 4.45E-01 | 0.30 (-0.41;1.00) | 5.10E-01 |
| X-21353 | 0.37 (-0.29;1.03) | 3.71E-01 | 0.34 (-0.32;1.01) | 4.11E-01 | 0.28 (-0.39;0.94) | 5.10E-01 |
| X-24295 | 0.37 (-0.86;1.59) | 6.47E-01 | 0.42 (-0.86;1.70) | 6.05E-01 | 0.55 (-0.77;1.86) | 5.10E-01 |
| 5-Hydroxylysine | 0.22 (-0.44;0.87) | 6.15E-01 | 0.28 (-0.40;0.95) | 5.16E-01 | 0.28 (-0.40;0.95) | 5.12E-01 |
| Campesterol | -0.32 (-0.97;0.33) | 4.42E-01 | -0.21 (-0.92;0.49) | 6.41E-01 | -0.29 (-1.00;0.42) | 5.16E-01 |
| Glycosyl-N-Behenoyl-Sphingadienine (D18:2/22:0)* | 0.08 (-0.58;0.74) | 8.58E-01 | 0.18 (-0.55;0.92) | 7.05E-01 | 0.30 (-0.44;1.05) | 5.16E-01 |
| Stearoylcarnitine (C18) | 0.11 (-0.56;0.77) | 8.17E-01 | 0.25 (-0.44;0.94) | 5.75E-01 | 0.28 (-0.41;0.97) | 5.18E-01 |
| X-14056 | -0.48 (-1.69;0.74) | 5.41E-01 | -0.55 (-1.80;0.70) | 4.85E-01 | -0.51 (-1.77;0.75) | 5.18E-01 |
| Palmitoleoyl-Linoleoyl-Glycerol (16:1/18:2) [1]* | -0.35 (-1.13;0.42) | 4.76E-01 | -0.34 (-1.15;0.47) | 5.10E-01 | -0.32 (-1.14;0.49) | 5.32E-01 |
| Palmitoleoylcarnitine (C16:1)* | -0.27 (-0.94;0.41) | 5.40E-01 | -0.31 (-0.99;0.37) | 4.71E-01 | -0.27 (-0.95;0.41) | 5.36E-01 |
| Dehydroepiandrosterone Sulfate (DHEA-S) | -0.16 (-0.87;0.55) | 7.44E-01 | -0.25 (-0.98;0.47) | 5.85E-01 | -0.28 (-1.01;0.44) | 5.39E-01 |
| Taurocholate | -0.23 (-0.88;0.42) | 5.87E-01 | -0.21 (-0.86;0.45) | 6.24E-01 | -0.26 (-0.92;0.40) | 5.43E-01 |
| Beta-Sitosterol | -0.40 (-1.18;0.38) | 4.17E-01 | -0.25 (-1.09;0.59) | 6.39E-01 | -0.32 (-1.16;0.52) | 5.44E-01 |
| Methionine | -0.27 (-0.94;0.41) | 5.40E-01 | -0.26 (-0.94;0.41) | 5.42E-01 | -0.26 (-0.93;0.42) | 5.47E-01 |
| X-11478 | -0.22 (-0.88;0.43) | 5.97E-01 | -0.26 (-0.92;0.40) | 5.33E-01 | -0.25 (-0.91;0.41) | 5.48E-01 |
| Sphingomyelin (D18:2/16:0, D18:1/16:1)* | -0.42 (-1.10;0.27) | 3.26E-01 | -0.35 (-1.08;0.38) | 4.49E-01 | -0.28 (-1.02;0.46) | 5.51E-01 |
| 1-Stearoyl-2-Linoleoyl-GPC (18:0/18:2)* | 0.17 (-0.49;0.84) | 6.95E-01 | 0.22 (-0.49;0.94) | 6.26E-01 | 0.27 (-0.44;0.99) | 5.51E-01 |
| 1-Stearoyl-2-Linoleoyl-GPI (18:0/18:2) | -0.33 (-0.98;0.33) | 4.32E-01 | -0.30 (-0.99;0.40) | 5.03E-01 | -0.26 (-0.96;0.43) | 5.51E-01 |
| Stearoyl-Arachidonoyl-Glycerol (18:0/20:4) [1]* | 0.25 (-0.53;1.03) | 6.19E-01 | 0.32 (-0.49;1.13) | 5.34E-01 | 0.31 (-0.50;1.11) | 5.51E-01 |
| 9,10-Dihome | 0.20 (-0.45;0.85) | 6.39E-01 | 0.25 (-0.41;0.91) | 5.56E-01 | 0.25 (-0.41;0.92) | 5.51E-01 |
| Glycolithocholate Sulfate* | -0.24 (-0.89;0.42) | 5.78E-01 | -0.23 (-0.89;0.43) | 5.91E-01 | -0.25 (-0.91;0.41) | 5.51E-01 |
| Glucuronide Of Piperine Metabolite C17H21NO3 (3)* | -0.33 (-1.55;0.90) | 6.86E-01 | -0.48 (-1.74;0.78) | 5.46E-01 | -0.46 (-1.73;0.80) | 5.69E-01 |
| Sphingomyelin (D18:1/22:1, D18:2/22:0, D16:1/24:1)* | -0.36 (-1.04;0.31) | 3.96E-01 | -0.30 (-1.02;0.41) | 5.05E-01 | -0.26 (-0.98;0.46) | 5.72E-01 |
| 3beta-Hydroxy-5-Cholestenoate | -0.21 (-0.86;0.45) | 6.27E-01 | -0.30 (-1.00;0.40) | 4.99E-01 | -0.25 (-0.95;0.45) | 5.73E-01 |
| Mannose | 0.23 (-0.43;0.89) | 5.87E-01 | 0.27 (-0.41;0.96) | 5.30E-01 | 0.25 (-0.45;0.95) | 5.76E-01 |
| N-Stearoyl-Sphingadienine (D18:2/18:0)* | -0.44 (-1.12;0.23) | 2.91E-01 | -0.33 (-1.05;0.38) | 4.63E-01 | -0.26 (-0.98;0.46) | 5.76E-01 |
| Dihomo-Linolenoylcarnitine (C20:3n3 Or 6)* | -0.39 (-1.06;0.28) | 3.55E-01 | -0.27 (-0.95;0.41) | 5.32E-01 | -0.24 (-0.92;0.44) | 5.79E-01 |
| 4-Hydroxyphenylpyruvate | -0.58 (-1.78;0.62) | 4.50E-01 | -0.46 (-1.67;0.75) | 5.48E-01 | -0.44 (-1.66;0.79) | 5.79E-01 |
| Asparagine | 0.25 (-0.40;0.91) | 5.51E-01 | 0.23 (-0.44;0.90) | 5.94E-01 | 0.24 (-0.43;0.91) | 5.79E-01 |
| X-12851 | -0.21 (-0.87;0.46) | 6.35E-01 | -0.21 (-0.88;0.46) | 6.22E-01 | -0.24 (-0.90;0.43) | 5.79E-01 |
| Threonate | 0.19 (-0.47;0.85) | 6.55E-01 | 0.25 (-0.42;0.92) | 5.64E-01 | 0.24 (-0.43;0.91) | 5.81E-01 |
| Octadecadienedioate (C18:2-DC)* | 0.19 (-0.47;0.84) | 6.58E-01 | 0.22 (-0.44;0.88) | 6.01E-01 | 0.23 (-0.43;0.89) | 5.82E-01 |
| Tartarate | -0.25 (-0.90;0.41) | 5.54E-01 | -0.23 (-0.88;0.43) | 5.95E-01 | -0.23 (-0.89;0.43) | 5.91E-01 |
| Laurate (12:0) | 0.31 (-0.36;0.99) | 4.69E-01 | 0.27 (-0.41;0.96) | 5.32E-01 | 0.24 (-0.45;0.93) | 5.92E-01 |
| Hexanoylglycine | -0.04 (-1.34;1.25) | 9.66E-01 | -0.39 (-1.72;0.94) | 6.48E-01 | -0.46 (-1.81;0.89) | 5.94E-01 |
| Ornithine | 0.14 (-0.52;0.79) | 7.64E-01 | 0.21 (-0.45;0.87) | 6.18E-01 | 0.22 (-0.44;0.88) | 6.03E-01 |
| Glycocholate | 0.24 (-0.41;0.90) | 5.65E-01 | 0.25 (-0.41;0.91) | 5.44E-01 | 0.22 (-0.44;0.88) | 6.06E-01 |
| Gamma-Glutamylalanine | -0.42 (-1.63;0.79) | 5.91E-01 | -0.38 (-1.59;0.84) | 6.31E-01 | -0.41 (-1.63;0.81) | 6.07E-01 |
| 1-Palmitoyl-2-Linoleoyl-GPC (16:0/18:2) | -0.20 (-0.85;0.46) | 6.42E-01 | -0.28 (-0.97;0.42) | 5.32E-01 | -0.23 (-0.93;0.47) | 6.09E-01 |
| N-Stearoyl-Sphingosine (D18:1/18:0)* | -0.38 (-1.04;0.29) | 3.67E-01 | -0.29 (-0.99;0.41) | 5.18E-01 | -0.23 (-0.94;0.47) | 6.10E-01 |
| Perfluorooctanesulfonate (PFOS) | 0.14 (-0.53;0.80) | 7.69E-01 | 0.20 (-0.48;0.88) | 6.43E-01 | 0.22 (-0.46;0.90) | 6.13E-01 |
| Branched-Chain, Straight-Chain, Or Cyclopropyl 12:1 Fatty Acid* | 0.25 (-0.42;0.92) | 5.60E-01 | 0.24 (-0.44;0.92) | 5.78E-01 | 0.22 (-0.46;0.90) | 6.13E-01 |
| S-Methylcysteine Sulfoxide | -0.23 (-0.88;0.42) | 5.83E-01 | -0.22 (-0.88;0.44) | 6.04E-01 | -0.21 (-0.87;0.45) | 6.21E-01 |
| Sphingomyelin (D18:1/21:0, D17:1/22:0, D16:1/23:0)* | -0.41 (-1.10;0.28) | 3.40E-01 | -0.28 (-1.05;0.48) | 5.61E-01 | -0.24 (-1.01;0.52) | 6.25E-01 |
| Octadecanedioate (C18-DC) | -0.15 (-0.82;0.52) | 7.44E-01 | -0.21 (-0.88;0.47) | 6.33E-01 | -0.21 (-0.89;0.46) | 6.28E-01 |
| Methionine Sulfoxide | -0.28 (-0.93;0.37) | 5.03E-01 | -0.19 (-0.86;0.47) | 6.48E-01 | -0.21 (-0.88;0.46) | 6.30E-01 |
| N6-Methyllysine | -0.18 (-0.83;0.48) | 6.84E-01 | -0.21 (-0.86;0.45) | 6.21E-01 | -0.21 (-0.86;0.45) | 6.30E-01 |
| 5-Oxoproline | -0.28 (-0.93;0.38) | 5.11E-01 | -0.25 (-0.90;0.41) | 5.57E-01 | -0.21 (-0.86;0.45) | 6.30E-01 |
| 1-Stearoyl-GPE (18:0) | -0.35 (-1.01;0.32) | 4.09E-01 | -0.23 (-0.93;0.48) | 6.17E-01 | -0.22 (-0.92;0.48) | 6.30E-01 |
| Sphingomyelin (D18:1/18:1, D18:2/18:0) | -0.47 (-1.18;0.23) | 2.79E-01 | -0.33 (-1.07;0.41) | 4.85E-01 | -0.23 (-0.98;0.52) | 6.35E-01 |
| Glycohyocholate | -0.13 (-0.79;0.52) | 7.69E-01 | -0.25 (-0.92;0.43) | 5.66E-01 | -0.21 (-0.88;0.47) | 6.35E-01 |
| 1-(1-Enyl-Palmitoyl)-2-Arachidonoyl-GPE (P-16:0/20:4)* | 0.13 (-0.52;0.78) | 7.70E-01 | 0.18 (-0.48;0.84) | 6.74E-01 | 0.20 (-0.46;0.87) | 6.35E-01 |
| Eicosenoylcarnitine (C20:1)* | 0.09 (-0.58;0.76) | 8.46E-01 | 0.18 (-0.51;0.86) | 6.96E-01 | 0.21 (-0.48;0.90) | 6.35E-01 |
| Pristanate | 0.30 (-0.36;0.96) | 4.76E-01 | 0.23 (-0.44;0.90) | 5.98E-01 | 0.20 (-0.46;0.87) | 6.35E-01 |
| X-11315 | 0.10 (-0.56;0.75) | 8.32E-01 | 0.10 (-0.57;0.76) | 8.32E-01 | 0.21 (-0.47;0.89) | 6.35E-01 |
| X-24241 | 0.17 (-0.60;0.95) | 7.44E-01 | 0.21 (-0.56;0.99) | 6.72E-01 | 0.24 (-0.54;1.02) | 6.35E-01 |
| X-24953 | 0.34 (-0.87;1.55) | 6.66E-01 | 0.37 (-0.87;1.61) | 6.42E-01 | 0.38 (-0.86;1.62) | 6.35E-01 |
| Alpha-Tocopherol | 0.01 (-0.64;0.67) | 9.80E-01 | 0.16 (-0.52;0.85) | 7.11E-01 | 0.21 (-0.48;0.89) | 6.38E-01 |
| X-25957 | 0.18 (-0.48;0.83) | 6.84E-01 | 0.21 (-0.45;0.86) | 6.21E-01 | 0.20 (-0.46;0.85) | 6.38E-01 |
| Ergothioneine | 0.11 (-0.55;0.77) | 8.14E-01 | 0.17 (-0.51;0.84) | 7.05E-01 | 0.20 (-0.47;0.88) | 6.41E-01 |
| X-18921 | -0.14 (-0.79;0.51) | 7.62E-01 | -0.21 (-0.87;0.44) | 6.13E-01 | -0.20 (-0.85;0.46) | 6.41E-01 |
| 1,2-Dilinoleoyl-GPC (18:2/18:2) | 0.16 (-0.50;0.82) | 7.13E-01 | 0.15 (-0.58;0.88) | 7.60E-01 | 0.22 (-0.52;0.95) | 6.47E-01 |
| X-25936 | 0.08 (-0.58;0.73) | 8.69E-01 | 0.13 (-0.52;0.79) | 7.60E-01 | 0.19 (-0.46;0.85) | 6.49E-01 |
| Cholic Acid Glucuronide | -0.31 (-1.53;0.91) | 7.05E-01 | -0.36 (-1.57;0.86) | 6.48E-01 | -0.35 (-1.57;0.87) | 6.55E-01 |
| Fructose | -0.62 (-1.84;0.59) | 4.17E-01 | -0.42 (-1.64;0.81) | 5.97E-01 | -0.37 (-1.65;0.91) | 6.55E-01 |
| 1-Stearoyl-2-Oleoyl-GPI (18:0/18:1)* | -0.21 (-0.86;0.45) | 6.27E-01 | -0.25 (-0.94;0.45) | 5.76E-01 | -0.20 (-0.89;0.50) | 6.61E-01 |
| 1-(1-Enyl-Palmitoyl)-2-Palmitoyl-GPC (P-16:0/16:0)* | -0.15 (-0.80;0.50) | 7.36E-01 | -0.27 (-0.96;0.42) | 5.43E-01 | -0.20 (-0.89;0.50) | 6.61E-01 |
| Arachidonoylcholine | -0.23 (-1.44;0.97) | 7.79E-01 | -0.25 (-1.53;1.02) | 7.66E-01 | -0.38 (-1.70;0.95) | 6.61E-01 |
| Docosatrienoate (22:3n3) | 0.39 (-0.82;1.61) | 6.19E-01 | 0.38 (-0.84;1.61) | 6.26E-01 | 0.35 (-0.88;1.58) | 6.62E-01 |
| Thymidine | 0.12 (-0.65;0.90) | 8.19E-01 | 0.12 (-0.66;0.90) | 8.21E-01 | 0.22 (-0.56;1.01) | 6.62E-01 |
| 1-(1-Enyl-Stearoyl)-GPE (P-18:0)* | 0.08 (-0.57;0.73) | 8.61E-01 | 0.18 (-0.52;0.88) | 6.95E-01 | 0.20 (-0.51;0.90) | 6.65E-01 |
| Sphingomyelin (D18:1/20:1, D18:2/20:0)* | -0.47 (-1.17;0.23) | 2.82E-01 | -0.30 (-1.03;0.43) | 5.19E-01 | -0.21 (-0.94;0.53) | 6.67E-01 |
| 9-Hydroxystearate | 0.26 (-0.42;0.94) | 5.53E-01 | 0.17 (-0.53;0.86) | 7.08E-01 | 0.19 (-0.50;0.89) | 6.71E-01 |
| 2-Methylserine | -0.27 (-0.92;0.38) | 5.19E-01 | -0.18 (-0.83;0.48) | 6.79E-01 | -0.18 (-0.83;0.48) | 6.78E-01 |
| 1-Stearoyl-GPG (18:0) | -0.26 (-0.92;0.39) | 5.33E-01 | -0.16 (-0.84;0.51) | 7.11E-01 | -0.18 (-0.85;0.49) | 6.79E-01 |
| Beta-Cryptoxanthin | -0.01 (-0.68;0.65) | 9.77E-01 | 0.14 (-0.56;0.84) | 7.66E-01 | 0.19 (-0.51;0.89) | 6.79E-01 |
| Glycosyl-N-(2-Hydroxynervonoyl)-Sphingosine (D18:1/24:1(2OH))* | -0.25 (-0.92;0.43) | 5.76E-01 | -0.21 (-0.89;0.48) | 6.42E-01 | -0.18 (-0.87;0.50) | 6.80E-01 |
| 1-Myristoylglycerol (14:0) | -0.30 (-1.51;0.90) | 7.07E-01 | -0.32 (-1.52;0.88) | 6.80E-01 | -0.31 (-1.52;0.89) | 6.91E-01 |
| Glycodeoxycholate 3-Sulfate | -0.14 (-0.79;0.52) | 7.64E-01 | -0.16 (-0.82;0.49) | 7.05E-01 | -0.17 (-0.83;0.49) | 6.98E-01 |
| N-Behenoyl-Sphingadienine (D18:2/22:0)* | -0.39 (-1.16;0.39) | 4.35E-01 | -0.27 (-1.08;0.53) | 6.00E-01 | -0.20 (-1.01;0.60) | 7.00E-01 |
| Ximenoylcarnitine (C26:1)* | -0.35 (-1.00;0.31) | 4.09E-01 | -0.26 (-0.95;0.44) | 5.62E-01 | -0.18 (-0.88;0.52) | 7.00E-01 |
| 1-Palmitoyl-2-Linoleoyl-GPI (16:0/18:2) | -0.16 (-0.81;0.50) | 7.25E-01 | -0.17 (-0.84;0.51) | 7.03E-01 | -0.17 (-0.85;0.51) | 7.02E-01 |
| 1-Arachidonylglycerol (20:4) | 0.08 (-1.16;1.32) | 9.29E-01 | 0.34 (-0.94;1.61) | 6.86E-01 | 0.32 (-0.96;1.60) | 7.03E-01 |
| X-22834 | 0.19 (-0.46;0.84) | 6.55E-01 | 0.20 (-0.46;0.86) | 6.42E-01 | 0.17 (-0.50;0.83) | 7.03E-01 |
| N-Stearoyl-Sphinganine (D18:0/18:0)* | 0.13 (-0.53;0.79) | 7.78E-01 | 0.15 (-0.57;0.87) | 7.50E-01 | 0.18 (-0.54;0.90) | 7.04E-01 |
| X-13658 | -0.23 (-1.44;0.97) | 7.78E-01 | -0.30 (-1.51;0.91) | 7.05E-01 | -0.30 (-1.51;0.91) | 7.04E-01 |
| Eicosanedioate (C20-DC) | 0.11 (-0.54;0.77) | 8.05E-01 | 0.11 (-0.55;0.77) | 8.07E-01 | 0.16 (-0.50;0.83) | 7.07E-01 |
| N-Acetylaspartate (NAA) | -0.24 (-1.44;0.96) | 7.73E-01 | -0.29 (-1.50;0.91) | 7.08E-01 | -0.30 (-1.50;0.91) | 7.09E-01 |
| 5alpha-Androstan-3alpha,17beta-Diol Monosulfate (2) | -0.24 (-1.27;0.80) | 7.38E-01 | -0.18 (-1.22;0.86) | 7.94E-01 | -0.25 (-1.29;0.79) | 7.16E-01 |
| Dihomo-Linoleoylcarnitine (C20:2)* | 0 (-0.67;0.67) | 9.97E-01 | 0.13 (-0.56;0.81) | 7.83E-01 | 0.16 (-0.52;0.85) | 7.20E-01 |
| X-21467 | -0.34 (-1.00;0.31) | 4.09E-01 | -0.17 (-0.88;0.54) | 7.10E-01 | -0.17 (-0.88;0.54) | 7.23E-01 |
| Pregnenediol Sulfate (C21H34O5S)* | 0.22 (-0.53;0.97) | 6.53E-01 | 0.20 (-0.56;0.96) | 6.84E-01 | 0.18 (-0.58;0.94) | 7.24E-01 |
| Palmitoylcarnitine (C16) | -0.26 (-0.93;0.41) | 5.45E-01 | -0.20 (-0.88;0.48) | 6.42E-01 | -0.16 (-0.84;0.52) | 7.26E-01 |
| Sphinganine-1-Phosphate | 0.06 (-0.59;0.72) | 8.90E-01 | 0.11 (-0.56;0.77) | 8.11E-01 | 0.15 (-0.51;0.81) | 7.30E-01 |
| Nisinate (24:6n3) | 0.34 (-0.88;1.55) | 6.71E-01 | 0.28 (-0.96;1.52) | 7.27E-01 | 0.28 (-0.96;1.52) | 7.37E-01 |
| 1-Linoleoyl-GPI (18:2)* | 0.05 (-0.60;0.71) | 9.13E-01 | 0.14 (-0.54;0.83) | 7.59E-01 | 0.15 (-0.53;0.84) | 7.37E-01 |
| Butyrylcarnitine (C4) | -0.19 (-0.85;0.46) | 6.51E-01 | -0.18 (-0.84;0.48) | 6.72E-01 | -0.15 (-0.81;0.52) | 7.38E-01 |
| Sphingomyelin (D18:1/19:0, D19:1/18:0)* | -0.40 (-1.09;0.30) | 3.69E-01 | -0.23 (-0.99;0.52) | 6.26E-01 | -0.17 (-0.92;0.59) | 7.39E-01 |
| 16a-Hydroxy DHEA 3-Sulfate | -0.10 (-0.78;0.59) | 8.40E-01 | -0.13 (-0.82;0.57) | 7.80E-01 | -0.15 (-0.85;0.54) | 7.41E-01 |
| Myristoyl-Linoleoyl-Glycerol (14:0/18:2) [2]* | 0.15 (-0.63;0.92) | 7.80E-01 | 0.21 (-0.60;1.02) | 6.94E-01 | 0.18 (-0.64;0.99) | 7.44E-01 |
| Linoleoyl-Linoleoyl-Glycerol (18:2/18:2) [1]* | -0.27 (-1.05;0.51) | 5.99E-01 | -0.18 (-0.98;0.62) | 7.29E-01 | -0.17 (-0.97;0.63) | 7.44E-01 |
| Oleoyl-Arachidonoyl-Glycerol (18:1/20:4) [1]* | 0.13 (-0.64;0.91) | 8.06E-01 | 0.20 (-0.61;1.02) | 7.05E-01 | 0.18 (-0.64;0.99) | 7.45E-01 |
| Docosahexaenoylcholine | -0.12 (-1.33;1.09) | 8.90E-01 | -0.18 (-1.46;1.09) | 8.32E-01 | -0.28 (-1.59;1.03) | 7.47E-01 |
| Lithocholate Sulfate (1) | 0.12 (-0.53;0.78) | 7.80E-01 | 0.19 (-0.48;0.85) | 6.63E-01 | 0.14 (-0.52;0.81) | 7.48E-01 |
| Stearoyl Sphingomyelin (D18:1/18:0) | -0.32 (-0.99;0.36) | 4.59E-01 | -0.22 (-0.92;0.49) | 6.30E-01 | -0.15 (-0.86;0.56) | 7.48E-01 |
| 1-(1-Enyl-Palmitoyl)-2-Palmitoleoyl-GPC (P-16:0/16:1)* | -0.06 (-0.73;0.61) | 9.09E-01 | -0.23 (-0.94;0.47) | 6.04E-01 | -0.15 (-0.86;0.56) | 7.49E-01 |
| 4-Hydroxy-2-Oxoglutaric Acid | 0.12 (-1.09;1.33) | 8.90E-01 | 0.23 (-0.98;1.43) | 7.79E-01 | 0.25 (-0.96;1.47) | 7.52E-01 |
| N-Oleoylserine | 0.13 (-0.52;0.79) | 7.69E-01 | 0.13 (-0.52;0.79) | 7.63E-01 | 0.14 (-0.52;0.80) | 7.53E-01 |
| 3-Hydroxydecanoate | 0.28 (-0.39;0.95) | 5.20E-01 | 0.22 (-0.46;0.89) | 6.21E-01 | 0.14 (-0.54;0.82) | 7.54E-01 |
| Sphingomyelin (D18:2/23:0, D18:1/23:1, D17:1/24:1)* | -0.33 (-1.04;0.38) | 4.69E-01 | -0.23 (-1.01;0.55) | 6.44E-01 | -0.16 (-0.95;0.63) | 7.56E-01 |
| N-Palmitoyl-Sphingosine (D18:1/16:0) | -0.01 (-0.67;0.65) | 9.80E-01 | 0.09 (-0.61;0.79) | 8.48E-01 | 0.14 (-0.56;0.84) | 7.60E-01 |
| Arachidoylcarnitine (C20)* | 0 (-0.66;0.66) | 9.99E-01 | 0.12 (-0.59;0.83) | 8.05E-01 | 0.14 (-0.57;0.85) | 7.67E-01 |
| 3-Methylcytidine | 0.24 (-0.42;0.91) | 5.72E-01 | 0.13 (-0.55;0.80) | 7.76E-01 | 0.13 (-0.54;0.81) | 7.67E-01 |
| Sphingomyelin (D17:1/14:0, D16:1/15:0)* | -0.37 (-1.09;0.36) | 4.26E-01 | -0.24 (-1.04;0.57) | 6.50E-01 | -0.16 (-0.98;0.66) | 7.67E-01 |
| Behenoyl Sphingomyelin (D18:1/22:0)* | 0.05 (-0.61;0.71) | 9.21E-01 | 0.10 (-0.61;0.80) | 8.38E-01 | 0.14 (-0.57;0.84) | 7.70E-01 |
| Arginine | 0.09 (-0.56;0.74) | 8.43E-01 | 0.08 (-0.59;0.74) | 8.58E-01 | 0.13 (-0.54;0.79) | 7.78E-01 |
| Palmitoyl-Oleoyl-Glycerol (16:0/18:1) [2]* | 0.13 (-0.65;0.91) | 8.08E-01 | 0.16 (-0.68;1.00) | 7.71E-01 | 0.15 (-0.68;0.99) | 7.83E-01 |
| X-17653 | 0 (-0.66;0.66) | 9.97E-01 | 0.13 (-0.54;0.80) | 7.66E-01 | 0.12 (-0.55;0.79) | 7.83E-01 |
| Palmitoleoyl-Oleoyl-Glycerol (16:1/18:1) [2]* | -0.06 (-0.84;0.73) | 9.25E-01 | -0.16 (-0.97;0.66) | 7.76E-01 | -0.15 (-0.97;0.67) | 7.86E-01 |
| X-21327 | 0.29 (-0.91;1.49) | 7.21E-01 | 0.24 (-0.97;1.44) | 7.67E-01 | 0.22 (-0.99;1.43) | 7.86E-01 |
| Glycosyl Ceramide (D18:1/20:0, D16:1/22:0)* | 0 (-0.66;0.66) | 9.97E-01 | 0.03 (-0.70;0.76) | 9.50E-01 | 0.13 (-0.61;0.87) | 7.90E-01 |
| Ceramide (D18:1/20:0, D16:1/22:0, D20:1/18:0)* | 0.02 (-0.76;0.80) | 9.72E-01 | 0.11 (-0.69;0.90) | 8.40E-01 | 0.14 (-0.66;0.93) | 7.96E-01 |
| 2-Stearoyl-GPE (18:0)* | -0.05 (-0.70;0.61) | 9.25E-01 | 0.14 (-0.55;0.82) | 7.68E-01 | 0.12 (-0.57;0.81) | 7.98E-01 |
| Cyclo(Leu-Pro) | -0.12 (-0.78;0.54) | 7.89E-01 | -0.09 (-0.79;0.62) | 8.53E-01 | -0.12 (-0.82;0.58) | 8.00E-01 |
| 1-Linoleoyl-GPE (18:2)* | 0.12 (-0.53;0.77) | 7.86E-01 | 0.12 (-0.56;0.80) | 7.98E-01 | 0.12 (-0.57;0.80) | 8.01E-01 |
| 1-(1-Enyl-Stearoyl)-2-Linoleoyl-GPE (P-18:0/18:2)* | -0.11 (-0.77;0.54) | 8.04E-01 | -0.13 (-0.81;0.56) | 7.80E-01 | -0.12 (-0.80;0.57) | 8.03E-01 |
| 7-Alpha-Hydroxy-3-Oxo-4-Cholestenoate (7-Hoca) | 0.09 (-0.57;0.75) | 8.44E-01 | 0.09 (-0.57;0.75) | 8.41E-01 | 0.11 (-0.55;0.77) | 8.07E-01 |
| Thioproline | 0.10 (-0.56;0.76) | 8.25E-01 | 0.12 (-0.54;0.78) | 7.91E-01 | 0.11 (-0.55;0.77) | 8.07E-01 |
| 1-Dihomo-Linolenylglycerol (20:3) | -0.31 (-1.58;0.96) | 7.16E-01 | -0.17 (-1.46;1.12) | 8.46E-01 | -0.21 (-1.51;1.09) | 8.09E-01 |
| Valine | -0.22 (-0.89;0.44) | 6.04E-01 | -0.12 (-0.79;0.56) | 7.99E-01 | -0.11 (-0.78;0.57) | 8.15E-01 |
| X-21441 | 0.16 (-0.50;0.83) | 7.16E-01 | 0.11 (-0.57;0.78) | 8.10E-01 | 0.11 (-0.57;0.78) | 8.15E-01 |
| Perfluorooctanoate (PFOA) | 0.08 (-0.59;0.74) | 8.69E-01 | 0.05 (-0.62;0.73) | 9.09E-01 | 0.10 (-0.57;0.78) | 8.21E-01 |
| 3,5-Dichloro-2,6-Dihydroxybenzoic Acid | -0.23 (-0.88;0.43) | 5.95E-01 | -0.16 (-0.82;0.50) | 7.08E-01 | -0.10 (-0.76;0.56) | 8.21E-01 |
| 3beta,7alpha-Dihydroxy-5-Cholestenoate | 0.13 (-0.53;0.79) | 7.78E-01 | 0.09 (-0.59;0.77) | 8.48E-01 | 0.10 (-0.58;0.78) | 8.23E-01 |
| Cholesterol | -0.08 (-0.74;0.59) | 8.65E-01 | 0.04 (-0.76;0.85) | 9.34E-01 | 0.12 (-0.69;0.93) | 8.23E-01 |
| 3-Methyl-2-Oxovalerate | 0.11 (-0.58;0.79) | 8.25E-01 | 0.13 (-0.57;0.83) | 7.80E-01 | 0.11 (-0.60;0.81) | 8.24E-01 |
| 1-(1-Enyl-Palmitoyl)-GPC (P-16:0)* | -0.20 (-0.85;0.45) | 6.39E-01 | -0.17 (-0.88;0.53) | 7.05E-01 | -0.11 (-0.82;0.61) | 8.24E-01 |
| X-12544 | 0.05 (-0.61;0.71) | 9.22E-01 | 0.11 (-0.55;0.77) | 8.00E-01 | 0.10 (-0.57;0.76) | 8.29E-01 |
| 1-Palmitoleoylglycerol (16:1)* | 0.06 (-0.61;0.72) | 9.11E-01 | 0.12 (-0.58;0.82) | 7.94E-01 | 0.10 (-0.60;0.80) | 8.29E-01 |
| 1-Stearoyl-2-Oleoyl-GPS (18:0/18:1) | -0.03 (-1.23;1.17) | 9.72E-01 | -0.16 (-1.37;1.04) | 8.40E-01 | -0.17 (-1.38;1.03) | 8.31E-01 |
| 1-Oleoyl-GPE (18:1) | 0.05 (-0.60;0.70) | 9.21E-01 | 0.10 (-0.56;0.77) | 8.14E-01 | 0.10 (-0.57;0.76) | 8.31E-01 |
| Docosahexaenoylcarnitine (C22:6)* | 0.08 (-1.13;1.29) | 9.30E-01 | 0.20 (-1.03;1.43) | 8.06E-01 | 0.18 (-1.06;1.41) | 8.31E-01 |
| Sphingosine 1-Phosphate | 0.01 (-0.64;0.66) | 9.80E-01 | 0.04 (-0.62;0.71) | 9.18E-01 | 0.09 (-0.57;0.76) | 8.33E-01 |
| Taurine | -0.04 (-0.70;0.61) | 9.28E-01 | 0 (-0.66;0.67) | 9.95E-01 | 0.09 (-0.58;0.77) | 8.41E-01 |
| 1-Palmitoyl-2-Oleoyl-GPC (16:0/18:1) | 0.09 (-0.57;0.75) | 8.41E-01 | 0.06 (-0.63;0.75) | 8.96E-01 | 0.09 (-0.60;0.78) | 8.46E-01 |
| X-16935 | -0.01 (-0.68;0.65) | 9.80E-01 | 0.14 (-0.54;0.81) | 7.66E-01 | 0.09 (-0.59;0.77) | 8.49E-01 |
| Lactosyl-N-Nervonoyl-Sphingosine (D18:1/24:1)* | 0.11 (-0.54;0.76) | 8.11E-01 | 0.02 (-0.69;0.73) | 9.60E-01 | 0.09 (-0.63;0.81) | 8.53E-01 |
| Thymol Sulfate | -0.02 (-0.68;0.64) | 9.69E-01 | -0.08 (-0.75;0.58) | 8.48E-01 | -0.08 (-0.74;0.58) | 8.57E-01 |
| Androstenediol (3alpha, 17alpha) Monosulfate (2) | 0.16 (-0.54;0.86) | 7.37E-01 | 0.11 (-0.59;0.82) | 8.08E-01 | 0.09 (-0.62;0.79) | 8.57E-01 |
| Diacylglycerol (16:1/18:2 [2], 16:0/18:3 [1])* | -0.12 (-0.89;0.66) | 8.25E-01 | -0.11 (-0.93;0.70) | 8.36E-01 | -0.10 (-0.92;0.72) | 8.57E-01 |
| Butyrate/Isobutyrate (4:0) | -0.12 (-0.78;0.53) | 7.80E-01 | -0.10 (-0.75;0.56) | 8.27E-01 | -0.08 (-0.74;0.58) | 8.60E-01 |
| Piperine | 0.03 (-0.62;0.69) | 9.43E-01 | 0.08 (-0.61;0.76) | 8.64E-01 | 0.08 (-0.60;0.77) | 8.61E-01 |
| 1-(1-Enyl-Palmitoyl)-2-Oleoyl-GPC (P-16:0/18:1)* | -0.04 (-0.71;0.62) | 9.29E-01 | -0.16 (-0.88;0.55) | 7.26E-01 | -0.09 (-0.81;0.64) | 8.61E-01 |
| N-Palmitoylserine | -0.12 (-0.78;0.54) | 7.89E-01 | -0.13 (-0.79;0.53) | 7.67E-01 | -0.08 (-0.74;0.59) | 8.61E-01 |
| Androstenediol (3beta,17beta) Monosulfate (1) | 0.04 (-0.69;0.76) | 9.45E-01 | -0.05 (-0.79;0.68) | 9.11E-01 | -0.09 (-0.82;0.65) | 8.61E-01 |
| Glycosyl Ceramide (D18:2/24:1, D18:1/24:2)* | -0.03 (-0.69;0.64) | 9.58E-01 | -0.02 (-0.75;0.70) | 9.60E-01 | 0.09 (-0.65;0.83) | 8.61E-01 |
| Oleoylcarnitine (C18:1) | 0.01 (-0.66;0.67) | 9.93E-01 | 0.04 (-0.63;0.70) | 9.34E-01 | 0.08 (-0.59;0.75) | 8.62E-01 |
| Palmitoyl-Linoleoyl-Glycerol (16:0/18:2) [2]* | 0.04 (-0.74;0.82) | 9.42E-01 | 0.11 (-0.71;0.93) | 8.40E-01 | 0.09 (-0.72;0.91) | 8.62E-01 |
| Pregnenetriol Sulfate* | 0.13 (-0.62;0.87) | 8.05E-01 | 0.11 (-0.64;0.86) | 8.32E-01 | 0.09 (-0.67;0.84) | 8.62E-01 |
| Palmitoyl-Sphingosine-Phosphoethanolamine (D18:1/16:0) | -0.12 (-0.78;0.54) | 7.96E-01 | -0.09 (-0.82;0.64) | 8.48E-01 | -0.08 (-0.82;0.65) | 8.63E-01 |
| Sphingomyelin (D18:1/17:0, D17:1/18:0, D19:1/16:0) | -0.13 (-0.81;0.54) | 7.76E-01 | 0 (-0.73;0.72) | 9.95E-01 | 0.08 (-0.65;0.81) | 8.74E-01 |
| 1-Palmitoyl-2-Arachidonoyl-GPI (16:0/20:4)* | 0.01 (-0.66;0.67) | 9.90E-01 | 0.07 (-0.62;0.76) | 8.82E-01 | 0.07 (-0.62;0.76) | 8.74E-01 |
| Lactate | 0.04 (-0.62;0.69) | 9.40E-01 | 0.08 (-0.58;0.74) | 8.56E-01 | 0.07 (-0.59;0.73) | 8.74E-01 |
| X-24970 | 0.08 (-0.57;0.74) | 8.53E-01 | 0.05 (-0.60;0.70) | 9.11E-01 | 0.07 (-0.59;0.72) | 8.77E-01 |
| Palmitoyl Sphingomyelin (D18:1/16:0) | -0.04 (-0.71;0.62) | 9.28E-01 | 0.02 (-0.72;0.75) | 9.73E-01 | 0.08 (-0.67;0.82) | 8.77E-01 |
| Gamma-Glutamyl-2-Aminobutyrate | -0.06 (-0.71;0.60) | 9.09E-01 | -0.09 (-0.74;0.57) | 8.43E-01 | -0.07 (-0.72;0.59) | 8.78E-01 |
| Linoleoyl-Arachidonoyl-Glycerol (18:2/20:4) [2]* | -0.12 (-0.90;0.65) | 8.19E-01 | -0.03 (-0.84;0.78) | 9.56E-01 | -0.08 (-0.89;0.73) | 8.78E-01 |
| Uracil | -0.03 (-0.69;0.62) | 9.42E-01 | 0.06 (-0.60;0.71) | 9.00E-01 | 0.07 (-0.59;0.72) | 8.78E-01 |
| X-24243 | -0.07 (-0.72;0.59) | 8.85E-01 | -0.06 (-0.72;0.60) | 8.94E-01 | -0.06 (-0.73;0.60) | 8.86E-01 |
| Stearoyl Ethanolamide | -0.13 (-0.93;0.66) | 8.10E-01 | -0.06 (-0.88;0.77) | 9.18E-01 | -0.08 (-0.90;0.75) | 8.91E-01 |
| Sphingomyelin (D18:2/18:1)* | -0.29 (-0.99;0.41) | 5.19E-01 | -0.16 (-0.89;0.57) | 7.50E-01 | -0.07 (-0.80;0.67) | 8.91E-01 |
| X-16964 | 0.05 (-0.73;0.82) | 9.31E-01 | 0.07 (-0.71;0.85) | 8.96E-01 | 0.07 (-0.71;0.85) | 8.91E-01 |
| 2-Piperidinone | 0.02 (-0.63;0.68) | 9.60E-01 | 0.04 (-0.63;0.70) | 9.34E-01 | 0.06 (-0.61;0.73) | 8.96E-01 |
| Cysteine | -0.01 (-0.67;0.64) | 9.76E-01 | 0.02 (-0.64;0.68) | 9.60E-01 | 0.06 (-0.60;0.71) | 9.00E-01 |
| Glycosyl-N-Stearoyl-Sphingosine (D18:1/18:0) | -0.02 (-0.69;0.66) | 9.72E-01 | -0.02 (-0.76;0.71) | 9.60E-01 | 0.06 (-0.68;0.80) | 9.02E-01 |
| Oleoyl-Oleoyl-Glycerol (18:1/18:1) [1]* | -0.13 (-0.90;0.65) | 8.14E-01 | -0.10 (-0.91;0.71) | 8.48E-01 | -0.06 (-0.87;0.74) | 9.04E-01 |
| 4-Cholesten-3-One | -0.09 (-0.75;0.57) | 8.40E-01 | 0.01 (-0.68;0.70) | 9.82E-01 | 0.05 (-0.64;0.74) | 9.09E-01 |
| X-26109 | -0.21 (-0.87;0.46) | 6.35E-01 | -0.10 (-0.79;0.59) | 8.32E-01 | -0.05 (-0.74;0.64) | 9.09E-01 |
| Oleoyl-Arachidonoyl-Glycerol (18:1/20:4) [2]* | 0.03 (-0.75;0.80) | 9.60E-01 | 0.09 (-0.72;0.91) | 8.61E-01 | 0.06 (-0.75;0.87) | 9.13E-01 |
| 1-Palmitoyl-GPI (16:0) | -0.05 (-0.71;0.62) | 9.23E-01 | 0.03 (-0.68;0.73) | 9.57E-01 | 0.05 (-0.66;0.76) | 9.15E-01 |
| N-Methylproline | -0.19 (-0.84;0.46) | 6.56E-01 | -0.07 (-0.73;0.60) | 8.78E-01 | -0.05 (-0.71;0.62) | 9.17E-01 |
| Metabolonic Lactone Sulfate | -0.03 (-0.68;0.63) | 9.52E-01 | 0.03 (-0.67;0.74) | 9.48E-01 | 0.05 (-0.66;0.75) | 9.17E-01 |
| Phosphate | -0.07 (-1.36;1.22) | 9.38E-01 | -0.12 (-1.41;1.17) | 8.92E-01 | -0.09 (-1.39;1.21) | 9.20E-01 |
| Gamma-Glutamylglutamine | -0.07 (-0.73;0.58) | 8.70E-01 | -0.10 (-0.77;0.57) | 8.32E-01 | -0.04 (-0.72;0.63) | 9.21E-01 |
| Gamma-Tocopherol/Beta-Tocopherol | -0.11 (-0.76;0.54) | 8.11E-01 | -0.07 (-0.73;0.59) | 8.79E-01 | -0.04 (-0.70;0.62) | 9.23E-01 |
| 4-Methyl-2-Oxopentanoate | 0.07 (-0.62;0.76) | 8.86E-01 | 0.06 (-0.63;0.76) | 8.96E-01 | 0.04 (-0.66;0.74) | 9.23E-01 |
| 1-(1-Enyl-Palmitoyl)-2-Linoleoyl-GPE (P-16:0/18:2)* | -0.09 (-0.74;0.56) | 8.45E-01 | -0.07 (-0.73;0.60) | 8.80E-01 | -0.04 (-0.71;0.62) | 9.23E-01 |
| N-Palmitoyl-Sphinganine (D18:0/16:0) | -0.14 (-0.80;0.51) | 7.48E-01 | 0.01 (-0.70;0.72) | 9.81E-01 | 0.04 (-0.67;0.76) | 9.25E-01 |
| Palmitoleoyl-Arachidonoyl-Glycerol (16:1/20:4) [2]* | -0.12 (-0.90;0.65) | 8.19E-01 | -0.05 (-0.85;0.75) | 9.27E-01 | -0.05 (-0.85;0.75) | 9.25E-01 |
| Margaroylcarnitine (C17)* | -0.20 (-0.87;0.47) | 6.47E-01 | -0.10 (-0.78;0.59) | 8.36E-01 | -0.04 (-0.73;0.65) | 9.29E-01 |
| Pipecolate | 0.04 (-0.62;0.70) | 9.37E-01 | -0.05 (-0.71;0.61) | 9.08E-01 | -0.04 (-0.70;0.63) | 9.32E-01 |
| Sphingomyelin (D18:1/20:0, D16:1/22:0)* | -0.11 (-0.77;0.55) | 8.14E-01 | -0.02 (-0.70;0.67) | 9.73E-01 | 0.04 (-0.66;0.73) | 9.36E-01 |
| Branched Chain 14:0 Dicarboxylic Acid** | -0.16 (-1.38;1.05) | 8.46E-01 | -0.05 (-1.28;1.19) | 9.56E-01 | -0.06 (-1.30;1.18) | 9.38E-01 |
| 4-Chlorobenzoic Acid | -0.04 (-0.69;0.61) | 9.31E-01 | 0 (-0.66;0.65) | 9.95E-01 | 0.03 (-0.62;0.69) | 9.40E-01 |
| Lactosyl-N-Palmitoyl-Sphingosine (D18:1/16:0) | 0 (-0.65;0.65) | 9.99E-01 | -0.05 (-0.73;0.64) | 9.18E-01 | 0.03 (-0.66;0.72) | 9.45E-01 |
| Octadecenedioate (C18:1-DC) | 0 (-0.67;0.66) | 9.97E-01 | -0.04 (-0.71;0.64) | 9.34E-01 | -0.03 (-0.70;0.65) | 9.49E-01 |
| 1-Linoleoyl-2-Linolenoyl-GPC (18:2/18:3)* | 0.04 (-0.62;0.70) | 9.31E-01 | -0.01 (-0.71;0.69) | 9.81E-01 | 0.03 (-0.68;0.73) | 9.53E-01 |
| 1-Linoleoylglycerol (18:2) | -0.13 (-0.78;0.53) | 7.80E-01 | 0.05 (-0.62;0.72) | 9.11E-01 | 0.02 (-0.64;0.69) | 9.55E-01 |
| 17alpha-Hydroxypregnenolone 3-Sulfate | 0.18 (-1.05;1.41) | 8.32E-01 | 0.06 (-1.18;1.29) | 9.45E-01 | 0.04 (-1.19;1.28) | 9.59E-01 |
| 2,4-Di-Tert-Butylphenol | 0.15 (-1.07;1.38) | 8.55E-01 | 0.07 (-1.22;1.35) | 9.35E-01 | 0.04 (-1.25;1.33) | 9.61E-01 |
| Sphingomyelin (D18:2/24:1, D18:1/24:2)* | -0.14 (-0.82;0.54) | 7.69E-01 | -0.10 (-0.82;0.61) | 8.32E-01 | -0.02 (-0.75;0.70) | 9.62E-01 |
| Linoleoyl-Arachidonoyl-Glycerol (18:2/20:4) [1]* | -0.02 (-0.80;0.75) | 9.66E-01 | 0.06 (-0.74;0.86) | 9.11E-01 | 0.02 (-0.78;0.83) | 9.62E-01 |
| Cerotoylcarnitine (C26)* | -0.26 (-0.92;0.40) | 5.45E-01 | -0.08 (-0.78;0.62) | 8.61E-01 | -0.02 (-0.72;0.68) | 9.62E-01 |
| Pregnenolone Sulfate | 0.10 (-0.60;0.81) | 8.36E-01 | 0.07 (-0.65;0.79) | 8.83E-01 | 0.02 (-0.70;0.74) | 9.62E-01 |
| Palmitoyl-Arachidonoyl-Glycerol (16:0/20:4) [2]* | 0.02 (-0.76;0.80) | 9.72E-01 | 0.06 (-0.75;0.87) | 9.11E-01 | -0.02 (-0.83;0.79) | 9.62E-01 |
| 1-Oleoyl-GPI (18:1) | -0.03 (-0.68;0.63) | 9.59E-01 | -0.01 (-0.69;0.68) | 9.92E-01 | 0.02 (-0.67;0.70) | 9.70E-01 |
| 1-(1-Enyl-Palmitoyl)-2-Arachidonoyl-GPC (P-16:0/20:4)* | -0.06 (-0.71;0.60) | 9.09E-01 | -0.03 (-0.70;0.63) | 9.39E-01 | 0.01 (-0.65;0.68) | 9.74E-01 |
| 3-Hydroxymyristate | 0.06 (-0.73;0.85) | 9.25E-01 | 0.09 (-0.72;0.89) | 8.69E-01 | -0.02 (-0.82;0.79) | 9.74E-01 |
| Valylglycine | 0.07 (-0.59;0.73) | 8.81E-01 | 0 (-0.67;0.67) | 9.99E-01 | 0.01 (-0.67;0.68) | 9.80E-01 |
| X-23974 | -0.03 (-0.69;0.62) | 9.45E-01 | 0.01 (-0.65;0.66) | 9.91E-01 | 0.01 (-0.65;0.66) | 9.81E-01 |
| Glycolithocholate | 0.03 (-0.63;0.68) | 9.59E-01 | 0.07 (-0.58;0.72) | 8.74E-01 | 0.01 (-0.65;0.66) | 9.85E-01 |
| Glycerol | 0.05 (-0.66;0.76) | 9.25E-01 | 0.02 (-0.70;0.74) | 9.65E-01 | -0.01 (-0.73;0.72) | 9.85E-01 |

Model 1 is adjusted for age, sex, and Rotterdam Study Cohort

Model 2 is additionally adjusted for body mass index, smoking status, alcohol use, serum cholesterol, lipid-lowering drugs, and prevalent cardiovascular disease

Model 3 is additionally adjusted for hypertension and diabetes

* P-values are FDR-corrected.

Abbreviations: CI = confidence interval; eGFRcreat = estimated glomerular filtration rate based on serum creatinine

**Supplemental Table 3.** Association between serum metabolites from the Nightingale platform and eGFRcys at baseline (n = 3,091)

|  | **Model 1** |  | **Model 2** |  | **Model 3** |  |
| --- | --- | --- | --- | --- | --- | --- |
| **Metabolite** | **Beta (95% CI)** | **P-value** | **Beta (95% CI)** | **P-value** | **Beta (95% CI)** | **P-value** |
| Citrate | -2.01 (-2.74;-1.29) | 5.67E-06 | -2.02 (-2.74;-1.30) | 4.54E-06 | -2.01 (-2.74;-1.29) | 5.67E-06 |
| Cholesteryl esters in small HDL | 1.73 (0.99;2.47) | 3.64E-04 | 1.73 (0.99;2.47) | 4.06E-04 | 1.73 (0.99;2.47) | 3.64E-04 |
| Glycine | -1.64 (-2.37;-0.92) | 5.79E-04 | -1.62 (-2.34;-0.89) | 7.60E-04 | -1.64 (-2.37;-0.92) | 5.79E-04 |
| Cholesterol in small HDL | 1.75 (0.96;2.53) | 7.25E-04 | 1.74 (0.95;2.53) | 7.60E-04 | 1.75 (0.96;2.53) | 7.25E-04 |
| Concentration of small HDL particles | 1.67 (0.87;2.48) | 1.86E-03 | 1.67 (0.87;2.48) | 1.45E-03 | 1.67 (0.87;2.48) | 1.86E-03 |
| Glutamine | -1.54 (-2.30;-0.79) | 2.16E-03 | -1.63 (-2.38;-0.88) | 8.16E-04 | -1.54 (-2.30;-0.79) | 2.16E-03 |
| Phenylalanine | -1.47 (-2.19;-0.74) | 2.28E-03 | -1.52 (-2.25;-0.80) | 1.33E-03 | -1.47 (-2.19;-0.74) | 2.28E-03 |
| Ratio of docosahexaenoic acid to total fatty acids | 1.50 (0.74;2.25) | 2.82E-03 | 1.55 (0.79;2.30) | 1.62E-03 | 1.50 (0.74;2.25) | 2.82E-03 |
| Triglycerides in very large HDL | -1.32 (-2.02;-0.63) | 4.62E-03 | -1.29 (-1.98;-0.60) | 5.95E-03 | -1.32 (-2.02;-0.63) | 4.62E-03 |
| Triglycerides in large HDL | -1.16 (-1.83;-0.50) | 1.36E-02 | -1.14 (-1.80;-0.47) | 1.48E-02 | -1.16 (-1.83;-0.50) | 1.36E-02 |
| Ratio of monounsaturated fatty acids to total fatty acids | -1.15 (-1.81;-0.49) | 1.36E-02 | -1.16 (-1.82;-0.51) | 1.10E-02 | -1.15 (-1.81;-0.49) | 1.36E-02 |
| Docosahexaenoic acid | 1.48 (0.62;2.34) | 1.38E-02 | 1.52 (0.66;2.38) | 1.10E-02 | 1.48 (0.62;2.34) | 1.38E-02 |
| Total lipids in small HDL | 1.34 (0.56;2.13) | 1.38E-02 | 1.34 (0.55;2.12) | 1.48E-02 | 1.34 (0.56;2.13) | 1.38E-02 |
| Degree of unsaturation | 1.07 (0.41;1.73) | 2.38E-02 | 1.10 (0.45;1.76) | 1.66E-02 | 1.07 (0.41;1.73) | 2.38E-02 |
| Triglycerides to total lipids ratio in medium LDL | -1.19 (-1.93;-0.45) | 2.40E-02 | -1.15 (-1.88;-0.41) | 2.80E-02 | -1.19 (-1.93;-0.45) | 2.40E-02 |
| Cholesteryl esters to total lipids ratio in small HDL | 1 (0.38;1.62) | 2.40E-02 | 0.98 (0.37;1.60) | 2.66E-02 | 1.00 (0.38;1.62) | 2.40E-02 |
| Triglycerides to total lipids ratio in large LDL | -1.20 (-1.96;-0.44) | 2.50E-02 | -1.15 (-1.91;-0.40) | 2.80E-02 | -1.20 (-1.96;-0.44) | 2.50E-02 |
| Free cholesterol to total lipids ratio in small HDL | -1.28 (-2.08;-0.47) | 2.50E-02 | -1.23 (-2.04;-0.43) | 2.80E-02 | -1.28 (-2.08;-0.47) | 2.50E-02 |
| Concentration of HDL particles | 1.30 (0.46;2.14) | 2.56E-02 | 1.30 (0.46;2.14) | 2.80E-02 | 1.30 (0.46;2.14) | 2.56E-02 |
| Cholesteryl esters in medium HDL | 1.16 (0.41;1.90) | 2.56E-02 | 1.14 (0.40;1.89) | 2.80E-02 | 1.16 (0.41;1.90) | 2.56E-02 |
| Ratio of polyunsaturated fatty acids to monounsaturated fatty acids | 1.04 (0.38;1.71) | 2.56E-02 | 1.05 (0.40;1.71) | 2.63E-02 | 1.04 (0.38;1.71) | 2.56E-02 |
| Phospholipids in small HDL | 1.20 (0.43;1.96) | 2.56E-02 | 1.19 (0.42;1.96) | 2.80E-02 | 1.20 (0.43;1.96) | 2.56E-02 |
| Total concentration of lipoprotein particles | 1.42 (0.50;2.35) | 2.56E-02 | 1.43 (0.51;2.36) | 2.80E-02 | 1.42 (0.50;2.35) | 2.56E-02 |
| Albumin | 1.11 (0.38;1.83) | 2.72E-02 | 1.11 (0.38;1.83) | 2.80E-02 | 1.11 (0.38;1.83) | 2.72E-02 |
| Cholesteryl esters to total lipids ratio in medium HDL | 0.95 (0.31;1.58) | 3.19E-02 | 0.92 (0.29;1.55) | 3.20E-02 | 0.95 (0.31;1.58) | 3.19E-02 |
| Cholesterol in medium HDL | 1.12 (0.36;1.88) | 3.41E-02 | 1.11 (0.35;1.87) | 3.20E-02 | 1.12 (0.36;1.88) | 3.41E-02 |
| Total lipids in chylomicrons and extremely large VLDL | -0.94 (-1.58;-0.30) | 3.41E-02 | -0.96 (-1.59;-0.33) | 2.81E-02 | -0.94 (-1.58;-0.30) | 3.41E-02 |
| Triglycerides to total lipids ratio in large HDL | -0.94 (-1.60;-0.29) | 3.85E-02 | -0.91 (-1.56;-0.26) | 4.00E-02 | -0.94 (-1.60;-0.29) | 3.85E-02 |
| Free cholesterol in chylomicrons and extremely large VLDL | -0.93 (-1.58;-0.28) | 3.85E-02 | -0.95 (-1.59;-0.31) | 3.20E-02 | -0.93 (-1.58;-0.28) | 3.85E-02 |
| Concentration of chylomicrons and extremely large VLDL particles | -0.94 (-1.58;-0.29) | 3.85E-02 | -0.96 (-1.60;-0.32) | 3.20E-02 | -0.94 (-1.58;-0.29) | 3.85E-02 |
| Cholesterol to total lipids ratio in medium HDL | 0.94 (0.28;1.59) | 3.86E-02 | 0.92 (0.27;1.57) | 4.00E-02 | 0.94 (0.28;1.59) | 3.86E-02 |
| Cholesterol in chylomicrons and extremely large VLDL | -0.92 (-1.56;-0.28) | 3.86E-02 | -0.93 (-1.57;-0.30) | 3.20E-02 | -0.92 (-1.56;-0.28) | 3.86E-02 |
| Ratio of omega-3 fatty acids to total fatty acids | 1.20 (0.35;2.05) | 3.99E-02 | 1.23 (0.38;2.08) | 3.39E-02 | 1.20 (0.35;2.05) | 3.99E-02 |
| Cholesterol to total lipids ratio in small LDL | 1.25 (0.36;2.13) | 3.99E-02 | 1.20 (0.33;2.08) | 4.55E-02 | 1.25 (0.36;2.13) | 3.99E-02 |
| Triglycerides to total lipids ratio in small LDL | -1.00 (-1.71;-0.29) | 3.99E-02 | -0.98 (-1.68;-0.28) | 4.00E-02 | -1.00 (-1.71;-0.29) | 3.99E-02 |
| Triglycerides in chylomicrons and extremely large VLDL | -0.89 (-1.53;-0.26) | 3.99E-02 | -0.93 (-1.56;-0.29) | 3.20E-02 | -0.89 (-1.53;-0.26) | 3.99E-02 |
| Concentration of very small VLDL particles | -1.31 (-2.25;-0.36) | 4.37E-02 | -1.28 (-2.23;-0.34) | 4.97E-02 | -1.31 (-2.25;-0.36) | 4.37E-02 |
| Cholesteryl esters in chylomicrons and extremely large VLDL | -0.89 (-1.53;-0.24) | 4.37E-02 | -0.90 (-1.54;-0.27) | 3.99E-02 | -0.89 (-1.53;-0.24) | 4.37E-02 |
| Cholesterol to total lipids ratio in large LDL | 1.06 (0.28;1.85) | 4.86E-02 | 1.01 (0.23;1.80) | 6.05E-02 | 1.06 (0.28;1.85) | 4.86E-02 |
| Phospholipids in chylomicrons and extremely large VLDL | -0.86 (-1.50;-0.23) | 4.86E-02 | -0.89 (-1.53;-0.26) | 4.00E-02 | -0.86 (-1.50;-0.23) | 4.86E-02 |
| Phospholipids in very small VLDL | -1.12 (-1.94;-0.29) | 4.96E-02 | -1.08 (-1.91;-0.25) | 5.98E-02 | -1.12 (-1.94;-0.29) | 4.96E-02 |
| Triglycerides to total lipids ratio in IDL | -1.04 (-1.81;-0.26) | 5.10E-02 | -0.99 (-1.75;-0.22) | 6.05E-02 | -1.04 (-1.81;-0.26) | 5.10E-02 |
| Free cholesterol in very small VLDL | -1.23 (-2.15;-0.30) | 5.25E-02 | -1.20 (-2.13;-0.28) | 5.98E-02 | -1.23 (-2.15;-0.30) | 5.25E-02 |
| Free cholesterol to total lipids ratio in small LDL | 0.86 (0.21;1.52) | 5.42E-02 | 0.87 (0.22;1.51) | 5.46E-02 | 0.86 (0.21;1.52) | 5.42E-02 |
| Total lipids in very small VLDL | -1.18 (-2.09;-0.28) | 5.53E-02 | -1.16 (-2.06;-0.25) | 6.30E-02 | -1.18 (-2.09;-0.28) | 5.53E-02 |
| Free cholesterol in small HDL | 1.21 (0.28;2.14) | 5.67E-02 | 1.22 (0.29;2.15) | 5.98E-02 | 1.21 (0.28;2.14) | 5.67E-02 |
| Triglycerides in IDL | -0.96 (-1.70;-0.22) | 5.71E-02 | -0.93 (-1.66;-0.19) | 6.64E-02 | -0.96 (-1.70;-0.22) | 5.71E-02 |
| Triglycerides in very small VLDL | -0.91 (-1.61;-0.21) | 5.72E-02 | -0.89 (-1.59;-0.19) | 6.43E-02 | -0.91 (-1.61;-0.21) | 5.72E-02 |
| Cholesterol to total lipids ratio in small HDL | 0.82 (0.18;1.46) | 6.23E-02 | 0.82 (0.18;1.46) | 6.30E-02 | 0.82 (0.18;1.46) | 6.23E-02 |
| Total lipids in medium HDL | 0.98 (0.20;1.75) | 6.49E-02 | 0.97 (0.20;1.75) | 6.64E-02 | 0.98 (0.20;1.75) | 6.49E-02 |
| Cholesteryl esters to total lipids ratio in IDL | 0.91 (0.19;1.63) | 6.53E-02 | 0.86 (0.14;1.57) | 7.89E-02 | 0.91 (0.19;1.63) | 6.53E-02 |
| Triglycerides to total lipids ratio in medium HDL | -0.81 (-1.46;-0.16) | 6.57E-02 | -0.81 (-1.45;-0.16) | 6.70E-02 | -0.81 (-1.46;-0.16) | 6.57E-02 |
| Concentration of medium HDL particles | 0.96 (0.19;1.74) | 6.64E-02 | 0.96 (0.19;1.74) | 6.70E-02 | 0.96 (0.19;1.74) | 6.64E-02 |
| Concentration of very large HDL particles | -0.90 (-1.63;-0.18) | 6.64E-02 | -0.90 (-1.62;-0.18) | 6.70E-02 | -0.90 (-1.63;-0.18) | 6.64E-02 |
| Glycoprotein acetyls | -0.94 (-1.69;-0.18) | 6.69E-02 | -0.97 (-1.72;-0.22) | 5.98E-02 | -0.94 (-1.69;-0.18) | 6.69E-02 |
| Cholesterol to total lipids ratio in IDL | 0.88 (0.16;1.61) | 7.35E-02 | 0.82 (0.11;1.54) | 9.56E-02 | 0.88 (0.16;1.61) | 7.35E-02 |
| Tyrosine | 0.83 (0.15;1.51) | 7.35E-02 | 0.76 (0.08;1.44) | 1.03E-01 | 0.83 (0.15;1.51) | 7.35E-02 |
| Triglycerides to total lipids ratio in small HDL | -0.79 (-1.45;-0.13) | 7.72E-02 | -0.79 (-1.44;-0.14) | 7.88E-02 | -0.79 (-1.45;-0.13) | 7.72E-02 |
| Phospholipids in medium HDL | 0.92 (0.15;1.69) | 7.96E-02 | 0.92 (0.14;1.69) | 8.31E-02 | 0.92 (0.15;1.69) | 7.96E-02 |
| Ratio of polyunsaturated fatty acids to total fatty acids | 0.79 (0.13;1.46) | 8.00E-02 | 0.81 (0.15;1.46) | 7.23E-02 | 0.79 (0.13;1.46) | 8.00E-02 |
| Cholesterol to total lipids ratio in very large HDL | 0.82 (0.13;1.50) | 8.16E-02 | 0.83 (0.14;1.52) | 7.89E-02 | 0.82 (0.13;1.50) | 8.16E-02 |
| Triglycerides in HDL | -0.76 (-1.42;-0.10) | 9.11E-02 | -0.75 (-1.40;-0.09) | 9.56E-02 | -0.76 (-1.42;-0.10) | 9.11E-02 |
| Free cholesterol in medium HDL | 0.93 (0.12;1.73) | 9.11E-02 | 0.93 (0.12;1.73) | 9.56E-02 | 0.93 (0.12;1.73) | 9.11E-02 |
| Phospholipids to total lipids ratio in very small VLDL | -0.78 (-1.46;-0.10) | 9.11E-02 | -0.73 (-1.40;-0.05) | 1.16E-01 | -0.78 (-1.46;-0.10) | 9.11E-02 |
| Cholesterol in large LDL | 1.71 (0.19;3.23) | 1.00E-01 | 1.69 (0.17;3.21) | 1.05E-01 | 1.71 (0.19;3.23) | 1.00E-01 |
| Triglycerides in LDL | -0.86 (-1.63;-0.10) | 1.00E-01 | -0.83 (-1.59;-0.07) | 1.10E-01 | -0.86 (-1.63;-0.10) | 1.00E-01 |
| Monounsaturated fatty acids | -0.81 (-1.53;-0.09) | 1.00E-01 | -0.81 (-1.52;-0.10) | 9.56E-02 | -0.81 (-1.53;-0.09) | 1.00E-01 |
| Cholesteryl esters in HDL | 0.86 (0.09;1.64) | 1.04E-01 | 0.86 (0.09;1.63) | 1.05E-01 | 0.86 (0.09;1.64) | 1.04E-01 |
| Cholesteryl esters in large LDL | 1.63 (0.16;3.11) | 1.04E-01 | 1.61 (0.14;3.09) | 1.10E-01 | 1.63 (0.16;3.11) | 1.04E-01 |
| Free cholesterol in medium LDL | 1.37 (0.13;2.61) | 1.04E-01 | 1.37 (0.13;2.61) | 1.06E-01 | 1.37 (0.13;2.61) | 1.04E-01 |
| Ratio of omega-6 fatty acids to omega-3 fatty acids | -0.94 (-1.80;-0.09) | 1.04E-01 | -0.96 (-1.81;-0.11) | 1.03E-01 | -0.94 (-1.80;-0.09) | 1.04E-01 |
| Triglycerides in small LDL | -0.79 (-1.51;-0.08) | 1.04E-01 | -0.78 (-1.49;-0.08) | 1.05E-01 | -0.79 (-1.51;-0.08) | 1.04E-01 |
| Triglycerides in large LDL | -0.85 (-1.63;-0.08) | 1.05E-01 | -0.81 (-1.59;-0.04) | 1.24E-01 | -0.85 (-1.63;-0.08) | 1.05E-01 |
| Phospholipids to total lipids ratio in medium LDL | 0.80 (0.06;1.53) | 1.09E-01 | 0.84 (0.11;1.57) | 9.56E-02 | 0.80 (0.06;1.53) | 1.09E-01 |
| Triglycerides in medium LDL | -0.82 (-1.58;-0.07) | 1.09E-01 | -0.79 (-1.54;-0.05) | 1.24E-01 | -0.82 (-1.58;-0.07) | 1.09E-01 |
| Free cholesterol in LDL | 1.48 (0.09;2.87) | 1.19E-01 | 1.47 (0.08;2.85) | 1.24E-01 | 1.48 (0.09;2.87) | 1.19E-01 |
| Total esterified cholesterol | 1.94 (0.10;3.78) | 1.23E-01 | 1.92 (0.08;3.76) | 1.26E-01 | 1.94 (0.10;3.78) | 1.23E-01 |
| Apolipoprotein A1 | 0.84 (0.03;1.65) | 1.29E-01 | 0.85 (0.04;1.65) | 1.26E-01 | 0.84 (0.03;1.65) | 1.29E-01 |
| Concentration of VLDL particles | -0.81 (-1.61;-0.02) | 1.38E-01 | -0.81 (-1.60;-0.03) | 1.31E-01 | -0.81 (-1.61;-0.02) | 1.38E-01 |
| Free cholesterol in very large HDL | -0.73 (-1.44;-0.02) | 1.38E-01 | -0.73 (-1.44;-0.02) | 1.33E-01 | -0.73 (-1.44;-0.02) | 1.38E-01 |
| Phospholipids in large LDL | 1.49 (0.02;2.96) | 1.40E-01 | 1.49 (0.02;2.96) | 1.35E-01 | 1.49 (0.02;2.96) | 1.40E-01 |
| Free cholesterol to total lipids ratio in medium LDL | 0.67 (0.01;1.34) | 1.40E-01 | 0.67 (0.02;1.33) | 1.33E-01 | 0.67 (0.01;1.34) | 1.40E-01 |
| Cholesteryl esters to total lipids ratio in large LDL | 0.70 (0.01;1.39) | 1.41E-01 | 0.66 (-0.03;1.34) | 1.63E-01 | 0.70 (0.01;1.39) | 1.41E-01 |
| HDL cholesterol | 0.79 (0.00;1.58) | 1.44E-01 | 0.79 (0.00;1.58) | 1.39E-01 | 0.79 (0.00;1.58) | 1.44E-01 |
| Phospholipids to total lipids ratio in medium HDL | -0.68 (-1.37;0.00) | 1.44E-01 | -0.69 (-1.37;-0.01) | 1.35E-01 | -0.68 (-1.37;0.00) | 1.44E-01 |
| Free cholesterol in large LDL | 1.41 (0.00;2.82) | 1.45E-01 | 1.38 (-0.02;2.78) | 1.48E-01 | 1.41 (0.00;2.82) | 1.45E-01 |
| Phospholipids to total lipids ratio in small VLDL | 0.74 (-0.01;1.49) | 1.52E-01 | 0.75 (0.01;1.50) | 1.36E-01 | 0.74 (-0.01;1.49) | 1.52E-01 |
| Ratio of triglycerides to phosphoglycerides | -0.69 (-1.39;0.01) | 1.52E-01 | -0.71 (-1.40;-0.02) | 1.33E-01 | -0.69 (-1.39;0.01) | 1.52E-01 |
| Cholesteryl esters in very large HDL | -0.68 (-1.38;0.01) | 1.52E-01 | -0.65 (-1.34;0.04) | 1.70E-01 | -0.68 (-1.38;0.01) | 1.52E-01 |
| Glucose | 0.95 (-0.03;1.93) | 1.53E-01 | 0.88 (0.04;1.72) | 1.26E-01 | 0.95 (-0.03;1.93) | 1.53E-01 |
| Total lipids in large LDL | 1.50 (-0.04;3.05) | 1.53E-01 | 1.50 (-0.04;3.05) | 1.54E-01 | 1.50 (-0.04;3.05) | 1.53E-01 |
| Omega-3 fatty acids | 0.84 (-0.03;1.71) | 1.53E-01 | 0.85 (-0.01;1.71) | 1.46E-01 | 0.84 (-0.03;1.71) | 1.53E-01 |
| Cholesterol in very small VLDL | -1.00 (-2.04;0.04) | 1.54E-01 | -0.97 (-2.01;0.07) | 1.70E-01 | -1.00 (-2.04;0.04) | 1.54E-01 |
| Free cholesterol in small LDL | 1.08 (-0.08;2.25) | 1.74E-01 | 1.09 (-0.07;2.25) | 1.70E-01 | 1.08 (-0.08;2.25) | 1.74E-01 |
| Concentration of small VLDL particles | -0.68 (-1.42;0.06) | 1.80E-01 | -0.68 (-1.41;0.06) | 1.78E-01 | -0.68 (-1.42;0.06) | 1.80E-01 |
| Triglycerides in small VLDL | -0.62 (-1.29;0.06) | 1.88E-01 | -0.62 (-1.29;0.05) | 1.72E-01 | -0.62 (-1.29;0.06) | 1.88E-01 |
| Total lipids in very large HDL | -0.67 (-1.42;0.07) | 1.89E-01 | -0.67 (-1.41;0.07) | 1.85E-01 | -0.67 (-1.42;0.07) | 1.89E-01 |
| Triglycerides to total lipids ratio in small VLDL | -0.67 (-1.42;0.07) | 1.91E-01 | -0.71 (-1.45;0.03) | 1.63E-01 | -0.67 (-1.42;0.07) | 1.91E-01 |
| Free cholesterol to total lipids ratio in small VLDL | 0.68 (-0.08;1.43) | 1.96E-01 | 0.68 (-0.06;1.43) | 1.83E-01 | 0.68 (-0.08;1.43) | 1.96E-01 |
| Concentration of IDL particles | -1.04 (-2.23;0.15) | 2.07E-01 | -1.02 (-2.20;0.17) | 2.17E-01 | -1.04 (-2.23;0.15) | 2.07E-01 |
| Phospholipids in very large HDL | -0.63 (-1.35;0.09) | 2.07E-01 | -0.64 (-1.35;0.07) | 1.91E-01 | -0.63 (-1.35;0.09) | 2.07E-01 |
| Ratio of apolipoprotein B to apolipoprotein A1 | -0.68 (-1.46;0.10) | 2.08E-01 | -0.68 (-1.46;0.10) | 2.03E-01 | -0.68 (-1.46;0.10) | 2.08E-01 |
| Cholesteryl esters in VLDL | -0.82 (-1.76;0.12) | 2.10E-01 | -0.83 (-1.77;0.11) | 2.03E-01 | -0.82 (-1.76;0.12) | 2.10E-01 |
| Total triglycerides | -0.60 (-1.29;0.09) | 2.13E-01 | -0.61 (-1.30;0.07) | 1.91E-01 | -0.60 (-1.29;0.09) | 2.13E-01 |
| VLDL cholesterol | -0.73 (-1.60;0.13) | 2.20E-01 | -0.75 (-1.60;0.11) | 2.08E-01 | -0.73 (-1.60;0.13) | 2.20E-01 |
| Clinical LDL cholesterol | 1.22 (-0.22;2.66) | 2.26E-01 | 1.19 (-0.25;2.63) | 2.36E-01 | 1.22 (-0.22;2.66) | 2.26E-01 |
| Total lipids in HDL | 0.66 (-0.14;1.47) | 2.41E-01 | 0.66 (-0.14;1.47) | 2.36E-01 | 0.66 (-0.14;1.47) | 2.41E-01 |
| Free cholesterol in VLDL | -0.65 (-1.43;0.14) | 2.41E-01 | -0.66 (-1.43;0.12) | 2.24E-01 | -0.65 (-1.43;0.14) | 2.41E-01 |
| LDL cholesterol | 1.16 (-0.25;2.56) | 2.41E-01 | 1.14 (-0.27;2.55) | 2.50E-01 | 1.16 (-0.25;2.56) | 2.41E-01 |
| Phospholipids in HDL | 0.65 (-0.15;1.44) | 2.45E-01 | 0.65 (-0.15;1.44) | 2.46E-01 | 0.65 (-0.15;1.44) | 2.45E-01 |
| Isoleucine | -0.63 (-1.40;0.15) | 2.45E-01 | -0.58 (-1.33;0.17) | 2.75E-01 | -0.63 (-1.40;0.15) | 2.45E-01 |
| Phospholipids in VLDL | -0.61 (-1.37;0.14) | 2.45E-01 | -0.62 (-1.37;0.13) | 2.36E-01 | -0.61 (-1.37;0.14) | 2.45E-01 |
| Cholesterol in very large HDL | -0.59 (-1.33;0.15) | 2.56E-01 | -0.58 (-1.32;0.15) | 2.57E-01 | -0.59 (-1.33;0.15) | 2.56E-01 |
| Total lipids in VLDL | -0.57 (-1.29;0.15) | 2.64E-01 | -0.58 (-1.30;0.13) | 2.44E-01 | -0.57 (-1.29;0.15) | 2.64E-01 |
| Total lipids in small VLDL | -0.60 (-1.36;0.17) | 2.67E-01 | -0.59 (-1.35;0.16) | 2.66E-01 | -0.60 (-1.36;0.17) | 2.67E-01 |
| Cholesterol to total lipids ratio in very small VLDL | 0.58 (-0.17;1.32) | 2.70E-01 | 0.54 (-0.19;1.26) | 3.00E-01 | 0.58 (-0.17;1.32) | 2.70E-01 |
| Phospholipids in LDL | 1.09 (-0.33;2.50) | 2.75E-01 | 1.09 (-0.33;2.50) | 2.75E-01 | 1.09 (-0.33;2.50) | 2.75E-01 |
| Total cholesterol | 1.46 (-0.45;3.37) | 2.77E-01 | 1.45 (-0.46;3.36) | 2.85E-01 | 1.46 (-0.45;3.37) | 2.77E-01 |
| Triglycerides in VLDL | -0.53 (-1.22;0.17) | 2.82E-01 | -0.55 (-1.23;0.14) | 2.52E-01 | -0.53 (-1.22;0.17) | 2.82E-01 |
| Cholesteryl esters to total lipids ratio in very small VLDL | 0.51 (-0.18;1.20) | 2.93E-01 | 0.49 (-0.19;1.16) | 3.05E-01 | 0.51 (-0.18;1.20) | 2.93E-01 |
| Triglycerides in medium HDL | -0.47 (-1.12;0.18) | 3.12E-01 | -0.47 (-1.11;0.18) | 3.05E-01 | -0.47 (-1.12;0.18) | 3.12E-01 |
| Triglycerides to total lipids ratio in very small VLDL | -0.57 (-1.37;0.22) | 3.12E-01 | -0.57 (-1.35;0.22) | 3.05E-01 | -0.57 (-1.37;0.22) | 3.12E-01 |
| Concentration of medium VLDL particles | -0.58 (-1.40;0.23) | 3.21E-01 | -0.60 (-1.41;0.21) | 3.00E-01 | -0.58 (-1.40;0.23) | 3.21E-01 |
| Ratio of omega-6 fatty acids to total fatty acids | 0.49 (-0.20;1.18) | 3.23E-01 | 0.50 (-0.18;1.17) | 3.03E-01 | 0.49 (-0.20;1.18) | 3.23E-01 |
| Concentration of large VLDL particles | -0.48 (-1.16;0.21) | 3.45E-01 | -0.50 (-1.18;0.19) | 3.05E-01 | -0.48 (-1.16;0.21) | 3.45E-01 |
| Cholesteryl esters to total lipids ratio in very large VLDL | 0.48 (-0.22;1.18) | 3.48E-01 | 0.52 (-0.17;1.22) | 2.85E-01 | 0.48 (-0.22;1.18) | 3.48E-01 |
| Cholesteryl esters in LDL | 0.90 (-0.43;2.23) | 3.53E-01 | 0.88 (-0.45;2.21) | 3.67E-01 | 0.90 (-0.43;2.23) | 3.53E-01 |
| Total lipids in LDL | 0.95 (-0.47;2.36) | 3.59E-01 | 0.94 (-0.48;2.35) | 3.67E-01 | 0.95 (-0.47;2.36) | 3.59E-01 |
| Cholesteryl esters in small VLDL | -0.57 (-1.42;0.28) | 3.59E-01 | -0.55 (-1.40;0.30) | 3.72E-01 | -0.57 (-1.42;0.28) | 3.59E-01 |
| Free cholesterol in very large VLDL | -0.44 (-1.10;0.22) | 3.59E-01 | -0.46 (-1.12;0.19) | 3.19E-01 | -0.44 (-1.10;0.22) | 3.59E-01 |
| Cholesteryl esters in very small VLDL | -0.67 (-1.67;0.34) | 3.62E-01 | -0.63 (-1.62;0.37) | 3.76E-01 | -0.67 (-1.67;0.34) | 3.62E-01 |
| Free cholesterol in medium VLDL | -0.59 (-1.50;0.32) | 3.75E-01 | -0.61 (-1.51;0.30) | 3.60E-01 | -0.59 (-1.50;0.32) | 3.75E-01 |
| 3-Hydroxybutyrate | -0.43 (-1.11;0.25) | 3.87E-01 | -0.44 (-1.12;0.24) | 3.72E-01 | -0.43 (-1.11;0.25) | 3.87E-01 |
| Remnant cholesterol (non-HDL, non-LDL -cholesterol) | -0.81 (-2.10;0.47) | 3.87E-01 | -0.82 (-2.11;0.46) | 3.73E-01 | -0.81 (-2.10;0.47) | 3.87E-01 |
| Cholesterol in small VLDL | -0.57 (-1.47;0.33) | 3.87E-01 | -0.55 (-1.45;0.35) | 3.82E-01 | -0.57 (-1.47;0.33) | 3.87E-01 |
| Phospholipids in small VLDL | -0.55 (-1.42;0.32) | 3.87E-01 | -0.54 (-1.40;0.33) | 3.76E-01 | -0.55 (-1.42;0.32) | 3.87E-01 |
| Valine | 0.48 (-0.28;1.23) | 3.87E-01 | 0.48 (-0.25;1.22) | 3.67E-01 | 0.48 (-0.28;1.23) | 3.87E-01 |
| Cholesteryl esters to total lipids ratio in medium LDL | -0.42 (-1.09;0.26) | 3.98E-01 | -0.45 (-1.12;0.22) | 3.58E-01 | -0.42 (-1.09;0.26) | 3.98E-01 |
| Total lipids in large VLDL | -0.41 (-1.11;0.28) | 4.05E-01 | -0.44 (-1.13;0.25) | 3.73E-01 | -0.41 (-1.11;0.28) | 4.05E-01 |
| Triglycerides in large VLDL | -0.42 (-1.11;0.27) | 4.05E-01 | -0.45 (-1.14;0.24) | 3.67E-01 | -0.42 (-1.11;0.27) | 4.05E-01 |
| Phospholipids in medium LDL | 0.70 (-0.46;1.86) | 4.05E-01 | 0.70 (-0.47;1.86) | 3.94E-01 | 0.70 (-0.46;1.86) | 4.05E-01 |
| Total lipids in medium VLDL | -0.47 (-1.24;0.31) | 4.05E-01 | -0.49 (-1.25;0.28) | 3.76E-01 | -0.47 (-1.24;0.31) | 4.05E-01 |
| Phospholipids in medium VLDL | -0.52 (-1.37;0.34) | 4.05E-01 | -0.53 (-1.38;0.32) | 3.76E-01 | -0.52 (-1.37;0.34) | 4.05E-01 |
| Triglycerides in medium VLDL | -0.42 (-1.12;0.27) | 4.05E-01 | -0.44 (-1.13;0.24) | 3.72E-01 | -0.42 (-1.12;0.27) | 4.05E-01 |
| Total fatty acids | -0.51 (-1.35;0.34) | 4.05E-01 | -0.51 (-1.34;0.32) | 3.82E-01 | -0.51 (-1.35;0.34) | 4.05E-01 |
| Triglycerides to total lipids ratio in very large HDL | -0.41 (-1.11;0.28) | 4.07E-01 | -0.39 (-1.08;0.30) | 4.12E-01 | -0.41 (-1.11;0.28) | 4.07E-01 |
| Cholesterol in very large VLDL | -0.41 (-1.10;0.28) | 4.07E-01 | -0.43 (-1.11;0.26) | 3.76E-01 | -0.41 (-1.10;0.28) | 4.07E-01 |
| Cholesteryl esters in large VLDL | -0.41 (-1.10;0.29) | 4.11E-01 | -0.42 (-1.12;0.27) | 3.82E-01 | -0.41 (-1.10;0.29) | 4.11E-01 |
| Total lipids in very large VLDL | -0.39 (-1.07;0.28) | 4.16E-01 | -0.42 (-1.10;0.25) | 3.76E-01 | -0.39 (-1.07;0.28) | 4.16E-01 |
| Cholesterol in large VLDL | -0.40 (-1.08;0.29) | 4.21E-01 | -0.41 (-1.10;0.27) | 3.89E-01 | -0.40 (-1.08;0.29) | 4.21E-01 |
| Linoleic acid | -0.58 (-1.59;0.43) | 4.23E-01 | -0.58 (-1.59;0.43) | 4.12E-01 | -0.58 (-1.59;0.43) | 4.23E-01 |
| Triglycerides in small HDL | -0.37 (-1.03;0.28) | 4.27E-01 | -0.38 (-1.03;0.28) | 4.12E-01 | -0.37 (-1.03;0.28) | 4.27E-01 |
| Free cholesterol in large VLDL | -0.38 (-1.06;0.30) | 4.38E-01 | -0.40 (-1.07;0.28) | 4.02E-01 | -0.38 (-1.06;0.30) | 4.38E-01 |
| Cholesterol to total lipids ratio in small VLDL | 0.39 (-0.31;1.10) | 4.38E-01 | 0.42 (-0.28;1.11) | 3.94E-01 | 0.39 (-0.31;1.10) | 4.38E-01 |
| Free cholesterol in HDL | 0.46 (-0.39;1.30) | 4.54E-01 | 0.46 (-0.38;1.30) | 4.41E-01 | 0.46 (-0.39;1.30) | 4.54E-01 |
| Cholesterol to total lipids ratio in medium LDL | 0.38 (-0.32;1.07) | 4.54E-01 | 0.32 (-0.38;1.01) | 5.27E-01 | 0.38 (-0.32;1.07) | 4.54E-01 |
| Concentration of very large VLDL particles | -0.36 (-1.03;0.31) | 4.57E-01 | -0.38 (-1.04;0.28) | 4.12E-01 | -0.36 (-1.03;0.31) | 4.57E-01 |
| Cholesterol in medium VLDL | -0.59 (-1.72;0.53) | 4.62E-01 | -0.61 (-1.73;0.51) | 4.41E-01 | -0.59 (-1.72;0.53) | 4.62E-01 |
| Average diameter for LDL particles | 0.39 (-0.35;1.13) | 4.63E-01 | 0.41 (-0.33;1.14) | 4.35E-01 | 0.39 (-0.35;1.13) | 4.63E-01 |
| Free cholesterol in small VLDL | -0.52 (-1.51;0.47) | 4.63E-01 | -0.51 (-1.50;0.48) | 4.67E-01 | -0.52 (-1.51;0.47) | 4.63E-01 |
| Cholesterol to total lipids ratio in very large VLDL | 0.37 (-0.34;1.08) | 4.67E-01 | 0.42 (-0.28;1.13) | 3.94E-01 | 0.37 (-0.34;1.08) | 4.67E-01 |
| Total cholines | 0.59 (-0.55;1.73) | 4.71E-01 | 0.60 (-0.54;1.75) | 4.54E-01 | 0.59 (-0.55;1.73) | 4.71E-01 |
| Phospholipids to total lipids ratio in very large HDL | -0.36 (-1.07;0.35) | 4.79E-01 | -0.38 (-1.08;0.32) | 4.41E-01 | -0.36 (-1.07;0.35) | 4.79E-01 |
| Free cholesterol to total lipids ratio in medium HDL | 0.46 (-0.46;1.38) | 4.85E-01 | 0.46 (-0.46;1.38) | 4.77E-01 | 0.46 (-0.46;1.38) | 4.85E-01 |
| Cholesteryl esters in very large VLDL | -0.35 (-1.06;0.35) | 4.85E-01 | -0.37 (-1.07;0.34) | 4.60E-01 | -0.35 (-1.06;0.35) | 4.85E-01 |
| Cholesteryl esters in medium VLDL | -0.44 (-1.33;0.45) | 4.93E-01 | -0.40 (-1.28;0.48) | 5.30E-01 | -0.44 (-1.33;0.45) | 4.93E-01 |
| Saturated fatty acids | -0.41 (-1.23;0.42) | 4.94E-01 | -0.42 (-1.23;0.40) | 4.68E-01 | -0.41 (-1.23;0.42) | 4.94E-01 |
| Triglycerides to total lipids ratio in medium VLDL | -0.37 (-1.13;0.39) | 5.01E-01 | -0.41 (-1.16;0.35) | 4.41E-01 | -0.37 (-1.13;0.39) | 5.01E-01 |
| Apolipoprotein B | -0.59 (-1.81;0.64) | 5.02E-01 | -0.59 (-1.82;0.64) | 5.04E-01 | -0.59 (-1.81;0.64) | 5.02E-01 |
| Cholesteryl esters in IDL | 0.64 (-0.70;1.98) | 5.02E-01 | 0.62 (-0.70;1.95) | 5.15E-01 | 0.64 (-0.70;1.98) | 5.02E-01 |
| Free cholesterol to total lipids ratio in very large HDL | 0.35 (-0.39;1.09) | 5.02E-01 | 0.35 (-0.38;1.09) | 5.04E-01 | 0.35 (-0.39;1.09) | 5.02E-01 |
| Sphingomyelins | 0.63 (-0.71;1.96) | 5.10E-01 | 0.64 (-0.69;1.98) | 5.04E-01 | 0.63 (-0.71;1.96) | 5.10E-01 |
| Phosphoglycerides | 0.47 (-0.57;1.52) | 5.27E-01 | 0.48 (-0.56;1.52) | 5.17E-01 | 0.47 (-0.57;1.52) | 5.27E-01 |
| Phosphatidylcholines | 0.44 (-0.59;1.48) | 5.66E-01 | 0.46 (-0.58;1.49) | 5.43E-01 | 0.44 (-0.59;1.48) | 5.66E-01 |
| Free cholesterol to total lipids ratio in very small VLDL | -0.36 (-1.21;0.50) | 5.80E-01 | -0.39 (-1.25;0.46) | 5.20E-01 | -0.36 (-1.21;0.50) | 5.80E-01 |
| Phospholipids to total lipids ratio in small HDL | -0.29 (-0.99;0.41) | 5.86E-01 | -0.31 (-1.01;0.40) | 5.45E-01 | -0.29 (-0.99;0.41) | 5.86E-01 |
| Cholesterol in IDL | 0.54 (-0.80;1.89) | 5.93E-01 | 0.53 (-0.80;1.86) | 5.99E-01 | 0.54 (-0.80;1.89) | 5.93E-01 |
| Average diameter for HDL particles | -0.28 (-1.01;0.45) | 6.15E-01 | -0.27 (-1.00;0.45) | 6.25E-01 | -0.28 (-1.01;0.45) | 6.15E-01 |
| Free cholesterol to total lipids ratio in large LDL | 0.25 (-0.43;0.94) | 6.34E-01 | 0.24 (-0.43;0.91) | 6.39E-01 | 0.25 (-0.43;0.94) | 6.34E-01 |
| Concentration of small LDL particles | -0.41 (-1.51;0.70) | 6.34E-01 | -0.43 (-1.53;0.67) | 6.04E-01 | -0.41 (-1.51;0.70) | 6.34E-01 |
| Triglycerides in very large VLDL | -0.24 (-0.89;0.41) | 6.35E-01 | -0.28 (-0.93;0.37) | 5.55E-01 | -0.24 (-0.89;0.41) | 6.35E-01 |
| Cholesterol to total lipids ratio in medium VLDL | 0.26 (-0.46;0.98) | 6.41E-01 | 0.28 (-0.43;0.98) | 6.04E-01 | 0.26 (-0.46;0.98) | 6.41E-01 |
| Free cholesterol to total lipids ratio in very large VLDL | -0.23 (-0.88;0.42) | 6.48E-01 | -0.20 (-0.86;0.45) | 6.88E-01 | -0.23 (-0.88;0.42) | 6.48E-01 |
| Cholesterol in medium LDL | 0.38 (-0.70;1.46) | 6.49E-01 | 0.35 (-0.72;1.43) | 6.77E-01 | 0.38 (-0.70;1.46) | 6.49E-01 |
| Total phospholipids in lipoprotein particles | 0.42 (-0.87;1.71) | 6.86E-01 | 0.42 (-0.87;1.70) | 6.80E-01 | 0.42 (-0.87;1.71) | 6.86E-01 |
| Phospholipids in very large VLDL | -0.20 (-0.81;0.41) | 6.86E-01 | -0.22 (-0.83;0.39) | 6.39E-01 | -0.20 (-0.81;0.41) | 6.86E-01 |
| Total lipids in medium LDL | 0.35 (-0.75;1.44) | 6.94E-01 | 0.33 (-0.76;1.42) | 6.97E-01 | 0.35 (-0.75;1.44) | 6.94E-01 |
| Cholesteryl esters to total lipids ratio in small VLDL | 0.21 (-0.46;0.88) | 6.94E-01 | 0.24 (-0.42;0.91) | 6.36E-01 | 0.21 (-0.46;0.88) | 6.94E-01 |
| Phospholipids in large VLDL | -0.20 (-0.85;0.45) | 7.11E-01 | -0.20 (-0.85;0.45) | 6.92E-01 | -0.20 (-0.85;0.45) | 7.11E-01 |
| Concentration of medium LDL particles | -0.30 (-1.29;0.69) | 7.14E-01 | -0.31 (-1.30;0.68) | 6.88E-01 | -0.30 (-1.29;0.69) | 7.14E-01 |
| Phospholipids to total lipids ratio in IDL | -0.17 (-0.76;0.42) | 7.30E-01 | -0.16 (-0.75;0.44) | 7.45E-01 | -0.17 (-0.76;0.42) | 7.30E-01 |
| Phospholipids to total lipids ratio in medium VLDL | 0.22 (-0.57;1.00) | 7.48E-01 | 0.25 (-0.52;1.03) | 6.77E-01 | 0.22 (-0.57;1.00) | 7.48E-01 |
| Cholesteryl esters to total lipids ratio in small LDL | -0.22 (-1.03;0.59) | 7.50E-01 | -0.26 (-1.07;0.55) | 6.85E-01 | -0.22 (-1.03;0.59) | 7.50E-01 |
| Cholesteryl esters to total lipids ratio in large VLDL | 0.19 (-0.53;0.91) | 7.53E-01 | 0.24 (-0.48;0.95) | 6.77E-01 | 0.19 (-0.53;0.91) | 7.53E-01 |
| Cholesterol in small LDL | 0.31 (-0.87;1.50) | 7.53E-01 | 0.29 (-0.90;1.47) | 7.73E-01 | 0.31 (-0.87;1.50) | 7.53E-01 |
| Average diameter for VLDL particles | -0.18 (-0.85;0.49) | 7.53E-01 | -0.21 (-0.87;0.45) | 6.80E-01 | -0.18 (-0.85;0.49) | 7.53E-01 |
| Total concentration of branched-chain amino acids (leucine + isoleucine + valine) | 0.19 (-0.59;0.97) | 7.84E-01 | 0.21 (-0.55;0.97) | 7.20E-01 | 0.19 (-0.59;0.97) | 7.84E-01 |
| Cholesterol to total lipids ratio in large VLDL | 0.17 (-0.53;0.87) | 7.90E-01 | 0.21 (-0.48;0.91) | 6.89E-01 | 0.17 (-0.53;0.87) | 7.90E-01 |
| Omega-6 fatty acids | -0.25 (-1.32;0.82) | 7.90E-01 | -0.25 (-1.32;0.81) | 7.80E-01 | -0.25 (-1.32;0.82) | 7.90E-01 |
| Leucine | 0.18 (-0.62;0.98) | 8.04E-01 | 0.22 (-0.56;1.01) | 7.17E-01 | 0.18 (-0.62;0.98) | 8.04E-01 |
| Cholesteryl esters in large HDL | 0.13 (-0.49;0.74) | 8.36E-01 | 0.12 (-0.50;0.73) | 8.39E-01 | 0.13 (-0.49;0.74) | 8.36E-01 |
| Phospholipids to total lipids ratio in large VLDL | 0.11 (-0.45;0.66) | 8.45E-01 | 0.14 (-0.42;0.69) | 7.65E-01 | 0.11 (-0.45;0.66) | 8.45E-01 |
| Phospholipids to total lipids ratio in small LDL | -0.18 (-1.08;0.73) | 8.45E-01 | -0.15 (-1.05;0.75) | 8.52E-01 | -0.18 (-1.08;0.73) | 8.45E-01 |
| Phospholipids to total lipids ratio in large LDL | 0.13 (-0.56;0.81) | 8.54E-01 | 0.14 (-0.54;0.82) | 8.19E-01 | 0.13 (-0.56;0.81) | 8.54E-01 |
| Acetone | -0.11 (-0.75;0.53) | 8.59E-01 | -0.14 (-0.79;0.50) | 8.05E-01 | -0.11 (-0.75;0.53) | 8.59E-01 |
| Free cholesterol to total lipids ratio in IDL | 0.14 (-0.63;0.92) | 8.59E-01 | 0.13 (-0.64;0.89) | 8.52E-01 | 0.14 (-0.63;0.92) | 8.59E-01 |
| Cholesteryl esters to total lipids ratio in large HDL | 0.09 (-0.44;0.62) | 8.59E-01 | 0.08 (-0.45;0.61) | 8.52E-01 | 0.09 (-0.44;0.62) | 8.59E-01 |
| Total lipids in large HDL | 0.13 (-0.62;0.89) | 8.59E-01 | 0.12 (-0.63;0.87) | 8.52E-01 | 0.13 (-0.62;0.89) | 8.59E-01 |
| Lactate | 0.10 (-0.51;0.71) | 8.59E-01 | 0.11 (-0.50;0.71) | 8.52E-01 | 0.10 (-0.51;0.71) | 8.59E-01 |
| Concentration of LDL particles | -0.20 (-1.43;1.02) | 8.59E-01 | -0.21 (-1.43;1.01) | 8.52E-01 | -0.20 (-1.43;1.02) | 8.59E-01 |
| Free cholesterol to total lipids ratio in medium VLDL | 0.14 (-0.65;0.93) | 8.59E-01 | 0.18 (-0.60;0.95) | 7.96E-01 | 0.14 (-0.65;0.93) | 8.59E-01 |
| Total lipids in lipoprotein particles | -0.21 (-1.42;0.99) | 8.59E-01 | -0.24 (-1.43;0.96) | 8.28E-01 | -0.21 (-1.42;0.99) | 8.59E-01 |
| Free cholesterol in IDL | 0.20 (-1.08;1.47) | 8.73E-01 | 0.20 (-1.06;1.46) | 8.52E-01 | 0.20 (-1.08;1.47) | 8.73E-01 |
| Cholesterol to total lipids ratio in large HDL | -0.08 (-0.66;0.50) | 8.88E-01 | -0.09 (-0.67;0.48) | 8.52E-01 | -0.08 (-0.66;0.50) | 8.88E-01 |
| Phospholipids in large HDL | 0.10 (-0.61;0.81) | 8.89E-01 | 0.08 (-0.63;0.79) | 8.84E-01 | 0.10 (-0.61;0.81) | 8.89E-01 |
| Free cholesterol to total lipids ratio in large VLDL | 0.08 (-0.54;0.69) | 8.98E-01 | 0.11 (-0.50;0.73) | 8.47E-01 | 0.08 (-0.54;0.69) | 8.98E-01 |
| Ratio of linoleic acid to total fatty acids | 0.10 (-0.67;0.87) | 8.98E-01 | 0.12 (-0.64;0.88) | 8.52E-01 | 0.10 (-0.67;0.87) | 8.98E-01 |
| Total cholesterol minus HDL-C | 0.19 (-1.27;1.65) | 8.98E-01 | 0.17 (-1.29;1.63) | 8.84E-01 | 0.19 (-1.27;1.65) | 8.98E-01 |
| Acetate | 0.09 (-0.70;0.88) | 9.00E-01 | 0.10 (-0.69;0.89) | 8.80E-01 | 0.09 (-0.70;0.88) | 9.00E-01 |
| Alanine | 0.07 (-0.59;0.74) | 9.00E-01 | 0.08 (-0.57;0.74) | 8.80E-01 | 0.07 (-0.59;0.74) | 9.00E-01 |
| Histidine | 0.08 (-0.62;0.78) | 9.00E-01 | 0.09 (-0.61;0.79) | 8.80E-01 | 0.08 (-0.62;0.78) | 9.00E-01 |
| Triglycerides to total lipids ratio in large VLDL | -0.08 (-0.77;0.61) | 9.00E-01 | -0.12 (-0.81;0.57) | 8.52E-01 | -0.08 (-0.77;0.61) | 9.00E-01 |
| Cholesteryl esters in medium LDL | 0.11 (-0.87;1.09) | 9.00E-01 | 0.09 (-0.89;1.06) | 9.23E-01 | 0.11 (-0.87;1.09) | 9.00E-01 |
| Cholesteryl esters to total lipids ratio in very large HDL | 0.11 (-0.87;1.10) | 9.00E-01 | 0.21 (-0.76;1.18) | 8.07E-01 | 0.11 (-0.87;1.10) | 9.00E-01 |
| Polyunsaturated fatty acids | 0.11 (-0.91;1.13) | 9.00E-01 | 0.12 (-0.90;1.13) | 8.84E-01 | 0.11 (-0.91;1.13) | 9.00E-01 |
| Ratio of saturated fatty acids to total fatty acids | 0.07 (-0.60;0.74) | 9.00E-01 | 0.05 (-0.62;0.72) | 9.33E-01 | 0.07 (-0.60;0.74) | 9.00E-01 |
| Total lipids in IDL | 0.14 (-1.27;1.55) | 9.05E-01 | 0.16 (-1.24;1.56) | 8.84E-01 | 0.14 (-1.27;1.55) | 9.05E-01 |
| Free cholesterol to total lipids ratio in large HDL | -0.05 (-0.68;0.59) | 9.36E-01 | -0.08 (-0.71;0.55) | 8.80E-01 | -0.05 (-0.68;0.59) | 9.36E-01 |
| Cholesteryl esters to total lipids ratio in medium VLDL | 0.05 (-0.58;0.68) | 9.36E-01 | 0.08 (-0.54;0.70) | 8.80E-01 | 0.05 (-0.58;0.68) | 9.36E-01 |
| Total free cholesterol | -0.14 (-1.91;1.63) | 9.36E-01 | -0.15 (-1.92;1.62) | 9.24E-01 | -0.14 (-1.91;1.63) | 9.36E-01 |
| Free cholesterol in large HDL | 0.04 (-0.66;0.73) | 9.64E-01 | 0.01 (-0.68;0.70) | 9.85E-01 | 0.04 (-0.66;0.73) | 9.64E-01 |
| Phospholipids to total lipids ratio in very large VLDL | -0.03 (-0.53;0.47) | 9.64E-01 | -0.02 (-0.52;0.48) | 9.76E-01 | -0.03 (-0.53;0.47) | 9.64E-01 |
| Total lipids in small LDL | 0.06 (-1.12;1.23) | 9.65E-01 | 0.03 (-1.13;1.20) | 9.84E-01 | 0.06 (-1.12;1.23) | 9.65E-01 |
| Triglycerides to total lipids ratio in very large VLDL | 0.03 (-0.76;0.82) | 9.72E-01 | -0.02 (-0.80;0.77) | 9.84E-01 | 0.03 (-0.76;0.82) | 9.72E-01 |
| Acetoacetate | -0.01 (-0.64;0.62) | 9.84E-01 | -0.02 (-0.64;0.61) | 9.84E-01 | -0.01 (-0.64;0.62) | 9.84E-01 |
| Concentration of large HDL particles | -0.01 (-0.71;0.69) | 9.84E-01 | -0.02 (-0.72;0.67) | 9.84E-01 | -0.01 (-0.71;0.69) | 9.84E-01 |
| Phospholipids to total lipids ratio in large HDL | 0.02 (-0.65;0.68) | 9.84E-01 | -0.01 (-0.67;0.66) | 9.89E-01 | 0.02 (-0.65;0.68) | 9.84E-01 |
| Concentration of large LDL particles | 0.02 (-1.26;1.30) | 9.84E-01 | 0.02 (-1.26;1.31) | 9.84E-01 | 0.02 (-1.26;1.30) | 9.84E-01 |
| Cholesteryl esters in small LDL | -0.02 (-1.14;1.09) | 9.84E-01 | -0.05 (-1.17;1.06) | 9.76E-01 | -0.02 (-1.14;1.09) | 9.84E-01 |
| Phospholipids in small LDL | -0.02 (-1.26;1.22) | 9.84E-01 | -0.03 (-1.27;1.21) | 9.84E-01 | -0.02 (-1.26;1.22) | 9.84E-01 |
| Cholesterol in large HDL | 0.01 (-0.66;0.67) | 9.88E-01 | 0.00 (-0.66;0.66) | 9.89E-01 | 0.01 (-0.66;0.67) | 9.88E-01 |
| Phospholipids in IDL | 0.00 (-1.30;1.30) | 9.99E-01 | 0.03 (-1.26;1.32) | 9.84E-01 | 0.00 (-1.30;1.30) | 9.99E-01 |

Model 1 is adjusted for age, sex, and Rotterdam Study Cohort

Model 2 is additionally adjusted for body mass index, smoking status, alcohol use, serum cholesterol, lipid-lowering drugs, and prevalent cardiovascular disease

Model 3 is additionally adjusted for hypertension and diabetes

All serum metabolites are log-transformed.

* P-values are FDR-corrected

Abbreviations: CI = confidence interval; eGFRcys = estimated glomerular filtration rate based on serum cystatin C; HDL = high-density lipoprotein; IDL = intermediate-density lipoprotein; LDL = low-density lipoprotein; VLDL = very low-density lipoprotein.

**Supplemental Table 4.** Association between serum metabolites from the Metabolon platform and eGFRcys at baseline (n = 1,452)

|  | **Model 1** |  | **Model 2** |  | **Model 3** |  |
| --- | --- | --- | --- | --- | --- | --- |
| **Metabolite** | **Beta (95% CI)** | **P-value** | **Beta (95% CI)** | **P-value** | **Beta (95% CI)** | **P-value** |
| 3-(3-Amino-3-Carboxypropyl)Uridine* | -4.87 (-5.66;-4.07) | 3.52E-30 | -5.47 (-6.10;-4.84) | 8.10E-62 | -5.48 (-6.11;-4.85) | 1.38E-61 |
| C-Glycosyltryptophan | -4.23 (-5.02;-3.43) | 6.29E-23 | -4.93 (-5.57;-4.30) | 3.81E-49 | -4.93 (-5.57;-4.29) | 5.88E-49 |
| 2,3-Dihydroxy-5-Methylthio-4-Pentenoate (DMPTA)* | -4.26 (-5.08;-3.44) | 5.24E-22 | -5.13 (-5.80;-4.47) | 9.66E-49 | -5.15 (-5.82;-4.48) | 5.94E-49 |
| Pseudouridine | -4.58 (-5.36;-3.79) | 8.98E-28 | -4.79 (-5.41;-4.16) | 2.27E-48 | -4.82 (-5.45;-4.19) | 1.47E-48 |
| X-12026 | -3.69 (-4.50;-2.88) | 5.10E-17 | -4.85 (-5.52;-4.18) | 1.87E-43 | -4.91 (-5.58;-4.23) | 8.21E-44 |
| Hydroxyasparagine** | -2.50 (-3.31;-1.69) | 2.79E-08 | -4.57 (-5.20;-3.93) | 7.74E-43 | -4.57 (-5.21;-3.94) | 1.00E-42 |
| Quinolinate | -3.34 (-4.14;-2.53) | 3.17E-14 | -4.54 (-5.19;-3.89) | 2.67E-40 | -4.55 (-5.20;-3.90) | 2.94E-40 |
| 5,6-Dihydrouridine | -4.18 (-4.97;-3.39) | 6.29E-23 | -4.37 (-5.01;-3.73) | 8.04E-39 | -4.37 (-5.01;-3.73) | 9.88E-39 |
| X-12100 | -2.60 (-3.41;-1.79) | 6.82E-09 | -4.09 (-4.73;-3.45) | 8.90E-34 | -4.09 (-4.73;-3.45) | 1.18E-33 |
| S-Adenosylhomocysteine (SAH) | -3.45 (-4.28;-2.62) | 2.94E-14 | -4.28 (-4.95;-3.60) | 3.85E-33 | -4.31 (-4.99;-3.63) | 2.49E-33 |
| X-25422 | -3.39 (-4.20;-2.57) | 2.37E-14 | -3.98 (-4.63;-3.32) | 4.46E-31 | -3.98 (-4.63;-3.33) | 5.20E-31 |
| Orotidine | -3.48 (-4.29;-2.67) | 4.27E-15 | -3.98 (-4.64;-3.33) | 1.35E-30 | -4.02 (-4.68;-3.36) | 6.81E-31 |
| N-Acetylserine | -2.53 (-3.34;-1.72) | 1.70E-08 | -3.86 (-4.50;-3.22) | 6.48E-30 | -3.87 (-4.52;-3.22) | 6.36E-30 |
| N-Formylmethionine | -3.27 (-4.08;-2.46) | 1.26E-13 | -3.84 (-4.49;-3.19) | 4.36E-29 | -3.87 (-4.52;-3.21) | 2.75E-29 |
| X-12117 | -4.11 (-4.91;-3.31) | 1.15E-21 | -3.93 (-4.60;-3.27) | 3.31E-29 | -3.95 (-4.61;-3.28) | 3.48E-29 |
| X-15503 | -3.36 (-4.18;-2.53) | 9.80E-14 | -4.07 (-4.76;-3.37) | 9.51E-29 | -4.11 (-4.80;-3.41) | 4.12E-29 |
| X-17676 | -3.93 (-4.73;-3.13) | 9.04E-20 | -3.80 (-4.46;-3.15) | 2.68E-28 | -3.81 (-4.46;-3.16) | 2.60E-28 |
| Vanillactate | -3.14 (-3.96;-2.33) | 1.75E-12 | -3.63 (-4.28;-2.99) | 2.79E-26 | -3.67 (-4.32;-3.01) | 1.67E-26 |
| Erythritol | -2.97 (-3.79;-2.15) | 3.92E-11 | -3.63 (-4.28;-2.98) | 3.36E-26 | -3.64 (-4.29;-2.99) | 3.54E-26 |
| Arabitol/Xylitol | -2.77 (-3.58;-1.95) | 8.96E-10 | -3.59 (-4.24;-2.94) | 1.22E-25 | -3.60 (-4.25;-2.95) | 1.05E-25 |
| N2,N2-Dimethylguanosine | -2.48 (-3.30;-1.66) | 4.71E-08 | -3.60 (-4.25;-2.95) | 1.32E-25 | -3.60 (-4.25;-2.95) | 1.59E-25 |
| Kynurenine | -2.55 (-3.37;-1.73) | 1.67E-08 | -3.60 (-4.26;-2.94) | 3.70E-25 | -3.61 (-4.27;-2.95) | 3.50E-25 |
| Glutamine_Degradant* | -3.35 (-4.30;-2.40) | 1.47E-10 | -4.17 (-4.93;-3.40) | 5.46E-25 | -4.21 (-4.98;-3.44) | 3.50E-25 |
| O-Sulfo-L-Tyrosine | -2.73 (-3.54;-1.92) | 8.96E-10 | -3.51 (-4.15;-2.87) | 3.18E-25 | -3.51 (-4.16;-2.87) | 3.60E-25 |
| X-25790 | -2.87 (-3.72;-2.02) | 8.96E-10 | -3.77 (-4.46;-3.08) | 4.22E-25 | -3.78 (-4.47;-3.09) | 4.72E-25 |
| X-25371 | -2.87 (-3.68;-2.06) | 1.44E-10 | -3.58 (-4.24;-2.91) | 1.93E-24 | -3.58 (-4.25;-2.92) | 1.93E-24 |
| N-Carbamoylvaline | -3.08 (-3.90;-2.26) | 9.49E-12 | -3.53 (-4.19;-2.86) | 1.09E-23 | -3.53 (-4.20;-2.86) | 1.22E-23 |
| Erythronate* | -2.12 (-2.94;-1.30) | 3.89E-06 | -3.40 (-4.04;-2.75) | 4.43E-23 | -3.45 (-4.10;-2.80) | 1.66E-23 |
| N-Acetylmethionine | -3.45 (-4.25;-2.64) | 4.27E-15 | -3.45 (-4.11;-2.80) | 2.00E-23 | -3.46 (-4.12;-2.81) | 1.98E-23 |
| Creatinine | -2.24 (-3.11;-1.37) | 4.81E-06 | -3.72 (-4.42;-3.01) | 3.07E-23 | -3.74 (-4.45;-3.03) | 2.20E-23 |
| Homocitrulline | -3.21 (-4.02;-2.41) | 2.27E-13 | -3.43 (-4.09;-2.78) | 3.07E-23 | -3.45 (-4.10;-2.79) | 2.37E-23 |
| N-Acetylalanine | -1.79 (-2.61;-0.98) | 1.12E-04 | -3.37 (-4.01;-2.72) | 4.90E-23 | -3.38 (-4.03;-2.74) | 3.67E-23 |
| Hydroxy-N6,N6,N6-Trimethyllysine* | -3.04 (-3.86;-2.22) | 1.22E-11 | -3.41 (-4.06;-2.76) | 4.36E-23 | -3.42 (-4.08;-2.77) | 4.35E-23 |
| N1-Methylinosine | -3.69 (-4.53;-2.86) | 4.10E-16 | -3.51 (-4.19;-2.84) | 9.91E-23 | -3.55 (-4.23;-2.87) | 5.29E-23 |
| X-13553 | -2.72 (-3.54;-1.89) | 2.75E-09 | -3.45 (-4.12;-2.78) | 1.93E-22 | -3.45 (-4.13;-2.78) | 2.24E-22 |
| N-Acetylneuraminate | -1.80 (-2.61;-0.98) | 1.06E-04 | -3.24 (-3.89;-2.59) | 4.55E-21 | -3.24 (-3.89;-2.59) | 5.68E-21 |
| N,N,N-Trimethyl-Alanylproline Betaine (Tmap) | -1.46 (-2.30;-0.62) | 2.83E-03 | -3.30 (-3.98;-2.63) | 3.57E-20 | -3.32 (-4.00;-2.64) | 2.83E-20 |
| Methionine Sulfone | -2.25 (-3.08;-1.42) | 1.21E-06 | -3.22 (-3.88;-2.56) | 5.38E-20 | -3.24 (-3.91;-2.58) | 4.18E-20 |
| N-Carbamoylalanine | -2.83 (-3.64;-2.02) | 2.11E-10 | -3.17 (-3.82;-2.52) | 3.55E-20 | -3.17 (-3.82;-2.51) | 4.39E-20 |
| X-12906 | -2.70 (-3.51;-1.88) | 2.07E-09 | -3.16 (-3.81;-2.51) | 5.93E-20 | -3.17 (-3.82;-2.52) | 5.12E-20 |
| N-Acetylvaline | -2.72 (-3.54;-1.90) | 1.65E-09 | -3.18 (-3.84;-2.51) | 2.13E-19 | -3.21 (-3.88;-2.54) | 1.22E-19 |
| N,N-Dimethyl-Pro-Pro | -1.04 (-1.86;-0.21) | 3.75E-02 | -3.19 (-3.86;-2.52) | 1.81E-19 | -3.20 (-3.87;-2.53) | 1.77E-19 |
| Vanillylmandelate (VMA) | -2.24 (-3.07;-1.41) | 1.36E-06 | -3.22 (-3.90;-2.54) | 5.45E-19 | -3.26 (-3.95;-2.57) | 3.75E-19 |
| N-Acetylglucosaminylasparagine | -2.58 (-3.40;-1.76) | 1.35E-08 | -3.14 (-3.80;-2.48) | 3.04E-19 | -3.14 (-3.80;-2.48) | 3.89E-19 |
| N6-Carbamoylthreonyladenosine | -2.45 (-3.28;-1.62) | 9.34E-08 | -3.11 (-3.78;-2.45) | 6.09E-19 | -3.13 (-3.79;-2.46) | 5.99E-19 |
| 3,4-Dihydroxybutyrate | -2.44 (-3.26;-1.63) | 6.77E-08 | -3.06 (-3.72;-2.40) | 2.61E-18 | -3.12 (-3.79;-2.46) | 7.88E-19 |
| X-24334 | -2.56 (-3.42;-1.70) | 7.18E-08 | -3.12 (-3.81;-2.43) | 2.44E-17 | -3.31 (-4.01;-2.60) | 1.19E-18 |
| 5-Methylthioadenosine (Mta) | -2.68 (-3.49;-1.87) | 2.44E-09 | -3.09 (-3.76;-2.42) | 2.36E-18 | -3.10 (-3.77;-2.43) | 1.98E-18 |
| N-Acetylthreonine | -2.30 (-3.11;-1.48) | 4.27E-07 | -3.02 (-3.67;-2.37) | 2.44E-18 | -3.02 (-3.68;-2.37) | 2.46E-18 |
| Dimethylarginine (SDMA + ADMA) | -1.12 (-1.94;-0.30) | 2.13E-02 | -3.01 (-3.66;-2.36) | 2.44E-18 | -3.02 (-3.67;-2.37) | 2.87E-18 |
| X-23659 | -2.35 (-3.18;-1.52) | 3.19E-07 | -3.09 (-3.75;-2.42) | 3.23E-18 | -3.10 (-3.77;-2.43) | 2.87E-18 |
| N2,N5-Diacetylornithine | -1.98 (-2.81;-1.16) | 2.11E-05 | -3.06 (-3.73;-2.39) | 7.06E-18 | -3.07 (-3.74;-2.40) | 7.54E-18 |
| Glutamine Conjugate Of C6h10o2 (1)* | -3.27 (-4.09;-2.45) | 1.99E-13 | -2.99 (-3.66;-2.33) | 2.90E-17 | -3.07 (-3.74;-2.39) | 1.05E-17 |
| Arabonate/Xylonate | -1.88 (-2.69;-1.07) | 4.36E-05 | -2.87 (-3.52;-2.23) | 8.16E-17 | -2.88 (-3.53;-2.23) | 8.42E-17 |
| N-Acetylhistidine | -1.91 (-2.72;-1.09) | 3.64E-05 | -2.92 (-3.58;-2.25) | 1.36E-16 | -2.92 (-3.58;-2.25) | 1.54E-16 |
| Gamma-Glutamylphenylalanine | -2.52 (-3.35;-1.69) | 4.67E-08 | -2.93 (-3.61;-2.26) | 3.38E-16 | -2.95 (-3.63;-2.27) | 2.95E-16 |
| Kynurenate | -2.64 (-3.47;-1.81) | 1.02E-08 | -2.94 (-3.63;-2.26) | 5.46E-16 | -2.96 (-3.64;-2.27) | 4.59E-16 |
| 1-Ribosyl-Imidazoleacetate* | -2.04 (-2.87;-1.22) | 1.21E-05 | -2.84 (-3.51;-2.18) | 7.76E-16 | -2.85 (-3.51;-2.19) | 7.90E-16 |
| X-24337 | -3.39 (-4.21;-2.57) | 3.23E-14 | -2.90 (-3.58;-2.22) | 9.31E-16 | -2.90 (-3.58;-2.22) | 1.06E-15 |
| 4-Hydroxyphenylacetylglutamine | -2.96 (-3.79;-2.14) | 8.28E-11 | -2.87 (-3.54;-2.19) | 1.53E-15 | -2.89 (-3.57;-2.22) | 1.08E-15 |
| X-23593 | -2.03 (-2.85;-1.21) | 9.86E-06 | -2.78 (-3.43;-2.12) | 1.82E-15 | -2.78 (-3.44;-2.13) | 2.00E-15 |
| Pimeloylcarnitine/3-Methyladipoylcarnitine (C7-Dc) | -1.87 (-2.68;-1.06) | 4.66E-05 | -2.74 (-3.39;-2.08) | 4.73E-15 | -2.77 (-3.43;-2.11) | 3.89E-15 |
| Adipoylcarnitine (C6-DC) | -2.79 (-3.60;-1.98) | 5.04E-10 | -2.74 (-3.41;-2.08) | 1.03E-14 | -2.77 (-3.43;-2.10) | 8.26E-15 |
| 5-(Galactosylhydroxy)-L-Lysine | -2.90 (-3.71;-2.09) | 8.98E-11 | -2.73 (-3.39;-2.07) | 8.84E-15 | -2.73 (-3.39;-2.07) | 1.01E-14 |
| Guaiacol Sulfate | -2.94 (-3.76;-2.12) | 8.98E-11 | -2.71 (-3.39;-2.04) | 5.19E-14 | -2.71 (-3.39;-2.04) | 5.77E-14 |
| 1-Methyl-4-Imidazoleacetate | -2.32 (-3.13;-1.51) | 2.88E-07 | -2.63 (-3.29;-1.97) | 8.37E-14 | -2.63 (-3.29;-1.97) | 8.74E-14 |
| 2-O-Methylascorbic Acid | -2.58 (-3.39;-1.77) | 9.15E-09 | -2.62 (-3.28;-1.96) | 1.23E-13 | -2.63 (-3.29;-1.96) | 1.26E-13 |
| X-13507 | -2.18 (-2.99;-1.37) | 1.67E-06 | -2.58 (-3.24;-1.93) | 1.88E-13 | -2.59 (-3.25;-1.93) | 1.84E-13 |
| Succinylcarnitine (C4-DC) | -1.94 (-2.76;-1.11) | 3.40E-05 | -2.61 (-3.28;-1.95) | 2.16E-13 | -2.62 (-3.29;-1.95) | 2.18E-13 |
| 7-Methylguanine | -1.75 (-2.57;-0.92) | 2.17E-04 | -2.62 (-3.29;-1.95) | 1.91E-13 | -2.62 (-3.28;-1.95) | 2.38E-13 |
| Picolinoylglycine | -2.68 (-3.54;-1.83) | 1.29E-08 | -2.72 (-3.43;-2.02) | 5.15E-13 | -2.75 (-3.46;-2.05) | 3.20E-13 |
| 3-Hydroxyhexanoylcarnitine (1) | -2.69 (-3.52;-1.87) | 3.44E-09 | -2.56 (-3.23;-1.88) | 1.73E-12 | -2.61 (-3.29;-1.93) | 9.59E-13 |
| Sulfate* | -1.45 (-2.26;-0.64) | 2.12E-03 | -2.46 (-3.11;-1.82) | 9.97E-13 | -2.46 (-3.11;-1.82) | 1.07E-12 |
| Methylsuccinoylcarnitine | -2.32 (-3.13;-1.51) | 2.64E-07 | -2.47 (-3.13;-1.82) | 2.42E-12 | -2.50 (-3.16;-1.84) | 1.88E-12 |
| X-12822 | -1.69 (-2.52;-0.86) | 3.66E-04 | -2.52 (-3.19;-1.85) | 2.45E-12 | -2.53 (-3.20;-1.86) | 2.35E-12 |
| X-18886 | -2.76 (-3.63;-1.90) | 7.99E-09 | -2.65 (-3.37;-1.94) | 4.44E-12 | -2.71 (-3.43;-1.99) | 2.35E-12 |
| X-13844 | -2.58 (-3.40;-1.76) | 1.35E-08 | -2.51 (-3.18;-1.84) | 2.42E-12 | -2.51 (-3.18;-1.84) | 2.53E-12 |
| Hydantoin-5-Propionate | -2.05 (-2.86;-1.23) | 7.88E-06 | -2.52 (-3.20;-1.85) | 3.56E-12 | -2.54 (-3.22;-1.86) | 2.69E-12 |
| 3-Methylglutarylcarnitine (2) | -2.58 (-3.40;-1.76) | 1.29E-08 | -2.58 (-3.27;-1.89) | 3.22E-12 | -2.58 (-3.28;-1.89) | 3.37E-12 |
| 3-Amino-2-Piperidone | -1.90 (-2.73;-1.08) | 4.51E-05 | -2.46 (-3.13;-1.79) | 6.77E-12 | -2.46 (-3.13;-1.79) | 8.11E-12 |
| X-24518 | -3.31 (-4.13;-2.49) | 1.22E-13 | -2.51 (-3.19;-1.82) | 1.13E-11 | -2.51 (-3.20;-1.82) | 1.25E-11 |
| N-Acetylglucosamine/N-Acetylgalactosamine | -2.16 (-2.97;-1.35) | 2.04E-06 | -2.38 (-3.03;-1.72) | 1.63E-11 | -2.39 (-3.05;-1.74) | 1.37E-11 |
| O-Cresol Sulfate | -2.97 (-3.78;-2.16) | 2.99E-11 | -2.82 (-3.60;-2.05) | 1.47E-11 | -2.83 (-3.61;-2.05) | 1.48E-11 |
| 3-Aminoisobutyrate | -1.77 (-2.59;-0.94) | 1.72E-04 | -2.40 (-3.06;-1.73) | 1.93E-11 | -2.41 (-3.07;-1.74) | 1.77E-11 |
| X-22162 | -1.36 (-2.18;-0.55) | 4.45E-03 | -2.35 (-3.00;-1.70) | 2.47E-11 | -2.36 (-3.02;-1.70) | 2.36E-11 |
| 2-Methylbutyrylcarnitine (C5) | -2.25 (-3.09;-1.41) | 1.63E-06 | -2.42 (-3.10;-1.73) | 5.87E-11 | -2.47 (-3.16;-1.78) | 2.81E-11 |
| X-12283 | -1.69 (-2.50;-0.87) | 2.94E-04 | -2.38 (-3.04;-1.71) | 2.68E-11 | -2.38 (-3.05;-1.71) | 3.05E-11 |
| Gamma-Glutamylisoleucine* | -2.30 (-3.15;-1.44) | 1.76E-06 | -2.49 (-3.20;-1.79) | 5.87E-11 | -2.54 (-3.25;-1.83) | 3.46E-11 |
| Indoleacetylglutamine | -2.46 (-3.29;-1.62) | 1.18E-07 | -2.36 (-3.04;-1.69) | 7.76E-11 | -2.38 (-3.06;-1.71) | 5.81E-11 |
| X-12411 | -2.20 (-3.04;-1.36) | 2.77E-06 | -2.37 (-3.04;-1.69) | 7.00E-11 | -2.38 (-3.06;-1.71) | 6.18E-11 |
| 11-Ketoetiocholanolone Glucuronide | -2.04 (-2.87;-1.22) | 9.76E-06 | -2.26 (-2.92;-1.60) | 2.02E-10 | -2.30 (-2.96;-1.64) | 1.26E-10 |
| Myo-Inositol | -1.78 (-2.59;-0.96) | 1.27E-04 | -2.26 (-2.91;-1.61) | 1.43E-10 | -2.27 (-2.92;-1.61) | 1.30E-10 |
| 3-Methoxycatechol Sulfate (2) | -2.34 (-3.16;-1.52) | 3.14E-07 | -2.31 (-2.97;-1.64) | 1.18E-10 | -2.30 (-2.97;-1.64) | 1.37E-10 |
| 11beta-Hydroxyandrosterone Glucuronide | -2.72 (-3.54;-1.89) | 2.48E-09 | -2.27 (-2.94;-1.61) | 2.56E-10 | -2.33 (-3.00;-1.65) | 1.62E-10 |
| N-Acetyl-1-Methylhistidine* | -2.04 (-2.86;-1.23) | 9.01E-06 | -2.28 (-2.94;-1.61) | 1.98E-10 | -2.28 (-2.95;-1.62) | 1.83E-10 |
| X-18887 | -2.01 (-2.82;-1.19) | 1.23E-05 | -2.28 (-2.95;-1.61) | 2.45E-10 | -2.31 (-2.99;-1.64) | 2.23E-10 |
| N-Acetylcarnosine | -2.27 (-3.24;-1.29) | 4.05E-05 | -2.63 (-3.41;-1.85) | 4.10E-10 | -2.68 (-3.47;-1.90) | 2.35E-10 |
| 3-Methyl Catechol Sulfate (1) | -2.87 (-3.68;-2.05) | 1.61E-10 | -2.33 (-3.01;-1.65) | 2.38E-10 | -2.32 (-3.01;-1.64) | 2.73E-10 |
| X-21821 | -1.65 (-2.46;-0.83) | 4.10E-04 | -2.25 (-2.91;-1.59) | 2.85E-10 | -2.25 (-2.91;-1.58) | 3.32E-10 |
| 1-Methylhistidine | -2.73 (-3.54;-1.91) | 1.35E-09 | -2.67 (-3.46;-1.87) | 4.47E-10 | -2.67 (-3.46;-1.87) | 4.53E-10 |
| 3-Hydroxyphenylacetoylglutamine | -2.01 (-2.82;-1.20) | 1.05E-05 | -2.19 (-2.85;-1.54) | 5.01E-10 | -2.19 (-2.85;-1.54) | 5.41E-10 |
| X-24556 | -1.34 (-2.16;-0.52) | 5.41E-03 | -2.21 (-2.87;-1.55) | 5.58E-10 | -2.22 (-2.88;-1.56) | 5.41E-10 |
| N-Acetyl-2-Aminoadipate | -2.31 (-3.12;-1.50) | 3.15E-07 | -2.26 (-2.94;-1.58) | 6.65E-10 | -2.27 (-2.95;-1.59) | 5.92E-10 |
| Homovanillate (HVA) | -2.06 (-2.87;-1.25) | 7.01E-06 | -2.17 (-2.83;-1.52) | 7.50E-10 | -2.19 (-2.84;-1.53) | 6.47E-10 |
| Cystathionine | -2.33 (-3.15;-1.51) | 3.19E-07 | -2.23 (-2.90;-1.56) | 6.75E-10 | -2.23 (-2.90;-1.56) | 7.30E-10 |
| Glutamine Conjugate Of C7h12o2* | -2.48 (-3.31;-1.66) | 5.26E-08 | -2.24 (-2.91;-1.56) | 8.35E-10 | -2.25 (-2.93;-1.58) | 7.51E-10 |
| 3-Hydroxy-3-Methylglutarate | -1.99 (-2.81;-1.18) | 1.49E-05 | -2.28 (-2.97;-1.59) | 9.85E-10 | -2.30 (-2.99;-1.60) | 8.60E-10 |
| X-12707 | -1.36 (-2.18;-0.54) | 4.48E-03 | -2.22 (-2.89;-1.55) | 8.27E-10 | -2.21 (-2.88;-1.54) | 9.76E-10 |
| X-17685 | -1.87 (-2.68;-1.05) | 5.65E-05 | -2.17 (-2.82;-1.51) | 1.03E-09 | -2.17 (-2.83;-1.51) | 1.10E-09 |
| Acisoga | -2.60 (-3.41;-1.79) | 5.84E-09 | -2.15 (-2.80;-1.49) | 1.25E-09 | -2.16 (-2.81;-1.50) | 1.17E-09 |
| Glutamine Conjugate Of C6h10o2 (2)* | -2.59 (-3.42;-1.77) | 1.17E-08 | -2.20 (-2.88;-1.53) | 1.74E-09 | -2.24 (-2.93;-1.56) | 1.20E-09 |
| N-Acetyltryptophan | -2.05 (-2.91;-1.18) | 2.72E-05 | -2.31 (-3.03;-1.60) | 1.74E-09 | -2.33 (-3.05;-1.62) | 1.46E-09 |
| 3-Methoxytyramine Sulfate | -1.91 (-2.72;-1.09) | 3.42E-05 | -2.13 (-2.78;-1.48) | 1.60E-09 | -2.13 (-2.79;-1.48) | 1.52E-09 |
| Adenosine 3',5'-Cyclic Monophosphate (Camp) | -1.95 (-2.76;-1.13) | 2.41E-05 | -2.13 (-2.79;-1.47) | 2.19E-09 | -2.15 (-2.81;-1.49) | 1.70E-09 |
| N4-Acetylcytidine | -2.06 (-2.87;-1.25) | 6.86E-06 | -2.15 (-2.81;-1.49) | 1.89E-09 | -2.16 (-2.82;-1.49) | 1.77E-09 |
| 1-Methyl-5-Imidazoleacetate | -2.11 (-2.94;-1.29) | 5.42E-06 | -2.16 (-2.83;-1.50) | 1.91E-09 | -2.17 (-2.84;-1.50) | 1.80E-09 |
| X-12680 | -1.76 (-2.57;-0.95) | 1.38E-04 | -2.15 (-2.81;-1.49) | 1.91E-09 | -2.15 (-2.81;-1.48) | 2.13E-09 |
| N1-Methyl-2-Pyridone-5-Carboxamide | -1.72 (-2.53;-0.91) | 1.99E-04 | -2.13 (-2.80;-1.47) | 2.37E-09 | -2.14 (-2.81;-1.48) | 2.22E-09 |
| 3-Methoxycatechol Sulfate (1) | -1.65 (-2.47;-0.83) | 4.05E-04 | -2.12 (-2.78;-1.47) | 2.03E-09 | -2.12 (-2.78;-1.46) | 2.26E-09 |
| Dimethylglycine | -1.96 (-2.79;-1.14) | 2.54E-05 | -2.17 (-2.84;-1.49) | 2.47E-09 | -2.17 (-2.84;-1.50) | 2.40E-09 |
| Urea | -1.51 (-2.33;-0.68) | 1.62E-03 | -2.15 (-2.82;-1.48) | 2.61E-09 | -2.16 (-2.83;-1.49) | 2.49E-09 |
| 2,3-Dihydroxyisovalerate | -1.09 (-1.92;-0.27) | 2.67E-02 | -2.20 (-2.88;-1.51) | 2.37E-09 | -2.20 (-2.88;-1.52) | 2.55E-09 |
| 4-Methylhexanoylglutamine | -2.10 (-2.93;-1.27) | 7.30E-06 | -2.16 (-2.83;-1.48) | 3.41E-09 | -2.15 (-2.83;-1.48) | 3.86E-09 |
| Tiglylcarnitine (C5:1-DC) | -1.72 (-2.54;-0.90) | 2.32E-04 | -2.06 (-2.72;-1.40) | 9.30E-09 | -2.09 (-2.76;-1.43) | 5.94E-09 |
| Imidazole Propionate | -2.84 (-3.67;-2) | 8.96E-10 | -2.20 (-2.90;-1.50) | 6.14E-09 | -2.21 (-2.91;-1.51) | 6.08E-09 |
| 1-Methyl-5-Imidazolelactate | -2.24 (-3.06;-1.41) | 1.34E-06 | -2.09 (-2.77;-1.42) | 9.61E-09 | -2.11 (-2.78;-1.43) | 8.14E-09 |
| Gamma-Carboxyglutamate | -0.97 (-1.79;-0.15) | 4.92E-02 | -2.03 (-2.69;-1.38) | 9.30E-09 | -2.03 (-2.69;-1.38) | 9.94E-09 |
| Pantoate | -2.03 (-2.84;-1.22) | 9.47E-06 | -2.04 (-2.70;-1.38) | 9.48E-09 | -2.04 (-2.70;-1.38) | 1.06E-08 |
| Creatine | 1.70 (0.80;2.60) | 1.07E-03 | 2.28 (1.54;3.02) | 1.19E-08 | 2.29 (1.55;3.04) | 1.10E-08 |
| X-24422 | -2.41 (-3.24;-1.59) | 1.18E-07 | -2.05 (-2.71;-1.38) | 1.33E-08 | -2.05 (-2.72;-1.39) | 1.30E-08 |
| X-24812 | -2.43 (-3.24;-1.62) | 5.39E-08 | -2.35 (-3.12;-1.58) | 1.90E-08 | -2.38 (-3.15;-1.60) | 1.45E-08 |
| Indolelactate | -1.08 (-1.94;-0.22) | 3.58E-02 | -2.09 (-2.78;-1.41) | 1.77E-08 | -2.10 (-2.78;-1.41) | 1.77E-08 |
| Orotate | -1.37 (-2.19;-0.55) | 4.38E-03 | -2.00 (-2.66;-1.35) | 1.81E-08 | -2.00 (-2.66;-1.35) | 1.93E-08 |
| Pentose Acid* | -1.11 (-1.93;-0.29) | 2.30E-02 | -2.00 (-2.66;-1.34) | 2.00E-08 | -2.01 (-2.67;-1.35) | 2.04E-08 |
| Imidazole Lactate | -1.16 (-1.99;-0.33) | 1.83E-02 | -2.01 (-2.67;-1.34) | 2.25E-08 | -2.01 (-2.67;-1.35) | 2.26E-08 |
| N-Acetyl-Isoputreanine | -1.44 (-2.26;-0.63) | 2.37E-03 | -2.00 (-2.66;-1.34) | 1.94E-08 | -1.99 (-2.65;-1.33) | 2.38E-08 |
| Aconitate [Cis Or Trans] | -1.96 (-2.77;-1.14) | 2.11E-05 | -2.00 (-2.68;-1.33) | 3.85E-08 | -2.05 (-2.73;-1.37) | 2.43E-08 |
| Glutarylcarnitine (C5-DC) | -1.64 (-2.47;-0.82) | 5.03E-04 | -1.99 (-2.65;-1.33) | 3.08E-08 | -2.02 (-2.69;-1.35) | 2.49E-08 |
| 3-Ureidopropionate | -2.02 (-2.89;-1.15) | 4.11E-05 | -2.13 (-2.83;-1.42) | 3.07E-08 | -2.14 (-2.85;-1.43) | 2.54E-08 |
| (S)-3-Hydroxybutyrylcarnitine | -2.76 (-3.58;-1.94) | 1.07E-09 | -2.02 (-2.71;-1.33) | 6.59E-08 | -2.12 (-2.83;-1.42) | 2.65E-08 |
| Urate | -2.04 (-2.92;-1.16) | 4.02E-05 | -2.16 (-2.88;-1.44) | 3.17E-08 | -2.18 (-2.90;-1.46) | 2.66E-08 |
| X-15486 | -2.24 (-3.05;-1.42) | 9.64E-07 | -1.99 (-2.65;-1.33) | 3.17E-08 | -2.01 (-2.68;-1.34) | 2.69E-08 |
| X-17357 | -1.76 (-2.58;-0.93) | 1.87E-04 | -1.97 (-2.63;-1.31) | 3.50E-08 | -1.99 (-2.65;-1.33) | 2.88E-08 |
| Citrulline | -1.12 (-1.93;-0.30) | 2.13E-02 | -1.99 (-2.65;-1.33) | 3.07E-08 | -2.00 (-2.67;-1.33) | 2.94E-08 |
| X-23739 | -1.30 (-2.12;-0.47) | 7.49E-03 | -1.99 (-2.66;-1.33) | 3.02E-08 | -1.99 (-2.66;-1.33) | 3.34E-08 |
| X-16580 | -1.43 (-2.25;-0.61) | 2.83E-03 | -1.93 (-2.59;-1.27) | 8.52E-08 | -2.00 (-2.67;-1.33) | 3.97E-08 |
| Androsterone Glucuronide | -2.55 (-3.46;-1.64) | 4.27E-07 | -2.14 (-2.87;-1.42) | 4.07E-08 | -2.15 (-2.88;-1.43) | 4.10E-08 |
| X-19141 | -0.99 (-1.82;-0.17) | 4.57E-02 | -1.95 (-2.61;-1.29) | 4.88E-08 | -1.97 (-2.63;-1.30) | 4.17E-08 |
| Ethyl Alpha-Glucopyranoside | -1.28 (-2.12;-0.44) | 9.69E-03 | -2.25 (-3.01;-1.49) | 4.59E-08 | -2.25 (-3.02;-1.49) | 4.88E-08 |
| X-24328 | -2.31 (-3.28;-1.34) | 2.41E-05 | -2.36 (-3.16;-1.56) | 5.51E-08 | -2.37 (-3.17;-1.57) | 5.07E-08 |
| 3-Methylglutaconate | -1.52 (-2.34;-0.69) | 1.47E-03 | -2.08 (-2.79;-1.37) | 6.69E-08 | -2.08 (-2.79;-1.37) | 7.11E-08 |
| Dihydroorotate | -1.69 (-2.52;-0.87) | 3.06E-04 | -1.94 (-2.60;-1.27) | 8.12E-08 | -1.95 (-2.62;-1.29) | 7.15E-08 |
| 3-Hydroxyoctanoylcarnitine (1) | -1.44 (-2.28;-0.60) | 3.40E-03 | -1.95 (-2.63;-1.27) | 1.26E-07 | -1.97 (-2.65;-1.29) | 1.07E-07 |
| Cytosine | -1.86 (-2.67;-1.05) | 5.17E-05 | -1.89 (-2.55;-1.24) | 9.81E-08 | -1.89 (-2.55;-1.24) | 1.09E-07 |
| Gamma-Glutamylcitrulline* | -1.07 (-1.89;-0.26) | 2.77E-02 | -1.91 (-2.57;-1.24) | 1.22E-07 | -1.93 (-2.60;-1.26) | 1.12E-07 |
| X-16397 | -1.49 (-2.30;-0.67) | 1.63E-03 | -1.87 (-2.52;-1.21) | 1.55E-07 | -1.90 (-2.56;-1.24) | 1.13E-07 |
| 3-Methyl Catechol Sulfate (2) | -2.67 (-3.49;-1.85) | 3.51E-09 | -1.97 (-2.65;-1.28) | 1.22E-07 | -1.96 (-2.65;-1.28) | 1.46E-07 |
| 4-Acetamidobutanoate | -1.91 (-2.73;-1.09) | 3.95E-05 | -1.85 (-2.50;-1.19) | 2.28E-07 | -1.85 (-2.51;-1.20) | 2.14E-07 |
| S-Carboxyethylcysteine | -2.54 (-3.35;-1.72) | 1.76E-08 | -1.99 (-2.70;-1.28) | 2.39E-07 | -2.00 (-2.71;-1.29) | 2.36E-07 |
| 8-Methoxykynurenate | -1.76 (-2.61;-0.91) | 2.94E-04 | -1.94 (-2.64;-1.24) | 4.06E-07 | -1.96 (-2.67;-1.26) | 3.33E-07 |
| Hexanoylglutamine | -1.93 (-2.76;-1.10) | 4.05E-05 | -1.86 (-2.54;-1.19) | 4.42E-07 | -1.87 (-2.55;-1.19) | 4.31E-07 |
| X-24344 | -2.55 (-3.37;-1.74) | 1.46E-08 | -2.03 (-2.76;-1.29) | 4.26E-07 | -2.03 (-2.77;-1.29) | 4.68E-07 |
| 4-Vinylguaiacol Sulfate | -2.52 (-3.35;-1.69) | 4.11E-08 | -1.98 (-2.71;-1.26) | 5.07E-07 | -2.00 (-2.73;-1.27) | 4.75E-07 |
| Serine | 1.74 (0.92;2.55) | 1.90E-04 | 1.80 (1.14;2.47) | 6.00E-07 | 1.82 (1.16;2.49) | 5.02E-07 |
| 3-Methylxanthine | -1.68 (-2.49;-0.86) | 3.11E-04 | -1.79 (-2.45;-1.14) | 5.07E-07 | -1.79 (-2.45;-1.14) | 5.28E-07 |
| Phenyllactate (PLA) | -1.47 (-2.35;-0.59) | 4.36E-03 | -1.93 (-2.64;-1.22) | 6.55E-07 | -1.95 (-2.66;-1.23) | 5.48E-07 |
| X-11444 | -1.78 (-2.62;-0.94) | 2.17E-04 | -1.82 (-2.49;-1.15) | 7.12E-07 | -1.83 (-2.51;-1.15) | 6.90E-07 |
| X-26106 | -1.65 (-2.48;-0.81) | 5.66E-04 | -1.80 (-2.48;-1.13) | 9.66E-07 | -1.84 (-2.51;-1.16) | 7.00E-07 |
| Undecenoylcarnitine (C11:1) | -1.55 (-2.38;-0.72) | 1.18E-03 | -1.80 (-2.46;-1.13) | 7.26E-07 | -1.80 (-2.47;-1.14) | 7.08E-07 |
| N1-Methyladenosine | -1.15 (-1.97;-0.33) | 1.84E-02 | -1.77 (-2.42;-1.12) | 6.95E-07 | -1.77 (-2.43;-1.12) | 7.14E-07 |
| X-24588 | -1.30 (-2.14;-0.46) | 8.50E-03 | -1.84 (-2.52;-1.16) | 7.34E-07 | -1.84 (-2.53;-1.16) | 7.46E-07 |
| N-Acetylphenylalanine | -2.19 (-3.04;-1.34) | 4.81E-06 | -1.92 (-2.63;-1.20) | 8.41E-07 | -1.92 (-2.64;-1.21) | 7.93E-07 |
| Gamma-Glutamylleucine | -1.73 (-2.61;-0.85) | 5.90E-04 | -1.91 (-2.63;-1.19) | 1.00E-06 | -1.93 (-2.65;-1.21) | 8.31E-07 |
| Glucuronate | -1.69 (-2.51;-0.87) | 2.97E-04 | -1.76 (-2.42;-1.10) | 1.07E-06 | -1.79 (-2.46;-1.12) | 8.57E-07 |
| X-26119 | -1.73 (-2.56;-0.91) | 2.32E-04 | -1.78 (-2.45;-1.12) | 9.54E-07 | -1.79 (-2.46;-1.12) | 8.63E-07 |
| X-23680 | -1.27 (-2.10;-0.45) | 8.61E-03 | -1.76 (-2.42;-1.10) | 1.00E-06 | -1.77 (-2.44;-1.11) | 8.76E-07 |
| 2-Aminophenol Sulfate | -1.36 (-2.18;-0.54) | 4.52E-03 | -1.78 (-2.44;-1.11) | 9.56E-07 | -1.77 (-2.44;-1.11) | 1.02E-06 |
| N-Acetylisoleucine | -1.42 (-2.25;-0.58) | 3.63E-03 | -1.81 (-2.50;-1.12) | 1.40E-06 | -1.84 (-2.53;-1.15) | 1.06E-06 |
| 3-Methoxytyrosine | -1.13 (-1.95;-0.31) | 2.01E-02 | -1.75 (-2.41;-1.09) | 1.33E-06 | -1.77 (-2.44;-1.10) | 1.11E-06 |
| X-13728 | -1.90 (-2.71;-1.08) | 3.73E-05 | -1.74 (-2.39;-1.09) | 1.02E-06 | -1.74 (-2.39;-1.09) | 1.11E-06 |
| 3-Ethylcatechol Sulfate (1) | -2.44 (-3.25;-1.63) | 5.28E-08 | -1.85 (-2.55;-1.16) | 1.07E-06 | -1.85 (-2.55;-1.15) | 1.16E-06 |
| Lanthionine | -1.68 (-2.50;-0.87) | 2.97E-04 | -1.75 (-2.41;-1.09) | 1.15E-06 | -1.75 (-2.41;-1.09) | 1.24E-06 |
| 3-Acetylphenol Sulfate | -1.92 (-2.74;-1.10) | 3.44E-05 | -1.80 (-2.48;-1.12) | 1.11E-06 | -1.79 (-2.47;-1.11) | 1.29E-06 |
| Phenylacetylglutamate | -1.72 (-2.55;-0.90) | 2.59E-04 | -1.77 (-2.45;-1.09) | 1.74E-06 | -1.80 (-2.48;-1.12) | 1.29E-06 |
| Hypotaurine | -0.83 (-1.65;0.00) | 1.03E-01 | -1.79 (-2.46;-1.11) | 1.36E-06 | -1.79 (-2.48;-1.11) | 1.37E-06 |
| 3-Hydroxydecanoylcarnitine | -1.22 (-2.06;-0.37) | 1.51E-02 | -1.79 (-2.47;-1.11) | 1.47E-06 | -1.79 (-2.48;-1.11) | 1.41E-06 |
| 2s,3r-Dihydroxybutyrate | -1.20 (-2.12;-0.27) | 3.01E-02 | -1.96 (-2.71;-1.20) | 2.45E-06 | -2.01 (-2.77;-1.24) | 1.49E-06 |
| (N(1) + N(8))-Acetylspermidine | -1.34 (-2.17;-0.51) | 5.88E-03 | -1.75 (-2.42;-1.08) | 1.79E-06 | -1.77 (-2.44;-1.09) | 1.66E-06 |
| Cortolone Glucuronide (1) | -1.85 (-2.67;-1.03) | 7.50E-05 | -1.82 (-2.52;-1.12) | 1.99E-06 | -1.84 (-2.54;-1.13) | 1.67E-06 |
| X-12101 | -1.58 (-2.40;-0.76) | 7.74E-04 | -1.71 (-2.38;-1.04) | 2.78E-06 | -1.76 (-2.44;-1.08) | 1.78E-06 |
| X-13431 | -0.99 (-1.82;-0.16) | 4.76E-02 | -1.70 (-2.36;-1.04) | 2.51E-06 | -1.72 (-2.38;-1.05) | 2.31E-06 |
| Ascorbic Acid 3-Sulfate* | -1.46 (-2.30;-0.63) | 2.63E-03 | -1.74 (-2.42;-1.07) | 2.29E-06 | -1.75 (-2.42;-1.07) | 2.32E-06 |
| Formiminoglutamate | -2.16 (-3.02;-1.30) | 8.73E-06 | -1.86 (-2.59;-1.13) | 3.20E-06 | -1.90 (-2.63;-1.16) | 2.35E-06 |
| Cytidine | -1.70 (-2.51;-0.88) | 2.54E-04 | -1.69 (-2.35;-1.03) | 2.47E-06 | -1.70 (-2.36;-1.04) | 2.35E-06 |
| Heptenedioate (C7:1-DC)* | -1.50 (-2.33;-0.68) | 1.73E-03 | -1.70 (-2.37;-1.03) | 3.31E-06 | -1.73 (-2.40;-1.05) | 2.71E-06 |
| 11beta-Hydroxyetiocholanolone Glucuronide* | -1.12 (-1.94;-0.29) | 2.27E-02 | -1.66 (-2.32;-0.99) | 5.12E-06 | -1.68 (-2.35;-1.01) | 4.21E-06 |
| X-24456 | -1.06 (-1.88;-0.24) | 3.08E-02 | -1.68 (-2.34;-1.01) | 3.69E-06 | -1.67 (-2.34;-1.01) | 4.26E-06 |
| N-Acetylcitrulline | -1.56 (-2.39;-0.74) | 1.01E-03 | -1.69 (-2.36;-1.01) | 4.98E-06 | -1.70 (-2.38;-1.03) | 4.30E-06 |
| 2r,3r-Dihydroxybutyrate | -0.91 (-1.73;-0.09) | 6.91E-02 | -1.66 (-2.32;-1.00) | 4.18E-06 | -1.66 (-2.32;-1.00) | 4.56E-06 |
| 2-Aminobutyrate | 1.46 (0.65;2.28) | 1.96E-03 | 1.68 (1.01;2.34) | 4.24E-06 | 1.68 (1.01;2.35) | 4.90E-06 |
| 2,6-Dihydroxybenzoic Acid | -1.07 (-1.89;-0.26) | 2.77E-02 | -1.65 (-2.31;-0.99) | 5.37E-06 | -1.65 (-2.31;-0.99) | 5.42E-06 |
| X-25810 | -1.10 (-1.93;-0.28) | 2.43E-02 | -1.64 (-2.31;-0.97) | 7.12E-06 | -1.65 (-2.32;-0.98) | 6.47E-06 |
| 3-Hydroxypyridine Sulfate | -1.83 (-2.65;-1.00) | 9.71E-05 | -1.68 (-2.37;-1.00) | 6.35E-06 | -1.69 (-2.37;-1.00) | 6.73E-06 |
| 3-Hydroxyoctanoylcarnitine (2) | -1.23 (-2.08;-0.38) | 1.41E-02 | -1.67 (-2.35;-0.99) | 7.91E-06 | -1.69 (-2.37;-1.00) | 6.73E-06 |
| X-12407 | -1.29 (-2.11;-0.48) | 7.04E-03 | -1.60 (-2.26;-0.95) | 8.97E-06 | -1.62 (-2.28;-0.96) | 7.73E-06 |
| Ascorbic Acid 2-Sulfate | -1.58 (-2.40;-0.77) | 7.07E-04 | -1.60 (-2.25;-0.94) | 9.59E-06 | -1.61 (-2.27;-0.95) | 8.60E-06 |
| 3-Hydroxy-2-Ethylpropionate | -1.18 (-2.00;-0.35) | 1.69E-02 | -1.60 (-2.26;-0.93) | 1.42E-05 | -1.64 (-2.32;-0.97) | 8.91E-06 |
| Citraconate/Glutaconate | -1.67 (-2.50;-0.84) | 4.10E-04 | -1.65 (-2.32;-0.97) | 8.75E-06 | -1.64 (-2.32;-0.97) | 9.43E-06 |
| N-Delta-Acetylornithine | -0.89 (-1.70;-0.07) | 7.50E-02 | -1.60 (-2.26;-0.94) | 9.27E-06 | -1.60 (-2.26;-0.94) | 9.86E-06 |
| N-(2-Furoyl)Glycine | -1.87 (-2.71;-1.04) | 7.55E-05 | -1.64 (-2.33;-0.95) | 1.47E-05 | -1.66 (-2.35;-0.97) | 1.25E-05 |
| X-12221 | -1.84 (-2.66;-1.02) | 8.33E-05 | -1.61 (-2.28;-0.94) | 1.17E-05 | -1.61 (-2.28;-0.94) | 1.28E-05 |
| Octadecenedioylcarnitine (C18:1-DC)* | -1.78 (-2.62;-0.93) | 2.17E-04 | -1.62 (-2.30;-0.94) | 1.36E-05 | -1.63 (-2.31;-0.95) | 1.32E-05 |
| Pyridoxate | -0.78 (-1.60;0.04) | 1.22E-01 | -1.58 (-2.25;-0.92) | 1.47E-05 | -1.60 (-2.26;-0.93) | 1.37E-05 |
| X-24462 | -0.92 (-1.75;-0.10) | 6.43E-02 | -1.57 (-2.23;-0.91) | 1.47E-05 | -1.58 (-2.24;-0.92) | 1.41E-05 |
| Decadienedioic Acid (C10:2-Dc)** | -1.71 (-2.52;-0.89) | 2.42E-04 | -1.57 (-2.23;-0.91) | 1.64E-05 | -1.57 (-2.24;-0.91) | 1.60E-05 |
| Hydroquinone Sulfate | -1.62 (-2.44;-0.81) | 4.84E-04 | -1.57 (-2.24;-0.91) | 1.63E-05 | -1.57 (-2.24;-0.90) | 1.84E-05 |
| 4-Guanidinobutanoate | -1.15 (-2.04;-0.27) | 2.98E-02 | -1.68 (-2.39;-0.97) | 1.77E-05 | -1.68 (-2.40;-0.97) | 1.85E-05 |
| Deoxycarnitine | -1.17 (-2.02;-0.32) | 2.11E-02 | -1.60 (-2.29;-0.92) | 2.01E-05 | -1.62 (-2.31;-0.93) | 1.96E-05 |
| X-23639 | -0.94 (-1.75;-0.12) | 5.82E-02 | -1.59 (-2.26;-0.92) | 1.64E-05 | -1.59 (-2.26;-0.91) | 1.98E-05 |
| 3-Hydroxysebacate | -1.78 (-2.63;-0.93) | 2.41E-04 | -1.60 (-2.30;-0.90) | 3.16E-05 | -1.66 (-2.37;-0.95) | 2.08E-05 |
| Nonanoylcarnitine (C9) | -1.18 (-2.01;-0.35) | 1.74E-02 | -1.57 (-2.24;-0.89) | 2.37E-05 | -1.57 (-2.25;-0.90) | 2.23E-05 |
| Cortisone | 1.93 (1.12;2.75) | 2.50E-05 | 1.56 (0.89;2.22) | 2.01E-05 | 1.56 (0.89;2.22) | 2.23E-05 |
| Carboxyethyl-GABA | -1.08 (-1.89;-0.26) | 2.73E-02 | -1.53 (-2.19;-0.87) | 2.37E-05 | -1.53 (-2.19;-0.87) | 2.36E-05 |
| 4-Hydroxyhippurate | -1.04 (-1.86;-0.22) | 3.35E-02 | -1.55 (-2.21;-0.88) | 2.42E-05 | -1.55 (-2.22;-0.89) | 2.38E-05 |
| 6-Hydroxyindole Sulfate | -1.40 (-2.22;-0.57) | 3.85E-03 | -1.55 (-2.23;-0.88) | 2.79E-05 | -1.56 (-2.24;-0.89) | 2.69E-05 |
| 5-Dodecenoylcarnitine (C12:1) | -1.48 (-2.32;-0.64) | 2.43E-03 | -1.58 (-2.26;-0.90) | 2.70E-05 | -1.58 (-2.27;-0.90) | 2.69E-05 |
| Gamma-Glutamylvaline | -1.55 (-2.38;-0.72) | 1.25E-03 | -1.55 (-2.22;-0.87) | 3.30E-05 | -1.56 (-2.24;-0.88) | 3.03E-05 |
| Octadecanedioylcarnitine (C18-DC)* | -1.35 (-2.19;-0.51) | 6.08E-03 | -1.55 (-2.24;-0.87) | 3.77E-05 | -1.58 (-2.26;-0.89) | 3.11E-05 |
| Gamma-Glutamylglycine | -0.90 (-1.72;-0.08) | 7.37E-02 | -1.57 (-2.25;-0.88) | 3.47E-05 | -1.57 (-2.27;-0.88) | 3.70E-05 |
| 3-Hydroxy-2-Methylpyridine Sulfate | -1.73 (-2.54;-0.91) | 2.06E-04 | -1.54 (-2.23;-0.86) | 4.14E-05 | -1.56 (-2.24;-0.87) | 3.81E-05 |
| N6,N6,N6-Trimethyllysine | -0.83 (-1.66;0.00) | 1.04E-01 | -1.51 (-2.18;-0.84) | 4.35E-05 | -1.55 (-2.23;-0.86) | 3.81E-05 |
| 3-Carboxy-4-Methyl-5-Pentyl-2-Furanpropionate (3-Cmpfp)** | -0.61 (-1.43;0.22) | 2.50E-01 | -1.52 (-2.19;-0.85) | 3.96E-05 | -1.53 (-2.20;-0.85) | 3.96E-05 |
| X-12410 | -1.14 (-1.96;-0.31) | 2.01E-02 | -1.49 (-2.16;-0.83) | 4.49E-05 | -1.51 (-2.17;-0.84) | 4.10E-05 |
| 3-Hydroxybutyroylglycine** | -1.71 (-2.52;-0.89) | 2.47E-04 | -1.50 (-2.17;-0.84) | 4.34E-05 | -1.51 (-2.18;-0.84) | 4.12E-05 |
| X-25420 | -1.76 (-2.61;-0.91) | 2.91E-04 | -1.61 (-2.32;-0.90) | 3.77E-05 | -1.61 (-2.33;-0.90) | 4.12E-05 |
| Ergothioneine | 2.37 (1.56;3.18) | 1.24E-07 | 1.54 (0.86;2.21) | 3.81E-05 | 1.54 (0.86;2.21) | 4.25E-05 |
| Cis-3,4-Methyleneheptanoylglycine | -1.56 (-2.39;-0.74) | 1.01E-03 | -1.49 (-2.16;-0.83) | 5.12E-05 | -1.51 (-2.18;-0.84) | 4.56E-05 |
| Myristoleoylcarnitine (C14:1)* | -1.29 (-2.12;-0.47) | 7.96E-03 | -1.49 (-2.15;-0.82) | 5.59E-05 | -1.49 (-2.16;-0.82) | 5.58E-05 |
| Catechol Sulfate | -1.68 (-2.49;-0.86) | 2.97E-04 | -1.50 (-2.16;-0.83) | 4.78E-05 | -1.49 (-2.16;-0.82) | 5.60E-05 |
| 1-(1-Enyl-Palmitoyl)-2-Arachidonoyl-Gpe (P-16:0/20:4)* | 1.59 (0.77;2.40) | 6.88E-04 | 1.48 (0.82;2.15) | 5.31E-05 | 1.48 (0.81;2.15) | 5.89E-05 |
| 1-Methylurate | -1.43 (-2.27;-0.59) | 3.40E-03 | -1.49 (-2.16;-0.82) | 5.66E-05 | -1.49 (-2.16;-0.82) | 5.90E-05 |
| 2-Hydroxysebacate | -1.73 (-2.56;-0.90) | 2.52E-04 | -1.49 (-2.16;-0.82) | 6.11E-05 | -1.50 (-2.18;-0.82) | 5.99E-05 |
| 3-(4-Hydroxyphenyl)Lactate | -1.30 (-2.18;-0.42) | 1.28E-02 | -1.57 (-2.28;-0.85) | 7.25E-05 | -1.59 (-2.31;-0.87) | 6.05E-05 |
| 7-Methylxanthine | -1.41 (-2.22;-0.59) | 3.15E-03 | -1.46 (-2.11;-0.80) | 6.25E-05 | -1.46 (-2.12;-0.80) | 6.67E-05 |
| 5,6-Dihydrothymine | -1.06 (-1.89;-0.24) | 3.07E-02 | -1.45 (-2.11;-0.79) | 7.25E-05 | -1.47 (-2.13;-0.80) | 6.79E-05 |
| 2-Naphthol Sulfate | -2.46 (-3.28;-1.64) | 5.28E-08 | -1.86 (-2.70;-1.01) | 7.25E-05 | -1.86 (-2.71;-1.01) | 7.53E-05 |
| Choline | -0.48 (-1.32;0.35) | 3.72E-01 | -1.47 (-2.15;-0.80) | 7.76E-05 | -1.47 (-2.15;-0.80) | 8.16E-05 |
| Tetrahydrocortisol Glucuronide | -1.38 (-2.25;-0.52) | 6.68E-03 | -1.49 (-2.19;-0.79) | 1.25E-04 | -1.52 (-2.23;-0.82) | 9.33E-05 |
| Allantoin | -1.17 (-1.99;-0.35) | 1.69E-02 | -1.43 (-2.09;-0.77) | 9.79E-05 | -1.44 (-2.10;-0.77) | 9.41E-05 |
| Glycine Conjugate Of C10h14o2 (1)* | -1.56 (-2.37;-0.74) | 8.88E-04 | -1.41 (-2.07;-0.75) | 1.18E-04 | -1.44 (-2.10;-0.77) | 9.59E-05 |
| N6-Methyladenosine | -1.34 (-2.16;-0.52) | 5.19E-03 | -1.42 (-2.07;-0.76) | 9.15E-05 | -1.42 (-2.07;-0.76) | 9.88E-05 |
| Laurylcarnitine (C12) | -1.06 (-1.90;-0.22) | 3.60E-02 | -1.46 (-2.13;-0.78) | 1.03E-04 | -1.46 (-2.14;-0.78) | 9.91E-05 |
| Mannitol/Sorbitol | -0.87 (-1.69;-0.05) | 8.13E-02 | -1.41 (-2.06;-0.75) | 1.16E-04 | -1.42 (-2.09;-0.76) | 1.00E-04 |
| 4-Vinylphenol Sulfate | -1.83 (-2.68;-0.99) | 1.34E-04 | -1.51 (-2.21;-0.80) | 1.09E-04 | -1.50 (-2.20;-0.80) | 1.19E-04 |
| 2-Hydroxyphenylacetate | -0.99 (-1.82;-0.16) | 4.92E-02 | -1.41 (-2.09;-0.74) | 1.52E-04 | -1.44 (-2.12;-0.76) | 1.20E-04 |
| Dodecanedioate (C12-DC) | -1.23 (-2.06;-0.41) | 1.16E-02 | -1.45 (-2.13;-0.77) | 1.28E-04 | -1.45 (-2.13;-0.76) | 1.37E-04 |
| P-Cresol Sulfate | -0.94 (-1.77;-0.12) | 5.87E-02 | -1.40 (-2.08;-0.73) | 1.80E-04 | -1.42 (-2.10;-0.74) | 1.58E-04 |
| 1-(1-Enyl-Stearoyl)-2-Arachidonoyl-Gpe (P-18:0/20:4)* | 1.47 (0.65;2.28) | 1.96E-03 | 1.41 (0.74;2.08) | 1.53E-04 | 1.41 (0.73;2.08) | 1.65E-04 |
| Alpha-Ketobutyrate | 0.99 (0.17;1.81) | 4.42E-02 | 1.45 (0.76;2.14) | 1.57E-04 | 1.45 (0.76;2.15) | 1.69E-04 |
| Choline Phosphate | 2.24 (1.43;3.06) | 8.50E-07 | 1.44 (0.75;2.13) | 1.60E-04 | 1.44 (0.75;2.13) | 1.69E-04 |
| X-21829 | -2.05 (-2.88;-1.23) | 1.05E-05 | -1.74 (-2.57;-0.90) | 1.76E-04 | -1.74 (-2.57;-0.91) | 1.77E-04 |
| Hippurate | -1.22 (-2.05;-0.40) | 1.22E-02 | -1.40 (-2.07;-0.73) | 1.78E-04 | -1.41 (-2.08;-0.73) | 1.78E-04 |
| Oxalate (Ethanedioate) | 2.02 (1.18;2.85) | 1.83E-05 | 1.41 (0.73;2.10) | 2.11E-04 | 1.43 (0.75;2.12) | 1.79E-04 |
| Lyxonate | -0.32 (-1.14;0.50) | 5.62E-01 | -1.37 (-2.02;-0.71) | 1.78E-04 | -1.37 (-2.03;-0.71) | 1.79E-04 |
| X-11632 | -1.35 (-2.18;-0.53) | 5.03E-03 | -1.39 (-2.06;-0.73) | 1.64E-04 | -1.39 (-2.06;-0.72) | 1.79E-04 |
| 1-Carboxyethylisoleucine | -2.22 (-3.06;-1.38) | 2.39E-06 | -1.74 (-2.61;-0.87) | 3.12E-04 | -1.83 (-2.72;-0.94) | 2.19E-04 |
| Gluconate | -0.73 (-1.56;0.09) | 1.53E-01 | -1.34 (-2.01;-0.68) | 2.85E-04 | -1.37 (-2.04;-0.70) | 2.28E-04 |
| X-12216 | -0.58 (-1.41;0.25) | 2.76E-01 | -1.39 (-2.06;-0.71) | 2.20E-04 | -1.39 (-2.06;-0.71) | 2.28E-04 |
| Tartronate (Hydroxymalonate) | 1.89 (1.06;2.72) | 5.48E-05 | 1.39 (0.70;2.08) | 2.79E-04 | 1.42 (0.73;2.11) | 2.28E-04 |
| Methyl Indole-3-Acetate | -1.25 (-2.08;-0.43) | 1.00E-02 | -1.35 (-2.02;-0.68) | 2.96E-04 | -1.38 (-2.05;-0.71) | 2.30E-04 |
| 4-Ethylcatechol Sulfate | -1.46 (-2.28;-0.64) | 2.21E-03 | -1.39 (-2.07;-0.71) | 2.28E-04 | -1.39 (-2.07;-0.71) | 2.31E-04 |
| Tetrahydrocortisone Glucuronide (5) | -1.53 (-2.38;-0.68) | 1.96E-03 | -1.39 (-2.09;-0.70) | 3.27E-04 | -1.44 (-2.14;-0.73) | 2.33E-04 |
| N-Acetylglutamine | -1.05 (-1.87;-0.22) | 3.45E-02 | -1.36 (-2.02;-0.69) | 2.44E-04 | -1.37 (-2.03;-0.70) | 2.34E-04 |
| Myristoylcarnitine (C14) | -1.30 (-2.14;-0.46) | 8.50E-03 | -1.38 (-2.06;-0.70) | 2.73E-04 | -1.39 (-2.07;-0.71) | 2.48E-04 |
| Cysteinylglycine Disulfide* | -0.33 (-1.15;0.49) | 5.51E-01 | -1.33 (-1.98;-0.67) | 2.90E-04 | -1.34 (-2.00;-0.68) | 2.52E-04 |
| N6,N6-Dimethyllysine | -1.15 (-1.97;-0.33) | 1.84E-02 | -1.35 (-2.00;-0.69) | 2.33E-04 | -1.34 (-2.00;-0.68) | 2.57E-04 |
| N-Acetyl-2-Aminooctanoate* | -0.65 (-1.49;0.19) | 2.28E-01 | -1.38 (-2.07;-0.70) | 2.88E-04 | -1.40 (-2.08;-0.71) | 2.62E-04 |
| X-12193 | -1.17 (-1.98;-0.35) | 1.65E-02 | -1.32 (-1.99;-0.66) | 3.16E-04 | -1.34 (-2.00;-0.68) | 2.81E-04 |
| 4-Hydroxyphenylacetate | -1.81 (-2.63;-0.98) | 1.12E-04 | -1.34 (-2.01;-0.67) | 3.23E-04 | -1.35 (-2.02;-0.68) | 2.94E-04 |
| Glycine Conjugate Of C10h12o2* | -1.60 (-2.41;-0.78) | 6.37E-04 | -1.30 (-1.96;-0.64) | 4.12E-04 | -1.33 (-1.99;-0.66) | 3.30E-04 |
| N-Methylpipecolate | -1.07 (-1.88;-0.25) | 2.90E-02 | -1.32 (-1.97;-0.66) | 3.23E-04 | -1.32 (-1.97;-0.66) | 3.33E-04 |
| Phosphoethanolamine | 1.79 (0.98;2.60) | 1.06E-04 | 1.34 (0.67;2.01) | 3.34E-04 | 1.34 (0.67;2.02) | 3.33E-04 |
| 2-Pyrrolidinone | -0.77 (-1.59;0.05) | 1.28E-01 | -1.31 (-1.96;-0.65) | 3.49E-04 | -1.31 (-1.97;-0.65) | 3.45E-04 |
| N-Acetylkynurenine (2) | -1.18 (-2.05;-0.32) | 2.15E-02 | -1.41 (-2.13;-0.69) | 4.34E-04 | -1.43 (-2.15;-0.71) | 3.71E-04 |
| X-17367 | -1.33 (-2.15;-0.51) | 5.77E-03 | -1.30 (-1.97;-0.64) | 4.25E-04 | -1.30 (-1.97;-0.64) | 4.59E-04 |
| Homoarginine | 1.31 (0.48;2.14) | 7.19E-03 | 1.33 (0.65;2.01) | 4.93E-04 | 1.33 (0.65;2.02) | 4.94E-04 |
| 5-Hydroxyindole Sulfate | -0.86 (-1.68;-0.04) | 8.61E-02 | -1.31 (-1.97;-0.64) | 4.76E-04 | -1.31 (-1.98;-0.64) | 4.96E-04 |
| Glycerate | 1.67 (0.85;2.50) | 3.47E-04 | 1.30 (0.62;1.98) | 5.83E-04 | 1.32 (0.64;2.00) | 5.03E-04 |
| Beta-Cryptoxanthin | 2.10 (1.27;2.93) | 7.01E-06 | 1.33 (0.64;2.03) | 6.24E-04 | 1.36 (0.66;2.06) | 5.08E-04 |
| 2-Hydroxy-4-(Methylthio)Butanoic Acid | -1.34 (-2.18;-0.50) | 6.87E-03 | -1.30 (-1.98;-0.62) | 6.51E-04 | -1.32 (-2.00;-0.63) | 5.55E-04 |
| X-11470 | -1.29 (-2.12;-0.46) | 8.50E-03 | -1.28 (-1.94;-0.61) | 5.83E-04 | -1.28 (-1.94;-0.62) | 5.67E-04 |
| N-Acetyltyrosine | -1.17 (-2.00;-0.34) | 1.74E-02 | -1.30 (-1.99;-0.62) | 6.53E-04 | -1.32 (-2.01;-0.63) | 5.71E-04 |
| 4-Methylguaiacol Sulfate | -1.19 (-2.01;-0.37) | 1.48E-02 | -1.26 (-1.92;-0.60) | 6.55E-04 | -1.27 (-1.93;-0.61) | 5.98E-04 |
| 3-Indoxyl Sulfate | -1.15 (-1.97;-0.32) | 1.95E-02 | -1.29 (-1.96;-0.62) | 6.16E-04 | -1.29 (-1.97;-0.62) | 6.04E-04 |
| Gamma-CEHC | -0.52 (-1.34;0.30) | 3.27E-01 | -1.28 (-1.95;-0.61) | 5.87E-04 | -1.28 (-1.94;-0.61) | 6.17E-04 |
| Cis-4-Decenoylcarnitine (C10:1) | -0.90 (-1.72;-0.07) | 7.45E-02 | -1.26 (-1.93;-0.60) | 6.49E-04 | -1.27 (-1.93;-0.61) | 6.22E-04 |
| Dihomo-Linolenoylcarnitine (C20:3n3 Or 6)* | -1.33 (-2.19;-0.48) | 7.84E-03 | -1.31 (-1.99;-0.63) | 5.90E-04 | -1.31 (-1.99;-0.62) | 6.25E-04 |
| Phenylacetylglutamine | -1.10 (-1.93;-0.27) | 2.59E-02 | -1.27 (-1.95;-0.60) | 7.88E-04 | -1.29 (-1.97;-0.61) | 6.80E-04 |
| X-23636 | 1.13 (0.31;1.95) | 2.01E-02 | 1.25 (0.59;1.91) | 7.49E-04 | 1.26 (0.60;1.93) | 6.99E-04 |
| 3,7-Dimethylurate | -1.29 (-2.11;-0.48) | 7.19E-03 | -1.25 (-1.90;-0.59) | 6.59E-04 | -1.25 (-1.90;-0.59) | 7.05E-04 |
| 2-Aminoheptanoate | -0.84 (-1.67;-0.01) | 9.72E-02 | -1.27 (-1.94;-0.60) | 7.40E-04 | -1.27 (-1.95;-0.60) | 7.36E-04 |
| X-24473 | -0.76 (-1.58;0.06) | 1.32E-01 | -1.27 (-1.94;-0.60) | 6.89E-04 | -1.27 (-1.94;-0.60) | 7.40E-04 |
| N-Acetylglutamate | -1.21 (-2.02;-0.39) | 1.26E-02 | -1.24 (-1.90;-0.58) | 8.11E-04 | -1.25 (-1.91;-0.58) | 7.78E-04 |
| Serotonin | 1.70 (0.89;2.51) | 2.41E-04 | 1.49 (0.70;2.28) | 8.03E-04 | 1.49 (0.69;2.28) | 8.21E-04 |
| 1-Palmitoyl-2-Docosahexaenoyl-Gpc (16:0/22:6) | 1.67 (0.86;2.48) | 3.21E-04 | 1.28 (0.59;1.97) | 8.62E-04 | 1.28 (0.60;1.97) | 8.60E-04 |
| 5-Acetylamino-6-Amino-3-Methyluracil | -1.48 (-2.30;-0.66) | 1.95E-03 | -1.24 (-1.90;-0.58) | 8.41E-04 | -1.24 (-1.90;-0.57) | 8.81E-04 |
| Tetradecadienedioate (C14:2-DC)* | -1.18 (-2.01;-0.36) | 1.52E-02 | -1.25 (-1.92;-0.58) | 8.71E-04 | -1.25 (-1.92;-0.58) | 8.84E-04 |
| Trigonelline (N'-Methylnicotinate) | -1.43 (-2.25;-0.62) | 2.57E-03 | -1.25 (-1.92;-0.58) | 8.53E-04 | -1.25 (-1.92;-0.58) | 8.85E-04 |
| Beta-Hydroxyisovaleroylcarnitine | -0.97 (-1.79;-0.14) | 5.17E-02 | -1.22 (-1.88;-0.56) | 1.01E-03 | -1.24 (-1.90;-0.57) | 8.86E-04 |
| 3-Hydroxyoleoylcarnitine | -1.30 (-2.15;-0.46) | 8.78E-03 | -1.26 (-1.93;-0.58) | 9.46E-04 | -1.27 (-1.95;-0.59) | 8.86E-04 |
| X-26107 | -1.65 (-2.51;-0.79) | 8.32E-04 | -1.27 (-1.96;-0.58) | 1.02E-03 | -1.29 (-1.99;-0.60) | 8.86E-04 |
| 1-Carboxyethylphenylalanine | -2.03 (-2.87;-1.20) | 1.61E-05 | -1.57 (-2.44;-0.71) | 1.17E-03 | -1.64 (-2.52;-0.76) | 9.14E-04 |
| Cinnamoylglycine | -1.33 (-2.16;-0.51) | 6.05E-03 | -1.24 (-1.91;-0.57) | 1.01E-03 | -1.25 (-1.92;-0.57) | 9.79E-04 |
| Dihomo-Linoleoylcarnitine (C20:2)* | -1.08 (-1.92;-0.23) | 3.35E-02 | -1.27 (-1.96;-0.59) | 9.34E-04 | -1.27 (-1.96;-0.58) | 9.79E-04 |
| X-24811 | -1.78 (-2.60;-0.96) | 1.33E-04 | -1.27 (-1.95;-0.59) | 8.99E-04 | -1.26 (-1.95;-0.58) | 9.80E-04 |
| Palmitoleoylcarnitine (C16:1)* | -1.21 (-2.04;-0.37) | 1.50E-02 | -1.24 (-1.92;-0.56) | 1.10E-03 | -1.25 (-1.93;-0.57) | 1.08E-03 |
| N6-Acetyllysine | -0.90 (-1.72;-0.09) | 7.00E-02 | -1.22 (-1.88;-0.55) | 1.09E-03 | -1.22 (-1.88;-0.55) | 1.10E-03 |
| X-21383 | -1.36 (-2.18;-0.54) | 4.47E-03 | -1.20 (-1.87;-0.54) | 1.17E-03 | -1.21 (-1.88;-0.55) | 1.10E-03 |
| X-15245 | 0.87 (0.05;1.69) | 8.34E-02 | 1.23 (0.56;1.90) | 1.01E-03 | 1.22 (0.55;1.90) | 1.17E-03 |
| Gamma-Glutamyltryptophan | -1.38 (-2.20;-0.55) | 4.49E-03 | -1.22 (-1.89;-0.55) | 1.19E-03 | -1.23 (-1.90;-0.55) | 1.17E-03 |
| X-11787 | -1.02 (-1.84;-0.19) | 4.08E-02 | -1.21 (-1.87;-0.54) | 1.19E-03 | -1.21 (-1.87;-0.54) | 1.25E-03 |
| 1-Palmitoyl-Gpg (16:0)* | -1.12 (-1.94;-0.30) | 2.18E-02 | -1.22 (-1.89;-0.54) | 1.27E-03 | -1.22 (-1.89;-0.54) | 1.25E-03 |
| X-24475 | -1.23 (-2.08;-0.39) | 1.32E-02 | -1.22 (-1.90;-0.55) | 1.21E-03 | -1.22 (-1.90;-0.54) | 1.33E-03 |
| 2-Hydroxyoctanoate | -0.96 (-1.78;-0.14) | 5.37E-02 | -1.19 (-1.85;-0.52) | 1.43E-03 | -1.20 (-1.86;-0.53) | 1.38E-03 |
| 1-Palmitoyl-2-Linoleoyl-Gpe (16:0/18:2) | -0.75 (-1.58;0.07) | 1.38E-01 | -1.18 (-1.84;-0.52) | 1.48E-03 | -1.20 (-1.86;-0.53) | 1.39E-03 |
| Tridecenedioate (C13:1-DC)* | -0.72 (-1.56;0.12) | 1.67E-01 | -1.23 (-1.91;-0.54) | 1.38E-03 | -1.23 (-1.91;-0.54) | 1.39E-03 |
| Ribitol | -0.76 (-1.58;0.06) | 1.35E-01 | -1.18 (-1.83;-0.52) | 1.45E-03 | -1.18 (-1.84;-0.52) | 1.41E-03 |
| Octanoylcarnitine (C8) | -0.87 (-1.70;-0.03) | 8.83E-02 | -1.19 (-1.86;-0.52) | 1.48E-03 | -1.20 (-1.87;-0.53) | 1.42E-03 |
| 2-Methoxyhydroquinone Sulfate (1) | -1.24 (-2.07;-0.41) | 1.16E-02 | -1.19 (-1.87;-0.52) | 1.58E-03 | -1.20 (-1.88;-0.53) | 1.51E-03 |
| 3-Methylhistidine | -1.10 (-1.91;-0.28) | 2.45E-02 | -1.17 (-1.83;-0.51) | 1.51E-03 | -1.17 (-1.83;-0.51) | 1.54E-03 |
| 4-Acetylphenol Sulfate | -0.41 (-1.23;0.42) | 4.57E-01 | -1.20 (-1.87;-0.53) | 1.45E-03 | -1.20 (-1.87;-0.52) | 1.54E-03 |
| Argininate* | -0.86 (-1.69;-0.03) | 9.11E-02 | -1.20 (-1.88;-0.52) | 1.59E-03 | -1.21 (-1.89;-0.53) | 1.58E-03 |
| X-25828 | 2.00 (1.14;2.86) | 4.31E-05 | 1.52 (0.65;2.40) | 1.97E-03 | 1.59 (0.69;2.48) | 1.58E-03 |
| N-Acetylleucine | -1.32 (-2.16;-0.48) | 7.74E-03 | -1.24 (-1.95;-0.53) | 1.99E-03 | -1.27 (-1.99;-0.55) | 1.65E-03 |
| Sebacate (C10-DC) | -1.29 (-2.13;-0.45) | 9.14E-03 | -1.19 (-1.87;-0.51) | 1.74E-03 | -1.20 (-1.88;-0.52) | 1.72E-03 |
| 4-Vinylcatechol Sulfate | -1.97 (-2.80;-1.14) | 2.73E-05 | -1.25 (-1.95;-0.54) | 1.64E-03 | -1.24 (-1.95;-0.53) | 1.79E-03 |
| Succinate | -0.50 (-1.32;0.32) | 3.45E-01 | -1.16 (-1.82;-0.49) | 1.94E-03 | -1.17 (-1.84;-0.50) | 1.79E-03 |
| Isobutyrylcarnitine (C4) | -0.83 (-1.65;-0.01) | 9.67E-02 | -1.15 (-1.81;-0.48) | 2.15E-03 | -1.16 (-1.83;-0.50) | 1.95E-03 |
| X-21796 | -0.93 (-1.78;-0.08) | 7.37E-02 | -1.19 (-1.87;-0.51) | 1.88E-03 | -1.19 (-1.88;-0.51) | 1.95E-03 |
| X-25343 | 0.93 (0.11;1.75) | 6.24E-02 | 1.16 (0.50;1.82) | 1.76E-03 | 1.16 (0.49;1.82) | 1.98E-03 |
| Dodecenedioate (C12:1-DC)* | -0.92 (-1.75;-0.09) | 6.92E-02 | -1.17 (-1.85;-0.50) | 2.02E-03 | -1.18 (-1.86;-0.50) | 2.02E-03 |
| Decanoylcarnitine (C10) | -0.99 (-1.83;-0.16) | 4.80E-02 | -1.16 (-1.82;-0.49) | 2.10E-03 | -1.16 (-1.83;-0.49) | 2.03E-03 |
| Ectoine | -1.13 (-1.96;-0.29) | 2.31E-02 | -1.15 (-1.82;-0.48) | 2.34E-03 | -1.16 (-1.83;-0.49) | 2.20E-03 |
| Linolenoylcarnitine (C18:3)* | -0.72 (-1.56;0.11) | 1.64E-01 | -1.14 (-1.81;-0.48) | 2.34E-03 | -1.15 (-1.82;-0.48) | 2.36E-03 |
| Retinol (Vitamin A) | -0.28 (-1.10;0.55) | 6.24E-01 | -1.14 (-1.81;-0.47) | 2.62E-03 | -1.16 (-1.83;-0.48) | 2.38E-03 |
| X-23780 | -1.06 (-1.88;-0.24) | 3.06E-02 | -1.14 (-1.80;-0.48) | 2.27E-03 | -1.13 (-1.79;-0.46) | 2.65E-03 |
| 3-Methyl-2-Oxobutyrate | 0.97 (0.15;1.79) | 5.09E-02 | 1.15 (0.48;1.83) | 2.40E-03 | 1.15 (0.47;1.82) | 2.71E-03 |
| X-21736 | -1.28 (-2.10;-0.45) | 8.50E-03 | -1.12 (-1.79;-0.44) | 3.41E-03 | -1.15 (-1.83;-0.47) | 2.71E-03 |
| Tryptophan | 1.06 (0.24;1.89) | 3.08E-02 | 1.13 (0.46;1.81) | 2.92E-03 | 1.14 (0.46;1.82) | 2.79E-03 |
| X-16087 | -1.07 (-1.90;-0.24) | 3.08E-02 | -1.13 (-1.81;-0.46) | 2.99E-03 | -1.14 (-1.82;-0.46) | 2.89E-03 |
| Indolebutyrate | -1.22 (-2.04;-0.40) | 1.22E-02 | -1.11 (-1.78;-0.45) | 2.88E-03 | -1.11 (-1.78;-0.45) | 2.98E-03 |
| Etiocholanolone Glucuronide | -0.71 (-1.55;0.13) | 1.74E-01 | -1.14 (-1.82;-0.46) | 3.18E-03 | -1.15 (-1.84;-0.46) | 2.98E-03 |
| 17alpha-Hydroxypregnanolone Glucuronide | -1.10 (-2.13;-0.07) | 8.13E-02 | -1.39 (-2.23;-0.55) | 3.33E-03 | -1.40 (-2.24;-0.56) | 3.15E-03 |
| Umbelliferone Sulfate | -0.71 (-1.54;0.12) | 1.71E-01 | -1.13 (-1.80;-0.45) | 3.09E-03 | -1.13 (-1.80;-0.45) | 3.16E-03 |
| Guanidinoacetate | 1.18 (0.34;2.03) | 1.84E-02 | 1.13 (0.44;1.83) | 3.75E-03 | 1.16 (0.46;1.86) | 3.30E-03 |
| N-Acetylproline | -1.19 (-2.02;-0.37) | 1.52E-02 | -1.11 (-1.79;-0.43) | 3.83E-03 | -1.12 (-1.81;-0.44) | 3.48E-03 |
| 4-Methoxyphenol Sulfate | -0.99 (-1.81;-0.18) | 4.36E-02 | -1.10 (-1.75;-0.44) | 3.20E-03 | -1.09 (-1.75;-0.43) | 3.52E-03 |
| X-17325 | -1.15 (-1.97;-0.33) | 1.84E-02 | -1.10 (-1.76;-0.44) | 3.37E-03 | -1.10 (-1.76;-0.43) | 3.59E-03 |
| S-1-Pyrroline-5-Carboxylate | 0.08 (-0.75;0.90) | 9.11E-01 | -1.11 (-1.78;-0.44) | 3.25E-03 | -1.10 (-1.78;-0.43) | 3.63E-03 |
| Behenoyl Dihydrosphingomyelin (D18:0/22:0)* | 1.56 (0.75;2.38) | 8.78E-04 | 1.19 (0.47;1.91) | 3.41E-03 | 1.18 (0.46;1.90) | 3.75E-03 |
| Eicosenoylcarnitine (C20:1)* | -0.92 (-1.76;-0.08) | 7.34E-02 | -1.13 (-1.82;-0.44) | 3.67E-03 | -1.13 (-1.82;-0.44) | 3.75E-03 |
| X-18888 | -1.28 (-2.12;-0.43) | 1.03E-02 | -1.10 (-1.79;-0.42) | 4.53E-03 | -1.14 (-1.83;-0.44) | 3.92E-03 |
| Pro-Hydroxy-Pro | -0.64 (-1.46;0.18) | 2.24E-01 | -1.10 (-1.76;-0.43) | 3.41E-03 | -1.10 (-1.77;-0.42) | 3.94E-03 |
| Acetylcarnitine (C2) | -0.90 (-1.72;-0.08) | 7.25E-02 | -1.08 (-1.74;-0.42) | 4.07E-03 | -1.09 (-1.75;-0.42) | 4.03E-03 |
| Vanillic Acid Glycine | -1.15 (-1.97;-0.34) | 1.73E-02 | -1.09 (-1.76;-0.43) | 3.76E-03 | -1.09 (-1.76;-0.42) | 4.08E-03 |
| 7-Alpha-Hydroxy-3-Oxo-4-Cholestenoate (7-Hoca) | -1.48 (-2.31;-0.66) | 1.86E-03 | -1.07 (-1.74;-0.41) | 4.08E-03 | -1.07 (-1.74;-0.41) | 4.19E-03 |
| Heneicosapentaenoate (21:5n3) | 1.32 (0.50;2.15) | 6.47E-03 | 1.09 (0.41;1.77) | 4.76E-03 | 1.11 (0.42;1.79) | 4.26E-03 |
| Tyramine O-Sulfate | -1.27 (-2.09;-0.44) | 9.44E-03 | -1.08 (-1.75;-0.41) | 4.61E-03 | -1.09 (-1.77;-0.42) | 4.35E-03 |
| 2-Hydroxybutyrate/2-Hydroxyisobutyrate | 0.64 (-0.18;1.46) | 2.19E-01 | 1.11 (0.42;1.80) | 4.36E-03 | 1.13 (0.43;1.83) | 4.46E-03 |
| 2-Acetamidophenol Sulfate | -0.59 (-1.41;0.23) | 2.60E-01 | -1.08 (-1.75;-0.41) | 4.43E-03 | -1.08 (-1.75;-0.40) | 4.67E-03 |
| Cis-3,4-Methyleneheptanoylcarnitine | -1.01 (-1.83;-0.19) | 4.10E-02 | -1.06 (-1.73;-0.39) | 5.29E-03 | -1.08 (-1.75;-0.40) | 4.71E-03 |
| Hexanoylcarnitine (C6) | -0.97 (-1.80;-0.14) | 5.28E-02 | -1.07 (-1.74;-0.40) | 5.08E-03 | -1.08 (-1.75;-0.40) | 4.75E-03 |
| X-24494 | -0.28 (-1.10;0.55) | 6.20E-01 | -1.05 (-1.71;-0.38) | 6.00E-03 | -1.06 (-1.73;-0.39) | 5.33E-03 |
| 1-Myristoyl-2-Arachidonoyl-Gpc (14:0/20:4)* | 1.17 (0.33;2.01) | 1.96E-02 | 1.15 (0.42;1.87) | 5.29E-03 | 1.15 (0.42;1.87) | 5.39E-03 |
| X-12830 | -1.19 (-2.01;-0.37) | 1.39E-02 | -1.03 (-1.69;-0.37) | 5.86E-03 | -1.04 (-1.70;-0.38) | 5.44E-03 |
| X-24757 | -1.07 (-1.89;-0.26) | 2.77E-02 | -1.06 (-1.72;-0.39) | 5.03E-03 | -1.05 (-1.72;-0.38) | 5.50E-03 |
| Uridine | 0.88 (0.06;1.70) | 7.83E-02 | 1.04 (0.37;1.70) | 6.06E-03 | 1.05 (0.38;1.72) | 5.56E-03 |
| Threonate | 1.43 (0.60;2.25) | 2.94E-03 | 1.03 (0.36;1.70) | 6.96E-03 | 1.05 (0.38;1.72) | 6.11E-03 |
| X-13866 | -0.68 (-1.50;0.14) | 1.89E-01 | -1.02 (-1.69;-0.36) | 6.82E-03 | -1.04 (-1.70;-0.37) | 6.11E-03 |
| N-Acetylglycine | -0.50 (-1.33;0.33) | 3.50E-01 | -1.09 (-1.79;-0.39) | 6.31E-03 | -1.09 (-1.79;-0.38) | 6.70E-03 |
| N-Acetylputrescine | -0.50 (-1.32;0.32) | 3.45E-01 | -1.04 (-1.70;-0.37) | 6.25E-03 | -1.03 (-1.70;-0.36) | 6.70E-03 |
| 5-Hydroxyhexanoate | -0.90 (-1.72;-0.08) | 7.27E-02 | -1.01 (-1.67;-0.35) | 7.02E-03 | -1.02 (-1.68;-0.36) | 6.74E-03 |
| Dopamine 3-O-Sulfate | -0.87 (-1.69;-0.05) | 8.43E-02 | -1.02 (-1.68;-0.35) | 7.02E-03 | -1.03 (-1.69;-0.36) | 6.84E-03 |
| 1-Palmitoyl-2-Arachidonoyl-GPC (16:0/20:4n6) | 0.89 (0.07;1.71) | 7.45E-02 | 1.08 (0.38;1.77) | 6.53E-03 | 1.07 (0.37;1.77) | 7.03E-03 |
| Tricosanoyl Sphingomyelin (D18:1/23:0)* | 1.64 (0.79;2.48) | 7.48E-04 | 1.19 (0.41;1.97) | 7.39E-03 | 1.20 (0.42;1.99) | 7.13E-03 |
| X-24544 | -1.02 (-1.91;-0.13) | 5.74E-02 | -1.08 (-1.78;-0.37) | 7.24E-03 | -1.08 (-1.79;-0.37) | 7.13E-03 |
| N,N,N-Trimethyl-5-Aminovalerate | -0.83 (-1.65;-0.01) | 9.67E-02 | -1.01 (-1.68;-0.34) | 8.44E-03 | -1.03 (-1.71;-0.36) | 7.16E-03 |
| Histidine | 1.79 (0.97;2.60) | 1.12E-04 | 1.01 (0.35;1.68) | 7.62E-03 | 1.02 (0.35;1.69) | 7.38E-03 |
| X-21733 | 1.35 (0.52;2.19) | 5.65E-03 | 1.04 (0.34;1.74) | 8.88E-03 | 1.06 (0.36;1.76) | 7.59E-03 |
| Taurocholenate Sulfate* | -1.10 (-1.92;-0.28) | 2.35E-02 | -1.01 (-1.68;-0.34) | 8.08E-03 | -1.01 (-1.69;-0.34) | 7.98E-03 |
| X-12844 | -0.56 (-1.38;0.26) | 2.86E-01 | -1.00 (-1.67;-0.33) | 9.08E-03 | -1.02 (-1.69;-0.34) | 8.01E-03 |
| Methylsuccinate | -0.86 (-1.68;-0.04) | 8.43E-02 | -0.97 (-1.63;-0.31) | 9.82E-03 | -0.99 (-1.65;-0.33) | 8.73E-03 |
| X-17354 | -0.57 (-1.39;0.25) | 2.77E-01 | -1.01 (-1.68;-0.33) | 8.88E-03 | -1.01 (-1.69;-0.33) | 8.73E-03 |
| 4-Methyl-2-Oxopentanoate | 1.09 (0.23;1.94) | 3.38E-02 | 1.06 (0.36;1.75) | 7.91E-03 | 1.05 (0.35;1.76) | 8.80E-03 |
| N-Oleoyltaurine | -1.30 (-2.11;-0.48) | 6.72E-03 | -0.97 (-1.63;-0.31) | 9.73E-03 | -0.98 (-1.64;-0.32) | 9.52E-03 |
| Phenol Sulfate | -0.72 (-1.54;0.10) | 1.56E-01 | -0.98 (-1.64;-0.32) | 9.63E-03 | -0.98 (-1.65;-0.32) | 9.55E-03 |
| X-24418 | -0.73 (-1.56;0.11) | 1.61E-01 | -1.00 (-1.68;-0.32) | 9.88E-03 | -1.00 (-1.68;-0.33) | 9.55E-03 |
| 1-Stearoyl-2-Docosahexaenoyl-Gpc (18:0/22:6) | 1.26 (0.44;2.08) | 8.92E-03 | 1.00 (0.32;1.68) | 1.00E-02 | 1.01 (0.33;1.69) | 9.56E-03 |
| Margaroylcarnitine (C17)* | -0.43 (-1.26;0.40) | 4.38E-01 | -1.02 (-1.70;-0.33) | 9.56E-03 | -1.02 (-1.71;-0.33) | 9.69E-03 |
| Lignoceroyl Sphingomyelin (D18:1/24:0) | 1.60 (0.78;2.41) | 6.70E-04 | 1.09 (0.35;1.83) | 1.01E-02 | 1.09 (0.35;1.83) | 1.02E-02 |
| Trimethylamine N-Oxide | -0.97 (-1.79;-0.15) | 4.99E-02 | -0.96 (-1.62;-0.30) | 1.12E-02 | -0.97 (-1.64;-0.31) | 1.03E-02 |
| Indole-3-Carboxylate | -1.27 (-2.09;-0.45) | 8.64E-03 | -0.97 (-1.64;-0.30) | 1.11E-02 | -0.98 (-1.65;-0.31) | 1.04E-02 |
| X-14939 | 0.72 (-0.09;1.54) | 1.55E-01 | 0.98 (0.31;1.65) | 1.02E-02 | 0.98 (0.31;1.65) | 1.05E-02 |
| X-23644 | -0.67 (-1.50;0.16) | 1.98E-01 | -0.98 (-1.65;-0.31) | 1.01E-02 | -0.98 (-1.65;-0.31) | 1.07E-02 |
| 3-Methyl-2-Oxovalerate | 0.75 (-0.11;1.61) | 1.61E-01 | 1.03 (0.33;1.73) | 9.98E-03 | 1.03 (0.32;1.74) | 1.10E-02 |
| 3-Hydroxyisobutyrate | 0.87 (0.04;1.69) | 8.50E-02 | 0.99 (0.31;1.66) | 1.02E-02 | 0.99 (0.31;1.67) | 1.13E-02 |
| Anthranilate | 0.08 (-0.76;0.91) | 9.11E-01 | -0.99 (-1.68;-0.30) | 1.16E-02 | -1.00 (-1.68;-0.31) | 1.14E-02 |
| 3-Methylcrotonylglycine | -0.86 (-1.67;-0.04) | 8.61E-02 | -1.15 (-1.94;-0.35) | 1.15E-02 | -1.15 (-1.95;-0.35) | 1.17E-02 |
| Sphingomyelin (D18:1/20:0, D16:1/22:0)* | 1.42 (0.60;2.24) | 3.10E-03 | 1.00 (0.31;1.69) | 1.16E-02 | 1.00 (0.31;1.69) | 1.17E-02 |
| X-17335 | -0.77 (-1.60;0.05) | 1.30E-01 | -0.95 (-1.63;-0.28) | 1.37E-02 | -0.98 (-1.65;-0.30) | 1.18E-02 |
| Isobutyrylglycine | -0.87 (-1.68;-0.05) | 8.29E-02 | -0.95 (-1.61;-0.29) | 1.19E-02 | -0.96 (-1.62;-0.29) | 1.19E-02 |
| X-22509 | -0.44 (-1.26;0.38) | 4.12E-01 | -0.96 (-1.63;-0.29) | 1.24E-02 | -0.96 (-1.63;-0.29) | 1.22E-02 |
| 3-Hydroxyhexanoate | -1.01 (-1.83;-0.18) | 4.29E-02 | -0.94 (-1.61;-0.28) | 1.39E-02 | -0.96 (-1.63;-0.29) | 1.23E-02 |
| X-15461 | -0.72 (-1.54;0.11) | 1.64E-01 | -0.95 (-1.62;-0.28) | 1.37E-02 | -0.96 (-1.63;-0.29) | 1.23E-02 |
| Glycerophosphoethanolamine | 1.85 (1.03;2.67) | 6.29E-05 | 0.97 (0.28;1.65) | 1.43E-02 | 0.98 (0.29;1.67) | 1.30E-02 |
| 2'-Deoxyuridine | 0.85 (0.03;1.68) | 9.17E-02 | 0.93 (0.26;1.59) | 1.58E-02 | 0.94 (0.27;1.61) | 1.44E-02 |
| Lysine | 1.09 (0.27;1.90) | 2.56E-02 | 0.92 (0.26;1.58) | 1.46E-02 | 0.93 (0.27;1.58) | 1.46E-02 |
| 4-Methylcatechol Sulfate | -0.50 (-1.32;0.32) | 3.50E-01 | -0.91 (-1.57;-0.26) | 1.58E-02 | -0.92 (-1.58;-0.26) | 1.48E-02 |
| 6-Oxopiperidine-2-Carboxylate | -0.68 (-1.50;0.14) | 1.91E-01 | -0.93 (-1.60;-0.26) | 1.61E-02 | -0.94 (-1.61;-0.27) | 1.52E-02 |
| Quinate | -1.08 (-1.90;-0.26) | 2.67E-02 | -0.94 (-1.61;-0.27) | 1.41E-02 | -0.93 (-1.60;-0.26) | 1.52E-02 |
| Caffeine | 1.08 (0.26;1.90) | 2.72E-02 | 0.97 (0.27;1.66) | 1.58E-02 | 0.97 (0.27;1.67) | 1.54E-02 |
| Lactate | 0.77 (-0.06;1.59) | 1.31E-01 | 0.94 (0.27;1.60) | 1.41E-02 | 0.93 (0.26;1.60) | 1.58E-02 |
| 1-Arachidonoyl-GPC (20:4n6)* | 1.26 (0.44;2.07) | 8.78E-03 | 0.95 (0.26;1.63) | 1.64E-02 | 0.95 (0.27;1.64) | 1.59E-02 |
| 1-Palmitoyl-2-Oleoyl-Gpe (16:0/18:1) | -0.82 (-1.64;0.00) | 1.02E-01 | -0.93 (-1.62;-0.25) | 1.80E-02 | -0.96 (-1.64;-0.27) | 1.59E-02 |
| Sphingomyelin (D18:0/20:0, D16:0/22:0)* | 1.41 (0.59;2.23) | 3.34E-03 | 1.00 (0.28;1.72) | 1.58E-02 | 1.00 (0.27;1.72) | 1.64E-02 |
| 1-Stearoyl-2-Arachidonoyl-Gpc (18:0/20:4) | 0.80 (-0.03;1.62) | 1.16E-01 | 0.98 (0.27;1.68) | 1.57E-02 | 0.97 (0.27;1.67) | 1.65E-02 |
| Palmitoylcarnitine (C16) | -0.23 (-1.07;0.60) | 6.83E-01 | -0.94 (-1.62;-0.25) | 1.71E-02 | -0.94 (-1.63;-0.26) | 1.67E-02 |
| P-Cresol Glucuronide* | -0.84 (-1.67;-0.01) | 9.67E-02 | -0.90 (-1.57;-0.23) | 2.06E-02 | -0.92 (-1.60;-0.25) | 1.74E-02 |
| N,N-Dimethylalanine | -0.91 (-1.72;-0.09) | 6.95E-02 | -0.89 (-1.55;-0.23) | 1.89E-02 | -0.91 (-1.57;-0.24) | 1.76E-02 |
| Docosahexaenoate (DHA; 22:6n3) | 1.29 (0.46;2.11) | 8.28E-03 | 0.93 (0.24;1.62) | 1.87E-02 | 0.94 (0.25;1.63) | 1.76E-02 |
| Oleoylcarnitine (C18:1) | -0.67 (-1.49;0.16) | 2.03E-01 | -0.92 (-1.59;-0.25) | 1.74E-02 | -0.92 (-1.59;-0.24) | 1.78E-02 |
| 4-Ethylphenylsulfate | -0.54 (-1.36;0.28) | 3.02E-01 | -0.92 (-1.59;-0.25) | 1.76E-02 | -0.92 (-1.59;-0.24) | 1.79E-02 |
| N-Palmitoylglycine | -0.79 (-1.61;0.03) | 1.20E-01 | -0.92 (-1.59;-0.25) | 1.68E-02 | -0.91 (-1.58;-0.24) | 1.81E-02 |
| X-23787 | -1.12 (-1.96;-0.29) | 2.40E-02 | -0.92 (-1.60;-0.24) | 1.93E-02 | -0.93 (-1.61;-0.25) | 1.81E-02 |
| Pregnanediol-3-Glucuronide | -0.25 (-1.08;0.58) | 6.56E-01 | -0.90 (-1.57;-0.23) | 2.05E-02 | -0.91 (-1.59;-0.24) | 1.86E-02 |
| X-23655 | -0.97 (-1.80;-0.14) | 5.27E-02 | -0.93 (-1.61;-0.24) | 1.86E-02 | -0.93 (-1.62;-0.24) | 1.86E-02 |
| 3-Hydroxydodecanedioate* | -1.05 (-1.89;-0.22) | 3.55E-02 | -0.91 (-1.59;-0.22) | 2.15E-02 | -0.94 (-1.63;-0.25) | 1.87E-02 |
| 3-Formylindole | -0.33 (-1.18;0.52) | 5.68E-01 | -0.94 (-1.64;-0.24) | 1.97E-02 | -0.94 (-1.64;-0.24) | 1.96E-02 |
| 1-Oleoyl-2-Linoleoyl-Gpe (18:1/18:2)* | -0.42 (-1.24;0.40) | 4.37E-01 | -0.88 (-1.54;-0.22) | 2.06E-02 | -0.89 (-1.56;-0.23) | 1.97E-02 |
| Taurochenodeoxycholic Acid 3-Sulfate | -1.15 (-1.96;-0.33) | 1.83E-02 | -0.89 (-1.56;-0.22) | 2.16E-02 | -0.90 (-1.57;-0.22) | 2.11E-02 |
| X-21364 | -0.83 (-1.69;0.03) | 1.16E-01 | -0.90 (-1.59;-0.20) | 2.58E-02 | -0.91 (-1.61;-0.22) | 2.34E-02 |
| X-23974 | -1.02 (-1.84;-0.20) | 3.74E-02 | -0.87 (-1.53;-0.21) | 2.19E-02 | -0.87 (-1.53;-0.21) | 2.35E-02 |
| 1-Carboxyethyltyrosine | -1.60 (-2.44;-0.76) | 8.88E-04 | -1.09 (-1.94;-0.24) | 2.80E-02 | -1.15 (-2.02;-0.27) | 2.38E-02 |
| Gamma-Glutamylthreonine | 0.05 (-0.77;0.87) | 9.36E-01 | -0.86 (-1.52;-0.20) | 2.36E-02 | -0.86 (-1.52;-0.20) | 2.50E-02 |
| X-17612 | -1.02 (-1.85;-0.18) | 4.36E-02 | -0.87 (-1.54;-0.19) | 2.72E-02 | -0.88 (-1.56;-0.20) | 2.50E-02 |
| N-Acetyl-Beta-Alanine | -0.54 (-1.38;0.30) | 3.19E-01 | -0.88 (-1.56;-0.20) | 2.53E-02 | -0.88 (-1.56;-0.20) | 2.53E-02 |
| Glucuronide Of C10h18o2 (7)* | -0.77 (-1.59;0.04) | 1.25E-01 | -0.85 (-1.52;-0.19) | 2.67E-02 | -0.85 (-1.52;-0.19) | 2.69E-02 |
| Butyrylglycine | -0.89 (-1.71;-0.07) | 7.45E-02 | -0.85 (-1.51;-0.19) | 2.74E-02 | -0.85 (-1.52;-0.19) | 2.76E-02 |
| Glycerol | -1.19 (-2.08;-0.31) | 2.39E-02 | -0.91 (-1.64;-0.19) | 2.99E-02 | -0.92 (-1.65;-0.20) | 2.83E-02 |
| Andro Steroid Monosulfate C19h28o6s (1)* | -1.02 (-1.89;-0.16) | 5.08E-02 | -0.89 (-1.58;-0.19) | 2.80E-02 | -0.89 (-1.59;-0.19) | 2.83E-02 |
| 1-Stearoyl-2-Linoleoyl-Gpe (18:0/18:2)* | -0.52 (-1.34;0.30) | 3.26E-01 | -0.84 (-1.52;-0.17) | 3.21E-02 | -0.86 (-1.54;-0.18) | 2.87E-02 |
| Malate | -0.34 (-1.16;0.49) | 5.44E-01 | -0.83 (-1.50;-0.16) | 3.45E-02 | -0.86 (-1.54;-0.18) | 2.87E-02 |
| 4-Hydroxycoumarin | -0.53 (-1.35;0.29) | 3.21E-01 | -0.84 (-1.50;-0.17) | 3.10E-02 | -0.85 (-1.52;-0.18) | 2.88E-02 |
| X-13695 | -0.96 (-1.79;-0.13) | 5.63E-02 | -0.86 (-1.54;-0.18) | 2.86E-02 | -0.86 (-1.54;-0.18) | 3.01E-02 |
| Beta-Hydroxyisovalerate | -0.74 (-1.58;0.11) | 1.60E-01 | -0.84 (-1.52;-0.16) | 3.37E-02 | -0.86 (-1.54;-0.18) | 3.03E-02 |
| 5-Hydroxylysine | -1.33 (-2.15;-0.51) | 5.72E-03 | -0.87 (-1.55;-0.19) | 2.80E-02 | -0.86 (-1.54;-0.18) | 3.04E-02 |
| Indolin-2-One | -0.46 (-1.28;0.36) | 3.90E-01 | -0.84 (-1.51;-0.17) | 3.19E-02 | -0.84 (-1.51;-0.17) | 3.16E-02 |
| Ethylmalonate | -0.49 (-1.32;0.33) | 3.53E-01 | -0.82 (-1.48;-0.16) | 3.33E-02 | -0.83 (-1.49;-0.16) | 3.19E-02 |
| Gamma-Glutamyltyrosine | -0.56 (-1.39;0.26) | 2.86E-01 | -0.83 (-1.50;-0.16) | 3.31E-02 | -0.84 (-1.51;-0.16) | 3.28E-02 |
| Arachidonoylcarnitine (C20:4) | -1.04 (-1.90;-0.18) | 4.42E-02 | -0.86 (-1.55;-0.17) | 3.24E-02 | -0.86 (-1.55;-0.16) | 3.36E-02 |
| 4-Allylphenol Sulfate | -0.63 (-1.45;0.19) | 2.25E-01 | -0.83 (-1.50;-0.16) | 3.24E-02 | -0.83 (-1.50;-0.16) | 3.41E-02 |
| Retinal | -0.78 (-1.60;0.04) | 1.22E-01 | -0.81 (-1.47;-0.15) | 3.57E-02 | -0.81 (-1.47;-0.15) | 3.57E-02 |
| 1-Stearoyl-Gpg (18:0) | -0.48 (-1.30;0.34) | 3.69E-01 | -0.83 (-1.50;-0.15) | 3.67E-02 | -0.83 (-1.51;-0.15) | 3.74E-02 |
| Carotene Diol (2) | 1.70 (0.87;2.53) | 3.01E-04 | 0.83 (0.13;1.54) | 4.30E-02 | 0.86 (0.15;1.56) | 3.78E-02 |
| N-Acetylarginine | -0.21 (-1.03;0.61) | 7.02E-01 | -0.81 (-1.48;-0.15) | 3.65E-02 | -0.81 (-1.47;-0.14) | 3.80E-02 |
| 2-Hydroxyhippurate (Salicylurate) | -0.87 (-1.70;-0.04) | 8.61E-02 | -0.91 (-1.66;-0.16) | 3.69E-02 | -0.90 (-1.65;-0.15) | 3.93E-02 |
| Asparagine | 1.41 (0.59;2.23) | 3.05E-03 | 0.80 (0.12;1.47) | 4.41E-02 | 0.81 (0.13;1.48) | 4.17E-02 |
| Ximenoylcarnitine (C26:1)* | -0.20 (-1.03;0.64) | 7.31E-01 | -0.83 (-1.53;-0.13) | 4.46E-02 | -0.84 (-1.55;-0.13) | 4.28E-02 |
| X-23587 | -0.93 (-1.75;-0.11) | 6.32E-02 | -0.78 (-1.45;-0.11) | 4.72E-02 | -0.80 (-1.47;-0.12) | 4.38E-02 |
| X-21470 | -0.65 (-1.51;0.22) | 2.40E-01 | -0.82 (-1.52;-0.12) | 4.51E-02 | -0.83 (-1.53;-0.13) | 4.48E-02 |
| 1h-Indole-7-Acetic Acid | -0.83 (-1.65;-0.01) | 9.67E-02 | -0.77 (-1.43;-0.11) | 4.83E-02 | -0.78 (-1.45;-0.12) | 4.48E-02 |
| Glutamate | 0.15 (-0.67;0.98) | 7.92E-01 | 0.85 (0.13;1.57) | 4.41E-02 | 0.85 (0.13;1.57) | 4.49E-02 |
| X-25520 | 0.03 (-0.79;0.85) | 9.67E-01 | -0.78 (-1.44;-0.12) | 4.38E-02 | -0.78 (-1.44;-0.12) | 4.56E-02 |
| Xanthurenate | -0.75 (-1.60;0.10) | 1.55E-01 | -0.80 (-1.50;-0.11) | 5.01E-02 | -0.82 (-1.51;-0.12) | 4.70E-02 |
| X-21441 | -0.65 (-1.50;0.19) | 2.25E-01 | -0.80 (-1.48;-0.12) | 4.47E-02 | -0.80 (-1.48;-0.11) | 4.71E-02 |
| 3-Hydroxyhippurate | -0.61 (-1.43;0.21) | 2.42E-01 | -0.78 (-1.44;-0.11) | 4.63E-02 | -0.78 (-1.44;-0.11) | 4.83E-02 |
| Carotene Diol (1) | 1.57 (0.75;2.39) | 9.15E-04 | 0.80 (0.10;1.51) | 5.27E-02 | 0.82 (0.11;1.53) | 4.85E-02 |
| Citrate | -0.54 (-1.35;0.28) | 3.10E-01 | -0.78 (-1.44;-0.11) | 4.92E-02 | -0.78 (-1.45;-0.11) | 4.86E-02 |
| X-15728 | -0.72 (-1.54;0.10) | 1.59E-01 | -0.77 (-1.43;-0.10) | 4.95E-02 | -0.77 (-1.44;-0.10) | 4.97E-02 |
| Pyrraline | -0.67 (-1.49;0.15) | 1.93E-01 | -0.77 (-1.43;-0.11) | 4.69E-02 | -0.76 (-1.42;-0.10) | 5.07E-02 |
| N-Palmitoyl-Heptadecasphingosine (D17:1/16:0)* | -0.38 (-1.22;0.45) | 4.91E-01 | -0.81 (-1.51;-0.11) | 5.03E-02 | -0.81 (-1.52;-0.10) | 5.16E-02 |
| N-Oleoylserine | -0.79 (-1.61;0.03) | 1.16E-01 | -0.77 (-1.43;-0.11) | 4.80E-02 | -0.76 (-1.43;-0.10) | 5.18E-02 |
| Cys-Gly, Oxidized | -0.17 (-0.99;0.66) | 7.74E-01 | -0.75 (-1.42;-0.09) | 5.50E-02 | -0.77 (-1.44;-0.10) | 5.18E-02 |
| Behenoyl Sphingomyelin (D18:1/22:0)* | 1.27 (0.45;2.09) | 8.55E-03 | 0.80 (0.10;1.51) | 5.37E-02 | 0.81 (0.10;1.51) | 5.33E-02 |
| 1-Stearoyl-2-Linoleoyl-Gpi (18:0/18:2) | 0.62 (-0.21;1.45) | 2.40E-01 | -0.80 (-1.50;-0.10) | 5.19E-02 | -0.79 (-1.49;-0.09) | 5.48E-02 |
| Sphingomyelin (D18:0/18:0, D19:0/17:0)* | 0.84 (0.01;1.67) | 9.81E-02 | 0.84 (0.10;1.58) | 5.27E-02 | 0.83 (0.09;1.57) | 5.68E-02 |
| Aspartate | 0.36 (-0.48;1.19) | 5.23E-01 | 0.81 (0.10;1.51) | 5.27E-02 | 0.80 (0.09;1.51) | 5.68E-02 |
| X-11850 | -0.62 (-1.44;0.21) | 2.40E-01 | -0.74 (-1.42;-0.07) | 6.18E-02 | -0.76 (-1.43;-0.08) | 5.73E-02 |
| Homostachydrine* | -1.14 (-1.95;-0.32) | 1.87E-02 | -0.76 (-1.43;-0.09) | 5.59E-02 | -0.76 (-1.43;-0.08) | 5.74E-02 |
| 1-Carboxyethylvaline | -1.33 (-2.16;-0.50) | 6.58E-03 | -0.93 (-1.77;-0.09) | 6.29E-02 | -0.97 (-1.83;-0.10) | 5.81E-02 |
| Linoleoylcarnitine (C18:2)* | -0.09 (-0.92;0.74) | 8.92E-01 | -0.76 (-1.44;-0.09) | 5.59E-02 | -0.76 (-1.44;-0.08) | 5.86E-02 |
| 1-Linoleoyl-2-Arachidonoyl-GPC (18:2/20:4n6)* | 1.51 (0.68;2.34) | 1.74E-03 | 0.80 (0.09;1.52) | 5.87E-02 | 0.81 (0.08;1.53) | 5.87E-02 |
| Eicosapentaenoate (EPA; 20:5n3) | 1.21 (0.38;2.04) | 1.36E-02 | 0.77 (0.08;1.47) | 6.07E-02 | 0.78 (0.08;1.48) | 5.97E-02 |
| X-11381 | -0.21 (-1.03;0.61) | 7.02E-01 | -0.73 (-1.40;-0.07) | 6.31E-02 | -0.74 (-1.41;-0.07) | 6.07E-02 |
| 1,3-Dimethylurate | -0.63 (-1.45;0.19) | 2.28E-01 | -0.74 (-1.40;-0.08) | 6.00E-02 | -0.73 (-1.40;-0.07) | 6.12E-02 |
| Piperine | 0.96 (0.14;1.78) | 5.31E-02 | 0.76 (0.08;1.45) | 6.09E-02 | 0.76 (0.07;1.45) | 6.36E-02 |
| Stearoyl Sphingomyelin (D18:1/18:0) | 0.91 (0.07;1.75) | 7.61E-02 | 0.77 (0.07;1.48) | 6.44E-02 | 0.78 (0.07;1.49) | 6.36E-02 |
| 1-(1-Enyl-Palmitoyl)-2-Oleoyl-Gpe (P-16:0/18:1)* | 0.78 (-0.04;1.60) | 1.24E-01 | 0.74 (0.07;1.40) | 6.11E-02 | 0.73 (0.06;1.40) | 6.52E-02 |
| 1-Palmitoyl-2-Arachidonoyl-Gpe (16:0/20:4)* | -0.55 (-1.37;0.28) | 3.02E-01 | -0.73 (-1.41;-0.05) | 7.12E-02 | -0.75 (-1.44;-0.06) | 6.53E-02 |
| Glycosyl Ceramide (D18:1/23:1, D17:1/24:1)* | -0.36 (-1.19;0.46) | 5.10E-01 | -0.91 (-1.76;-0.07) | 6.99E-02 | -0.94 (-1.80;-0.08) | 6.55E-02 |
| Sphingomyelin (D18:1/25:0, D19:0/24:1, D20:1/23:0, D19:1/24:0)* | 1.32 (0.50;2.14) | 6.21E-03 | 0.75 (0.05;1.44) | 7.08E-02 | 0.77 (0.06;1.47) | 6.55E-02 |
| X-11852 | -0.09 (-0.92;0.73) | 8.83E-01 | -0.72 (-1.38;-0.06) | 6.78E-02 | -0.73 (-1.39;-0.06) | 6.55E-02 |
| X-26097 | -0.35 (-1.17;0.48) | 5.32E-01 | -0.72 (-1.38;-0.06) | 6.78E-02 | -0.71 (-1.38;-0.05) | 7.06E-02 |
| Glucose | 0.49 (-0.33;1.31) | 3.57E-01 | 0.73 (0.05;1.41) | 7.09E-02 | 0.78 (0.05;1.50) | 7.14E-02 |
| X-12111 | 0.11 (-0.71;0.94) | 8.59E-01 | -0.73 (-1.40;-0.05) | 6.91E-02 | -0.72 (-1.40;-0.05) | 7.22E-02 |
| Glycohyocholate | -0.95 (-1.77;-0.13) | 5.63E-02 | -0.72 (-1.40;-0.05) | 7.25E-02 | -0.72 (-1.40;-0.04) | 7.40E-02 |
| Pipecolate | 1.03 (0.21;1.85) | 3.74E-02 | 0.71 (0.05;1.38) | 7.08E-02 | 0.71 (0.04;1.38) | 7.40E-02 |
| 2-Ketocaprylate | -0.35 (-1.18;0.47) | 5.23E-01 | -0.70 (-1.37;-0.04) | 7.65E-02 | -0.71 (-1.37;-0.04) | 7.55E-02 |
| Edta | 0.75 (-0.07;1.58) | 1.37E-01 | 0.70 (0.04;1.37) | 7.60E-02 | 0.70 (0.04;1.37) | 7.74E-02 |
| 1-Stearoyl-2-Arachidonoyl-Gps (18:0/20:4) | 0.87 (0.05;1.69) | 8.17E-02 | 0.83 (0.04;1.63) | 7.75E-02 | 0.84 (0.04;1.63) | 7.74E-02 |
| X-25267 | 0.24 (-0.58;1.06) | 6.71E-01 | 0.69 (0.03;1.35) | 7.97E-02 | 0.69 (0.03;1.35) | 8.01E-02 |
| X-26108 | -0.80 (-1.65;0.06) | 1.31E-01 | -0.71 (-1.40;-0.02) | 8.73E-02 | -0.71 (-1.40;-0.01) | 8.88E-02 |
| Ceramide (D18:2/24:1, D18:1/24:2)* | -0.28 (-1.10;0.55) | 6.20E-01 | -0.70 (-1.39;-0.01) | 9.08E-02 | -0.70 (-1.39;-0.01) | 9.09E-02 |
| Hexadecenedioate (C16:1-DC)* | -0.64 (-1.46;0.19) | 2.28E-01 | -0.68 (-1.36;-0.01) | 9.05E-02 | -0.68 (-1.36;-0.01) | 9.09E-02 |
| Citramalate | -0.69 (-1.51;0.13) | 1.77E-01 | -0.67 (-1.34;0.00) | 9.43E-02 | -0.68 (-1.35;-0.01) | 9.14E-02 |
| Arginine | 0.96 (0.14;1.77) | 5.27E-02 | 0.66 (0.00;1.33) | 9.85E-02 | 0.68 (0.01;1.35) | 9.20E-02 |
| Stearoyl Ethanolamide | -0.63 (-1.47;0.21) | 2.40E-01 | -0.85 (-1.70;-0.01) | 9.18E-02 | -0.85 (-1.70;-0.01) | 9.29E-02 |
| X-12544 | 0.61 (-0.22;1.43) | 2.49E-01 | 0.67 (0.01;1.34) | 9.20E-02 | 0.67 (0.00;1.34) | 9.44E-02 |
| Isovalerylglycine | -0.71 (-1.52;0.11) | 1.64E-01 | -0.65 (-1.31;0.01) | 9.93E-02 | -0.66 (-1.32;0.00) | 9.51E-02 |
| 1-Palmitoyl-2-Linoleoyl-Gpi (16:0/18:2) | -0.17 (-0.99;0.66) | 7.71E-01 | -0.69 (-1.37;-0.01) | 9.05E-02 | -0.68 (-1.37;0.00) | 9.51E-02 |
| X-21607 | 0.79 (-0.03;1.61) | 1.16E-01 | 0.66 (0.00;1.32) | 9.38E-02 | 0.66 (0.00;1.32) | 9.57E-02 |
| 5alpha-Pregnan-3beta,20alpha-Diol Disulfate | -0.90 (-1.75;-0.05) | 8.29E-02 | -0.68 (-1.37;0.00) | 9.64E-02 | -0.68 (-1.37;0.00) | 9.72E-02 |
| X-12851 | -0.61 (-1.43;0.22) | 2.50E-01 | -0.67 (-1.34;0.00) | 9.83E-02 | -0.67 (-1.34;0.00) | 9.75E-02 |
| Glycochenodeoxycholate 3-Sulfate | -1.02 (-1.86;-0.19) | 4.02E-02 | -0.67 (-1.36;0.01) | 9.91E-02 | -0.68 (-1.36;0.00) | 9.76E-02 |
| X-12729 | -0.04 (-0.87;0.78) | 9.49E-01 | -0.65 (-1.31;0.01) | 1.02E-01 | -0.65 (-1.31;0.01) | 1.05E-01 |
| 1-(1-Enyl-Stearoyl)-2-Oleoyl-Gpe (P-18:0/18:1) | 0.81 (-0.02;1.63) | 1.11E-01 | 0.66 (-0.01;1.33) | 1.00E-01 | 0.65 (-0.02;1.32) | 1.06E-01 |
| N-Palmitoyl-Sphinganine (D18:0/16:0) | -0.14 (-0.96;0.68) | 8.12E-01 | -0.68 (-1.40;0.04) | 1.18E-01 | -0.70 (-1.43;0.02) | 1.06E-01 |
| Pantothenate | -0.21 (-1.04;0.61) | 7.02E-01 | -0.65 (-1.31;0.02) | 1.10E-01 | -0.65 (-1.32;0.02) | 1.06E-01 |
| N-Acetylasparagine | -0.69 (-1.51;0.13) | 1.80E-01 | -0.65 (-1.31;0.01) | 1.04E-01 | -0.65 (-1.31;0.02) | 1.07E-01 |
| 3beta,7alpha-Dihydroxy-5-Cholestenoate | -0.75 (-1.58;0.07) | 1.41E-01 | -0.67 (-1.36;0.01) | 1.01E-01 | -0.67 (-1.35;0.02) | 1.07E-01 |
| X-11843 | -0.51 (-1.33;0.32) | 3.44E-01 | -0.64 (-1.32;0.03) | 1.15E-01 | -0.66 (-1.33;0.02) | 1.07E-01 |
| Cysteine Sulfinic Acid | -0.84 (-1.66;-0.02) | 9.27E-02 | -0.66 (-1.34;0.02) | 1.05E-01 | -0.66 (-1.34;0.02) | 1.08E-01 |
| 1-Palmitoyl-2-Oleoyl-Gpi (16:0/18:1)* | -0.05 (-0.88;0.78) | 9.40E-01 | -0.68 (-1.37;0.02) | 1.07E-01 | -0.68 (-1.38;0.02) | 1.08E-01 |
| Threonine | 1.14 (0.32;1.96) | 1.87E-02 | 0.63 (-0.03;1.29) | 1.16E-01 | 0.64 (-0.02;1.30) | 1.11E-01 |
| N-Stearoyltaurine | -0.63 (-1.46;0.19) | 2.25E-01 | -0.64 (-1.32;0.03) | 1.18E-01 | -0.65 (-1.32;0.03) | 1.16E-01 |
| Palmitoyl-Oleoyl-Glycerol (16:0/18:1) [1]* | -1.22 (-2.04;-0.40) | 1.21E-02 | -0.81 (-1.68;0.05) | 1.21E-01 | -0.82 (-1.68;0.05) | 1.19E-01 |
| Taurolithocholate 3-Sulfate | -0.54 (-1.36;0.27) | 3.02E-01 | -0.62 (-1.28;0.05) | 1.27E-01 | -0.63 (-1.30;0.04) | 1.19E-01 |
| Glycerophosphorylcholine (GPC) | 1.84 (1.02;2.66) | 7.35E-05 | 0.65 (-0.06;1.35) | 1.33E-01 | 0.67 (-0.04;1.38) | 1.21E-01 |
| Deoxycholate | 0.80 (-0.02;1.62) | 1.13E-01 | 0.64 (-0.03;1.31) | 1.16E-01 | 0.63 (-0.04;1.30) | 1.21E-01 |
| Gamma-Glutamylglutamate | -1.33 (-2.16;-0.51) | 6.08E-03 | -0.65 (-1.34;0.05) | 1.25E-01 | -0.65 (-1.35;0.04) | 1.21E-01 |
| Oleoyl Ethanolamide | -0.76 (-1.58;0.07) | 1.37E-01 | -0.65 (-1.33;0.03) | 1.15E-01 | -0.64 (-1.33;0.04) | 1.22E-01 |
| Phenylpyruvate | -0.52 (-1.36;0.31) | 3.31E-01 | -0.62 (-1.29;0.05) | 1.27E-01 | -0.63 (-1.30;0.04) | 1.24E-01 |
| Glycosyl-N-(2-Hydroxynervonoyl)-Sphingosine (D18:1/24:1(2oh))* | -1.35 (-2.20;-0.50) | 6.90E-03 | -0.65 (-1.34;0.04) | 1.24E-01 | -0.65 (-1.34;0.05) | 1.24E-01 |
| 1-(1-Enyl-Palmitoyl)-2-Arachidonoyl-Gpc (P-16:0/20:4)* | 0.96 (0.14;1.78) | 5.17E-02 | 0.62 (-0.05;1.29) | 1.28E-01 | 0.62 (-0.05;1.29) | 1.30E-01 |
| Sphingomyelin (D17:2/16:0, D18:2/15:0)* | 0.25 (-0.67;1.18) | 6.89E-01 | -0.79 (-1.65;0.06) | 1.25E-01 | -0.80 (-1.66;0.07) | 1.30E-01 |
| Beta-Alanine | -0.39 (-1.21;0.44) | 4.79E-01 | -0.61 (-1.27;0.06) | 1.34E-01 | -0.61 (-1.28;0.05) | 1.31E-01 |
| Glycosyl Ceramide (D18:2/24:1, D18:1/24:2)* | 0.00 (-0.83;0.83) | 9.98E-01 | -0.68 (-1.42;0.06) | 1.28E-01 | -0.69 (-1.44;0.06) | 1.31E-01 |
| X-11478 | 0.43 (-0.39;1.25) | 4.29E-01 | 0.61 (-0.05;1.28) | 1.29E-01 | 0.61 (-0.05;1.28) | 1.32E-01 |
| 9-Hydroxystearate | -0.91 (-1.76;-0.05) | 8.27E-02 | -0.65 (-1.34;0.05) | 1.27E-01 | -0.64 (-1.34;0.06) | 1.32E-01 |
| Tyrosine | 0.74 (-0.08;1.56) | 1.42E-01 | 0.61 (-0.05;1.27) | 1.28E-01 | 0.61 (-0.05;1.27) | 1.32E-01 |
| 1-Oleoyl-2-Arachidonoyl-Gpe (18:1/20:4)* | -0.42 (-1.24;0.41) | 4.46E-01 | -0.61 (-1.29;0.08) | 1.48E-01 | -0.63 (-1.32;0.06) | 1.32E-01 |
| X-17438 | -0.85 (-1.68;-0.02) | 9.62E-02 | -0.61 (-1.29;0.06) | 1.35E-01 | -0.62 (-1.30;0.06) | 1.34E-01 |
| 5alpha-Pregnan-3beta,20alpha-Diol Monosulfate (2) | -0.69 (-1.52;0.14) | 1.89E-01 | -0.62 (-1.28;0.05) | 1.30E-01 | -0.61 (-1.28;0.06) | 1.34E-01 |
| Hexadecanedioate (C16-DC) | -0.69 (-1.53;0.15) | 1.93E-01 | -0.62 (-1.30;0.07) | 1.39E-01 | -0.62 (-1.31;0.06) | 1.34E-01 |
| 1-Stearoyl-2-Oleoyl-Gpi (18:0/18:1)* | 0.25 (-0.57;1.08) | 6.50E-01 | -0.64 (-1.34;0.06) | 1.32E-01 | -0.64 (-1.34;0.06) | 1.34E-01 |
| X-12740 | -0.57 (-1.39;0.25) | 2.76E-01 | -0.60 (-1.26;0.06) | 1.33E-01 | -0.60 (-1.26;0.06) | 1.35E-01 |
| Propionylcarnitine (C3) | -0.48 (-1.31;0.35) | 3.71E-01 | -0.60 (-1.28;0.07) | 1.45E-01 | -0.62 (-1.30;0.06) | 1.36E-01 |
| X-10458 | -0.52 (-1.34;0.30) | 3.22E-01 | -0.60 (-1.26;0.06) | 1.34E-01 | -0.60 (-1.27;0.06) | 1.36E-01 |
| Sphinganine | 0.67 (-0.15;1.49) | 1.93E-01 | 0.61 (-0.06;1.29) | 1.35E-01 | 0.61 (-0.06;1.29) | 1.36E-01 |
| Adenosine 5'-Monophosphate (Amp) | 1.13 (0.31;1.95) | 2.01E-02 | 0.60 (-0.06;1.27) | 1.35E-01 | 0.60 (-0.06;1.27) | 1.36E-01 |
| X-12306 | -0.18 (-1.00;0.63) | 7.43E-01 | -0.60 (-1.26;0.07) | 1.41E-01 | -0.60 (-1.26;0.07) | 1.41E-01 |
| 1-Stearoyl-2-Oleoyl-Gpe (18:0/18:1) | -0.63 (-1.45;0.19) | 2.26E-01 | -0.59 (-1.28;0.10) | 1.62E-01 | -0.62 (-1.32;0.07) | 1.43E-01 |
| Palmitoyl-Docosahexaenoyl-Glycerol (16:0/22:6) [1]* | 0.50 (-0.32;1.32) | 3.43E-01 | 0.75 (-0.09;1.58) | 1.41E-01 | 0.75 (-0.09;1.59) | 1.43E-01 |
| X-16938 | -0.61 (-1.43;0.21) | 2.43E-01 | -0.71 (-1.51;0.08) | 1.42E-01 | -0.71 (-1.51;0.09) | 1.44E-01 |
| 1-(1-Enyl-Palmitoyl)-2-Linoleoyl-Gpc (P-16:0/18:2)* | 1.51 (0.69;2.34) | 1.61E-03 | 0.63 (-0.10;1.35) | 1.57E-01 | 0.65 (-0.08;1.37) | 1.45E-01 |
| 1-Arachidonoyl-Gpi (20:4)* | 0.87 (0.05;1.69) | 8.24E-02 | 0.61 (-0.07;1.29) | 1.44E-01 | 0.61 (-0.08;1.29) | 1.46E-01 |
| Leucine | 0.46 (-0.40;1.32) | 4.16E-01 | 0.64 (-0.08;1.35) | 1.43E-01 | 0.63 (-0.09;1.36) | 1.54E-01 |
| 1,7-Dimethylurate | -0.45 (-1.28;0.38) | 4.12E-01 | -0.59 (-1.27;0.09) | 1.54E-01 | -0.59 (-1.28;0.09) | 1.55E-01 |
| N-Methylhydroxyproline** | -0.04 (-0.87;0.78) | 9.49E-01 | -0.59 (-1.25;0.08) | 1.50E-01 | -0.58 (-1.25;0.09) | 1.55E-01 |
| Cyclo(Leu-Pro) | 0.54 (-0.28;1.37) | 3.09E-01 | 0.61 (-0.10;1.31) | 1.61E-01 | 0.61 (-0.09;1.32) | 1.56E-01 |
| Linoleoyl-Linoleoyl-Glycerol (18:2/18:2) [1]* | 0.50 (-0.32;1.32) | 3.50E-01 | 0.71 (-0.11;1.53) | 1.57E-01 | 0.71 (-0.11;1.53) | 1.57E-01 |
| 5alpha-Androstan-3beta,17alpha-Diol Disulfate | -0.52 (-1.37;0.34) | 3.50E-01 | -0.62 (-1.32;0.09) | 1.56E-01 | -0.62 (-1.33;0.09) | 1.57E-01 |
| Thioproline | -0.72 (-1.54;0.10) | 1.61E-01 | -0.58 (-1.25;0.09) | 1.57E-01 | -0.58 (-1.25;0.09) | 1.57E-01 |
| Indoleacetoylcarnitine* | 0.64 (-0.19;1.47) | 2.27E-01 | 0.59 (-0.08;1.26) | 1.52E-01 | 0.59 (-0.09;1.26) | 1.57E-01 |
| Arachidonate (20:4n6) | 0.64 (-0.18;1.47) | 2.20E-01 | 0.59 (-0.09;1.27) | 1.58E-01 | 0.59 (-0.09;1.27) | 1.58E-01 |
| 1-Linoleoyl-Gpc (18:2) | 1.49 (0.67;2.31) | 1.73E-03 | 0.60 (-0.12;1.31) | 1.77E-01 | 0.62 (-0.10;1.34) | 1.59E-01 |
| Perfluorooctanoate (PFOA) | -0.38 (-1.21;0.45) | 4.96E-01 | -0.57 (-1.25;0.11) | 1.76E-01 | -0.58 (-1.26;0.10) | 1.66E-01 |
| X-21339 | 0.20 (-0.62;1.03) | 7.17E-01 | 0.58 (-0.10;1.26) | 1.65E-01 | 0.58 (-0.10;1.26) | 1.66E-01 |
| Androstenediol (3beta,17beta) Disulfate (2) | -0.47 (-1.42;0.48) | 4.57E-01 | -0.64 (-1.39;0.11) | 1.68E-01 | -0.64 (-1.40;0.11) | 1.66E-01 |
| 1-Linolenoyl-Gpc (18:3)* | 1.41 (0.59;2.22) | 3.12E-03 | 0.55 (-0.12;1.23) | 1.86E-01 | 0.58 (-0.10;1.26) | 1.68E-01 |
| 3-(3-Hydroxyphenyl)Propionate Sulfate | -0.32 (-1.14;0.50) | 5.68E-01 | -0.57 (-1.23;0.10) | 1.64E-01 | -0.56 (-1.23;0.10) | 1.69E-01 |
| 1-Oleoyl-2-Docosahexaenoyl-Gpc (18:1/22:6)* | 1.34 (0.51;2.17) | 6.08E-03 | 0.58 (-0.12;1.27) | 1.81E-01 | 0.59 (-0.11;1.29) | 1.71E-01 |
| Lactosyl-N-Nervonoyl-Sphingosine (D18:1/24:1)* | -0.40 (-1.22;0.42) | 4.60E-01 | -0.61 (-1.32;0.11) | 1.68E-01 | -0.61 (-1.33;0.12) | 1.72E-01 |
| X-25419 | 0.99 (0.16;1.81) | 4.79E-02 | 0.56 (-0.12;1.23) | 1.84E-01 | 0.56 (-0.12;1.25) | 1.79E-01 |
| 4-Chlorobenzoic Acid | -0.78 (-1.60;0.03) | 1.20E-01 | -0.53 (-1.18;0.13) | 1.95E-01 | -0.54 (-1.20;0.12) | 1.83E-01 |
| X-16964 | -0.40 (-1.22;0.42) | 4.60E-01 | -0.66 (-1.45;0.14) | 1.81E-01 | -0.66 (-1.45;0.14) | 1.83E-01 |
| X-18899 | 0.76 (-0.07;1.59) | 1.39E-01 | 0.55 (-0.13;1.23) | 1.88E-01 | 0.56 (-0.12;1.23) | 1.83E-01 |
| Tetradecanedioate (C14-DC) | -0.37 (-1.21;0.46) | 5.07E-01 | -0.56 (-1.24;0.12) | 1.84E-01 | -0.56 (-1.24;0.12) | 1.84E-01 |
| 1-(1-Enyl-Palmitoyl)-2-Linoleoyl-Gpe (P-16:0/18:2)* | 0.96 (0.14;1.78) | 5.37E-02 | 0.55 (-0.12;1.21) | 1.88E-01 | 0.55 (-0.12;1.22) | 1.85E-01 |
| Dodecadienoate (12:2)* | 0.86 (0.04;1.68) | 8.61E-02 | 0.55 (-0.12;1.22) | 1.84E-01 | 0.55 (-0.12;1.22) | 1.85E-01 |
| Sphingomyelin (D18:1/22:1, D18:2/22:0, D16:1/24:1)* | 1.08 (0.25;1.92) | 3.04E-02 | 0.58 (-0.15;1.30) | 1.98E-01 | 0.59 (-0.13;1.32) | 1.88E-01 |
| X-25172 | 0.62 (-0.21;1.44) | 2.42E-01 | 0.53 (-0.13;1.19) | 1.99E-01 | 0.54 (-0.13;1.21) | 1.92E-01 |
| Alanine | 0.50 (-0.32;1.32) | 3.45E-01 | 0.55 (-0.12;1.21) | 1.84E-01 | 0.54 (-0.13;1.21) | 1.96E-01 |
| Pregnenetriol Disulfate* | -0.65 (-1.57;0.26) | 2.65E-01 | -0.58 (-1.31;0.16) | 2.10E-01 | -0.59 (-1.32;0.15) | 2.03E-01 |
| Ceramide (D18:1/17:0, D17:1/18:0)* | -0.86 (-1.70;-0.03) | 9.11E-02 | -0.55 (-1.26;0.16) | 2.15E-01 | -0.57 (-1.28;0.15) | 2.05E-01 |
| Myristoleate (14:1n5) | -0.58 (-1.47;0.31) | 3.16E-01 | -0.58 (-1.31;0.14) | 1.97E-01 | -0.58 (-1.31;0.16) | 2.07E-01 |
| 1-Palmitoyl-2-Docosahexaenoyl-Gpe (16:0/22:6)* | -0.36 (-1.19;0.48) | 5.24E-01 | -0.54 (-1.24;0.16) | 2.18E-01 | -0.55 (-1.26;0.15) | 2.09E-01 |
| Stearoylcarnitine (C18) | 0.26 (-0.58;1.09) | 6.50E-01 | -0.54 (-1.24;0.15) | 2.13E-01 | -0.54 (-1.24;0.15) | 2.12E-01 |
| 1-Palmitoyl-2-Linoleoyl-Gpc (16:0/18:2) | 1.21 (0.39;2.03) | 1.30E-02 | 0.54 (-0.16;1.24) | 2.14E-01 | 0.55 (-0.16;1.25) | 2.14E-01 |
| N-Acetyl-Aspartyl-Glutamate (Naag) | -0.29 (-1.12;0.55) | 6.17E-01 | -0.53 (-1.19;0.14) | 2.05E-01 | -0.52 (-1.19;0.15) | 2.15E-01 |
| 2-Stearoyl-Gpe (18:0)* | 0.29 (-0.54;1.11) | 6.12E-01 | -0.54 (-1.24;0.15) | 2.12E-01 | -0.54 (-1.23;0.16) | 2.19E-01 |
| Sphingomyelin (D18:2/14:0, D18:1/14:1)* | 0.22 (-0.73;1.17) | 7.32E-01 | -0.67 (-1.54;0.19) | 2.12E-01 | -0.68 (-1.56;0.21) | 2.24E-01 |
| Linoleoyl-Arachidonoyl-Glycerol (18:2/20:4) [1]* | 0.34 (-0.48;1.16) | 5.38E-01 | 0.63 (-0.19;1.45) | 2.24E-01 | 0.63 (-0.19;1.45) | 2.24E-01 |
| 4-Hydroxyglutamate | -1.48 (-2.32;-0.64) | 2.39E-03 | -0.55 (-1.28;0.18) | 2.30E-01 | -0.56 (-1.29;0.18) | 2.26E-01 |
| Docosapentaenoate (N3 DPA; 22:5n3) | 0.76 (-0.07;1.60) | 1.39E-01 | 0.52 (-0.17;1.22) | 2.31E-01 | 0.53 (-0.17;1.23) | 2.26E-01 |
| Bilirubin (Z,Z) | 1.32 (0.50;2.14) | 6.26E-03 | 0.50 (-0.17;1.18) | 2.35E-01 | 0.51 (-0.16;1.19) | 2.28E-01 |
| 2-Hydroxybehenate | 0.52 (-0.30;1.34) | 3.27E-01 | 0.50 (-0.17;1.17) | 2.39E-01 | 0.51 (-0.17;1.18) | 2.34E-01 |
| X-11315 | -0.11 (-0.93;0.72) | 8.68E-01 | -0.51 (-1.18;0.16) | 2.30E-01 | -0.51 (-1.20;0.17) | 2.35E-01 |
| Theophylline | 0.55 (-0.27;1.37) | 2.96E-01 | 0.50 (-0.17;1.18) | 2.39E-01 | 0.50 (-0.18;1.18) | 2.41E-01 |
| 12-Hete | 0.51 (-0.31;1.33) | 3.32E-01 | 0.49 (-0.18;1.15) | 2.46E-01 | 0.49 (-0.17;1.16) | 2.41E-01 |
| Eicosenedioate (C20:1-DC)* | 1.14 (0.32;1.96) | 1.87E-02 | 0.50 (-0.18;1.17) | 2.42E-01 | 0.50 (-0.17;1.17) | 2.41E-01 |
| Isoleucylglycine | -0.07 (-0.90;0.76) | 9.13E-01 | -0.52 (-1.19;0.16) | 2.21E-01 | -0.51 (-1.19;0.18) | 2.44E-01 |
| 3-Phosphoglycerate | 0.98 (0.17;1.80) | 4.56E-02 | 0.49 (-0.17;1.15) | 2.36E-01 | 0.49 (-0.18;1.15) | 2.48E-01 |
| X-24546 | -0.28 (-1.18;0.62) | 6.50E-01 | -0.53 (-1.26;0.20) | 2.56E-01 | -0.54 (-1.27;0.20) | 2.48E-01 |
| Laurate (12:0) | -0.54 (-1.38;0.30) | 3.19E-01 | -0.51 (-1.20;0.18) | 2.39E-01 | -0.51 (-1.20;0.19) | 2.51E-01 |
| 5alpha-Pregnan-3beta,20beta-Diol Monosulfate (1) | -0.43 (-1.26;0.40) | 4.37E-01 | -0.48 (-1.15;0.18) | 2.51E-01 | -0.48 (-1.15;0.18) | 2.56E-01 |
| 1-Palmitoyl-Gpe (16:0) | 0.58 (-0.24;1.40) | 2.70E-01 | -0.50 (-1.18;0.18) | 2.43E-01 | -0.49 (-1.18;0.19) | 2.56E-01 |
| Sphingomyelin (D18:1/18:1, D18:2/18:0) | 0.66 (-0.21;1.53) | 2.31E-01 | 0.53 (-0.22;1.28) | 2.66E-01 | 0.54 (-0.21;1.30) | 2.57E-01 |
| Hydroxy-CMPF* | 1.51 (0.70;2.33) | 1.28E-03 | 0.47 (-0.20;1.14) | 2.70E-01 | 0.48 (-0.19;1.16) | 2.57E-01 |
| X-12847 | -0.51 (-1.35;0.32) | 3.45E-01 | -0.49 (-1.18;0.19) | 2.54E-01 | -0.49 (-1.17;0.20) | 2.67E-01 |
| 3beta-Hydroxy-5-Cholestenoate | -0.08 (-0.90;0.74) | 9.09E-01 | -0.51 (-1.21;0.19) | 2.53E-01 | -0.50 (-1.21;0.21) | 2.67E-01 |
| Taurochenodeoxycholate | -0.88 (-1.70;-0.07) | 7.61E-02 | -0.47 (-1.14;0.19) | 2.64E-01 | -0.47 (-1.14;0.20) | 2.68E-01 |
| 5-Methyluridine (Ribothymidine) | 0.25 (-0.58;1.08) | 6.59E-01 | 0.46 (-0.21;1.13) | 2.78E-01 | 0.47 (-0.20;1.14) | 2.69E-01 |
| 3-Hydroxyoctanoate | -0.65 (-1.49;0.19) | 2.23E-01 | -0.47 (-1.15;0.22) | 2.86E-01 | -0.49 (-1.18;0.21) | 2.70E-01 |
| Ornithine | -0.09 (-0.91;0.73) | 8.85E-01 | -0.48 (-1.14;0.18) | 2.54E-01 | -0.47 (-1.14;0.20) | 2.70E-01 |
| X-18921 | 0.52 (-0.30;1.34) | 3.26E-01 | 0.46 (-0.20;1.12) | 2.71E-01 | 0.47 (-0.20;1.13) | 2.70E-01 |
| X-25957 | 0.45 (-0.37;1.27) | 3.99E-01 | 0.46 (-0.20;1.12) | 2.79E-01 | 0.47 (-0.20;1.13) | 2.70E-01 |
| Glutarate (C5-DC) | -0.46 (-1.28;0.36) | 3.88E-01 | -0.47 (-1.13;0.19) | 2.69E-01 | -0.47 (-1.13;0.20) | 2.70E-01 |
| X-25936 | 0.32 (-0.50;1.15) | 5.62E-01 | 0.46 (-0.20;1.12) | 2.73E-01 | 0.47 (-0.20;1.13) | 2.71E-01 |
| Cis-4-Decenoate (10:1n6)* | 0.69 (-0.13;1.51) | 1.81E-01 | 0.46 (-0.20;1.13) | 2.74E-01 | 0.47 (-0.20;1.13) | 2.72E-01 |
| 2'-O-Methylcytidine | -0.25 (-1.07;0.57) | 6.59E-01 | -0.47 (-1.13;0.20) | 2.74E-01 | -0.47 (-1.14;0.20) | 2.72E-01 |
| Arachidoylcarnitine (C20)* | 0.32 (-0.51;1.15) | 5.68E-01 | -0.49 (-1.21;0.22) | 2.76E-01 | -0.50 (-1.21;0.22) | 2.75E-01 |
| Linoleoyl Ethanolamide | -0.71 (-1.53;0.11) | 1.64E-01 | -0.47 (-1.14;0.20) | 2.69E-01 | -0.46 (-1.13;0.21) | 2.77E-01 |
| Nicotinamide | 0.92 (0.09;1.75) | 6.79E-02 | 0.46 (-0.21;1.13) | 2.82E-01 | 0.47 (-0.21;1.14) | 2.77E-01 |
| Sphingomyelin (D18:1/21:0, D17:1/22:0, D16:1/23:0)* | 1.32 (0.47;2.18) | 8.50E-03 | 0.53 (-0.25;1.30) | 2.87E-01 | 0.54 (-0.24;1.31) | 2.79E-01 |
| X-12462 | 0.86 (0.04;1.68) | 8.61E-02 | 0.46 (-0.20;1.12) | 2.76E-01 | 0.46 (-0.21;1.12) | 2.79E-01 |
| 1-Linoleoyl-Gpe (18:2)* | 0.43 (-0.39;1.25) | 4.25E-01 | -0.48 (-1.17;0.20) | 2.68E-01 | -0.47 (-1.16;0.21) | 2.79E-01 |
| 1-(1-Enyl-Stearoyl)-Gpe (P-18:0)* | 1.34 (0.53;2.16) | 5.02E-03 | 0.47 (-0.23;1.18) | 2.99E-01 | 0.48 (-0.22;1.19) | 2.84E-01 |
| Proline | -0.42 (-1.26;0.42) | 4.46E-01 | -0.45 (-1.13;0.23) | 3.02E-01 | -0.47 (-1.15;0.22) | 2.88E-01 |
| Butyrate/Isobutyrate (4:0) | -0.61 (-1.43;0.21) | 2.40E-01 | -0.44 (-1.10;0.22) | 3.02E-01 | -0.45 (-1.11;0.21) | 2.91E-01 |
| N2-Acetyl,N6,N6-Dimethyllysine | 0.03 (-0.79;0.85) | 9.63E-01 | -0.46 (-1.12;0.21) | 2.82E-01 | -0.45 (-1.11;0.22) | 2.91E-01 |
| 2-Piperidinone | 0.36 (-0.47;1.19) | 5.23E-01 | 0.44 (-0.23;1.12) | 3.06E-01 | 0.45 (-0.22;1.13) | 2.98E-01 |
| Maleate | -0.44 (-1.26;0.38) | 4.14E-01 | -0.44 (-1.11;0.23) | 3.10E-01 | -0.45 (-1.12;0.23) | 3.00E-01 |
| Sphingomyelin (D18:2/24:2)* | 0.30 (-0.57;1.17) | 6.19E-01 | -0.50 (-1.23;0.23) | 2.85E-01 | -0.50 (-1.24;0.25) | 3.00E-01 |
| Beta-Sitosterol | 0.42 (-0.40;1.24) | 4.44E-01 | -0.57 (-1.42;0.29) | 3.00E-01 | -0.57 (-1.43;0.29) | 3.01E-01 |
| Cysteinylglycine | -0.42 (-1.24;0.41) | 4.49E-01 | -0.43 (-1.10;0.23) | 3.16E-01 | -0.44 (-1.11;0.23) | 3.04E-01 |
| 16-Hydroxypalmitate | -0.53 (-1.36;0.30) | 3.20E-01 | -0.45 (-1.12;0.23) | 3.02E-01 | -0.45 (-1.12;0.23) | 3.08E-01 |
| X-18922 | -0.46 (-1.28;0.36) | 3.87E-01 | -0.43 (-1.10;0.23) | 3.13E-01 | -0.44 (-1.11;0.23) | 3.08E-01 |
| 2-Palmitoleoyl-Gpc (16:1)* | 0.57 (-0.25;1.39) | 2.78E-01 | 0.42 (-0.25;1.09) | 3.30E-01 | 0.44 (-0.23;1.11) | 3.14E-01 |
| Ceramide (D16:1/24:1, D18:1/22:1)* | -0.12 (-0.95;0.70) | 8.48E-01 | -0.44 (-1.11;0.24) | 3.15E-01 | -0.44 (-1.11;0.24) | 3.14E-01 |
| Sphingomyelin (D18:1/20:1, D18:2/20:0)* | 0.81 (-0.06;1.68) | 1.32E-01 | 0.45 (-0.28;1.18) | 3.40E-01 | 0.48 (-0.26;1.22) | 3.20E-01 |
| X-12816 | -0.59 (-1.41;0.24) | 2.64E-01 | -0.42 (-1.09;0.24) | 3.24E-01 | -0.43 (-1.09;0.24) | 3.21E-01 |
| Bilirubin (E,E)* | 0.89 (0.07;1.71) | 7.45E-02 | 0.43 (-0.25;1.10) | 3.26E-01 | 0.43 (-0.24;1.11) | 3.23E-01 |
| X-11372 | 0.40 (-0.43;1.23) | 4.68E-01 | 0.43 (-0.25;1.11) | 3.25E-01 | 0.43 (-0.25;1.11) | 3.25E-01 |
| 1-Stearoyl-2-Arachidonoyl-Gpi (18:0/20:4) | 0.85 (0.03;1.68) | 8.97E-02 | 0.45 (-0.25;1.15) | 3.24E-01 | 0.45 (-0.26;1.15) | 3.29E-01 |
| 1-Methylnicotinamide | 0.13 (-0.69;0.95) | 8.37E-01 | -0.41 (-1.07;0.25) | 3.36E-01 | -0.42 (-1.08;0.24) | 3.29E-01 |
| X-24306 | 0.28 (-0.56;1.11) | 6.24E-01 | 0.42 (-0.25;1.09) | 3.30E-01 | 0.42 (-0.25;1.10) | 3.29E-01 |
| Glycocholenate Sulfate* | -0.40 (-1.24;0.44) | 4.77E-01 | -0.43 (-1.11;0.25) | 3.27E-01 | -0.43 (-1.12;0.25) | 3.30E-01 |
| Ursodeoxycholate | -0.01 (-0.84;0.82) | 9.91E-01 | 0.41 (-0.25;1.08) | 3.39E-01 | 0.42 (-0.25;1.09) | 3.30E-01 |
| N-Palmitoyl-Sphingosine (D18:1/16:0) | 0.15 (-0.67;0.97) | 7.99E-01 | -0.44 (-1.15;0.27) | 3.40E-01 | -0.45 (-1.16;0.26) | 3.30E-01 |
| X-22776 | 0.46 (-0.36;1.28) | 3.87E-01 | 0.42 (-0.24;1.08) | 3.30E-01 | 0.42 (-0.25;1.08) | 3.30E-01 |
| (14 Or 15)-Methylpalmitate (A17:0 Or I17:0) | -0.35 (-1.19;0.50) | 5.44E-01 | -0.45 (-1.14;0.25) | 3.22E-01 | -0.44 (-1.13;0.26) | 3.31E-01 |
| Myristate (14:0) | -0.36 (-1.20;0.48) | 5.23E-01 | -0.44 (-1.13;0.25) | 3.25E-01 | -0.43 (-1.13;0.26) | 3.35E-01 |
| Caprylate (8:0) | 0.58 (-0.25;1.40) | 2.75E-01 | 0.42 (-0.25;1.09) | 3.37E-01 | 0.42 (-0.25;1.09) | 3.35E-01 |
| 1-Palmitoleoylglycerol (16:1)* | -0.74 (-1.57;0.08) | 1.48E-01 | -0.44 (-1.14;0.27) | 3.38E-01 | -0.44 (-1.15;0.27) | 3.35E-01 |
| X-12112 | -0.35 (-1.18;0.47) | 5.23E-01 | -0.42 (-1.08;0.25) | 3.30E-01 | -0.41 (-1.08;0.25) | 3.37E-01 |
| Palmitoyl Dihydrosphingomyelin (D18:0/16:0)* | 1.27 (0.45;2.10) | 8.84E-03 | 0.44 (-0.28;1.17) | 3.45E-01 | 0.45 (-0.28;1.18) | 3.40E-01 |
| Ethyl Beta-Glucopyranoside | 0.48 (-0.34;1.31) | 3.64E-01 | -0.43 (-1.10;0.25) | 3.30E-01 | -0.42 (-1.10;0.26) | 3.41E-01 |
| Androstenediol (3beta,17beta) Monosulfate (1) | 0.68 (-0.25;1.61) | 2.49E-01 | 0.46 (-0.29;1.20) | 3.44E-01 | 0.46 (-0.29;1.20) | 3.43E-01 |
| 5alpha-Androstan-3alpha,17beta-Diol Monosulfate (2) | -1.21 (-2.54;0.13) | 1.44E-01 | -0.65 (-1.69;0.39) | 3.37E-01 | -0.64 (-1.69;0.41) | 3.46E-01 |
| X-21310 | -0.03 (-0.85;0.79) | 9.62E-01 | -0.42 (-1.09;0.25) | 3.34E-01 | -0.41 (-1.08;0.26) | 3.46E-01 |
| Isoursodeoxycholate | -0.01 (-0.83;0.81) | 9.86E-01 | 0.41 (-0.26;1.07) | 3.45E-01 | 0.40 (-0.26;1.07) | 3.47E-01 |
| Oleoyl-Arachidonoyl-Glycerol (18:1/20:4) [1]* | 0.22 (-0.60;1.04) | 6.93E-01 | 0.51 (-0.33;1.34) | 3.48E-01 | 0.51 (-0.33;1.34) | 3.47E-01 |
| X-18913 | -0.50 (-1.34;0.34) | 3.60E-01 | -0.41 (-1.08;0.27) | 3.53E-01 | -0.41 (-1.09;0.27) | 3.47E-01 |
| X-24243 | 0.25 (-0.58;1.07) | 6.59E-01 | 0.39 (-0.27;1.06) | 3.66E-01 | 0.41 (-0.26;1.08) | 3.47E-01 |
| Valylglycine | -0.38 (-1.22;0.45) | 4.91E-01 | -0.43 (-1.11;0.25) | 3.25E-01 | -0.42 (-1.10;0.27) | 3.48E-01 |
| 2-Methylserine | -0.33 (-1.15;0.49) | 5.45E-01 | -0.41 (-1.07;0.25) | 3.38E-01 | -0.40 (-1.06;0.26) | 3.49E-01 |
| X-19438 | 0.31 (-0.51;1.14) | 5.74E-01 | 0.41 (-0.26;1.08) | 3.43E-01 | 0.41 (-0.26;1.08) | 3.49E-01 |
| Gamma-Glutamyl-Alpha-Lysine | 0.06 (-0.76;0.88) | 9.21E-01 | -0.40 (-1.06;0.26) | 3.45E-01 | -0.40 (-1.06;0.26) | 3.50E-01 |
| X-24949 | 0.07 (-0.76;0.89) | 9.17E-01 | 0.41 (-0.26;1.08) | 3.49E-01 | 0.41 (-0.27;1.08) | 3.53E-01 |
| X-12849 | -0.21 (-1.03;0.62) | 7.12E-01 | -0.47 (-1.28;0.33) | 3.67E-01 | -0.48 (-1.29;0.33) | 3.59E-01 |
| Androstenediol (3beta,17beta) Monosulfate (2) | -0.39 (-1.21;0.44) | 4.78E-01 | -0.41 (-1.09;0.28) | 3.58E-01 | -0.41 (-1.09;0.28) | 3.59E-01 |
| 10-Undecenoate (11:1n1) | 0.35 (-0.48;1.18) | 5.27E-01 | 0.39 (-0.28;1.06) | 3.70E-01 | 0.40 (-0.28;1.07) | 3.64E-01 |
| Sulfate Of Piperine Metabolite C18h21no3 (3)* | 0.29 (-0.54;1.13) | 6.09E-01 | 0.41 (-0.27;1.09) | 3.52E-01 | 0.40 (-0.28;1.08) | 3.68E-01 |
| Trans-4-Hydroxyproline | -0.80 (-1.62;0.03) | 1.17E-01 | -0.39 (-1.07;0.28) | 3.70E-01 | -0.40 (-1.07;0.28) | 3.68E-01 |
| Pentadecanoate (15:0) | -0.39 (-1.23;0.44) | 4.78E-01 | -0.41 (-1.10;0.28) | 3.58E-01 | -0.40 (-1.09;0.29) | 3.74E-01 |
| X-21319 | 0.17 (-0.65;0.99) | 7.67E-01 | 0.39 (-0.27;1.05) | 3.67E-01 | 0.39 (-0.28;1.05) | 3.74E-01 |
| Palmitoyl-Sphingosine-Phosphoethanolamine (D18:1/16:0) | 0.36 (-0.47;1.19) | 5.23E-01 | -0.43 (-1.16;0.30) | 3.66E-01 | -0.42 (-1.16;0.31) | 3.78E-01 |
| 1-(1-Enyl-Palmitoyl)-Gpe (P-16:0)* | 1.14 (0.33;1.96) | 1.86E-02 | 0.38 (-0.30;1.07) | 3.91E-01 | 0.39 (-0.29;1.08) | 3.80E-01 |
| Mannose | -0.40 (-1.23;0.44) | 4.72E-01 | 0.41 (-0.28;1.11) | 3.58E-01 | 0.41 (-0.30;1.11) | 3.80E-01 |
| 1-(1-Enyl-Palmitoyl)-2-Palmitoleoyl-Gpc (P-16:0/16:1)* | -0.12 (-0.97;0.73) | 8.51E-01 | -0.41 (-1.12;0.30) | 3.71E-01 | -0.41 (-1.12;0.31) | 3.89E-01 |
| Glycosyl Ceramide (D18:1/20:0, D16:1/22:0)* | 0.40 (-0.42;1.22) | 4.61E-01 | -0.41 (-1.16;0.33) | 3.96E-01 | -0.42 (-1.17;0.33) | 3.91E-01 |
| Alpha-Tocopherol | 1.01 (0.19;1.83) | 4.02E-02 | 0.39 (-0.30;1.08) | 3.86E-01 | 0.39 (-0.30;1.08) | 3.92E-01 |
| X-07765 | -0.06 (-0.88;0.76) | 9.20E-01 | -0.38 (-1.05;0.29) | 3.89E-01 | -0.38 (-1.05;0.29) | 3.93E-01 |
| X-13684 | -0.54 (-1.39;0.30) | 3.20E-01 | -0.47 (-1.31;0.37) | 3.94E-01 | -0.47 (-1.31;0.37) | 3.93E-01 |
| Glycolithocholate | -0.57 (-1.39;0.25) | 2.76E-01 | -0.36 (-1.02;0.29) | 4.01E-01 | -0.37 (-1.03;0.29) | 3.95E-01 |
| 1-Stearoyl-Gpe (18:0) | 0.83 (0.00;1.65) | 1.02E-01 | -0.40 (-1.11;0.31) | 3.88E-01 | -0.40 (-1.10;0.31) | 3.95E-01 |
| 3-Hydroxybutyrate (Bhba) | 0.26 (-0.56;1.09) | 6.38E-01 | 0.37 (-0.30;1.05) | 3.96E-01 | 0.38 (-0.30;1.05) | 3.95E-01 |
| Methyl Glucopyranoside (Alpha + Beta) | 0.36 (-0.48;1.19) | 5.23E-01 | -0.39 (-1.07;0.29) | 3.84E-01 | -0.38 (-1.07;0.31) | 3.99E-01 |
| Glycosyl-N-Stearoyl-Sphingosine (D18:1/18:0) | 0.34 (-0.50;1.18) | 5.47E-01 | -0.41 (-1.15;0.33) | 4.04E-01 | -0.41 (-1.16;0.33) | 4.02E-01 |
| Myristoyl Dihydrosphingomyelin (D18:0/14:0)* | 1.19 (0.35;2.02) | 1.74E-02 | 0.39 (-0.34;1.12) | 4.17E-01 | 0.41 (-0.33;1.14) | 4.02E-01 |
| Xanthine | 0.03 (-0.79;0.86) | 9.62E-01 | -0.36 (-1.03;0.31) | 4.11E-01 | -0.37 (-1.04;0.30) | 4.02E-01 |
| Fumarate | -0.03 (-0.85;0.79) | 9.66E-01 | -0.35 (-1.01;0.31) | 4.25E-01 | -0.37 (-1.03;0.30) | 4.02E-01 |
| X-11880 | 0.09 (-0.74;0.92) | 8.91E-01 | 0.38 (-0.30;1.05) | 3.98E-01 | 0.37 (-0.31;1.05) | 4.02E-01 |
| 1-Palmitoyl-2-Dihomo-Linolenoyl-GPC (16:0/20:3n3 Or 6)* | 0.79 (-0.04;1.62) | 1.20E-01 | 0.37 (-0.32;1.06) | 4.10E-01 | 0.38 (-0.31;1.07) | 4.02E-01 |
| N-Palmitoylserine | -0.42 (-1.24;0.40) | 4.42E-01 | -0.37 (-1.03;0.30) | 4.03E-01 | -0.36 (-1.03;0.31) | 4.12E-01 |
| 4-Allylcatechol Sulfate | -0.24 (-1.07;0.58) | 6.66E-01 | -0.37 (-1.03;0.29) | 3.95E-01 | -0.36 (-1.03;0.31) | 4.13E-01 |
| X-26111 | 0.60 (-0.22;1.42) | 2.49E-01 | 0.36 (-0.31;1.02) | 4.11E-01 | 0.36 (-0.31;1.03) | 4.13E-01 |
| 1-Stearoyl-2-Arachidonoyl-Gpe (18:0/20:4) | -0.31 (-1.14;0.51) | 5.78E-01 | -0.36 (-1.06;0.34) | 4.38E-01 | -0.38 (-1.09;0.32) | 4.13E-01 |
| Linoleoyl-Arachidonoyl-Glycerol (18:2/20:4) [2]* | 0.33 (-0.49;1.14) | 5.55E-01 | 0.45 (-0.38;1.27) | 4.15E-01 | 0.45 (-0.38;1.28) | 4.13E-01 |
| Taurocholate | -0.56 (-1.38;0.26) | 2.86E-01 | -0.35 (-1.01;0.32) | 4.28E-01 | -0.36 (-1.03;0.31) | 4.13E-01 |
| X-23782 | 0.61 (-0.22;1.44) | 2.49E-01 | 0.36 (-0.32;1.04) | 4.25E-01 | 0.37 (-0.32;1.05) | 4.13E-01 |
| Picolinate | 0.51 (-0.34;1.36) | 3.50E-01 | 0.37 (-0.32;1.06) | 4.17E-01 | 0.37 (-0.32;1.06) | 4.19E-01 |
| 5-Dodecenoate (12:1n7) | -0.25 (-1.13;0.63) | 6.74E-01 | -0.39 (-1.11;0.33) | 4.09E-01 | -0.38 (-1.11;0.34) | 4.19E-01 |
| Palmitoleate (16:1n7) | -0.54 (-1.41;0.32) | 3.31E-01 | -0.39 (-1.11;0.33) | 4.10E-01 | -0.38 (-1.10;0.34) | 4.19E-01 |
| 1,3,7-Trimethylurate | -0.27 (-1.10;0.56) | 6.33E-01 | -0.36 (-1.05;0.33) | 4.28E-01 | -0.37 (-1.06;0.33) | 4.19E-01 |
| Arachidate (20:0) | 0.59 (-0.23;1.41) | 2.56E-01 | 0.35 (-0.32;1.01) | 4.33E-01 | 0.36 (-0.32;1.03) | 4.19E-01 |
| 1-(1-Enyl-Oleoyl)-Gpe (P-18:1)* | 1.05 (0.22;1.87) | 3.51E-02 | 0.36 (-0.34;1.05) | 4.36E-01 | 0.36 (-0.33;1.06) | 4.24E-01 |
| Sphingomyelin (D18:1/24:1, D18:2/24:0)* | 0.80 (-0.03;1.62) | 1.16E-01 | 0.37 (-0.36;1.10) | 4.38E-01 | 0.39 (-0.35;1.12) | 4.24E-01 |
| Sphingomyelin (D17:1/14:0, D16:1/15:0)* | 0.74 (-0.16;1.64) | 1.93E-01 | -0.44 (-1.26;0.37) | 4.10E-01 | -0.44 (-1.27;0.39) | 4.24E-01 |
| Hydroxypalmitoyl Sphingomyelin (D18:1/16:0(Oh))** | 0.39 (-0.43;1.22) | 4.73E-01 | -0.39 (-1.11;0.32) | 4.06E-01 | -0.38 (-1.11;0.34) | 4.24E-01 |
| Stearidonate (18:4n3) | 0.30 (-0.55;1.15) | 6.07E-01 | 0.35 (-0.34;1.05) | 4.38E-01 | 0.36 (-0.33;1.06) | 4.25E-01 |
| Tryptophan Betaine | 0.51 (-0.33;1.34) | 3.50E-01 | 0.36 (-0.33;1.04) | 4.29E-01 | 0.36 (-0.33;1.04) | 4.25E-01 |
| X-24947 | -0.20 (-1.03;0.63) | 7.25E-01 | -0.36 (-1.03;0.31) | 4.21E-01 | -0.35 (-1.02;0.32) | 4.29E-01 |
| 16a-Hydroxy DHEA 3-Sulfate | -0.62 (-1.49;0.26) | 2.72E-01 | -0.36 (-1.06;0.34) | 4.37E-01 | -0.36 (-1.07;0.34) | 4.32E-01 |
| 4-Hydroxychlorothalonil | 0.67 (-0.16;1.50) | 2.04E-01 | 0.35 (-0.33;1.03) | 4.35E-01 | 0.35 (-0.34;1.03) | 4.42E-01 |
| Lignoceroylcarnitine (C24)* | 1.02 (0.20;1.84) | 3.81E-02 | 0.35 (-0.33;1.04) | 4.32E-01 | 0.35 (-0.34;1.03) | 4.42E-01 |
| Palmitoleoyl-Oleoyl-Glycerol (16:1/18:1) [2]* | -0.75 (-1.58;0.08) | 1.43E-01 | -0.42 (-1.25;0.42) | 4.47E-01 | -0.42 (-1.26;0.42) | 4.47E-01 |
| N-Stearoyl-Sphingadienine (D18:2/18:0)* | -0.50 (-1.33;0.34) | 3.60E-01 | -0.35 (-1.08;0.37) | 4.66E-01 | -0.36 (-1.09;0.36) | 4.50E-01 |
| Eugenol Sulfate | -0.28 (-1.12;0.55) | 6.17E-01 | -0.34 (-1.01;0.33) | 4.44E-01 | -0.33 (-1.01;0.34) | 4.52E-01 |
| Pregnenediol Disulfate (C21h34o8s2)* | -0.13 (-1.11;0.84) | 8.57E-01 | -0.38 (-1.16;0.40) | 4.65E-01 | -0.39 (-1.17;0.40) | 4.59E-01 |
| (2-Butoxyethoxy)Acetic Acid | -0.52 (-1.34;0.31) | 3.31E-01 | -0.39 (-1.19;0.40) | 4.56E-01 | -0.39 (-1.19;0.41) | 4.61E-01 |
| Bilirubin (E,Z Or Z,E)* | 1.17 (0.34;2.00) | 1.73E-02 | 0.33 (-0.35;1.00) | 4.66E-01 | 0.33 (-0.35;1.01) | 4.62E-01 |
| Methionine | 0.56 (-0.27;1.40) | 2.95E-01 | 0.34 (-0.34;1.02) | 4.51E-01 | 0.33 (-0.35;1.01) | 4.62E-01 |
| X-11849 | -0.01 (-0.84;0.81) | 9.86E-01 | -0.33 (-0.99;0.33) | 4.48E-01 | -0.33 (-0.99;0.34) | 4.62E-01 |
| Glycoursodeoxycholate | -0.08 (-0.91;0.74) | 9.04E-01 | 0.32 (-0.35;0.98) | 4.71E-01 | 0.32 (-0.34;0.98) | 4.70E-01 |
| Phenylalanine | 0.00 (-0.83;0.82) | 9.95E-01 | -0.32 (-0.99;0.35) | 4.73E-01 | -0.32 (-1.00;0.35) | 4.71E-01 |
| Dihomo-Linoleate (20:2n6) | 0.21 (-0.61;1.03) | 7.11E-01 | 0.32 (-0.35;0.99) | 4.80E-01 | 0.32 (-0.35;0.99) | 4.74E-01 |
| Thyroxine | -0.60 (-1.43;0.22) | 2.51E-01 | -0.34 (-1.01;0.33) | 4.45E-01 | -0.32 (-1.00;0.36) | 4.77E-01 |
| Sphingosine | 0.80 (-0.02;1.62) | 1.10E-01 | 0.33 (-0.35;1.00) | 4.65E-01 | 0.32 (-0.35;0.99) | 4.79E-01 |
| Pregnenetriol Sulfate* | 0.38 (-0.57;1.34) | 5.52E-01 | 0.36 (-0.40;1.12) | 4.77E-01 | 0.36 (-0.40;1.13) | 4.79E-01 |
| Sphingomyelin (D18:1/17:0, D17:1/18:0, D19:1/16:0) | 0.98 (0.14;1.82) | 5.37E-02 | 0.34 (-0.39;1.07) | 4.89E-01 | 0.35 (-0.39;1.09) | 4.80E-01 |
| Fructosyllysine | 0 (-0.82;0.82) | 9.96E-01 | 0.34 (-0.34;1.02) | 4.48E-01 | 0.33 (-0.37;1.03) | 4.86E-01 |
| Bilirubin Degradation Product, C17h18n2o4 (1)** | 0.98 (0.15;1.81) | 5.05E-02 | 0.32 (-0.36;0.99) | 4.80E-01 | 0.32 (-0.36;0.99) | 4.89E-01 |
| Sphingomyelin (D18:1/19:0, D19:1/18:0)* | 0.71 (-0.16;1.57) | 1.93E-01 | 0.35 (-0.41;1.11) | 4.97E-01 | 0.35 (-0.41;1.12) | 4.94E-01 |
| Cerotoylcarnitine (C26)* | 0.56 (-0.27;1.40) | 2.91E-01 | -0.33 (-1.03;0.38) | 4.89E-01 | -0.33 (-1.04;0.38) | 4.94E-01 |
| N1-Methyl-4-Pyridone-3-Carboxamide | -0.46 (-1.28;0.36) | 3.88E-01 | -0.37 (-1.17;0.43) | 4.95E-01 | -0.37 (-1.17;0.44) | 4.95E-01 |
| 1-Oleoyl-Gpi (18:1) | 0.27 (-0.55;1.09) | 6.33E-01 | -0.31 (-1.00;0.37) | 4.97E-01 | -0.31 (-1.00;0.37) | 4.95E-01 |
| Valine | 0.50 (-0.34;1.33) | 3.56E-01 | 0.32 (-0.36;0.99) | 4.83E-01 | 0.31 (-0.37;0.99) | 4.95E-01 |
| X-21258 | -0.57 (-1.40;0.25) | 2.78E-01 | -0.31 (-0.97;0.36) | 4.95E-01 | -0.30 (-0.97;0.36) | 4.95E-01 |
| Taurine | 0.95 (0.13;1.78) | 5.41E-02 | 0.28 (-0.39;0.95) | 5.31E-01 | 0.31 (-0.37;0.99) | 4.97E-01 |
| 1-Stearoyl-Gpi (18:0) | 1.14 (0.31;1.97) | 2.12E-02 | 0.32 (-0.39;1.03) | 5.00E-01 | 0.32 (-0.39;1.03) | 4.98E-01 |
| Gamma-Glutamyl-Epsilon-Lysine | 0.28 (-0.54;1.10) | 6.17E-01 | 0.36 (-0.44;1.15) | 5.00E-01 | 0.36 (-0.43;1.16) | 4.98E-01 |
| 1-Oleoyl-Gpe (18:1) | 0.33 (-0.50;1.15) | 5.57E-01 | -0.31 (-0.97;0.36) | 4.96E-01 | -0.30 (-0.98;0.37) | 4.98E-01 |
| X-21353 | 0.21 (-0.62;1.04) | 7.12E-01 | 0.30 (-0.37;0.97) | 5.01E-01 | 0.30 (-0.37;0.98) | 4.99E-01 |
| Dihomo-Linolenate (20:3n3 Or N6) | 0.29 (-0.54;1.12) | 6.12E-01 | 0.30 (-0.38;0.99) | 5.04E-01 | 0.31 (-0.38;0.99) | 5.00E-01 |
| 2-Hydroxynervonate* | -0.76 (-1.58;0.06) | 1.32E-01 | -0.30 (-0.98;0.37) | 5.00E-01 | -0.30 (-0.98;0.38) | 5.05E-01 |
| Ceramide (D18:1/14:0, D16:1/16:0)* | 0.29 (-0.54;1.12) | 6.12E-01 | -0.31 (-0.99;0.38) | 5.00E-01 | -0.31 (-0.99;0.38) | 5.05E-01 |
| Spermidine | 0.10 (-0.74;0.93) | 8.83E-01 | -0.30 (-0.98;0.37) | 5.02E-01 | -0.30 (-0.98;0.38) | 5.11E-01 |
| Isovalerate (I5:0) | 0.42 (-0.41;1.25) | 4.44E-01 | 0.30 (-0.38;0.97) | 5.10E-01 | 0.29 (-0.38;0.97) | 5.17E-01 |
| Sulfate Of Piperine Metabolite C18h21no3 (1)* | 0.06 (-0.77;0.89) | 9.26E-01 | 0.31 (-0.37;0.98) | 5.00E-01 | 0.30 (-0.38;0.98) | 5.17E-01 |
| Paraxanthine | 0.24 (-0.58;1.07) | 6.66E-01 | 0.29 (-0.39;0.96) | 5.25E-01 | 0.29 (-0.38;0.97) | 5.18E-01 |
| N-Palmitoyl-Sphingadienine (D18:2/16:0)* | 0.22 (-0.61;1.06) | 6.95E-01 | -0.30 (-0.99;0.39) | 5.12E-01 | -0.30 (-0.99;0.39) | 5.18E-01 |
| Ceramide (D18:1/20:0, D16:1/22:0, D20:1/18:0)* | 0.32 (-0.50;1.14) | 5.62E-01 | 0.35 (-0.46;1.16) | 5.20E-01 | 0.35 (-0.46;1.17) | 5.18E-01 |
| (2 Or 3)-Decenoate (10:1n7 Or N8) | 0.46 (-0.36;1.28) | 3.88E-01 | 0.29 (-0.37;0.96) | 5.13E-01 | 0.29 (-0.38;0.95) | 5.18E-01 |
| Isoleucine | 0.00 (-0.87;0.86) | 9.95E-01 | 0.33 (-0.40;1.06) | 5.00E-01 | 0.32 (-0.42;1.05) | 5.18E-01 |
| Pristanate | 0.20 (-0.62;1.03) | 7.21E-01 | -0.30 (-0.97;0.38) | 5.07E-01 | -0.29 (-0.97;0.38) | 5.18E-01 |
| X-22771 | 0.02 (-0.79;0.84) | 9.71E-01 | -0.35 (-1.15;0.45) | 5.17E-01 | -0.35 (-1.15;0.46) | 5.18E-01 |
| Alpha-Ketoglutarate | 0.34 (-0.48;1.16) | 5.38E-01 | 0.30 (-0.37;0.97) | 5.02E-01 | 0.29 (-0.38;0.96) | 5.20E-01 |
| 3-Phenylpropionate (Hydrocinnamate) | 0.37 (-0.45;1.19) | 5.04E-01 | 0.29 (-0.38;0.96) | 5.22E-01 | 0.29 (-0.38;0.96) | 5.21E-01 |
| Oleoyl-Arachidonoyl-Glycerol (18:1/20:4) [2]* | 0.07 (-0.75;0.89) | 9.16E-01 | 0.35 (-0.48;1.18) | 5.25E-01 | 0.36 (-0.48;1.19) | 5.23E-01 |
| 2,3-Dihydroxypyridine | -0.47 (-1.29;0.36) | 3.87E-01 | -0.28 (-0.96;0.39) | 5.30E-01 | -0.29 (-0.96;0.39) | 5.28E-01 |
| Sphingomyelin (D18:1/22:2, D18:2/22:1, D16:1/24:2)* | 0.56 (-0.31;1.43) | 3.20E-01 | -0.32 (-1.06;0.41) | 5.13E-01 | -0.32 (-1.06;0.43) | 5.29E-01 |
| X-11483 | -0.22 (-1.04;0.60) | 6.99E-01 | -0.27 (-0.93;0.39) | 5.35E-01 | -0.28 (-0.94;0.38) | 5.29E-01 |
| X-21467 | -0.47 (-1.29;0.36) | 3.86E-01 | -0.30 (-1.01;0.42) | 5.35E-01 | -0.30 (-1.02;0.41) | 5.29E-01 |
| X-11308 | 0.14 (-0.69;0.98) | 8.10E-01 | 0.29 (-0.39;0.98) | 5.22E-01 | 0.29 (-0.40;0.98) | 5.31E-01 |
| 4-Cholesten-3-One | 0.79 (-0.03;1.62) | 1.20E-01 | 0.30 (-0.39;1.00) | 5.13E-01 | 0.29 (-0.41;0.99) | 5.32E-01 |
| X-21834 | -0.38 (-1.20;0.44) | 4.81E-01 | -0.28 (-0.94;0.38) | 5.29E-01 | -0.28 (-0.94;0.39) | 5.35E-01 |
| 2-Hydroxyarachidate* | -0.73 (-1.54;0.09) | 1.53E-01 | -0.28 (-0.96;0.39) | 5.29E-01 | -0.28 (-0.96;0.40) | 5.42E-01 |
| Deoxycholic Acid Glucuronide | -0.07 (-0.91;0.77) | 9.17E-01 | -0.27 (-0.94;0.41) | 5.55E-01 | -0.28 (-0.96;0.40) | 5.42E-01 |
| Gamma-Glutamylmethionine | -0.07 (-0.90;0.76) | 9.16E-01 | -0.27 (-0.95;0.41) | 5.52E-01 | -0.28 (-0.95;0.40) | 5.47E-01 |
| Glycine | 0.19 (-0.64;1.02) | 7.44E-01 | -0.29 (-0.99;0.40) | 5.29E-01 | -0.29 (-0.99;0.42) | 5.49E-01 |
| Taurodeoxycholic Acid 3-Sulfate | 0.21 (-0.61;1.03) | 7.08E-01 | -0.25 (-0.91;0.41) | 5.83E-01 | -0.27 (-0.93;0.40) | 5.54E-01 |
| Linoleoyl-Linoleoyl-Glycerol (18:2/18:2) [2]* | -0.40 (-1.22;0.42) | 4.66E-01 | -0.32 (-1.13;0.48) | 5.52E-01 | -0.32 (-1.13;0.48) | 5.57E-01 |
| 3-Hydroxylaurate | 0.26 (-0.58;1.10) | 6.50E-01 | 0.27 (-0.41;0.96) | 5.49E-01 | 0.27 (-0.42;0.96) | 5.59E-01 |
| S-Methylcysteine Sulfoxide | 0.13 (-0.69;0.95) | 8.31E-01 | -0.27 (-0.94;0.39) | 5.38E-01 | -0.26 (-0.93;0.40) | 5.59E-01 |
| Hyocholate | -0.07 (-0.90;0.76) | 9.15E-01 | -0.27 (-0.94;0.39) | 5.38E-01 | -0.27 (-0.94;0.41) | 5.60E-01 |
| Gamma-Glutamyl-2-Aminobutyrate | 0.28 (-0.54;1.10) | 6.12E-01 | 0.26 (-0.40;0.92) | 5.57E-01 | 0.26 (-0.40;0.92) | 5.60E-01 |
| 1-Palmitoyl-Gpi (16:0) | 0.65 (-0.19;1.48) | 2.25E-01 | -0.29 (-1.00;0.43) | 5.46E-01 | -0.28 (-1.00;0.43) | 5.60E-01 |
| Branched-Chain, Straight-Chain, Or Cyclopropyl 10:1 Fatty Acid (1)* | -0.20 (-1.03;0.63) | 7.19E-01 | -0.28 (-0.96;0.40) | 5.37E-01 | -0.27 (-0.96;0.42) | 5.61E-01 |
| X-17690 | -0.27 (-1.10;0.56) | 6.38E-01 | -0.27 (-0.94;0.40) | 5.52E-01 | -0.26 (-0.94;0.41) | 5.63E-01 |
| X-21842 | -0.07 (-0.89;0.75) | 9.12E-01 | -0.26 (-0.93;0.41) | 5.58E-01 | -0.26 (-0.93;0.41) | 5.67E-01 |
| Androstenediol (3alpha, 17alpha) Monosulfate (3) | 0.18 (-0.77;1.12) | 7.90E-01 | 0.29 (-0.48;1.06) | 5.73E-01 | 0.30 (-0.47;1.06) | 5.68E-01 |
| 1-Carboxyethylleucine | -0.82 (-1.67;0.02) | 1.13E-01 | -0.33 (-1.18;0.52) | 5.65E-01 | -0.33 (-1.20;0.53) | 5.68E-01 |
| Lithocholate Sulfate (1) | -0.07 (-0.90;0.75) | 9.15E-01 | 0.26 (-0.41;0.93) | 5.58E-01 | 0.26 (-0.41;0.93) | 5.68E-01 |
| Saccharin | -0.26 (-1.08;0.55) | 6.37E-01 | -0.30 (-1.10;0.49) | 5.70E-01 | -0.30 (-1.10;0.49) | 5.69E-01 |
| X-12812 | 0.26 (-0.56;1.09) | 6.42E-01 | -0.24 (-0.91;0.43) | 5.94E-01 | -0.26 (-0.93;0.41) | 5.69E-01 |
| Indolepropionate | 0.38 (-0.44;1.20) | 4.82E-01 | -0.27 (-0.95;0.40) | 5.45E-01 | -0.26 (-0.94;0.42) | 5.69E-01 |
| 1-Palmitoyl-Gpc (16:0) | 1.27 (0.45;2.08) | 8.46E-03 | 0.25 (-0.45;0.95) | 6.01E-01 | 0.27 (-0.44;0.97) | 5.69E-01 |
| X-16935 | -0.48 (-1.31;0.36) | 3.81E-01 | -0.26 (-0.94;0.42) | 5.67E-01 | -0.26 (-0.95;0.42) | 5.69E-01 |
| 3-(3-Hydroxyphenyl)Propionate | 0.29 (-0.53;1.11) | 6.03E-01 | 0.24 (-0.43;0.91) | 5.95E-01 | 0.25 (-0.42;0.92) | 5.73E-01 |
| Palmitoyl-Oleoyl-Glycerol (16:0/18:1) [2]* | -0.77 (-1.59;0.06) | 1.31E-01 | -0.32 (-1.18;0.54) | 5.85E-01 | -0.32 (-1.18;0.54) | 5.82E-01 |
| 1-(1-Enyl-Stearoyl)-2-Linoleoyl-Gpe (P-18:0/18:2)* | 0.81 (-0.01;1.64) | 1.09E-01 | 0.25 (-0.44;0.94) | 5.91E-01 | 0.25 (-0.44;0.94) | 5.87E-01 |
| Palmitoleoyl-Linoleoyl-Glycerol (16:1/18:2) [1]* | -0.51 (-1.33;0.31) | 3.31E-01 | -0.30 (-1.13;0.53) | 5.95E-01 | -0.30 (-1.14;0.53) | 5.92E-01 |
| Taurodeoxycholate | -0.03 (-0.85;0.79) | 9.66E-01 | -0.22 (-0.89;0.44) | 6.22E-01 | -0.24 (-0.91;0.43) | 5.99E-01 |
| Sphingomyelin (D18:2/23:1)* | 0.47 (-0.43;1.36) | 4.29E-01 | -0.29 (-1.07;0.49) | 5.83E-01 | -0.28 (-1.08;0.51) | 6.01E-01 |
| Gamma-Tocopherol/Beta-Tocopherol | 0.58 (-0.24;1.40) | 2.66E-01 | 0.23 (-0.43;0.90) | 6.08E-01 | 0.24 (-0.43;0.90) | 6.02E-01 |
| 2-Aminoadipate | -0.82 (-1.65;0.01) | 1.10E-01 | -0.30 (-1.13;0.54) | 6.00E-01 | -0.30 (-1.13;0.54) | 6.02E-01 |
| Bilirubin Degradation Product, C17h18n2o4 (2)** | 0.86 (0.03;1.69) | 8.85E-02 | 0.24 (-0.43;0.92) | 5.96E-01 | 0.24 (-0.44;0.92) | 6.05E-01 |
| 2-Hydroxy-3-Methylvalerate | -0.11 (-0.96;0.74) | 8.68E-01 | -0.23 (-0.93;0.47) | 6.30E-01 | -0.25 (-0.95;0.46) | 6.05E-01 |
| 1-(1-Enyl-Palmitoyl)-Gpc (P-16:0)* | 0.85 (0.03;1.67) | 8.88E-02 | -0.27 (-0.97;0.44) | 5.73E-01 | -0.25 (-0.97;0.47) | 6.06E-01 |
| Carotene Diol (3) | 0.98 (0.16;1.81) | 4.85E-02 | 0.24 (-0.45;0.92) | 6.11E-01 | 0.24 (-0.45;0.93) | 6.06E-01 |
| X-26109 | -0.58 (-1.41;0.25) | 2.72E-01 | -0.24 (-0.93;0.45) | 6.15E-01 | -0.24 (-0.93;0.45) | 6.11E-01 |
| Myristoyl-Linoleoyl-Glycerol (14:0/18:2) [2]* | -0.62 (-1.43;0.20) | 2.38E-01 | -0.29 (-1.12;0.55) | 6.16E-01 | -0.29 (-1.13;0.55) | 6.13E-01 |
| Erucate (22:1n9) | 0.21 (-0.61;1.03) | 7.08E-01 | 0.22 (-0.45;0.89) | 6.37E-01 | 0.23 (-0.45;0.90) | 6.19E-01 |
| Sphingomyelin (D17:1/16:0, D18:1/15:0, D16:1/17:0)* | 0.64 (-0.20;1.48) | 2.31E-01 | -0.26 (-1.00;0.48) | 6.11E-01 | -0.25 (-1.00;0.49) | 6.19E-01 |
| S-Methylcysteine | 0.13 (-0.69;0.95) | 8.36E-01 | -0.24 (-0.90;0.43) | 6.00E-01 | -0.22 (-0.89;0.44) | 6.21E-01 |
| Sphingomyelin (D18:2/23:0, D18:1/23:1, D17:1/24:1)* | 1.03 (0.15;1.90) | 5.27E-02 | 0.25 (-0.54;1.03) | 6.49E-01 | 0.27 (-0.53;1.07) | 6.21E-01 |
| 5-Oxoproline | -0.24 (-1.05;0.58) | 6.74E-01 | -0.23 (-0.89;0.43) | 6.15E-01 | -0.22 (-0.89;0.44) | 6.21E-01 |
| X-21442 | 0.02 (-0.80;0.84) | 9.72E-01 | 0.23 (-0.43;0.89) | 6.15E-01 | 0.22 (-0.44;0.89) | 6.23E-01 |
| Bilirubin Degradation Product, C17h18n2o4 (3)** | 0.87 (0.05;1.70) | 8.43E-02 | 0.23 (-0.45;0.90) | 6.20E-01 | 0.23 (-0.45;0.91) | 6.24E-01 |
| Phenylacetylcarnitine | 0.01 (-0.82;0.83) | 9.91E-01 | -0.21 (-0.88;0.46) | 6.44E-01 | -0.22 (-0.90;0.45) | 6.26E-01 |
| 1,5-Anhydroglucitol (1,5-Ag) | -0.18 (-1.01;0.65) | 7.52E-01 | -0.23 (-0.90;0.44) | 6.21E-01 | -0.23 (-0.92;0.47) | 6.33E-01 |
| 1-Methylxanthine | -0.29 (-1.12;0.54) | 6.09E-01 | -0.22 (-0.88;0.45) | 6.30E-01 | -0.22 (-0.88;0.45) | 6.35E-01 |
| Glycosyl-N-Behenoyl-Sphingadienine (D18:2/22:0)* | 0.49 (-0.33;1.32) | 3.54E-01 | -0.24 (-0.98;0.50) | 6.37E-01 | -0.24 (-1.00;0.51) | 6.37E-01 |
| (16 Or 17)-Methylstearate (A19:0 Or I19:0) | 0.33 (-0.51;1.16) | 5.64E-01 | 0.21 (-0.47;0.90) | 6.48E-01 | 0.22 (-0.47;0.91) | 6.39E-01 |
| 2-Oxoarginine* | -0.17 (-1.00;0.67) | 7.76E-01 | -0.21 (-0.90;0.47) | 6.49E-01 | -0.22 (-0.91;0.47) | 6.39E-01 |
| Sphingomyelin (D18:2/16:0, D18:1/16:1)* | 0.54 (-0.30;1.39) | 3.21E-01 | -0.25 (-0.98;0.49) | 6.21E-01 | -0.24 (-0.98;0.51) | 6.42E-01 |
| Tartarate | 0.40 (-0.43;1.22) | 4.66E-01 | 0.21 (-0.46;0.87) | 6.50E-01 | 0.21 (-0.45;0.88) | 6.44E-01 |
| 1-Stearoyl-2-Docosahexaenoyl-Gpe (18:0/22:6)* | -0.47 (-1.30;0.36) | 3.87E-01 | -0.22 (-0.94;0.51) | 6.61E-01 | -0.23 (-0.96;0.50) | 6.45E-01 |
| N-Behenoyl-Sphingadienine (D18:2/22:0)* | 0.47 (-0.35;1.30) | 3.80E-01 | 0.26 (-0.57;1.08) | 6.49E-01 | 0.26 (-0.57;1.09) | 6.45E-01 |
| Betaine | 0.62 (-0.21;1.45) | 2.43E-01 | 0.21 (-0.47;0.90) | 6.51E-01 | 0.22 (-0.47;0.91) | 6.45E-01 |
| X-17653 | 0.07 (-0.76;0.89) | 9.16E-01 | 0.21 (-0.46;0.89) | 6.48E-01 | 0.21 (-0.46;0.89) | 6.47E-01 |
| Sphingomyelin (D18:2/18:1)* | 0.63 (-0.23;1.49) | 2.53E-01 | 0.22 (-0.52;0.95) | 6.66E-01 | 0.23 (-0.51;0.98) | 6.49E-01 |
| Cholesterol | 0.90 (0.07;1.73) | 7.47E-02 | 0.26 (-0.56;1.07) | 6.44E-01 | 0.25 (-0.56;1.07) | 6.50E-01 |
| Indoleacetate | -0.28 (-1.10;0.55) | 6.20E-01 | -0.19 (-0.86;0.47) | 6.71E-01 | -0.21 (-0.88;0.46) | 6.50E-01 |
| 10-Heptadecenoate (17:1n7) | -0.34 (-1.19;0.51) | 5.52E-01 | -0.22 (-0.92;0.48) | 6.44E-01 | -0.22 (-0.92;0.49) | 6.52E-01 |
| Sphingomyelin (D18:1/14:0, D16:1/16:0)* | 0.83 (-0.01;1.67) | 1.09E-01 | -0.23 (-0.97;0.50) | 6.44E-01 | -0.23 (-0.97;0.51) | 6.52E-01 |
| N2-Acetyl,N6-Methyllysine | 0.08 (-0.74;0.90) | 9.01E-01 | -0.21 (-0.87;0.45) | 6.44E-01 | -0.20 (-0.86;0.46) | 6.52E-01 |
| Thymol Sulfate | 0.14 (-0.68;0.97) | 8.09E-01 | 0.20 (-0.47;0.86) | 6.65E-01 | 0.20 (-0.46;0.87) | 6.53E-01 |
| Eicosenoate (20:1) | -0.03 (-0.85;0.79) | 9.67E-01 | 0.20 (-0.47;0.87) | 6.69E-01 | 0.20 (-0.47;0.88) | 6.57E-01 |
| 1-Palmitoleoyl-2-Linolenoyl-Gpc (16:1/18:3)* | 0.87 (0.03;1.71) | 8.91E-02 | 0.21 (-0.48;0.89) | 6.61E-01 | 0.21 (-0.48;0.90) | 6.57E-01 |
| X-12798 | 0.59 (-0.23;1.41) | 2.61E-01 | 0.21 (-0.45;0.87) | 6.44E-01 | 0.20 (-0.47;0.87) | 6.58E-01 |
| Octadecanedioate (C18-DC) | -0.57 (-1.41;0.27) | 2.91E-01 | -0.20 (-0.88;0.48) | 6.70E-01 | -0.20 (-0.89;0.48) | 6.58E-01 |
| Glycosyl-N-Palmitoyl-Sphingosine (D18:1/16:0) | 0.55 (-0.27;1.37) | 2.95E-01 | -0.22 (-0.94;0.51) | 6.61E-01 | -0.22 (-0.95;0.51) | 6.60E-01 |
| X-16576 | 0.56 (-0.26;1.38) | 2.87E-01 | 0.19 (-0.48;0.86) | 6.73E-01 | 0.20 (-0.48;0.88) | 6.61E-01 |
| Glycolithocholate Sulfate* | -0.12 (-0.94;0.70) | 8.49E-01 | -0.18 (-0.85;0.48) | 6.92E-01 | -0.20 (-0.87;0.48) | 6.71E-01 |
| Pregnenolone Sulfate | -0.21 (-1.11;0.69) | 7.36E-01 | -0.21 (-0.94;0.51) | 6.65E-01 | -0.21 (-0.94;0.52) | 6.73E-01 |
| Caproate (6:0) | -0.41 (-1.23;0.41) | 4.46E-01 | -0.19 (-0.85;0.46) | 6.66E-01 | -0.19 (-0.85;0.47) | 6.74E-01 |
| Uracil | 0.31 (-0.52;1.13) | 5.85E-01 | 0.19 (-0.48;0.85) | 6.83E-01 | 0.19 (-0.48;0.86) | 6.77E-01 |
| 1-Linoleoyl-2-Linolenoyl-Gpc (18:2/18:3)* | 1.12 (0.29;1.94) | 2.35E-02 | 0.20 (-0.51;0.90) | 6.88E-01 | 0.20 (-0.51;0.91) | 6.79E-01 |
| 1-Linoleoyl-Gpg (18:2)* | 0.47 (-0.35;1.29) | 3.80E-01 | 0.19 (-0.48;0.87) | 6.73E-01 | 0.19 (-0.49;0.86) | 6.83E-01 |
| 3-Hydroxystachydrine* | 0.43 (-0.39;1.25) | 4.32E-01 | -0.20 (-0.87;0.47) | 6.66E-01 | -0.19 (-0.86;0.49) | 6.83E-01 |
| Dehydroepiandrosterone Sulfate (Dhea-S) | 0.48 (-0.44;1.39) | 4.32E-01 | 0.20 (-0.53;0.93) | 6.91E-01 | 0.20 (-0.53;0.94) | 6.86E-01 |
| Stearoyl-Arachidonoyl-Glycerol (18:0/20:4) [2]* | 0.39 (-0.44;1.21) | 4.78E-01 | 0.23 (-0.59;1.05) | 6.84E-01 | 0.23 (-0.59;1.05) | 6.86E-01 |
| X-24307 | -0.24 (-1.08;0.59) | 6.66E-01 | 0.18 (-0.49;0.84) | 7.02E-01 | 0.19 (-0.49;0.86) | 6.86E-01 |
| 3-Carboxy-4-Methyl-5-Propyl-2-Furanpropanoate (Cmpf) | 0.84 (0.02;1.66) | 9.18E-02 | 0.18 (-0.49;0.85) | 7.01E-01 | 0.18 (-0.49;0.85) | 6.91E-01 |
| Sphingomyelin (D18:1/20:2, D18:2/20:1, D16:1/22:2)* | 0.12 (-0.75;1.00) | 8.53E-01 | -0.20 (-0.92;0.52) | 6.88E-01 | -0.19 (-0.92;0.54) | 7.00E-01 |
| Theobromine | -0.07 (-0.89;0.75) | 9.16E-01 | -0.18 (-0.84;0.48) | 6.88E-01 | -0.18 (-0.84;0.49) | 7.04E-01 |
| Androsterone Sulfate | -0.13 (-0.97;0.72) | 8.44E-01 | -0.19 (-0.87;0.50) | 6.93E-01 | -0.18 (-0.87;0.51) | 7.04E-01 |
| 2-Hydroxypalmitate | 0.76 (-0.06;1.58) | 1.32E-01 | 0.17 (-0.51;0.85) | 7.21E-01 | 0.18 (-0.50;0.86) | 7.04E-01 |
| 1-Stearoyl-2-Linoleoyl-Gpc (18:0/18:2)* | 0.93 (0.10;1.76) | 6.74E-02 | 0.19 (-0.53;0.91) | 7.10E-01 | 0.19 (-0.53;0.91) | 7.05E-01 |
| 2-Hydroxystearate | 0.61 (-0.22;1.43) | 2.47E-01 | 0.17 (-0.51;0.85) | 7.21E-01 | 0.18 (-0.50;0.86) | 7.05E-01 |
| Stearate (18:0) | 0.13 (-0.69;0.95) | 8.29E-01 | 0.17 (-0.50;0.84) | 7.17E-01 | 0.18 (-0.50;0.85) | 7.06E-01 |
| Methionine Sulfoxide | 0.26 (-0.56;1.08) | 6.44E-01 | 0.18 (-0.50;0.85) | 7.06E-01 | 0.17 (-0.50;0.85) | 7.08E-01 |
| 1-Palmitoyl-2-Stearoyl-Gpc (16:0/18:0) | 1.12 (0.30;1.94) | 2.15E-02 | 0.18 (-0.52;0.88) | 7.10E-01 | 0.18 (-0.52;0.88) | 7.08E-01 |
| 2-Aminooctanoate | 0.71 (-0.12;1.54) | 1.71E-01 | 0.17 (-0.51;0.85) | 7.21E-01 | 0.17 (-0.51;0.85) | 7.11E-01 |
| 3b-Hydroxy-5-Cholenoic Acid | 0.20 (-0.62;1.02) | 7.17E-01 | 0.15 (-0.52;0.82) | 7.48E-01 | 0.17 (-0.51;0.84) | 7.26E-01 |
| Diacylglycerol (16:1/18:2 [2], 16:0/18:3 [1])* | -0.46 (-1.28;0.36) | 3.87E-01 | -0.20 (-1.04;0.64) | 7.33E-01 | -0.21 (-1.05;0.64) | 7.26E-01 |
| Alpha-Hydroxyisovalerate | 0.36 (-0.49;1.22) | 5.24E-01 | 0.18 (-0.53;0.88) | 7.17E-01 | 0.17 (-0.54;0.88) | 7.36E-01 |
| Stachydrine | 0.40 (-0.42;1.22) | 4.61E-01 | -0.16 (-0.83;0.51) | 7.34E-01 | -0.16 (-0.83;0.52) | 7.44E-01 |
| Corticosterone | -0.28 (-1.10;0.54) | 6.20E-01 | -0.15 (-0.82;0.53) | 7.60E-01 | -0.16 (-0.83;0.52) | 7.44E-01 |
| X-17010 | -0.27 (-1.09;0.55) | 6.33E-01 | 0.15 (-0.51;0.81) | 7.51E-01 | 0.15 (-0.51;0.81) | 7.45E-01 |
| Phenylacetate | 0.31 (-0.52;1.13) | 5.86E-01 | 0.16 (-0.51;0.84) | 7.32E-01 | 0.16 (-0.53;0.84) | 7.46E-01 |
| Butyrylcarnitine (C4) | -0.22 (-1.05;0.60) | 6.89E-01 | -0.15 (-0.81;0.52) | 7.60E-01 | -0.15 (-0.82;0.52) | 7.46E-01 |
| Docosadienoate (22:2n6) | 0.08 (-0.74;0.90) | 9.10E-01 | 0.15 (-0.52;0.82) | 7.54E-01 | 0.15 (-0.52;0.83) | 7.46E-01 |
| Glutamine | 0.78 (-0.04;1.60) | 1.21E-01 | 0.14 (-0.53;0.81) | 7.70E-01 | 0.15 (-0.52;0.83) | 7.46E-01 |
| Stearoyl-Arachidonoyl-Glycerol (18:0/20:4) [1]* | 0.16 (-0.67;0.98) | 7.90E-01 | 0.19 (-0.64;1.01) | 7.53E-01 | 0.19 (-0.64;1.01) | 7.50E-01 |
| X-21471 | -0.55 (-1.38;0.28) | 3.02E-01 | -0.16 (-0.86;0.55) | 7.56E-01 | -0.16 (-0.87;0.55) | 7.52E-01 |
| X-24241 | -0.11 (-0.93;0.71) | 8.57E-01 | -0.18 (-0.97;0.62) | 7.54E-01 | -0.18 (-0.98;0.62) | 7.52E-01 |
| X-12456 | -0.40 (-1.23;0.42) | 4.60E-01 | 0.17 (-0.55;0.88) | 7.39E-01 | 0.16 (-0.56;0.88) | 7.55E-01 |
| Glycoursodeoxycholic Acid Sulfate (1) | -0.63 (-1.47;0.21) | 2.40E-01 | -0.15 (-0.83;0.53) | 7.59E-01 | -0.15 (-0.83;0.53) | 7.56E-01 |
| Oleate/Vaccenate (18:1) | -0.27 (-1.09;0.56) | 6.38E-01 | -0.16 (-0.84;0.52) | 7.47E-01 | -0.15 (-0.83;0.53) | 7.58E-01 |
| Oleoyl-Oleoyl-Glycerol (18:1/18:1) [1]* | -0.09 (-0.91;0.73) | 8.85E-01 | 0.17 (-0.65;1.00) | 7.65E-01 | 0.18 (-0.65;1.00) | 7.63E-01 |
| N-Stearoyl-Sphinganine (D18:0/18:0)* | -0.47 (-1.29;0.35) | 3.80E-01 | -0.13 (-0.86;0.59) | 8.06E-01 | -0.16 (-0.89;0.57) | 7.63E-01 |
| Palmitate (16:0) | -0.24 (-1.06;0.59) | 6.74E-01 | -0.15 (-0.82;0.53) | 7.60E-01 | -0.14 (-0.82;0.54) | 7.68E-01 |
| N-Methylproline | 0.60 (-0.21;1.42) | 2.48E-01 | 0.13 (-0.54;0.80) | 7.90E-01 | 0.14 (-0.53;0.81) | 7.70E-01 |
| Cholesterol Sulfate | 0.78 (-0.07;1.63) | 1.38E-01 | -0.15 (-0.89;0.60) | 7.88E-01 | -0.15 (-0.90;0.59) | 7.72E-01 |
| 12,13-Dihome | 0.35 (-0.48;1.17) | 5.33E-01 | -0.15 (-0.83;0.53) | 7.60E-01 | -0.14 (-0.82;0.55) | 7.81E-01 |
| 10-Nonadecenoate (19:1n9) | -0.18 (-1.02;0.65) | 7.52E-01 | -0.14 (-0.83;0.55) | 7.69E-01 | -0.14 (-0.83;0.55) | 7.84E-01 |
| Docosapentaenoate (N6 DPA; 22:5n6) | 0.23 (-0.59;1.06) | 6.75E-01 | -0.13 (-0.79;0.54) | 7.92E-01 | -0.13 (-0.79;0.53) | 7.88E-01 |
| Sphingosine 1-Phosphate | 0.63 (-0.19;1.45) | 2.26E-01 | 0.13 (-0.53;0.79) | 7.87E-01 | 0.13 (-0.54;0.80) | 7.91E-01 |
| Glucuronide Of Piperine Metabolite C17h21no3 (4)* | -0.16 (-0.99;0.68) | 7.92E-01 | -0.12 (-0.81;0.57) | 8.14E-01 | -0.13 (-0.83;0.56) | 7.91E-01 |
| Linolenate [Alpha Or Gamma; (18:3n3 Or 6)] | 0.21 (-0.63;1.05) | 7.12E-01 | 0.12 (-0.56;0.80) | 8.15E-01 | 0.13 (-0.56;0.81) | 7.99E-01 |
| Cis-3,4-Methyleneheptanoate | -0.40 (-1.22;0.42) | 4.61E-01 | -0.11 (-0.78;0.55) | 8.15E-01 | -0.12 (-0.79;0.54) | 8.01E-01 |
| Glycodeoxycholate 3-Sulfate | 0.48 (-0.35;1.30) | 3.72E-01 | 0.14 (-0.52;0.80) | 7.68E-01 | 0.12 (-0.55;0.79) | 8.02E-01 |
| Phytanate | 0.22 (-0.60;1.05) | 6.95E-01 | -0.13 (-0.80;0.54) | 7.92E-01 | -0.12 (-0.80;0.55) | 8.03E-01 |
| Carnitine | 0.04 (-0.78;0.86) | 9.52E-01 | -0.11 (-0.78;0.55) | 8.17E-01 | -0.12 (-0.79;0.55) | 8.11E-01 |
| Pregnenediol Sulfate (C21h34o5s)* | 0.47 (-0.49;1.44) | 4.60E-01 | 0.13 (-0.64;0.90) | 8.17E-01 | 0.14 (-0.63;0.90) | 8.11E-01 |
| Nonadecanoate (19:0) | 0.30 (-0.52;1.12) | 5.90E-01 | 0.11 (-0.56;0.79) | 8.22E-01 | 0.12 (-0.56;0.79) | 8.16E-01 |
| X-11847 | 0.39 (-0.43;1.21) | 4.78E-01 | -0.12 (-0.78;0.54) | 8.04E-01 | -0.11 (-0.78;0.55) | 8.18E-01 |
| 1-Linoleoyl-Gpi (18:2)* | 0.88 (0.06;1.70) | 8.06E-02 | 0.12 (-0.57;0.80) | 8.18E-01 | 0.12 (-0.57;0.81) | 8.20E-01 |
| X-18901 | 0.25 (-0.58;1.07) | 6.59E-01 | -0.12 (-0.80;0.56) | 8.15E-01 | -0.11 (-0.79;0.57) | 8.21E-01 |
| 3-Bromo-5-Chloro-2,6-Dihydroxybenzoic Acid* | 0.03 (-0.79;0.85) | 9.67E-01 | 0.12 (-0.55;0.78) | 8.12E-01 | 0.11 (-0.56;0.77) | 8.27E-01 |
| X-23665 | -0.55 (-1.37;0.28) | 3.03E-01 | -0.11 (-0.78;0.56) | 8.22E-01 | -0.11 (-0.78;0.56) | 8.27E-01 |
| 1,2-Dipalmitoyl-Gpc (16:0/16:0) | 0.66 (-0.16;1.48) | 2.04E-01 | -0.11 (-0.80;0.58) | 8.28E-01 | -0.11 (-0.80;0.58) | 8.29E-01 |
| Oleoyl-Linoleoyl-Glycerol (18:1/18:2) [1] | -0.05 (-0.87;0.77) | 9.41E-01 | 0.11 (-0.57;0.79) | 8.26E-01 | 0.11 (-0.58;0.79) | 8.36E-01 |
| Alpha-Hydroxyisocaproate | -0.34 (-1.18;0.50) | 5.51E-01 | -0.09 (-0.78;0.60) | 8.71E-01 | -0.11 (-0.80;0.59) | 8.39E-01 |
| Octadecenedioate (C18:1-DC) | -0.06 (-0.89;0.76) | 9.19E-01 | -0.10 (-0.78;0.58) | 8.41E-01 | -0.10 (-0.78;0.58) | 8.39E-01 |
| Cortisol | 0.23 (-0.60;1.06) | 6.83E-01 | 0.12 (-0.55;0.79) | 8.10E-01 | 0.10 (-0.57;0.78) | 8.43E-01 |
| Branched-Chain, Straight-Chain, Or Cyclopropyl 12:1 Fatty Acid* | 0.39 (-0.44;1.23) | 4.78E-01 | -0.10 (-0.78;0.59) | 8.52E-01 | -0.10 (-0.79;0.58) | 8.44E-01 |
| Eicosanedioate (C20-DC) | 0.17 (-0.66;0.99) | 7.74E-01 | -0.10 (-0.77;0.57) | 8.41E-01 | -0.10 (-0.77;0.57) | 8.45E-01 |
| 1-Myristoyl-2-Palmitoyl-Gpc (14:0/16:0) | 0.51 (-0.32;1.34) | 3.39E-01 | 0.09 (-0.59;0.78) | 8.59E-01 | 0.10 (-0.59;0.79) | 8.45E-01 |
| 1-Stearoyl-Gpc (18:0) | 1.17 (0.35;1.99) | 1.64E-02 | 0.08 (-0.65;0.82) | 8.85E-01 | 0.11 (-0.63;0.85) | 8.46E-01 |
| 1-Arachidonoyl-GPE (20:4n6)* | 0.35 (-0.47;1.17) | 5.23E-01 | 0.09 (-0.58;0.77) | 8.53E-01 | 0.10 (-0.57;0.77) | 8.46E-01 |
| 3-Methylcytidine | -0.58 (-1.43;0.26) | 2.82E-01 | -0.12 (-0.80;0.56) | 8.16E-01 | -0.10 (-0.78;0.59) | 8.50E-01 |
| Palmitoyl-Arachidonoyl-Glycerol (16:0/20:4) [2]* | -0.54 (-1.36;0.28) | 3.02E-01 | -0.11 (-0.94;0.72) | 8.62E-01 | -0.11 (-0.95;0.72) | 8.57E-01 |
| 1-Palmitoyl-2-Oleoyl-Gpc (16:0/18:1) | 0.59 (-0.23;1.41) | 2.61E-01 | 0.09 (-0.60;0.79) | 8.60E-01 | 0.09 (-0.60;0.79) | 8.58E-01 |
| X-22834 | 0.01 (-0.81;0.83) | 9.87E-01 | 0.10 (-0.57;0.76) | 8.45E-01 | 0.09 (-0.58;0.76) | 8.66E-01 |
| 2-Hydroxyglutarate | 0.05 (-0.77;0.87) | 9.40E-01 | -0.10 (-0.90;0.71) | 8.76E-01 | -0.10 (-0.91;0.70) | 8.68E-01 |
| 1-Linoleoylglycerol (18:2) | 0.41 (-0.41;1.23) | 4.46E-01 | 0.09 (-0.59;0.76) | 8.70E-01 | 0.09 (-0.59;0.76) | 8.68E-01 |
| Glyco-Beta-Muricholate** | 0.19 (-0.63;1.01) | 7.30E-01 | -0.07 (-0.74;0.59) | 8.88E-01 | -0.08 (-0.75;0.59) | 8.80E-01 |
| Thymidine | 0.14 (-0.67;0.96) | 8.08E-01 | 0.09 (-0.71;0.88) | 8.88E-01 | 0.10 (-0.71;0.90) | 8.80E-01 |
| Octadecadienedioate (C18:2-DC)* | 0.02 (-0.80;0.84) | 9.77E-01 | -0.08 (-0.74;0.59) | 8.82E-01 | -0.08 (-0.74;0.59) | 8.81E-01 |
| X-11299 | 0.30 (-0.52;1.11) | 6.00E-01 | -0.07 (-0.74;0.59) | 8.88E-01 | -0.08 (-0.74;0.59) | 8.85E-01 |
| 1-(1-Enyl-Palmitoyl)-2-Palmitoyl-Gpc (P-16:0/16:0)* | 0.62 (-0.20;1.44) | 2.38E-01 | 0.07 (-0.63;0.76) | 9.02E-01 | 0.08 (-0.62;0.78) | 8.89E-01 |
| X-25265 | -0.02 (-0.84;0.80) | 9.75E-01 | -0.07 (-0.73;0.59) | 8.93E-01 | -0.07 (-0.73;0.59) | 8.89E-01 |
| X-11795 | 0.20 (-0.63;1.03) | 7.20E-01 | 0.07 (-0.61;0.75) | 9.01E-01 | 0.07 (-0.61;0.76) | 8.91E-01 |
| Lactosyl-N-Behenoyl-Sphingosine (D18:1/22:0)* | 0.29 (-0.54;1.12) | 6.12E-01 | -0.08 (-0.79;0.64) | 8.94E-01 | -0.08 (-0.79;0.64) | 8.91E-01 |
| Sulfate Of Piperine Metabolite C16h19no3 (2)* | 0.07 (-0.76;0.90) | 9.12E-01 | -0.07 (-0.75;0.62) | 9.02E-01 | -0.07 (-0.76;0.62) | 8.94E-01 |
| Glycerol 3-Phosphate | 0.49 (-0.33;1.31) | 3.52E-01 | 0.08 (-0.59;0.74) | 8.83E-01 | 0.06 (-0.60;0.73) | 9.04E-01 |
| Hexadecasphingosine (D16:1)* | 0.01 (-0.81;0.83) | 9.91E-01 | 0.07 (-0.62;0.75) | 9.02E-01 | 0.06 (-0.62;0.75) | 9.08E-01 |
| 2-Palmitoyl-Gpc (16:0)* | 0.97 (0.15;1.79) | 4.97E-02 | -0.07 (-0.77;0.63) | 9.02E-01 | -0.06 (-0.76;0.64) | 9.12E-01 |
| Palmitoyl-Linoleoyl-Glycerol (16:0/18:2) [2]* | -0.25 (-1.08;0.57) | 6.51E-01 | 0.08 (-0.77;0.92) | 9.10E-01 | 0.08 (-0.77;0.92) | 9.12E-01 |
| Palmitoleoyl-Arachidonoyl-Glycerol (16:1/20:4) [2]* | -0.39 (-1.21;0.43) | 4.68E-01 | -0.07 (-0.89;0.75) | 9.10E-01 | -0.07 (-0.89;0.75) | 9.13E-01 |
| 13-Hode + 9-Hode | -0.03 (-0.86;0.79) | 9.66E-01 | -0.06 (-0.73;0.61) | 9.10E-01 | -0.06 (-0.72;0.61) | 9.15E-01 |
| Hexadecadienoate (16:2n6) | 0.01 (-0.84;0.86) | 9.92E-01 | -0.07 (-0.77;0.63) | 9.02E-01 | -0.06 (-0.76;0.64) | 9.15E-01 |
| Trans-Urocanate | 0.10 (-0.72;0.92) | 8.74E-01 | -0.07 (-0.73;0.59) | 9.01E-01 | -0.06 (-0.72;0.61) | 9.15E-01 |
| Dibutyl Sulfosuccinate | 0.10 (-0.72;0.92) | 8.74E-01 | 0.07 (-0.73;0.86) | 9.20E-01 | 0.07 (-0.73;0.86) | 9.16E-01 |
| 2-Hydroxydecanoate | -0.02 (-0.85;0.81) | 9.77E-01 | -0.06 (-0.73;0.60) | 9.09E-01 | -0.06 (-0.72;0.61) | 9.18E-01 |
| X-21286 | 0.29 (-0.53;1.12) | 6.07E-01 | 0.06 (-0.63;0.74) | 9.20E-01 | 0.06 (-0.63;0.74) | 9.19E-01 |
| X-17654 | 0.15 (-0.66;0.97) | 7.90E-01 | 0.05 (-0.62;0.71) | 9.29E-01 | 0.05 (-0.61;0.72) | 9.22E-01 |
| Oleoyl-Linoleoyl-Glycerol (18:1/18:2) [2] | -0.17 (-1.00;0.65) | 7.61E-01 | -0.05 (-0.74;0.64) | 9.29E-01 | -0.05 (-0.74;0.63) | 9.22E-01 |
| Sulfate Of Piperine Metabolite C16h19no3 (3)* | 0.10 (-0.73;0.93) | 8.72E-01 | -0.05 (-0.74;0.64) | 9.29E-01 | -0.05 (-0.74;0.63) | 9.23E-01 |
| Margarate (17:0) | 0.04 (-0.79;0.87) | 9.59E-01 | 0.05 (-0.63;0.72) | 9.33E-01 | 0.05 (-0.63;0.73) | 9.27E-01 |
| Isovalerylcarnitine (C5) | 0 (-0.84;0.84) | 9.98E-01 | 0.06 (-0.63;0.75) | 9.12E-01 | 0.05 (-0.64;0.74) | 9.29E-01 |
| 1-Stearoyl-2-Oleoyl-Gpc (18:0/18:1) | 0.36 (-0.46;1.19) | 5.10E-01 | 0.05 (-0.64;0.74) | 9.26E-01 | 0.05 (-0.64;0.74) | 9.29E-01 |
| Cysteine | 0.23 (-0.59;1.05) | 6.85E-01 | 0.05 (-0.62;0.71) | 9.30E-01 | 0.05 (-0.62;0.71) | 9.34E-01 |
| 3-Hydroxymyristate | 0.08 (-0.76;0.91) | 9.11E-01 | -0.05 (-0.87;0.77) | 9.35E-01 | -0.06 (-0.89;0.77) | 9.34E-01 |
| Biliverdin | 0.61 (-0.23;1.44) | 2.52E-01 | 0.03 (-0.65;0.72) | 9.48E-01 | 0.04 (-0.64;0.73) | 9.37E-01 |
| 1-Lignoceroyl-Gpc (24:0) | 1.42 (0.61;2.24) | 2.81E-03 | -0.06 (-0.84;0.71) | 9.20E-01 | -0.05 (-0.83;0.73) | 9.37E-01 |
| N-Stearoyl-Sphingosine (D18:1/18:0)* | -0.19 (-1.02;0.63) | 7.32E-01 | -0.03 (-0.74;0.68) | 9.59E-01 | -0.04 (-0.76;0.67) | 9.39E-01 |
| Linoleoyl-Linolenoyl-Glycerol (18:2/18:3) [2]* | -0.10 (-0.92;0.72) | 8.73E-01 | -0.05 (-0.87;0.77) | 9.38E-01 | -0.05 (-0.87;0.77) | 9.41E-01 |
| Glycochenodeoxycholate Glucuronide (1) | -0.12 (-0.98;0.74) | 8.56E-01 | 0.05 (-0.65;0.76) | 9.26E-01 | 0.04 (-0.67;0.75) | 9.43E-01 |
| 1-Oleoyl-Gpc (18:1) | 1.02 (0.21;1.84) | 3.71E-02 | 0.02 (-0.68;0.72) | 9.68E-01 | 0.04 (-0.67;0.74) | 9.45E-01 |
| 1-Palmitoyl-2-Arachidonoyl-Gpi (16:0/20:4)* | 0.32 (-0.51;1.14) | 5.68E-01 | -0.05 (-0.74;0.65) | 9.33E-01 | -0.04 (-0.73;0.66) | 9.45E-01 |
| Metabolonic Lactone Sulfate | -0.65 (-1.47;0.17) | 2.13E-01 | -0.04 (-0.75;0.67) | 9.48E-01 | -0.04 (-0.75;0.67) | 9.45E-01 |
| Sarcosine | 0.01 (-0.82;0.83) | 9.94E-01 | 0.03 (-0.63;0.70) | 9.48E-01 | 0.04 (-0.63;0.70) | 9.45E-01 |
| 5alpha-Androstan-3beta,17beta-Diol Disulfate | -0.03 (-1.01;0.94) | 9.67E-01 | -0.04 (-0.85;0.77) | 9.49E-01 | -0.04 (-0.86;0.78) | 9.49E-01 |
| Beta-Citrylglutamate | 0.23 (-0.59;1.05) | 6.77E-01 | -0.02 (-0.69;0.65) | 9.63E-01 | -0.03 (-0.71;0.64) | 9.49E-01 |
| X-24970 | 0.26 (-0.56;1.08) | 6.45E-01 | 0.03 (-0.63;0.69) | 9.59E-01 | 0.03 (-0.63;0.70) | 9.51E-01 |
| Palmitoyl Sphingomyelin (D18:1/16:0) | 0.84 (0.02;1.67) | 9.62E-02 | -0.05 (-0.79;0.69) | 9.33E-01 | -0.04 (-0.78;0.71) | 9.51E-01 |
| 1-Palmitoleoyl-Gpc (16:1)* | 0.67 (-0.15;1.50) | 1.93E-01 | -0.05 (-0.73;0.64) | 9.33E-01 | -0.03 (-0.72;0.66) | 9.55E-01 |
| Androstenediol (3alpha, 17alpha) Monosulfate (2) | -0.18 (-1.08;0.71) | 7.71E-01 | -0.04 (-0.75;0.67) | 9.43E-01 | -0.03 (-0.74;0.68) | 9.55E-01 |
| 5alpha-Androstan-3beta,17beta-Diol Monosulfate (2) | 0.04 (-0.85;0.93) | 9.56E-01 | 0.03 (-0.70;0.76) | 9.59E-01 | 0.03 (-0.70;0.76) | 9.56E-01 |
| 3,5-Dichloro-2,6-Dihydroxybenzoic Acid | -0.03 (-0.85;0.79) | 9.68E-01 | 0.04 (-0.63;0.70) | 9.43E-01 | 0.03 (-0.64;0.70) | 9.56E-01 |
| 3-Hydroxydecanoate | -0.17 (-1.01;0.66) | 7.67E-01 | 0.04 (-0.65;0.72) | 9.48E-01 | 0.03 (-0.66;0.72) | 9.57E-01 |
| X-25271 | 0.34 (-0.49;1.18) | 5.38E-01 | 0.04 (-0.63;0.71) | 9.46E-01 | 0.03 (-0.64;0.70) | 9.59E-01 |
| 9,10-Dihome | 0.27 (-0.55;1.09) | 6.33E-01 | -0.04 (-0.70;0.63) | 9.46E-01 | -0.03 (-0.70;0.64) | 9.60E-01 |
| Gamma-Glutamylglutamine | 0.55 (-0.27;1.37) | 2.96E-01 | 0.02 (-0.66;0.69) | 9.76E-01 | 0.03 (-0.65;0.70) | 9.62E-01 |
| 5alpha-Androstan-3alpha,17beta-Diol Monosulfate (1) | -0.12 (-0.97;0.72) | 8.48E-01 | -0.03 (-0.71;0.66) | 9.59E-01 | -0.02 (-0.71;0.67) | 9.67E-01 |
| Caprate (10:0) | 0.34 (-0.50;1.18) | 5.44E-01 | 0.01 (-0.68;0.71) | 9.78E-01 | 0.02 (-0.68;0.72) | 9.67E-01 |
| 1,2-Dilinoleoyl-Gpc (18:2/18:2) | 1.24 (0.41;2.07) | 1.17E-02 | 0.01 (-0.72;0.75) | 9.78E-01 | 0.02 (-0.72;0.77) | 9.68E-01 |
| Sphingomyelin (D18:2/21:0, D16:2/23:0)* | 0.52 (-0.38;1.43) | 3.71E-01 | -0.03 (-0.83;0.76) | 9.58E-01 | -0.03 (-0.83;0.78) | 9.68E-01 |
| Tetradecadienoate (14:2)* | 0.05 (-0.79;0.88) | 9.46E-01 | -0.03 (-0.70;0.65) | 9.59E-01 | -0.02 (-0.70;0.66) | 9.68E-01 |
| Campesterol | 0.45 (-0.37;1.27) | 4.06E-01 | 0.01 (-0.69;0.72) | 9.80E-01 | 0.02 (-0.69;0.73) | 9.69E-01 |
| Sphinganine-1-Phosphate | 0.30 (-0.53;1.12) | 6.00E-01 | 0.02 (-0.64;0.69) | 9.66E-01 | 0.02 (-0.65;0.69) | 9.72E-01 |
| Epiandrosterone Sulfate | 0.07 (-0.81;0.95) | 9.20E-01 | -0.02 (-0.74;0.70) | 9.73E-01 | -0.02 (-0.74;0.71) | 9.74E-01 |
| Androstenediol (3beta,17beta) Disulfate (1) | 0.29 (-0.63;1.21) | 6.46E-01 | -0.01 (-0.78;0.76) | 9.80E-01 | -0.02 (-0.79;0.75) | 9.74E-01 |
| Lactosyl-N-Palmitoyl-Sphingosine (D18:1/16:0) | 0.22 (-0.60;1.04) | 6.95E-01 | -0.03 (-0.72;0.66) | 9.57E-01 | -0.02 (-0.72;0.68) | 9.74E-01 |
| Sphingomyelin (D18:2/24:1, D18:1/24:2)* | 0.53 (-0.32;1.37) | 3.31E-01 | -0.04 (-0.76;0.69) | 9.49E-01 | -0.02 (-0.75;0.72) | 9.74E-01 |
| Glycochenodeoxycholate | -0.21 (-1.03;0.62) | 7.12E-01 | -0.02 (-0.69;0.65) | 9.71E-01 | -0.01 (-0.68;0.66) | 9.74E-01 |
| Salicylate | -0.07 (-0.89;0.76) | 9.17E-01 | 0.00 (-0.74;0.75) | 9.94E-01 | 0.02 (-0.73;0.76) | 9.74E-01 |
| N6-Methyllysine | 0.25 (-0.57;1.07) | 6.59E-01 | -0.02 (-0.68;0.64) | 9.59E-01 | -0.01 (-0.67;0.65) | 9.76E-01 |
| Linoleate (18:2n6) | 0.08 (-0.74;0.91) | 9.00E-01 | 0.00 (-0.67;0.67) | 9.98E-01 | 0.01 (-0.67;0.69) | 9.82E-01 |
| 1-Palmitoyl-2-Palmitoleoyl-Gpc (16:0/16:1)* | -0.01 (-0.84;0.83) | 9.92E-01 | 0.01 (-0.70;0.71) | 9.90E-01 | 0.01 (-0.70;0.72) | 9.86E-01 |
| 1-(1-Enyl-Palmitoyl)-2-Oleoyl-Gpc (P-16:0/18:1)* | 0.85 (0.02;1.69) | 9.31E-02 | -0.01 (-0.73;0.71) | 9.90E-01 | 0.01 (-0.72;0.73) | 9.89E-01 |
| 6-Bromotryptophan | 0.42 (-0.40;1.24) | 4.37E-01 | -0.01 (-0.67;0.65) | 9.87E-01 | 0.00 (-0.67;0.67) | 9.92E-01 |
| Perfluorooctanesulfonate (PFOS) | 0.12 (-0.72;0.97) | 8.48E-01 | 0.01 (-0.67;0.69) | 9.87E-01 | 0.00 (-0.69;0.68) | 9.94E-01 |
| Glycocholate | -0.40 (-1.23;0.42) | 4.60E-01 | 0.00 (-0.66;0.67) | 9.93E-01 | 0.00 (-0.67;0.67) | 9.94E-01 |

Model 1 is adjusted for age, sex, and Rotterdam Study Cohort

Model 2 is additionally adjusted for body mass index, smoking status, alcohol use, serum cholesterol, lipid-lowering drugs, and prevalent cardiovascular disease

Model 3 is additionally adjusted for hypertension and diabetes

* P-values are FDR-corrected

Abbreviations: CI = confidence interval; eGFRcreat = estimated glomerular filtration rate based on serum cystatin C.

**Supplemental Table 5.** Association between serum metabolites from the Nightingale platform and urine albumin-to-creatinine ratio at baseline (n = 2,510)

|  | **Model 1** |  | **Model 2** |  | **Model 3** |  |
| --- | --- | --- | --- | --- | --- | --- |
| **Metabolite** | **Beta (95% CI)** | **P-value** | **Beta (95% CI)** | **P-value** | **Beta (95% CI)** | **P-value** |
| Cholesteryl esters to total lipids ratio in IDL | -0.17 (-0.21;-0.13) | 2.43E-13 | -0.13 (-0.17;-0.08) | 5.37E-06 | -0.12 (-0.16;-0.07) | 6.55E-05 |
| Cholesterol to total lipids ratio in IDL | -0.17 (-0.21;-0.13) | 6.87E-14 | -0.13 (-0.18;-0.08) | 7.42E-06 | -0.12 (-0.16;-0.07) | 1.15E-04 |
| Cholesteryl esters to total lipids ratio in large HDL | -0.13 (-0.17;-0.08) | 3.09E-08 | -0.11 (-0.15;-0.06) | 3.96E-05 | -0.10 (-0.14;-0.06) | 1.15E-04 |
| Cholesteryl esters in large HDL | -0.14 (-0.18;-0.10) | 5.02E-09 | -0.11 (-0.15;-0.06) | 1.62E-04 | -0.10 (-0.14;-0.05) | 8.19E-04 |
| Cholesterol to total lipids ratio in large LDL | -0.15 (-0.19;-0.11) | 5.13E-11 | -0.11 (-0.16;-0.06) | 4.99E-04 | -0.10 (-0.15;-0.05) | 1.99E-03 |
| Creatinine | 0.10 (0.05;0.15) | 1.38E-04 | 0.10 (0.05;0.15) | 1.27E-03 | 0.10 (0.05;0.15) | 3.49E-03 |
| Cholesterol to total lipids ratio in large HDL | -0.11 (-0.15;-0.07) | 1.18E-06 | -0.09 (-0.13;-0.05) | 1.19E-03 | -0.08 (-0.12;-0.04) | 3.49E-03 |
| Triglycerides to total lipids ratio in large LDL | 0.15 (0.11;0.19) | 1.90E-11 | 0.10 (0.05;0.15) | 1.13E-03 | 0.09 (0.04;0.14) | 4.58E-03 |
| Glycoprotein acetyls | 0.11 (0.07;0.15) | 1.56E-06 | 0.09 (0.05;0.13) | 1.27E-03 | 0.08 (0.04;0.13) | 6.66E-03 |
| Cholesteryl esters to total lipids ratio in medium HDL | -0.11 (-0.15;-0.07) | 2.09E-06 | -0.09 (-0.13;-0.05) | 1.27E-03 | -0.08 (-0.12;-0.04) | 6.66E-03 |
| Triglycerides to total lipids ratio in IDL | 0.16 (0.12;0.20) | 9.57E-12 | 0.10 (0.05;0.15) | 1.19E-03 | 0.09 (0.04;0.14) | 6.66E-03 |
| Triglycerides to total lipids ratio in medium LDL | 0.15 (0.11;0.19) | 7.85E-11 | 0.10 (0.05;0.14) | 1.35E-03 | 0.09 (0.04;0.13) | 8.50E-03 |
| Cholesterol in large HDL | -0.12 (-0.17;-0.08) | 2.81E-07 | -0.09 (-0.13;-0.04) | 2.17E-03 | -0.08 (-0.13;-0.03) | 8.58E-03 |
| Triglycerides in large LDL | 0.06 (0.02;0.10) | 7.95E-03 | 0.10 (0.05;0.15) | 2.00E-03 | 0.09 (0.04;0.14) | 8.58E-03 |
| Cholesterol to total lipids ratio in medium LDL | -0.12 (-0.16;-0.07) | 4.08E-07 | -0.09 (-0.13;-0.04) | 3.04E-03 | -0.08 (-0.13;-0.04) | 8.58E-03 |
| Cholesteryl esters to total lipids ratio in very small VLDL | -0.15 (-0.20;-0.11) | 2.08E-11 | -0.10 (-0.15;-0.05) | 1.27E-03 | -0.09 (-0.13;-0.04) | 8.58E-03 |
| Phospholipids in very small VLDL | 0.01 (-0.03;0.05) | 7.56E-01 | 0.10 (0.04;0.15) | 4.32E-03 | 0.10 (0.04;0.15) | 9.30E-03 |
| Triglycerides in IDL | 0.06 (0.02;0.11) | 5.27E-03 | 0.09 (0.04;0.13) | 3.04E-03 | 0.08 (0.03;0.13) | 9.43E-03 |
| Free cholesterol to total lipids ratio in large HDL | -0.11 (-0.15;-0.07) | 2.76E-06 | -0.08 (-0.12;-0.04) | 2.71E-03 | -0.07 (-0.11;-0.03) | 9.43E-03 |
| Cholesterol to total lipids ratio in medium HDL | -0.11 (-0.16;-0.07) | 7.66E-07 | -0.08 (-0.13;-0.04) | 2.55E-03 | -0.07 (-0.12;-0.03) | 9.43E-03 |
| Free cholesterol in very small VLDL | -0.01 (-0.05;0.03) | 6.78E-01 | 0.10 (0.04;0.17) | 6.17E-03 | 0.10 (0.04;0.16) | 9.43E-03 |
| Concentration of very small VLDL particles | -0.01 (-0.05;0.03) | 6.99E-01 | 0.11 (0.04;0.17) | 6.17E-03 | 0.10 (0.04;0.16) | 9.43E-03 |
| Phospholipids to total lipids ratio in very small VLDL | 0.10 (0.06;0.14) | 6.78E-06 | 0.08 (0.04;0.12) | 3.97E-03 | 0.07 (0.03;0.12) | 9.43E-03 |
| Total lipids in very small VLDL | -0.01 (-0.05;0.04) | 8.23E-01 | 0.10 (0.04;0.16) | 6.78E-03 | 0.10 (0.04;0.16) | 1.17E-02 |
| Triglycerides in LDL | 0.06 (0.02;0.10) | 5.83E-03 | 0.09 (0.04;0.14) | 3.31E-03 | 0.08 (0.03;0.13) | 1.41E-02 |
| Cholesteryl esters in small HDL | -0.09 (-0.14;-0.05) | 6.50E-05 | -0.07 (-0.12;-0.03) | 1.23E-02 | -0.08 (-0.12;-0.03) | 1.41E-02 |
| Free cholesterol in large HDL | -0.12 (-0.16;-0.08) | 5.61E-07 | -0.08 (-0.13;-0.04) | 3.97E-03 | -0.07 (-0.12;-0.03) | 1.65E-02 |
| Cholesterol to total lipids ratio in small LDL | -0.13 (-0.17;-0.09) | 1.10E-08 | -0.08 (-0.13;-0.04) | 6.17E-03 | -0.08 (-0.13;-0.03) | 1.66E-02 |
| Free cholesterol to total lipids ratio in large LDL | -0.14 (-0.18;-0.10) | 3.60E-09 | -0.09 (-0.13;-0.04) | 2.76E-03 | -0.07 (-0.12;-0.03) | 1.77E-02 |
| Cholesteryl esters in medium HDL | -0.11 (-0.15;-0.06) | 1.81E-05 | -0.08 (-0.13;-0.03) | 8.89E-03 | -0.07 (-0.12;-0.03) | 2.10E-02 |
| Triglycerides to total lipids ratio in large HDL | 0.10 (0.06;0.14) | 8.11E-06 | 0.07 (0.03;0.12) | 6.17E-03 | 0.06 (0.02;0.11) | 2.42E-02 |
| Free cholesterol to total lipids ratio in small HDL | 0 (-0.05;0.04) | 8.96E-01 | 0.08 (0.02;0.13) | 2.55E-02 | 0.08 (0.03;0.13) | 2.66E-02 |
| Cholesterol to total lipids ratio in very small VLDL | -0.15 (-0.19;-0.11) | 8.72E-11 | -0.09 (-0.14;-0.04) | 4.32E-03 | -0.07 (-0.12;-0.02) | 2.82E-02 |
| Triglycerides to total lipids ratio in large VLDL | -0.02 (-0.06;0.02) | 4.01E-01 | -0.06 (-0.10;-0.02) | 4.00E-02 | -0.06 (-0.10;-0.02) | 2.97E-02 |
| Triglycerides in medium LDL | 0.06 (0.02;0.11) | 4.74E-03 | 0.08 (0.03;0.13) | 6.40E-03 | 0.07 (0.02;0.12) | 3.17E-02 |
| Concentration of large HDL particles | -0.12 (-0.16;-0.07) | 1.75E-06 | -0.08 (-0.13;-0.03) | 9.31E-03 | -0.07 (-0.12;-0.02) | 3.23E-02 |
| Cholesterol in medium HDL | -0.11 (-0.15;-0.06) | 3.22E-05 | -0.08 (-0.12;-0.03) | 1.66E-02 | -0.07 (-0.12;-0.02) | 3.23E-02 |
| Cholesterol in small HDL | -0.09 (-0.13;-0.04) | 2.25E-04 | -0.07 (-0.12;-0.02) | 3.88E-02 | -0.07 (-0.12;-0.02) | 3.23E-02 |
| Triglycerides in very small VLDL | 0.08 (0.04;0.12) | 6.91E-04 | 0.07 (0.03;0.12) | 1.08E-02 | 0.06 (0.02;0.11) | 3.50E-02 |
| Triglycerides in very large HDL | 0.05 (0.01;0.09) | 3.99E-02 | 0.07 (0.02;0.11) | 1.95E-02 | 0.06 (0.02;0.11) | 5.56E-02 |
| Concentration of small HDL particles | -0.08 (-0.12;-0.03) | 9.38E-04 | -0.06 (-0.11;-0.01) | 6.86E-02 | -0.07 (-0.11;-0.02) | 5.84E-02 |
| Phospholipids to total lipids ratio in medium HDL | 0.11 (0.07;0.15) | 2.76E-06 | 0.07 (0.02;0.11) | 2.40E-02 | 0.06 (0.01;0.10) | 6.54E-02 |
| Cholesteryl esters to total lipids ratio in small HDL | -0.09 (-0.13;-0.05) | 9.60E-05 | -0.06 (-0.10;-0.02) | 2.80E-02 | -0.05 (-0.10;-0.01) | 6.54E-02 |
| Concentration of HDL particles | -0.11 (-0.15;-0.06) | 4.03E-05 | -0.07 (-0.12;-0.02) | 5.58E-02 | -0.07 (-0.12;-0.01) | 7.33E-02 |
| Cholesteryl esters in small VLDL | 0 (-0.04;0.04) | 9.31E-01 | 0.08 (0.02;0.13) | 4.25E-02 | 0.07 (0.01;0.12) | 7.76E-02 |
| Total concentration of lipoprotein particles | -0.11 (-0.16;-0.06) | 1.52E-05 | -0.07 (-0.13;-0.02) | 6.05E-02 | -0.07 (-0.13;-0.01) | 7.91E-02 |
| Cholesteryl esters in HDL | -0.11 (-0.16;-0.07) | 9.60E-06 | -0.07 (-0.12;-0.02) | 3.88E-02 | -0.06 (-0.11;-0.01) | 8.28E-02 |
| Cholesterol to total lipids ratio in large VLDL | -0.01 (-0.05;0.03) | 6.50E-01 | 0.04 (0.00;0.09) | 1.38E-01 | 0.05 (0.01;0.10) | 8.28E-02 |
| Cholesteryl esters to total lipids ratio in large VLDL | -0.03 (-0.07;0.02) | 3.12E-01 | 0.04 (0.00;0.09) | 1.79E-01 | 0.05 (0.01;0.10) | 9.10E-02 |
| Cholesterol in small VLDL | -0.01 (-0.05;0.03) | 6.65E-01 | 0.08 (0.02;0.14) | 5.58E-02 | 0.07 (0.01;0.13) | 9.61E-02 |
| Concentration of medium HDL particles | -0.09 (-0.14;-0.05) | 3.14E-04 | -0.06 (-0.11;-0.01) | 6.86E-02 | -0.06 (-0.11;-0.01) | 1.13E-01 |
| Total lipids in medium HDL | -0.09 (-0.13;-0.04) | 8.42E-04 | -0.06 (-0.11;-0.01) | 7.73E-02 | -0.06 (-0.10;-0.01) | 1.24E-01 |
| HDL cholesterol | -0.11 (-0.16;-0.06) | 1.67E-05 | -0.07 (-0.12;-0.01) | 6.05E-02 | -0.06 (-0.11;-0.01) | 1.29E-01 |
| Phospholipids in large HDL | -0.09 (-0.13;-0.04) | 2.87E-04 | -0.06 (-0.10;-0.01) | 5.84E-02 | -0.05 (-0.10;-0.01) | 1.30E-01 |
| Free cholesterol in medium HDL | -0.10 (-0.15;-0.05) | 1.43E-04 | -0.06 (-0.11;-0.01) | 7.84E-02 | -0.06 (-0.11;-0.01) | 1.38E-01 |
| 3-Hydroxybutyrate | 0.06 (0.01;0.10) | 2.02E-02 | 0.05 (0.01;0.10) | 6.76E-02 | 0.05 (0.00;0.09) | 1.40E-01 |
| Total lipids in small HDL | -0.06 (-0.10;-0.01) | 1.90E-02 | -0.05 (-0.10;0.00) | 1.55E-01 | -0.05 (-0.10;0.00) | 1.40E-01 |
| Phospholipids in small HDL | -0.05 (-0.10;-0.01) | 2.74E-02 | -0.05 (-0.09;0.00) | 1.51E-01 | -0.05 (-0.10;0.00) | 1.40E-01 |
| Cholesteryl esters to total lipids ratio in very large HDL | -0.06 (-0.10;-0.02) | 1.37E-02 | -0.05 (-0.09;-0.01) | 8.54E-02 | -0.04 (-0.09;0.00) | 1.49E-01 |
| Free cholesterol in large LDL | -0.12 (-0.16;-0.08) | 3.17E-07 | -0.11 (-0.20;-0.02) | 6.19E-02 | -0.10 (-0.18;-0.01) | 1.51E-01 |
| Acetone | 0.04 (0.00;0.08) | 8.75E-02 | 0.05 (0.00;0.09) | 1.00E-01 | 0.04 (0.00;0.08) | 1.56E-01 |
| Apolipoprotein A1 | -0.09 (-0.14;-0.05) | 2.86E-04 | -0.06 (-0.11;-0.01) | 1.02E-01 | -0.05 (-0.10;0.00) | 1.64E-01 |
| Cholesteryl esters in IDL | -0.13 (-0.17;-0.08) | 1.55E-07 | -0.11 (-0.20;-0.03) | 5.84E-02 | -0.09 (-0.18;0.00) | 1.64E-01 |
| Phospholipids in medium HDL | -0.07 (-0.12;-0.03) | 4.30E-03 | -0.05 (-0.10;0.00) | 1.10E-01 | -0.05 (-0.10;0.00) | 1.64E-01 |
| Triglycerides to total lipids ratio in medium HDL | 0.08 (0.04;0.12) | 6.21E-04 | 0.05 (0.01;0.09) | 7.73E-02 | 0.04 (0.00;0.09) | 1.64E-01 |
| Total esterified cholesterol | -0.11 (-0.16;-0.07) | 1.75E-06 | -0.13 (-0.25;-0.02) | 8.21E-02 | -0.12 (-0.23;-0.01) | 1.64E-01 |
| Cholesterol in very small VLDL | -0.06 (-0.10;-0.02) | 1.11E-02 | 0.06 (-0.01;0.13) | 1.94E-01 | 0.07 (0.00;0.14) | 1.64E-01 |
| Total lipids in large HDL | -0.10 (-0.15;-0.06) | 3.95E-05 | -0.06 (-0.11;-0.01) | 6.86E-02 | -0.05 (-0.10;0.00) | 1.64E-01 |
| Phospholipids in small VLDL | 0 (-0.04;0.04) | 8.80E-01 | 0.07 (0.01;0.13) | 7.81E-02 | 0.06 (0.00;0.12) | 1.65E-01 |
| Ratio of apolipoprotein B to apolipoprotein A1 | 0 (-0.04;0.04) | 9.27E-01 | 0.06 (0.00;0.11) | 1.06E-01 | 0.05 (0.00;0.10) | 1.69E-01 |
| Glutamine | 0.02 (-0.02;0.06) | 3.65E-01 | 0.04 (0.00;0.08) | 1.96E-01 | 0.04 (0.00;0.08) | 1.69E-01 |
| Free cholesterol in small VLDL | -0.03 (-0.07;0.01) | 2.80E-01 | 0.07 (0.01;0.14) | 9.43E-02 | 0.07 (0.00;0.13) | 1.69E-01 |
| Cholesteryl esters in VLDL | -0.03 (-0.07;0.01) | 2.81E-01 | 0.07 (0.01;0.13) | 1.06E-01 | 0.06 (0.00;0.12) | 1.75E-01 |
| Triglycerides to total lipids ratio in small HDL | 0.09 (0.05;0.13) | 1.42E-04 | 0.05 (0.01;0.10) | 7.84E-02 | 0.04 (0.00;0.09) | 1.76E-01 |
| Citrate | 0.04 (0.00;0.08) | 1.09E-01 | 0.05 (0.01;0.09) | 8.21E-02 | 0.04 (0.00;0.08) | 1.78E-01 |
| Triglycerides to total lipids ratio in very large HDL | 0.09 (0.05;0.14) | 5.17E-05 | 0.06 (0.01;0.10) | 6.28E-02 | 0.04 (0.00;0.09) | 1.78E-01 |
| Phospholipids to total lipids ratio in medium VLDL | -0.05 (-0.10;-0.01) | 2.18E-02 | 0.04 (-0.01;0.09) | 3.17E-01 | 0.05 (0.00;0.10) | 1.79E-01 |
| Triglycerides to total lipids ratio in small LDL | 0.11 (0.07;0.16) | 7.05E-07 | 0.06 (0.01;0.10) | 6.46E-02 | 0.04 (0.00;0.09) | 1.85E-01 |
| Concentration of small VLDL particles | 0.03 (-0.01;0.07) | 2.01E-01 | 0.06 (0.01;0.11) | 8.21E-02 | 0.05 (0.00;0.09) | 1.91E-01 |
| Cholesteryl esters to total lipids ratio in large LDL | -0.05 (-0.09;-0.01) | 2.61E-02 | -0.03 (-0.08;0.01) | 2.78E-01 | -0.04 (-0.09;0.00) | 2.08E-01 |
| Free cholesterol to total lipids ratio in large VLDL | 0.02 (-0.02;0.06) | 4.63E-01 | 0.04 (0.00;0.08) | 1.93E-01 | 0.04 (0.00;0.08) | 2.08E-01 |
| Total lipids in HDL | -0.09 (-0.14;-0.05) | 3.06E-04 | -0.05 (-0.10;0.00) | 1.36E-01 | -0.05 (-0.10;0.00) | 2.12E-01 |
| Total lipids in small VLDL | 0.02 (-0.02;0.06) | 3.81E-01 | 0.06 (0.01;0.11) | 9.25E-02 | 0.05 (0.00;0.10) | 2.12E-01 |
| Concentration of VLDL particles | 0.01 (-0.03;0.05) | 6.64E-01 | 0.06 (0.01;0.11) | 9.43E-02 | 0.05 (0.00;0.10) | 2.12E-01 |
| Cholesterol to total lipids ratio in small HDL | -0.09 (-0.13;-0.05) | 1.42E-04 | -0.05 (-0.09;0.00) | 1.12E-01 | -0.04 (-0.08;0.00) | 2.18E-01 |
| Phospholipids in HDL | -0.09 (-0.13;-0.04) | 9.65E-04 | -0.05 (-0.10;0.00) | 1.51E-01 | -0.05 (-0.10;0.01) | 2.23E-01 |
| Triglycerides in small LDL | 0.06 (0.01;0.10) | 1.48E-02 | 0.05 (0.01;0.10) | 8.21E-02 | 0.04 (-0.01;0.09) | 2.32E-01 |
| Cholesterol in IDL | -0.12 (-0.17;-0.08) | 2.66E-07 | -0.10 (-0.19;-0.01) | 9.18E-02 | -0.08 (-0.17;0.01) | 2.43E-01 |
| Triglycerides in large HDL | 0.03 (-0.02;0.07) | 2.81E-01 | 0.04 (0.00;0.08) | 1.89E-01 | 0.04 (-0.01;0.08) | 2.52E-01 |
| Ratio of omega-6 fatty acids to total fatty acids | -0.09 (-0.13;-0.05) | 1.17E-04 | -0.05 (-0.10;-0.01) | 9.25E-02 | -0.04 (-0.08;0.01) | 2.58E-01 |
| Free cholesterol in LDL | -0.11 (-0.15;-0.07) | 2.61E-06 | -0.09 (-0.17;0.00) | 1.51E-01 | -0.07 (-0.16;0.01) | 2.62E-01 |
| Valine | 0 (-0.04;0.05) | 9.31E-01 | -0.03 (-0.07;0.02) | 4.51E-01 | -0.04 (-0.09;0.01) | 2.62E-01 |
| VLDL cholesterol | -0.01 (-0.05;0.03) | 6.55E-01 | 0.06 (0.00;0.11) | 1.44E-01 | 0.05 (-0.01;0.10) | 2.62E-01 |
| Free cholesterol to total lipids ratio in medium LDL | -0.09 (-0.14;-0.05) | 3.84E-05 | -0.05 (-0.09;0.00) | 1.06E-01 | -0.04 (-0.08;0.01) | 2.68E-01 |
| Phospholipids to total lipids ratio in IDL | 0.04 (0.00;0.08) | 7.99E-02 | 0.04 (-0.01;0.08) | 2.08E-01 | 0.03 (-0.01;0.07) | 2.79E-01 |
| Free cholesterol to total lipids ratio in medium VLDL | -0.06 (-0.11;-0.02) | 6.96E-03 | 0.03 (-0.02;0.08) | 4.87E-01 | 0.04 (-0.01;0.09) | 2.84E-01 |
| Ratio of polyunsaturated fatty acids to monounsaturated fatty acids | -0.09 (-0.13;-0.05) | 1.42E-04 | -0.05 (-0.09;0.00) | 1.34E-01 | -0.04 (-0.08;0.01) | 2.86E-01 |
| Free cholesterol to total lipids ratio in small LDL | -0.08 (-0.12;-0.04) | 4.22E-04 | -0.04 (-0.09;0.00) | 1.34E-01 | -0.03 (-0.08;0.01) | 2.86E-01 |
| Cholesterol to total lipids ratio in very large VLDL | -0.04 (-0.08;0.00) | 9.37E-02 | 0.02 (-0.02;0.07) | 4.98E-01 | 0.04 (-0.01;0.08) | 3.05E-01 |
| Triglycerides to total lipids ratio in very large VLDL | 0.02 (-0.02;0.06) | 4.49E-01 | -0.03 (-0.07;0.02) | 4.00E-01 | -0.03 (-0.08;0.01) | 3.11E-01 |
| Alanine | 0 (-0.04;0.04) | 9.06E-01 | -0.02 (-0.06;0.02) | 5.78E-01 | -0.03 (-0.07;0.01) | 3.12E-01 |
| Triglycerides in HDL | 0.05 (0.01;0.10) | 1.90E-02 | 0.04 (0.00;0.08) | 1.67E-01 | 0.03 (-0.01;0.08) | 3.12E-01 |
| Leucine | 0.01 (-0.04;0.05) | 8.21E-01 | -0.02 (-0.07;0.03) | 5.48E-01 | -0.04 (-0.09;0.01) | 3.12E-01 |
| Free cholesterol to total lipids ratio in medium HDL | -0.11 (-0.15;-0.06) | 1.81E-05 | -0.05 (-0.11;0.00) | 1.83E-01 | -0.04 (-0.10;0.01) | 3.12E-01 |
| Ratio of polyunsaturated fatty acids to total fatty acids | -0.08 (-0.12;-0.04) | 5.99E-04 | -0.04 (-0.09;0.00) | 1.49E-01 | -0.03 (-0.08;0.01) | 3.12E-01 |
| Phospholipids to total lipids ratio in very large HDL | -0.07 (-0.11;-0.03) | 2.34E-03 | -0.04 (-0.08;0.00) | 1.55E-01 | -0.03 (-0.07;0.01) | 3.12E-01 |
| Ratio of monounsaturated fatty acids to total fatty acids | 0.09 (0.05;0.13) | 1.42E-04 | 0.04 (0.00;0.09) | 1.61E-01 | 0.03 (-0.01;0.08) | 3.14E-01 |
| Apolipoprotein B | -0.05 (-0.09;-0.01) | 2.17E-02 | 0.07 (-0.01;0.15) | 2.35E-01 | 0.06 (-0.02;0.14) | 3.17E-01 |
| Total cholesterol | -0.11 (-0.15;-0.06) | 8.11E-06 | -0.10 (-0.22;0.02) | 2.27E-01 | -0.09 (-0.21;0.03) | 3.21E-01 |
| Free cholesterol in HDL | -0.10 (-0.15;-0.05) | 1.39E-04 | -0.05 (-0.10;0.01) | 2.09E-01 | -0.04 (-0.09;0.01) | 3.28E-01 |
| Histidine | -0.04 (-0.08;0.00) | 7.87E-02 | -0.03 (-0.07;0.01) | 2.76E-01 | -0.03 (-0.07;0.01) | 3.40E-01 |
| Glycine | -0.05 (-0.09;-0.01) | 3.68E-02 | -0.04 (-0.09;0.00) | 1.79E-01 | -0.03 (-0.08;0.01) | 3.69E-01 |
| Total concentration of branched-chain amino acids (leucine + isoleucine + valine) | 0.01 (-0.03;0.06) | 6.50E-01 | -0.02 (-0.07;0.03) | 6.26E-01 | -0.03 (-0.08;0.02) | 3.76E-01 |
| Cholesteryl esters to total lipids ratio in very large VLDL | -0.04 (-0.09;0.00) | 6.68E-02 | 0.02 (-0.03;0.06) | 6.13E-01 | 0.03 (-0.01;0.08) | 3.80E-01 |
| Triglycerides to total lipids ratio in medium VLDL | 0.06 (0.02;0.10) | 9.82E-03 | -0.02 (-0.07;0.03) | 6.13E-01 | -0.03 (-0.08;0.02) | 3.91E-01 |
| Cholesterol in large LDL | -0.10 (-0.14;-0.06) | 1.81E-05 | -0.07 (-0.16;0.03) | 3.19E-01 | -0.06 (-0.16;0.03) | 4.16E-01 |
| Cholesteryl esters in very large HDL | -0.10 (-0.14;-0.05) | 7.17E-05 | -0.04 (-0.09;0.00) | 1.92E-01 | -0.03 (-0.08;0.02) | 4.20E-01 |
| Triglycerides to total lipids ratio in very small VLDL | 0.12 (0.08;0.16) | 2.66E-07 | 0.05 (0.00;0.10) | 1.79E-01 | 0.03 (-0.02;0.08) | 4.29E-01 |
| Concentration of LDL particles | -0.05 (-0.10;-0.01) | 1.72E-02 | 0.06 (-0.02;0.14) | 3.15E-01 | 0.05 (-0.03;0.13) | 4.37E-01 |
| Ratio of linoleic acid to total fatty acids | -0.10 (-0.14;-0.06) | 9.08E-06 | -0.04 (-0.09;0.00) | 1.93E-01 | -0.03 (-0.08;0.02) | 4.39E-01 |
| Free cholesterol in VLDL | 0.00 (-0.04;0.05) | 8.68E-01 | 0.04 (-0.01;0.10) | 2.22E-01 | 0.03 (-0.02;0.08) | 4.39E-01 |
| Phospholipids in VLDL | 0.01 (-0.03;0.06) | 5.91E-01 | 0.04 (-0.01;0.09) | 2.13E-01 | 0.03 (-0.02;0.08) | 4.39E-01 |
| Phospholipids to total lipids ratio in large HDL | -0.01 (-0.05;0.03) | 7.28E-01 | -0.02 (-0.07;0.02) | 4.16E-01 | -0.03 (-0.07;0.02) | 4.42E-01 |
| Acetoacetate | 0.04 (0.00;0.08) | 9.04E-02 | 0.03 (-0.01;0.07) | 3.07E-01 | 0.02 (-0.02;0.07) | 4.45E-01 |
| Monounsaturated fatty acids | 0.03 (-0.01;0.07) | 1.70E-01 | 0.04 (-0.01;0.09) | 1.98E-01 | 0.03 (-0.02;0.08) | 4.45E-01 |
| Phospholipids in very large HDL | -0.08 (-0.12;-0.04) | 6.09E-04 | -0.04 (-0.08;0.01) | 2.13E-01 | -0.03 (-0.07;0.02) | 4.45E-01 |
| Phosphatidylcholines | -0.08 (-0.12;-0.03) | 2.29E-03 | -0.03 (-0.10;0.03) | 4.98E-01 | -0.04 (-0.10;0.03) | 4.46E-01 |
| Free cholesterol in small HDL | -0.06 (-0.10;-0.01) | 2.17E-02 | -0.03 (-0.08;0.03) | 5.41E-01 | -0.03 (-0.09;0.02) | 4.56E-01 |
| Cholesterol in medium VLDL | -0.06 (-0.10;-0.02) | 5.90E-03 | 0.04 (-0.03;0.12) | 4.67E-01 | 0.04 (-0.03;0.12) | 4.71E-01 |
| Triglycerides in small HDL | 0.06 (0.02;0.10) | 8.51E-03 | 0.04 (-0.01;0.08) | 2.53E-01 | 0.03 (-0.02;0.07) | 4.71E-01 |
| Tyrosine | 0.00 (-0.04;0.04) | 9.31E-01 | -0.02 (-0.06;0.03) | 6.26E-01 | -0.02 (-0.07;0.02) | 4.71E-01 |
| Average diameter for VLDL particles | 0.02 (-0.02;0.07) | 3.49E-01 | -0.01 (-0.06;0.03) | 7.28E-01 | -0.03 (-0.07;0.02) | 4.71E-01 |
| Concentration of medium LDL particles | -0.03 (-0.07;0.01) | 1.97E-01 | 0.05 (-0.02;0.11) | 3.00E-01 | 0.04 (-0.03;0.10) | 4.76E-01 |
| Free cholesterol in medium LDL | -0.09 (-0.13;-0.05) | 5.63E-05 | -0.05 (-0.13;0.03) | 4.20E-01 | -0.04 (-0.12;0.03) | 4.77E-01 |
| Concentration of large LDL particles | -0.07 (-0.11;-0.02) | 4.16E-03 | 0.05 (-0.03;0.13) | 4.51E-01 | 0.05 (-0.04;0.13) | 4.78E-01 |
| Remnant cholesterol (non-HDL. non-LDL -cholesterol) | -0.06 (-0.11;-0.02) | 5.12E-03 | 0.05 (-0.04;0.14) | 4.48E-01 | 0.05 (-0.04;0.13) | 4.78E-01 |
| Concentration of small LDL particles | -0.04 (-0.08;0.00) | 6.94E-02 | 0.05 (-0.02;0.12) | 3.19E-01 | 0.04 (-0.03;0.11) | 4.78E-01 |
| Clinical LDL cholesterol | -0.10 (-0.14;-0.06) | 2.20E-05 | -0.06 (-0.15;0.03) | 3.84E-01 | -0.05 (-0.14;0.04) | 4.81E-01 |
| Free cholesterol to total lipids ratio in very large HDL | 0.08 (0.03;0.12) | 1.34E-03 | 0.04 (-0.01;0.08) | 3.00E-01 | 0.03 (-0.02;0.07) | 4.81E-01 |
| Free cholesterol to total lipids ratio in very small VLDL | -0.05 (-0.09;-0.01) | 2.82E-02 | 0.02 (-0.03;0.07) | 5.98E-01 | 0.03 (-0.02;0.08) | 4.81E-01 |
| Free cholesterol in medium VLDL | -0.03 (-0.07;0.01) | 2.08E-01 | 0.04 (-0.02;0.10) | 3.42E-01 | 0.03 (-0.03;0.09) | 4.88E-01 |
| Triglycerides in medium HDL | 0.04 (0.00;0.09) | 5.91E-02 | 0.03 (-0.01;0.07) | 3.57E-01 | 0.02 (-0.02;0.06) | 5.17E-01 |
| Triglycerides in small VLDL | 0.05 (0.01;0.09) | 2.66E-02 | 0.03 (-0.01;0.08) | 2.81E-01 | 0.02 (-0.02;0.07) | 5.27E-01 |
| Cholesteryl esters to total lipids ratio in small VLDL | -0.06 (-0.10;-0.02) | 1.37E-02 | 0.01 (-0.03;0.06) | 7.70E-01 | 0.02 (-0.02;0.07) | 5.30E-01 |
| Cholesteryl esters in large LDL | -0.09 (-0.13;-0.05) | 7.20E-05 | -0.05 (-0.14;0.05) | 5.08E-01 | -0.05 (-0.14;0.05) | 5.36E-01 |
| Saturated fatty acids | 0.00 (-0.04;0.05) | 8.80E-01 | 0.04 (-0.01;0.09) | 3.00E-01 | 0.03 (-0.03;0.08) | 6.02E-01 |
| Total fatty acids | 0.00 (-0.04;0.04) | 9.78E-01 | 0.04 (-0.01;0.10) | 3.03E-01 | 0.02 (-0.03;0.08) | 6.25E-01 |
| Phospholipids in medium VLDL | -0.02 (-0.06;0.02) | 3.90E-01 | 0.04 (-0.02;0.09) | 4.16E-01 | 0.03 (-0.03;0.08) | 6.26E-01 |
| Free cholesterol to total lipids ratio in small VLDL | -0.11 (-0.15;-0.07) | 2.09E-06 | -0.04 (-0.09;0.01) | 3.03E-01 | -0.02 (-0.07;0.03) | 6.32E-01 |
| Phospholipids in large LDL | -0.10 (-0.14;-0.05) | 2.99E-05 | -0.05 (-0.14;0.05) | 4.94E-01 | -0.04 (-0.14;0.05) | 6.39E-01 |
| Cholesteryl esters to total lipids ratio in small LDL | -0.03 (-0.08;0.01) | 1.41E-01 | -0.01 (-0.06;0.03) | 7.02E-01 | -0.02 (-0.06;0.02) | 6.45E-01 |
| LDL cholesterol | -0.09 (-0.13;-0.04) | 1.71E-04 | -0.04 (-0.13;0.05) | 6.16E-01 | -0.04 (-0.13;0.05) | 6.48E-01 |
| Free cholesterol to total lipids ratio in IDL | -0.08 (-0.12;-0.03) | 8.25E-04 | -0.03 (-0.07;0.02) | 4.16E-01 | -0.02 (-0.06;0.03) | 6.54E-01 |
| Cholesterol in very large HDL | -0.09 (-0.13;-0.05) | 1.53E-04 | -0.03 (-0.08;0.02) | 3.78E-01 | -0.02 (-0.07;0.03) | 6.54E-01 |
| Total cholines | -0.08 (-0.13;-0.04) | 1.09E-03 | -0.02 (-0.10;0.05) | 6.87E-01 | -0.03 (-0.10;0.04) | 6.64E-01 |
| Phospholipids to total lipids ratio in medium LDL | -0.05 (-0.09;-0.01) | 2.45E-02 | -0.02 (-0.07;0.02) | 4.44E-01 | -0.02 (-0.06;0.03) | 6.64E-01 |
| Degree of unsaturation | -0.05 (-0.09;-0.01) | 3.33E-02 | -0.02 (-0.06;0.02) | 5.45E-01 | -0.02 (-0.06;0.03) | 6.78E-01 |
| Free cholesterol in IDL | -0.11 (-0.16;-0.07) | 1.56E-06 | -0.05 (-0.14;0.03) | 3.83E-01 | -0.03 (-0.12;0.05) | 6.81E-01 |
| Concentration of IDL particles | -0.08 (-0.12;-0.04) | 5.57E-04 | 0.02 (-0.06;0.10) | 7.95E-01 | 0.03 (-0.05;0.11) | 6.81E-01 |
| Concentration of medium VLDL particles | -0.02 (-0.06;0.02) | 5.01E-01 | 0.03 (-0.02;0.09) | 4.56E-01 | 0.02 (-0.03;0.08) | 6.81E-01 |
| Triglycerides in large VLDL | 0.01 (-0.03;0.05) | 6.52E-01 | -0.01 (-0.05;0.04) | 8.87E-01 | -0.02 (-0.06;0.03) | 6.98E-01 |
| Cholesterol to total lipids ratio in very large HDL | 0.01 (-0.03;0.05) | 6.59E-01 | -0.01 (-0.06;0.03) | 7.60E-01 | -0.02 (-0.06;0.03) | 6.98E-01 |
| Cholesteryl esters in large VLDL | 0.01 (-0.03;0.05) | 7.38E-01 | 0.03 (-0.02;0.07) | 4.87E-01 | 0.02 (-0.03;0.06) | 7.15E-01 |
| Phospholipids in small LDL | -0.05 (-0.09;-0.01) | 1.82E-02 | 0.03 (-0.04;0.11) | 5.91E-01 | 0.03 (-0.05;0.10) | 7.15E-01 |
| Total phospholipids in lipoprotein particles | -0.08 (-0.13;-0.04) | 7.12E-04 | -0.02 (-0.11;0.06) | 7.52E-01 | -0.03 (-0.11;0.05) | 7.15E-01 |
| Cholesteryl esters in very large VLDL | 0.01 (-0.04;0.05) | 8.39E-01 | 0.03 (-0.02;0.07) | 4.87E-01 | 0.02 (-0.03;0.06) | 7.15E-01 |
| Cholesteryl esters in very small VLDL | -0.08 (-0.13;-0.04) | 2.88E-04 | 0.01 (-0.06;0.08) | 8.44E-01 | 0.02 (-0.04;0.09) | 7.15E-01 |
| Total lipids in large LDL | -0.09 (-0.13;-0.05) | 7.65E-05 | -0.04 (-0.13;0.06) | 6.30E-01 | -0.03 (-0.13;0.06) | 7.22E-01 |
| Phospholipids to total lipids ratio in large LDL | -0.01 (-0.05;0.03) | 6.71E-01 | -0.02 (-0.07;0.02) | 4.87E-01 | -0.01 (-0.06;0.03) | 7.29E-01 |
| Phosphoglycerides | -0.07 (-0.11;-0.02) | 8.96E-03 | -0.01 (-0.08;0.05) | 8.12E-01 | -0.02 (-0.09;0.04) | 7.29E-01 |
| Free cholesterol in small LDL | -0.08 (-0.12;-0.04) | 3.13E-04 | -0.03 (-0.10;0.05) | 6.50E-01 | -0.02 (-0.10;0.05) | 7.29E-01 |
| Glucose | 0.09 (0.05;0.13) | 1.11E-04 | 0.05 (0.01;0.10) | 9.18E-02 | 0.02 (-0.04;0.07) | 7.47E-01 |
| Total lipids in VLDL | 0.02 (-0.02;0.06) | 4.86E-01 | 0.03 (-0.02;0.07) | 4.51E-01 | 0.02 (-0.03;0.06) | 7.47E-01 |
| Phenylalanine | 0.01 (-0.04;0.05) | 7.90E-01 | -0.01 (-0.05;0.03) | 7.78E-01 | -0.01 (-0.06;0.03) | 7.49E-01 |
| Ratio of triglycerides to phosphoglycerides | 0.07 (0.02;0.11) | 5.83E-03 | 0.03 (-0.02;0.07) | 4.51E-01 | 0.01 (-0.03;0.06) | 7.49E-01 |
| Phospholipids to total lipids ratio in small HDL | 0.01 (-0.03;0.05) | 6.55E-01 | -0.02 (-0.06;0.03) | 6.87E-01 | -0.01 (-0.06;0.03) | 7.51E-01 |
| Phospholipids to total lipids ratio in small VLDL | -0.10 (-0.14;-0.06) | 1.20E-05 | -0.03 (-0.08;0.02) | 4.50E-01 | -0.02 (-0.06;0.03) | 7.52E-01 |
| Phospholipids to total lipids ratio in small LDL | 0.01 (-0.03;0.05) | 6.55E-01 | 0.00 (-0.04;0.05) | 8.92E-01 | 0.01 (-0.03;0.06) | 7.69E-01 |
| Free cholesterol to total lipids ratio in very large VLDL | -0.04 (-0.09;0.00) | 5.63E-02 | 0.01 (-0.04;0.05) | 8.65E-01 | 0.01 (-0.03;0.06) | 7.69E-01 |
| Cholesterol to total lipids ratio in medium VLDL | -0.08 (-0.12;-0.04) | 6.21E-04 | 0.00 (-0.05;0.05) | 9.59E-01 | 0.01 (-0.04;0.06) | 7.81E-01 |
| Triglycerides in chylomicrons and extremely large VLDL | 0.02 (-0.03;0.06) | 5.61E-01 | 0.00 (-0.05;0.04) | 8.92E-01 | -0.01 (-0.05;0.03) | 7.81E-01 |
| Cholesteryl esters in LDL | -0.08 (-0.12;-0.04) | 7.25E-04 | -0.02 (-0.10;0.07) | 8.16E-01 | -0.02 (-0.11;0.06) | 7.88E-01 |
| Cholesteryl esters to total lipids ratio in medium VLDL | -0.09 (-0.13;-0.05) | 1.11E-04 | -0.03 (-0.07;0.02) | 4.77E-01 | -0.01 (-0.06;0.03) | 7.88E-01 |
| Total lipids in IDL | -0.11 (-0.15;-0.06) | 8.04E-06 | -0.05 (-0.14;0.05) | 5.23E-01 | -0.02 (-0.12;0.07) | 7.97E-01 |
| Cholesteryl esters in chylomicrons and extremely large VLDL | 0.04 (-0.01;0.08) | 1.28E-01 | 0.02 (-0.02;0.07) | 5.12E-01 | 0.01 (-0.03;0.06) | 7.97E-01 |
| Acetate | -0.03 (-0.07;0.01) | 2.01E-01 | -0.01 (-0.05;0.03) | 7.19E-01 | -0.01 (-0.05;0.03) | 8.00E-01 |
| Average diameter for HDL particles | -0.06 (-0.10;-0.01) | 2.17E-02 | -0.02 (-0.07;0.03) | 5.90E-01 | -0.01 (-0.06;0.04) | 8.00E-01 |
| Total lipids in small LDL | -0.05 (-0.09;-0.01) | 2.13E-02 | 0.03 (-0.05;0.11) | 6.27E-01 | 0.02 (-0.06;0.10) | 8.00E-01 |
| Triglycerides to total lipids ratio in small VLDL | 0.08 (0.04;0.12) | 7.49E-04 | 0.00 (-0.05;0.05) | 9.96E-01 | -0.01 (-0.06;0.04) | 8.00E-01 |
| Albumin | -0.03 (-0.07;0.01) | 2.57E-01 | -0.01 (-0.05;0.03) | 8.16E-01 | -0.01 (-0.05;0.03) | 8.02E-01 |
| Ratio of omega-3 fatty acids to total fatty acids | 0.02 (-0.02;0.06) | 5.08E-01 | 0.00 (-0.05;0.04) | 9.15E-01 | -0.01 (-0.05;0.03) | 8.02E-01 |
| Total lipids in very large HDL | -0.08 (-0.12;-0.04) | 9.17E-04 | -0.02 (-0.07;0.03) | 5.48E-01 | -0.01 (-0.06;0.04) | 8.02E-01 |
| Cholesterol in chylomicrons and extremely large VLDL | 0.04 (0.00;0.08) | 7.87E-02 | 0.02 (-0.02;0.07) | 5.22E-01 | 0.01 (-0.03;0.05) | 8.02E-01 |
| Cholesterol in very large VLDL | 0.01 (-0.03;0.05) | 6.05E-01 | 0.02 (-0.03;0.07) | 5.78E-01 | 0.01 (-0.04;0.06) | 8.09E-01 |
| Triglycerides in very large VLDL | 0.03 (-0.02;0.07) | 3.09E-01 | 0.00 (-0.04;0.04) | 9.96E-01 | -0.01 (-0.05;0.03) | 8.11E-01 |
| Total triglycerides | 0.04 (0.00;0.08) | 1.09E-01 | 0.02 (-0.02;0.07) | 5.12E-01 | 0.01 (-0.04;0.06) | 8.24E-01 |
| Ratio of docosahexaenoic acid to total fatty acids | 0.00 (-0.04;0.04) | 9.06E-01 | -0.01 (-0.05;0.03) | 8.12E-01 | -0.01 (-0.05;0.03) | 8.49E-01 |
| Cholesterol in large VLDL | 0.01 (-0.03;0.06) | 5.94E-01 | 0.02 (-0.03;0.07) | 6.13E-01 | 0.01 (-0.04;0.06) | 8.62E-01 |
| Phospholipids to total lipids ratio in very large VLDL | -0.02 (-0.06;0.02) | 4.77E-01 | -0.01 (-0.05;0.03) | 7.78E-01 | -0.01 (-0.05;0.03) | 8.62E-01 |
| Ratio of saturated fatty acids to total fatty acids | 0.02 (-0.02;0.06) | 5.29E-01 | 0.01 (-0.03;0.05) | 7.49E-01 | 0.01 (-0.03;0.05) | 8.63E-01 |
| Total lipids in medium VLDL | -0.01 (-0.05;0.03) | 6.78E-01 | 0.02 (-0.03;0.07) | 6.29E-01 | 0.01 (-0.04;0.06) | 8.84E-01 |
| Cholesterol to total lipids ratio in small VLDL | -0.08 (-0.12;-0.04) | 3.90E-04 | -0.01 (-0.05;0.04) | 8.91E-01 | 0.01 (-0.04;0.06) | 8.84E-01 |
| Phospholipids in very large VLDL | 0.01 (-0.03;0.05) | 6.78E-01 | 0.00 (-0.04;0.04) | 9.96E-01 | -0.01 (-0.05;0.04) | 8.84E-01 |
| Cholesteryl esters to total lipids ratio in medium LDL | 0.02 (-0.02;0.06) | 3.57E-01 | 0.00 (-0.04;0.04) | 9.95E-01 | -0.01 (-0.05;0.04) | 8.87E-01 |
| Phospholipids in LDL | -0.08 (-0.12;-0.04) | 3.78E-04 | -0.01 (-0.10;0.08) | 8.51E-01 | -0.01 (-0.10;0.08) | 8.91E-01 |
| Free cholesterol in chylomicrons and extremely large VLDL | 0.05 (0.00;0.09) | 4.99E-02 | 0.02 (-0.03;0.06) | 6.02E-01 | 0.01 (-0.04;0.05) | 8.91E-01 |
| Cholesteryl esters in medium VLDL | -0.09 (-0.14;-0.05) | 3.84E-05 | -0.02 (-0.09;0.05) | 7.11E-01 | -0.01 (-0.08;0.06) | 9.14E-01 |
| Total lipids in large VLDL | 0.02 (-0.03;0.06) | 5.48E-01 | 0.01 (-0.04;0.05) | 8.87E-01 | -0.01 (-0.05;0.04) | 9.22E-01 |
| Omega-6 fatty acids | -0.06 (-0.10;-0.02) | 9.44E-03 | 0.00 (-0.06;0.07) | 9.59E-01 | -0.01 (-0.07;0.06) | 9.22E-01 |
| Cholesteryl esters in small LDL | -0.05 (-0.09;-0.01) | 1.90E-02 | 0.02 (-0.05;0.09) | 7.60E-01 | 0.01 (-0.06;0.08) | 9.22E-01 |
| Total lipids in LDL | -0.08 (-0.12;-0.04) | 6.91E-04 | -0.01 (-0.10;0.08) | 9.22E-01 | -0.01 (-0.10;0.08) | 9.25E-01 |
| Total free cholesterol | -0.08 (-0.13;-0.04) | 3.30E-04 | 0.02 (-0.10;0.13) | 8.65E-01 | 0.01 (-0.10;0.13) | 9.25E-01 |
| Triglycerides in medium VLDL | 0.01 (-0.03;0.05) | 6.37E-01 | 0.01 (-0.04;0.05) | 8.82E-01 | -0.01 (-0.05;0.04) | 9.28E-01 |
| Cholesterol in medium LDL | -0.06 (-0.10;-0.02) | 1.07E-02 | 0.00 (-0.07;0.07) | 9.91E-01 | -0.01 (-0.08;0.06) | 9.35E-01 |
| Total lipids in chylomicrons and extremely large VLDL | 0.03 (-0.01;0.07) | 1.63E-01 | 0.01 (-0.04;0.05) | 8.39E-01 | 0.00 (-0.05;0.04) | 9.56E-01 |
| Concentration of chylomicrons and extremely large VLDL particles | 0.04 (0.00;0.08) | 8.36E-02 | 0.02 (-0.03;0.06) | 6.83E-01 | 0.00 (-0.04;0.05) | 9.56E-01 |
| Phospholipids in large VLDL | 0.02 (-0.02;0.06) | 5.14E-01 | 0.00 (-0.04;0.05) | 8.92E-01 | 0.00 (-0.05;0.04) | 9.63E-01 |
| Total cholesterol minus HDL-C | -0.08 (-0.12;-0.04) | 6.91E-04 | 0.01 (-0.09;0.11) | 8.92E-01 | 0.01 (-0.09;0.10) | 9.80E-01 |
| Total lipids in very large VLDL | 0.02 (-0.02;0.07) | 3.47E-01 | 0.01 (-0.04;0.05) | 8.34E-01 | 0.00 (-0.05;0.04) | 9.80E-01 |
| Free cholesterol in large VLDL | 0.02 (-0.02;0.06) | 4.77E-01 | 0.01 (-0.03;0.06) | 7.62E-01 | 0.00 (-0.04;0.05) | 9.80E-01 |
| Phospholipids to total lipids ratio in large VLDL | 0.01 (-0.03;0.05) | 6.37E-01 | 0v (-0.04;0.04) | 8.92E-01 | 0.00 (-0.04;0.04) | 9.80E-01 |
| Linoleic acid | -0.06 (-0.11;-0.02) | 5.27E-03 | 0v (-0.06;0.07) | 9.30E-01 | 0.00 (-0.06;0.06) | 9.80E-01 |
| Lactate | 0.02 (-0.02;0.06) | 3.86E-01 | 0.01 (-0.03;0.05) | 7.71E-01 | 0.00 (-0.04;0.04) | 9.80E-01 |
| Average diameter for LDL particles | -0.06 (-0.10;-0.02) | 6.45E-03 | -0.01 (-0.05;0.03) | 7.71E-01 | 0.00 (-0.05;0.04) | 9.80E-01 |
| Total lipids in medium LDL | -0.05 (-0.09;-0.01) | 2.01E-02 | 0.01 (-0.06;0.09) | 8.16E-01 | 0.00 (-0.07;0.08) | 9.80E-01 |
| Phospholipids in medium LDL | -0.06 (-0.10;-0.02) | 5.95E-03 | 0.00 (-0.07;0.08) | 9.59E-01 | -0.01 (-0.08;0.07) | 9.80E-01 |
| Omega-3 fatty acids | 0.01 (-0.03;0.06) | 6.00E-01 | 0.01 (-0.04;0.06) | 8.16E-01 | 0.00 (-0.05;0.04) | 9.80E-01 |
| Ratio of omega-6 fatty acids to omega-3 fatty acids | -0.04 (-0.08;0.00) | 1.25E-01 | -0.01 (-0.05;0.04) | 8.34E-01 | 0.00 (-0.04;0.05) | 9.80E-01 |
| Sphingomyelins | -0.09 (-0.14;-0.05) | 2.11E-04 | 0.00 (-0.09;0.08) | 9.57E-01 | 0.00 (-0.08;0.09) | 9.80E-01 |
| Triglycerides in VLDL | 0.03 (-0.01;0.07) | 2.36E-01 | 0.01 (-0.03;0.06) | 7.78E-01 | 0.00 (-0.05;0.04) | 9.80E-01 |
| Free cholesterol in very large HDL | -0.07 (-0.11;-0.02) | 5.03E-03 | -0.01 (-0.05;0.04) | 8.45E-01 | 0.00 (-0.04;0.05) | 9.80E-01 |
| Concentration of very large HDL particles | -0.07 (-0.12;-0.03) | 2.51E-03 | -0.01 (-0.06;0.04) | 7.71E-01 | 0.00 (-0.05;0.05) | 9.80E-01 |
| Phospholipids in chylomicrons and extremely large VLDL | 0.04 (0.00;0.08) | 9.43E-02 | 0.01 (-0.03;0.06) | 7.58E-01 | 0.00 (-0.04;0.05) | 9.80E-01 |
| Concentration of very large VLDL particles | 0.03 (-0.02;0.07) | 3.10E-01 | 0.01 (-0.04;0.05) | 8.12E-01 | 0.00 (-0.05;0.04) | 9.81E-01 |
| Isoleucine | 0.05 (0.00;0.10) | 5.79E-02 | 0.01 (-0.04;0.06) | 7.66E-01 | 0.00 (-0.05;0.05) | 9.81E-01 |
| Docosahexaenoic acid | 0.00 (-0.05;0.04) | 8.75E-01 | 0.01 (-0.04;0.05) | 8.65E-01 | 0.00 (-0.05;0.05) | 9.87E-01 |
| Free cholesterol in very large VLDL | 0.02 (-0.02;0.06) | 5.01E-01 | 0.01 (-0.03;0.06) | 7.78E-01 | 0.00 (-0.04;0.05) | 9.91E-01 |
| Phospholipids in IDL | -0.10 (-0.14;-0.06) | 2.51E-05 | -0.02 (-0.10;0.07) | 8.16E-01 | 0.00 (-0.09;0.09) | 9.92E-01 |
| Cholesteryl esters in medium LDL | -0.04 (-0.09;0.00) | 5.28E-02 | 0.01 (-0.05;0.08) | 8.34E-01 | 0.00 (-0.06;0.07) | 9.92E-01 |
| Polyunsaturated fatty acids | -0.04 (-0.09;0.00) | 5.99E-02 | 0.02 (-0.05;0.08) | 7.78E-01 | 0.00 (-0.06;0.07) | 9.92E-01 |
| Total lipids in lipoprotein particles | -0.06 (-0.10;-0.01) | 1.56E-02 | 0.02 (-0.06;0.09) | 8.12E-01 | 0.00 (-0.08;0.08) | 9.92E-01 |
| Concentration of large VLDL particles | 0.02 (-0.02;0.06) | 3.95E-01 | 0.01 (-0.03;0.06) | 7.78E-01 | 0.00 (-0.05;0.05) | 9.98E-01 |
| Cholesterol in small LDL | -0.06 (-0.10;-0.02) | 5.76E-03 | 0.01 (-0.07;0.09) | 8.92E-01 | 0.00 (-0.08;0.08) | 1.00E+00 |

Model 1 is adjusted for age, sex, and Rotterdam Study Cohort

Model 2 is additionally adjusted for body mass index, smoking status, alcohol use, serum cholesterol, lipid-lowering drugs, and prevalent cardiovascular disease

Model 3 is additionally adjusted for hypertension and diabetes

All serum metabolites and urine ACR are log-transformed.

* P-values are FDR-corrected

Abbreviations: ACR = albumin-to-creatinine ratio; CI = confidence interval; HDL = high-density lipoprotein; IDL = Intermediate-density lipoprotein; LDL = low-density lipoprotein; VLDL = very low-density lipoprotein.

**Supplemental Table 6.** Association between serum metabolites from the Metabolon platform and urine albumin-to-creatinine ratio at baseline (n = 1,290)

|  | **Model 1** |  | **Model 2** |  | **Model 3** |  |
| --- | --- | --- | --- | --- | --- | --- |
| **Metabolite** | **Beta (95% CI)** | **P-value** | **Beta (95% CI)** | **P-value** | **Beta (95% CI)** | **P-value** |
| Perfluorooctanoate (PFOA) | -0.33 (-0.44;-0.23) | 1.36E-06 | -0.31 (-0.42;-0.21) | 1.85E-05 | -0.32 (-0.43;-0.21) | 1.35E-05 |
| X-12707 | 0.24 (0.14;0.35) | 7.54E-04 | 0.28 (0.18;0.39) | 6.51E-05 | 0.28 (0.17;0.38) | 8.85E-05 |
| X-13553 | 0.35 (0.21;0.49) | 5.46E-04 | 0.36 (0.22;0.50) | 3.23E-04 | 0.35 (0.21;0.49) | 6.29E-04 |
| X-25371 | 0.35 (0.20;0.50) | 7.54E-04 | 0.36 (0.20;0.51) | 1.28E-03 | 0.37 (0.21;0.52) | 6.64E-04 |
| 3-(3-Amino-3-Carboxypropyl)Uridine* | 0.40 (0.23;0.58) | 9.08E-04 | 0.38 (0.21;0.56) | 2.51E-03 | 0.38 (0.21;0.56) | 3.75E-03 |
| 4-Hydroxychlorothalonil | -0.28 (-0.38;-0.17) | 1.00E-04 | -0.24 (-0.34;-0.14) | 1.40E-03 | -0.23 (-0.33;-0.12) | 3.75E-03 |
| Orotidine | 0.19 (0.11;0.27) | 7.54E-04 | 0.17 (0.09;0.26) | 2.51E-03 | 0.17 (0.09;0.25) | 4.04E-03 |
| O-Cresol Sulfate | 0.12 (0.06;0.17) | 1.81E-03 | 0.13 (0.07;0.19) | 2.51E-03 | 0.13 (0.07;0.19) | 4.04E-03 |
| 1,5-Anhydroglucitol (1,5-AG) | -0.31 (-0.44;-0.18) | 5.38E-04 | -0.28 (-0.41;-0.15) | 2.41E-03 | -0.27 (-0.40;-0.14) | 4.70E-03 |
| C-Glycosyltryptophan | 0.42 (0.22;0.62) | 3.19E-03 | 0.40 (0.20;0.60) | 6.82E-03 | 0.41 (0.22;0.61) | 4.70E-03 |
| N-Carbamoylvaline | 0.20 (0.09;0.30) | 1.46E-02 | 0.20 (0.10;0.31) | 1.33E-02 | 0.22 (0.11;0.32) | 5.48E-03 |
| 3-Hydroxy-2-Methylpyridine Sulfate | 0.09 (0.05;0.14) | 4.79E-03 | 0.10 (0.05;0.15) | 4.25E-03 | 0.10 (0.05;0.14) | 5.48E-03 |
| 4-Vinylguaiacol Sulfate | 0.11 (0.06;0.16) | 2.09E-03 | 0.11 (0.06;0.16) | 6.60E-03 | 0.11 (0.05;0.16) | 8.41E-03 |
| X-25422 | 0.22 (0.12;0.32) | 1.44E-03 | 0.19 (0.09;0.29) | 1.50E-02 | 0.20 (0.10;0.29) | 8.41E-03 |
| Urea | 0.30 (0.14;0.46) | 1.50E-02 | 0.29 (0.13;0.45) | 2.06E-02 | 0.31 (0.15;0.47) | 1.19E-02 |
| 2,3-Dihydroxy-5-Methylthio-4-Pentenoate (DMTPA)* | 0.45 (0.25;0.66) | 1.81E-03 | 0.40 (0.19;0.61) | 1.54E-02 | 0.39 (0.18;0.60) | 1.54E-02 |
| 3-Bromo-5-Chloro-2,6-Dihydroxybenzoic Acid* | -0.19 (-0.28;-0.09) | 9.14E-03 | -0.18 (-0.28;-0.09) | 1.33E-02 | -0.18 (-0.27;-0.08) | 1.54E-02 |
| N-Acetylserine | 0.31 (0.14;0.48) | 1.46E-02 | 0.29 (0.13;0.46) | 2.77E-02 | 0.31 (0.14;0.47) | 1.79E-02 |
| N-Carbamoylalanine | 0.17 (0.08;0.26) | 1.26E-02 | 0.17 (0.07;0.26) | 2.06E-02 | 0.17 (0.08;0.26) | 1.79E-02 |
| Sulfate* | 0.35 (0.17;0.54) | 1.34E-02 | 0.33 (0.14;0.52) | 2.66E-02 | 0.34 (0.16;0.53) | 1.80E-02 |
| N6-Carbamoylthreonyladenosine | 0.28 (0.12;0.44) | 1.89E-02 | 0.27 (0.11;0.43) | 3.25E-02 | 0.29 (0.13;0.44) | 1.91E-02 |
| Pseudouridine | 0.46 (0.19;0.73) | 2.27E-02 | 0.46 (0.20;0.73) | 3.10E-02 | 0.49 (0.22;0.76) | 1.91E-02 |
| 4-Ethylphenylsulfate | 0.09 (0.03;0.14) | 5.71E-02 | 0.10 (0.04;0.16) | 3.48E-02 | 0.10 (0.05;0.16) | 2.36E-02 |
| 3-Hydroxypyridine Sulfate | 0.11 (0.05;0.17) | 1.89E-02 | 0.11 (0.05;0.17) | 1.80E-02 | 0.11 (0.05;0.17) | 2.45E-02 |
| 4-Acetamidobutanoate | 0.19 (0.09;0.30) | 1.71E-02 | 0.18 (0.08;0.29) | 3.10E-02 | 0.19 (0.08;0.29) | 2.87E-02 |
| X-13844 | 0.09 (0.03;0.15) | 4.01E-02 | 0.10 (0.04;0.15) | 3.85E-02 | 0.10 (0.04;0.16) | 3.14E-02 |
| 3-Methyl Catechol Sulfate (1) | 0.11 (0.05;0.18) | 1.71E-02 | 0.11 (0.05;0.18) | 2.66E-02 | 0.11 (0.05;0.17) | 3.15E-02 |
| Myo-Inositol | 0.23 (0.08;0.38) | 5.27E-02 | 0.25 (0.10;0.41) | 3.66E-02 | 0.26 (0.11;0.42) | 3.15E-02 |
| X-17612 | 0.08 (0.03;0.12) | 3.96E-02 | 0.08 (0.03;0.13) | 3.66E-02 | 0.08 (0.03;0.13) | 3.15E-02 |
| N1-Methylinosine | 0.27 (0.13;0.40) | 6.67E-03 | 0.22 (0.09;0.36) | 3.77E-02 | 0.23 (0.09;0.36) | 3.45E-02 |
| Lanthionine | 0.11 (0.05;0.18) | 2.66E-02 | 0.11 (0.05;0.18) | 3.66E-02 | 0.12 (0.05;0.18) | 3.45E-02 |
| 4-Methoxyphenol Sulfate | 0.07 (0.02;0.11) | 6.32E-02 | 0.07 (0.03;0.12) | 3.66E-02 | 0.08 (0.03;0.12) | 3.45E-02 |
| 2-Hydroxyphenylacetate | 0.11 (0.05;0.17) | 1.89E-02 | 0.10 (0.04;0.16) | 3.85E-02 | 0.10 (0.04;0.17) | 3.45E-02 |
| X-24334 | 0.23 (0.12;0.34) | 4.09E-03 | 0.20 (0.09;0.31) | 2.64E-02 | 0.19 (0.07;0.30) | 3.45E-02 |
| X-24344 | 0.08 (0.04;0.13) | 2.02E-02 | 0.09 (0.04;0.15) | 3.10E-02 | 0.09 (0.04;0.14) | 3.45E-02 |
| N-Formylmethionine | 0.37 (0.16;0.59) | 1.99E-02 | 0.36 (0.15;0.57) | 3.48E-02 | 0.35 (0.14;0.57) | 3.48E-02 |
| Citraconate/Glutaconate | 0.11 (0.05;0.18) | 2.02E-02 | 0.11 (0.04;0.18) | 3.48E-02 | 0.11 (0.04;0.17) | 3.56E-02 |
| Sphingomyelin (D18:2/24:2)* | 0.07 (-0.08;0.22) | 6.59E-01 | 0.22 (0.06;0.38) | 9.98E-02 | 0.27 (0.10;0.43) | 3.56E-02 |
| Erythronate* | 0.30 (0.14;0.47) | 1.46E-02 | 0.27 (0.11;0.44) | 3.48E-02 | 0.26 (0.10;0.43) | 3.83E-02 |
| N-Stearoyltaurine | -0.15 (-0.24;-0.07) | 1.89E-02 | -0.14 (-0.22;-0.05) | 4.86E-02 | -0.14 (-0.23;-0.05) | 3.83E-02 |
| 2,6-Dihydroxybenzoic Acid | 0.11 (0.03;0.20) | 8.87E-02 | 0.14 (0.06;0.23) | 3.48E-02 | 0.14 (0.05;0.22) | 3.83E-02 |
| N-Acetylalanine | 0.31 (0.13;0.49) | 2.02E-02 | 0.28 (0.10;0.46) | 5.15E-02 | 0.29 (0.11;0.47) | 4.02E-02 |
| N2,N2-Dimethylguanosine | 0.29 (0.13;0.45) | 1.83E-02 | 0.24 (0.08;0.41) | 6.78E-02 | 0.26 (0.10;0.42) | 4.16E-02 |
| 4-Allylphenol Sulfate | 0.06 (0.00;0.13) | 2.13E-01 | 0.10 (0.04;0.17) | 4.49E-02 | 0.10 (0.04;0.17) | 4.16E-02 |
| Ascorbic Acid 2-Sulfate | 0.13 (0.05;0.22) | 4.02E-02 | 0.14 (0.05;0.22) | 4.49E-02 | 0.14 (0.05;0.22) | 4.16E-02 |
| 4-Vinylcatechol Sulfate | 0.08 (0.03;0.13) | 3.11E-02 | 0.08 (0.03;0.13) | 4.49E-02 | 0.08 (0.03;0.13) | 4.16E-02 |
| X-12026 | 0.20 (0.04;0.36) | 1.15E-01 | 0.24 (0.07;0.41) | 8.25E-02 | 0.27 (0.10;0.44) | 4.16E-02 |
| Ascorbic Acid 3-Sulfate* | 0.16 (0.06;0.26) | 3.72E-02 | 0.16 (0.06;0.26) | 4.00E-02 | 0.16 (0.06;0.26) | 4.22E-02 |
| 3-Ethylcatechol Sulfate (1) | 0.10 (0.04;0.15) | 2.74E-02 | 0.10 (0.04;0.16) | 3.77E-02 | 0.10 (0.04;0.16) | 4.22E-02 |
| 4-Acetylphenol Sulfate | 0.08 (0.01;0.15) | 1.23E-01 | 0.10 (0.04;0.17) | 4.87E-02 | 0.10 (0.04;0.17) | 4.40E-02 |
| Guaiacol Sulfate | 0.14 (0.05;0.23) | 2.98E-02 | 0.14 (0.05;0.23) | 3.77E-02 | 0.14 (0.05;0.22) | 4.40E-02 |
| Dimethylglycine | 0.28 (0.13;0.44) | 1.71E-02 | 0.25 (0.09;0.41) | 4.49E-02 | 0.25 (0.09;0.40) | 4.45E-02 |
| Dopamine 3-O-Sulfate | 0.15 (0.05;0.25) | 5.45E-02 | 0.17 (0.07;0.27) | 3.77E-02 | 0.16 (0.06;0.26) | 4.74E-02 |
| X-12713 | 0.51 (0.18;0.84) | 3.96E-02 | 0.54 (0.21;0.88) | 3.90E-02 | 0.53 (0.19;0.87) | 4.75E-02 |
| X-17676 | 0.18 (0.05;0.31) | 7.75E-02 | 0.20 (0.07;0.33) | 6.15E-02 | 0.20 (0.07;0.33) | 4.76E-02 |
| X-12411 | 0.15 (0.05;0.24) | 3.72E-02 | 0.13 (0.04;0.23) | 7.08E-02 | 0.14 (0.05;0.23) | 4.91E-02 |
| X-24812 | 0.16 (0.07;0.26) | 1.65E-02 | 0.16 (0.05;0.26) | 6.78E-02 | 0.16 (0.05;0.26) | 6.28E-02 |
| Trigonelline (N'-Methylnicotinate) | 0.09 (0.03;0.16) | 7.23E-02 | 0.09 (0.03;0.16) | 8.91E-02 | 0.10 (0.03;0.16) | 6.30E-02 |
| X-25790 | 0.13 (0.03;0.24) | 1.08E-01 | 0.15 (0.04;0.26) | 9.98E-02 | 0.16 (0.05;0.27) | 6.30E-02 |
| X-12216 | 0.06 (-0.01;0.12) | 2.86E-01 | 0.09 (0.03;0.15) | 8.35E-02 | 0.09 (0.03;0.15) | 6.38E-02 |
| 4-Hydroxyphenylacetylglutamine | 0.15 (0.06;0.23) | 2.08E-02 | 0.13 (0.04;0.21) | 6.97E-02 | 0.13 (0.04;0.21) | 6.80E-02 |
| Vanillylmandelate (VMA) | 0.17 (0.02;0.32) | 1.70E-01 | 0.21 (0.06;0.36) | 9.65E-02 | 0.23 (0.07;0.38) | 6.80E-02 |
| Glucuronate | 0.18 (0.08;0.28) | 1.81E-02 | 0.15 (0.05;0.25) | 6.97E-02 | 0.15 (0.05;0.25) | 7.14E-02 |
| Acisoga | 0.20 (0.08;0.32) | 1.99E-02 | 0.18 (0.06;0.29) | 5.26E-02 | 0.17 (0.05;0.28) | 7.15E-02 |
| X-19141 | 0.09 (0.00;0.19) | 2.26E-01 | 0.13 (0.04;0.22) | 9.17E-02 | 0.14 (0.04;0.23) | 7.15E-02 |
| 4-Ethylcatechol Sulfate | 0.08 (0.01;0.15) | 1.30E-01 | 0.10 (0.03;0.17) | 7.57E-02 | 0.10 (0.03;0.16) | 7.45E-02 |
| 3-Aminoisobutyrate | 0.14 (0.03;0.26) | 1.07E-01 | 0.16 (0.04;0.27) | 9.98E-02 | 0.17 (0.05;0.28) | 7.74E-02 |
| 1-Palmitoyl-2-Linoleoyl-GPE (16:0/18:2) | 0.15 (0.04;0.25) | 8.18E-02 | 0.16 (0.05;0.27) | 6.78E-02 | 0.16 (0.05;0.26) | 7.78E-02 |
| 3-Methyl Catechol Sulfate (2) | 0.07 (0.03;0.12) | 3.78E-02 | 0.07 (0.02;0.11) | 7.59E-02 | 0.07 (0.02;0.11) | 7.93E-02 |
| Choline Phosphate | -0.44 (-0.68;-0.20) | 1.66E-02 | -0.35 (-0.60;-0.10) | 8.94E-02 | -0.35 (-0.60;-0.11) | 7.98E-02 |
| Lyxonate | 0.14 (0.04;0.24) | 7.11E-02 | 0.14 (0.04;0.23) | 9.17E-02 | 0.14 (0.04;0.24) | 8.01E-02 |
| Gluconate | 0.15 (0.05;0.24) | 5.35E-02 | 0.14 (0.04;0.24) | 8.35E-02 | 0.14 (0.04;0.24) | 8.05E-02 |
| Arabonate/Xylonate | 0.15 (0.04;0.26) | 8.48E-02 | 0.15 (0.04;0.26) | 9.17E-02 | 0.15 (0.05;0.26) | 8.18E-02 |
| Glcnac Sulfate Conjugate Of C21H34O2 Steroid** | 0.20 (0.06;0.34) | 7.03E-02 | 0.21 (0.06;0.35) | 8.18E-02 | 0.20 (0.06;0.35) | 8.18E-02 |
| N-(2-Furoyl)Glycine | 0.09 (0.04;0.15) | 2.74E-02 | 0.09 (0.03;0.14) | 6.78E-02 | 0.08 (0.02;0.14) | 8.37E-02 |
| Sphingomyelin (D18:1/20:2, D18:2/20:1, D16:1/22:2)* | 0.11 (-0.03;0.25) | 3.57E-01 | 0.16 (0.02;0.30) | 1.77E-01 | 0.20 (0.06;0.34) | 8.37E-02 |
| Sphingomyelin (D18:1/22:2, D18:2/22:1, D16:1/24:2)* | 0.06 (-0.10;0.22) | 7.20E-01 | 0.19 (0.03;0.35) | 1.80E-01 | 0.23 (0.07;0.39) | 8.37E-02 |
| Glycosyl-N-(2-Hydroxynervonoyl)-Sphingosine (D18:1/24:1(2OH))* | 0.13 (0.03;0.24) | 1.07E-01 | 0.14 (0.03;0.24) | 1.20E-01 | 0.15 (0.04;0.25) | 8.37E-02 |
| Methylsuccinoylcarnitine | 0.11 (0.02;0.21) | 1.38E-01 | 0.11 (0.02;0.21) | 1.70E-01 | 0.13 (0.04;0.23) | 8.57E-02 |
| N-Acetylneuraminate | 0.21 (0.06;0.36) | 7.11E-02 | 0.19 (0.04;0.34) | 1.27E-01 | 0.20 (0.06;0.35) | 8.57E-02 |
| Quinolinate | 0.17 (0.05;0.28) | 6.36E-02 | 0.15 (0.04;0.27) | 1.20E-01 | 0.16 (0.05;0.28) | 8.57E-02 |
| Methylsuccinate | 0.15 (0.04;0.26) | 8.11E-02 | 0.15 (0.04;0.26) | 1.20E-01 | 0.16 (0.04;0.27) | 8.57E-02 |
| X-23655 | 0.09 (0.03;0.16) | 4.54E-02 | 0.09 (0.03;0.16) | 6.78E-02 | 0.09 (0.02;0.15) | 8.57E-02 |
| 17alpha-Hydroxypregnanolone Glucuronide | 0.09 (0.01;0.16) | 1.38E-01 | 0.10 (0.03;0.17) | 9.98E-02 | 0.10 (0.03;0.17) | 8.66E-02 |
| N-Acetyl-Isoputreanine | 0.16 (0.04;0.28) | 9.51E-02 | 0.16 (0.04;0.28) | 1.13E-01 | 0.17 (0.05;0.28) | 8.66E-02 |
| Hydroxy-N6,N6,N6-Trimethyllysine* | 0.20 (0.07;0.34) | 5.26E-02 | 0.18 (0.04;0.31) | 1.22E-01 | 0.19 (0.05;0.32) | 8.90E-02 |
| Homovanillate (HVA) | 0.14 (0.04;0.25) | 7.75E-02 | 0.14 (0.04;0.25) | 9.98E-02 | 0.14 (0.04;0.25) | 8.90E-02 |
| N-Acetylthreonine | 0.23 (0.07;0.39) | 6.93E-02 | 0.21 (0.05;0.37) | 1.20E-01 | 0.22 (0.06;0.38) | 9.27E-02 |
| N6,N6-Dimethyllysine | 0.09 (0.02;0.16) | 1.08E-01 | 0.09 (0.02;0.16) | 1.40E-01 | 0.09 (0.03;0.16) | 9.27E-02 |
| Ectoine | 0.08 (0.01;0.15) | 1.38E-01 | 0.09 (0.02;0.16) | 1.37E-01 | 0.10 (0.03;0.17) | 9.30E-02 |
| N-Acetylmethionine | 0.24 (0.06;0.41) | 9.03E-02 | 0.23 (0.05;0.41) | 1.37E-01 | 0.24 (0.07;0.42) | 9.30E-02 |
| Dimethyl Sulfone | 0.39 (0.11;0.68) | 7.75E-02 | 0.36 (0.07;0.65) | 1.43E-01 | 0.40 (0.10;0.69) | 9.85E-02 |
| 5,6-Dihydrouridine | 0.31 (0.08;0.54) | 7.74E-02 | 0.28 (0.05;0.51) | 1.49E-01 | 0.30 (0.08;0.53) | 9.94E-02 |
| Beta-Hydroxyisovaleroylcarnitine | 0.20 (0.04;0.36) | 1.03E-01 | 0.20 (0.04;0.35) | 1.42E-01 | 0.21 (0.05;0.36) | 9.94E-02 |
| Cis-4-Decenoylcarnitine (C10:1) | 0.14 (0.03;0.24) | 9.03E-02 | 0.15 (0.04;0.25) | 9.17E-02 | 0.14 (0.04;0.25) | 9.94E-02 |
| 3-Methoxytyramine Sulfate | 0.15 (0.04;0.25) | 7.75E-02 | 0.15 (0.04;0.25) | 9.98E-02 | 0.14 (0.04;0.25) | 9.94E-02 |
| Perfluorooctanesulfonate (PFOS) | -0.16 (-0.26;-0.06) | 3.96E-02 | -0.13 (-0.23;-0.04) | 1.10E-01 | -0.13 (-0.23;-0.03) | 9.94E-02 |
| X-22509 | 0.03 (-0.01;0.07) | 3.35E-01 | 0.05 (0.01;0.09) | 1.20E-01 | 0.05 (0.01;0.09) | 9.94E-02 |
| Etiocholanolone Glucuronide | 0.06 (0.00;0.12) | 2.77E-01 | 0.09 (0.03;0.16) | 9.65E-02 | 0.09 (0.02;0.15) | 1.02E-01 |
| X-12847 | 0.06 (0.01;0.11) | 9.51E-02 | 0.06 (0.01;0.11) | 1.37E-01 | 0.07 (0.02;0.12) | 1.02E-01 |
[truncated: 152,185 more chars]
